# EAES and SAGES 2018 consensus conference on acute diverticulitis management: evidence-based recommendations for clinical practice

**DOI:** 10.1007/s00464-019-06882-z

**Published:** 2019-06-27

**Authors:** Nader K. Francis, Patricia Sylla, Maria Abou-Khalil, Simone Arolfo, David Berler, Nathan J. Curtis, Scott C. Dolejs, Richard Garfinkle, Marguerite Gorter-Stam, Daniel A. Hashimoto, Taryn E. Hassinger, Charlotte J. L. Molenaar, Philip H. Pucher, Valérie Schuermans, Alberto Arezzo, Ferdinando Agresta, Stavros A. Antoniou, Tan Arulampalam, Marylise Boutros, Nicole Bouvy, Kenneth Campbell, Todd Francone, Stephen P. Haggerty, Traci L. Hedrick, Dimitrios Stefanidis, Mike S. Truitt, Jillian Kelly, Hans Ket, Brian J. Dunkin, Andrea Pietrabissa

**Affiliations:** 1 Department of General Surgery, Yeovil District Hospital NHS Foundation Trust, Yeovil, UK; 2 Division of Colon and Rectal Surgery, Mount Sinai Hospital, New York, NY USA; 3 Division of Colon and Rectal Surgery, Jewish General Hospital, McGill University, Montreal, Canada; 4 Department of Surgical Sciences, University of Torino, Toriano, Italy; 5 Department of Surgery and Cancer, Imperial College London, London, UK; 6 Department of Surgery, Indiana University School of Medicine, Indianapolis, IN USA; 7 Department of Surgery, VU University Medical Center, Amsterdam, The Netherlands; 8 Department of Surgery, Massachusetts General Hospital, Boston, MA USA; 9 Department of Surgery, The University of Virginia Health System, Charlottesville, VA USA; 10 Department of Surgery, Spaarne Gasthuis, Hoofddorp, The Netherlands; 11 Department of General Surgery, Portsmouth Hospitals NHS Trust, Portsmouth, UK; 12 Department of Surgery, Maastricht University Medical Centre, Maastricht, The Netherlands; 13 Department of General Surgery, ULSS5 del Veneto, Adria, Italy; 14 Department of Surgery, European University of Cyprus, Nicosia, Cyprus; 15 Department of General Surgery, Colchester General Hospital, Colchester, UK; 16 Department of Surgery, Ninewells Hospital, Dundee, UK; 17 School of Medicine, University of Dundee, Dundee, UK; 18 Division of Colon & Rectal Surgery, Newton-Wellesley Hospital, Newton, MA USA; 19 Division of General Surgery, NorthShore University Health System, Evanston, IL USA; 20 Pritzker School of Medicine, University of Chicago, Chicago, IL USA; 21 Methodist Dallas Medical Center, Colchester, TX USA; 22 SAGES, Los Angeles, CA USA; 23 Department of Surgery, Houston Methodist Hospital, Houston, TX USA; 24 Department of Surgery, Fondazione IRCCS Policlinico San Matteo, Pavia, Italy

**Keywords:** Diverticulitis, Acute, Elective surgery, Emergency surgery, Lavage, Consensus, Guidelines

## Abstract

**Background:**

Acute diverticulitis (AD) presents a unique diagnostic and therapeutic challenge for general surgeons. This collaborative project between EAES and SAGES aimed to summarize recent evidence and draw statements of recommendation to guide our members on comprehensive AD management.

**Methods:**

Systematic reviews of the literature were conducted across six AD topics by an international steering group including experts from both societies. Topics encompassed the epidemiology, diagnosis, management of non-complicated and complicated AD as well as emergency and elective operative AD management. Consensus statements and recommendations were generated, and the quality of the evidence and recommendation strength rated with the GRADE system. Modified Delphi methodology was used to reach consensus among experts prior to surveying the EAES and SAGES membership on the recommendations and likelihood to impact their practice. Results were presented at both EAES and SAGES annual meetings with live re-voting carried out for recommendations with < 70% agreement.

**Results:**

A total of 51 consensus statements and 41 recommendations across all six topics were agreed upon by the experts and submitted for members’ online voting. Based on 1004 complete surveys and over 300 live votes at the SAGES and EAES Diverticulitis Consensus Conference (DCC), consensus was achieved for 97.6% (40/41) of recommendations with 92% (38/41) agreement on the likelihood that these recommendations would change practice if not already applied. Areas of persistent disagreement included the selective use of imaging to guide AD diagnosis, recommendations against antibiotics in non-complicated AD, and routine colonic evaluation after resolution of non-complicated diverticulitis.

**Conclusion:**

This joint EAES and SAGES consensus conference updates clinicians on the current evidence and provides a set of recommendations that can guide clinical AD management practice.

**Electronic supplementary material:**

The online version of this article (10.1007/s00464-019-06882-z) contains supplementary material, which is available to authorized users.

Symptomatic left-sided colonic diverticular disease is a common clinical entity with rising prevalence, healthcare costs and societal impact in industrialized countries. Acute diverticulitis (AD), which ranges in severity from minor subclinical episodes to major complications, presents diagnostic and therapeutic challenges. While the overarching goal in treatment is to minimize morbidity, a number of factors influence the management of AD besides clinical severity and co-morbidities. Variations in institutional protocols and resources, specialty training, surgical expertise, as well as societal and patient expectations regarding cost of care and quality of life, may impact clinical management and subsequent outcomes. Several surgical and medical professional societies have recognized the need to standardize the management of AD based on metrics previously identified as clinically relevant in several guidelines and consensus statements published over the past two decades. Evidence-based recommendations regarding the management of AD are often conflicting primarily due to the lack of good quality data, and regional variations in AD practices [1].

Since the 1999 European Association for Endoscopic Surgery (EAES) AD consensus conference, more recent data regarding the epidemiology, diagnosis, non-operative management and surgical techniques including the use of minimally invasive strategies have been published, warranting an update of AD management guidelines [2]. The aim of this joint EAES and Society of American Gastrointestinal and Endoscopic Surgeons (SAGES) collaborative consensus conferences was to generate an updated and comprehensive set of evidence-based AD management recommendations. This consensus conference was also designed to canvass the wider opinion of both memberships regarding the statements and recommendations to maximize potential uptake and impact on practice.

## Methods

A steering group comprising of 24 experts and residents from SAGES and EAES, two project leads, and two librarians was assembled. The subject of acute diverticulitis was divided into six main topics: (i) epidemiology, (ii) diagnosis and classification, iii) non-resectional management of uncomplicated AD, (iv) non-resectional management of complicated AD, (v) emergency operative management of AD, and (iv) elective operative management of AD. Research questions for each topic were formulated, revised, and unanimously approved by all experts. The steering group was divided into six teams each composed of a senior mentor and surgical resident from each society who were assigned one topic (Table 1). Each team conducted their literature research and drafted statements and recommendations on their research questions. The literature review process conformed to PRISMA statement standards for systematic reviews and meta-analyses [3].

**Table 1:** Steering group members and topic allocations

Team & topic	Experts		Residents	
Epidemiology and natural history	EAES	Ferdinando Agresta	EAES	Valérie Schuermans
	SAGES	Steve Hagerty	SAGES	David Berler
Diagnosis and classification	EAES	Nicole Bouvy	EAES	Charlotte Molenaar
	SAGES	Dimitrios Stefanidis	SAGES	Scott Dolejs
Uncomplicated acute diverticulitis	EAES	Tan Arulampalam	EAES	Marguerite Gorter-Stam
	SAGES	Marylise Boutros	SAGES	Richard Garfinkle
Complicated acute diverticulitis	EAES	Alberto Arezzo	EAES	Nathan Curtis
	SAGES	Todd Francone	SAGES	Dan Hashimoto
Emergency surgery	EAES	Kenneth Campbell	EAES	Simone Arolfo
	SAGES	Mike Truitt	SAGES	Maria Abou-Khalil
Elective surgery	EAES	Stavros Antoniou	EAES	Philip Pucher
	SAGES	Traci Hedrick	SAGES	Taryn Hassinger
Project leads	EAES	Nader Francis	SAGES	Patricia Sylla
Project mentors	EAES	Andrea Pietrabissa	SAGES	Brian Dunkin

### Search methods and inclusion criteria

Based on the research questions, the literature search was designed and performed by two certified EAES and SAGES librarians. The PubMed, Embase and CENTRAL databases were queried between October 26th 2017 and November 8th 2017. Study inclusion criteria were systematic reviews, randomized clinical trials, cohort studies and case series of more than ten patients on the subject of colonic diverticulitis and diverticulosis involving the bowel distal to the splenic flexure published in the English language after 1998. Animal studies, case reports, narrative reviews, commentaries and studies on diverticular disease affecting the bowel proximal to the splenic flexure were excluded. Search syntaxes are displayed in Appendix 1.

### Manuscript selection

Prior to reviewing search results, a calibration session was held for all participants and librarians to standardize article selection within and across teams. The two residents from each team independently reviewed their articles from their topic using freely available Rayyan QCRI electronic platform [4]. All decisions were recorded and disagreements between reviewers regarding articles’ inclusion or exclusion were resolved by their respective topic experts during a live discussion. The free, open source Abstrackr web-based citation screening tool was used to screen all abstracts.

### Content summary and grading of included articles

Structured summaries were generated for each included article. The quality of evidence on each outcome was assessed according to the Grades of Recommendation, Assessment, Development, and Evaluation (GRADE) methodology and predefined criteria, including study quality, inconsistency, indirectness, imprecision, reporting bias, strong evidence of association and effect of confounders. The level of evidence was rated as high, moderate, low, or very low quality [5].

### Drafting statement and recommendations and assigning strength

With the exception of Topic 1 where no recommendations regarding AD epidemiology could be made, statements and recommendations were generated in response to each topic question based on the literature review, using GRADE criteria for assigning strength. All were presented at a steering group meeting in London January 2018 attended by all experts, mentors, project leads and residents. The content and strength of each statement and recommendation (strong for/against using an intervention; weak for/against using an intervention) were reviewed taking into account the quality of the supporting evidence, as well as risks versus benefits, patient values and preferences, and expert opinions.

A modified Delphi methodology was followed to reach agreement among the experts on all statements and recommendations [6]. Each was subjected to live voting by the experts using the Pollev electronic platform (https://www.polleverywhere.com). When unanimous consensus was not achieved, supporting evidence from included articles was presented. After group discussion and, if necessary, modification of the statements and/or recommendations, a second round of voting was carried out. The statements and recommendations were approved only if ≥ 70% expert agreement was achieved.

### Membership survey

To reach wider consensus as well as assess potential uptake of recommendations and impact on surgical practice, the final draft of consensus statements and recommendations were emailed to the EAES and SAGES memberships between March and May 2018 using Survey Monkey™ (San Mateo, CA, USA). Members were asked to anonymously vote on each recommendation and indicate if they agreed (Yes; No), and whether that recommendation might alter their current practice (Yes, No or already my current practice).

### Data analysis and final consensus development

Based on the survey results, ≥ 70% “yes” was categorized as agreement with a given statement of recommendation. Agreement on the likelihood to change practice was defined as ≥ 70% stating their intent to change practice or that this already formed their current practice. When agreement on a given recommendation and likelihood to change practice was both achieved, this was categorized as consensus among the membership. These results were presented (with no further voting) at the SAGES and EAES Diverticulitis Consensus Conferences (DCC) sessions held during the SAGES 2018 Annual Meeting in Seattle on April 12, 2018 and the 26th EAES Congress in London on June 1, 2018.

When agreement was not achieved on either the recommendation or likelihood to change practice, live re-voting was carried out during the SAGES and EAES DCCs. Following a brief summary of the literature used to generate the statement and recommendation, the audience was asked to vote. Live voting was moderated by the chairs of the sessions [Pollev for SAGES and CrowdComms Elements for EAES (www.crowdcomms.com)]. Live voting results were immediately displayed to the audience and recorded to generate the final consensus manuscript.

## Results

The literature searches yielded 8418 articles across the six topics. After title and abstract screening, 570 full text articles were included leading to the generation of 132 initial statements and recommendations across the six topics. All PRISMA diagrams are displayed shown in Supplementary Fig. 1.

At the London Steering meeting, 51 statements were dropped and 25 were merged to minimize redundancy. Additional statements, such as those regarding AD epidemiology and classification, did not lend themselves to recommendations, while for other statements, no evidence could be identified to generate recommendations. Ultimately, 51 statements and 41 recommendation were submitted for online voting by the EAES and SAGES membership. The full text literature analyses and references used to generate statements and recommendations for all six topics are included as Supplementary Materials 1–6.

A total of 1004 completed membership survey responses were received. Initial agreement was recorded for 97.5% (40/41) of recommendations and 88% (36/41) on likelihood to change current practice and or already current practice (Table 2). When combining live re-voting results from the SAGES and EAES DCCs, disagreement with the recommendation to consider non-antibiotic therapy in immunocompetent patients with non-complicated AD persisted. Agreement on the likelihood to change current practice and/or already current practice was reached for two additional recommendations with overall consensus in 92% (38/41. Consensus on the willingness to change practice could not be achieved with respect to consideration for selective imaging in patients with pain localized to the left lower quadrant, absence of vomiting, and CRP > 50 mg/L, trial of non-antibiotic therapy in immunocompetent patients with non-complicated AD, and against performing routine colonic evaluation after treatment of uncomplicated AD.

**Table 2:** Summary of generated statements and recommendations for each topic and individual subtopic where consensus was not reached

Acute diverticulitis topic	# Expert statements and recommendations	Disagreement with recommendation (survey)	Disagreement with practice/change in practice	Disagreement with recommendation (meeting)	Disagreement with practice/change in practice (meeting)	# Recommendations with final consensus
1 Epidemiology and natural history	13 Statements 0 Recommendations	Not applicable				
2 Diagnosis and classifications	5 Statements 4 Recommendations	0	2.2b -CRP 2.2c -Selective imaging post AD	N/A	2.2c - SAGES and EAES	3
3 Uncomplicated	5 Statements 5 Recommendations	3.2—trial of non-ABX in uncomplicated AD	3.2—trial of non-ABX in uncomplicated AD 3.4—selective colonic evaluation post AD	3.2 (SAGES only)	3.2—SAGES only 3.4—SAGES only	3
4 Complicated	10 Statements 10 Recommendations	0	0	N/A	N/A	10
5 Emergency surgery	7 Statements 6 Recommendations	0	0	N/A	N/A	6
6 Elective surgery	11 Statements 16 Recommendations	0	0	N/A	N/A	16
Total	51 Statements 41 Recommendations	1	4	1	3	38

The number of Asterix (*) denotes the number of statements for which no evidence was identified in the literature searches (Q5.4, Q6.3, Q6.6, Q6.8 and Q6.10)

For each AD topic, the consensus statements and recommendations, initial survey results and final agreement or disagreements are shown below. Recommendations that did not reach ≥ 70% membership agreement and/or ≥ 70% of respondents willing to change practice or currently practicing the recommendation are summarized in Tables 2 and 3.

**Table 3:** Voting and outcome data for the statements and recommendations where initial consensus was not reached

Topic	Statement and Recommendation	Strength	Member agreement with recommendation	Likelihood to change practice	Further voting and final verdict
Topic 2: diverticulitis diagnosis and classification	Selective imaging in patients with pain localized to the left lower quadrant, absence of vomiting, a CRP > 50 mg/L, and/or a prior history of acute diverticulitis We recommend selective imaging in patients with pain localized to the left lower quadrant, absence of vomiting, a CRP > 50 mg/L, and/or a prior history of acute diverticulitis (LoE: moderate. Strength: weak)	Weak	76.25%	401 members (42.52%) already their current practice 170 (18%) agreed that this likely to change their practice 372 (39.45%) disagreed that it was likely to change their practice	Likelihood to change practice SAGES meetings Yes 53% (112/210) EAES meeting Yes 36% (42/114) Consensus was achieved on the recommendation but not the likelihood to change practice
	Numerous studies have demonstrated the diagnostic and prognostic value of C-reactive protein for patients with acute diverticulitis We recommend that CRP be included in the laboratory evaluation of a patient with acute diverticulitis. (LoE: moderate. Strength: strong)	Strong	78.3%	367 members (37%) already their current practice 325 (32.7%) agreed that this likely to change their practice 299 (30.1%) disagreed that it was likely to change their practice	Likelihood to change practice SAGES meeting Yes 83.3% (175/210) EAES meetings 75% (90/120) Consensus achieved on likelihood to change practice with the 2nd round of voting
Topic 3: Non-resection management of uncomplicated diverticulitis	In immunocompetent individuals presenting with uncomplicated acute diverticulitis, symptomatic treatment without antibiotics provides similar outcomes to treatment with antibiotics A trial of non-antibiotic therapy can be considered with appropriate follow-up in select immunocompetent individuals presenting with uncomplicated acute diverticulitis (LoE: high. Strength: weak)	Weak	59.12%	228 members (26.48%) already their current practice 199 (23.11%) agreed that this likely to change their practice 434 (50.41%) disagreed that it was likely to change their practice	Recommendation SAGES meeting yes 61.7% (129/209) EAES meetings yes 70.8% (90/127) Likelihood to change practice SAGES meeting yes 46% (98/212) EAES meeting yes 71% (80/122) Consensus was not reached on the recommendation nor its likelihood to change practice
	In the absence of high-risk features, the detection rate for advanced adenomas or malignant lesions with colonic evaluation after an episode of uncomplicated acute diverticulitis is very low Our expert group recommends against routine colonic evaluation after successfully treated uncomplicated acute diverticulitis, unless high-risk features are present. (LoE: moderate. Strength: weak)	Moderate	73.96%	389 members (48.9%) already their current practice 147 (18.5%) agreed that this likely to change their practice 259 (38.6%) disagreed that it was likely to change their practice.	Likelihood to change practice SAGES meeting yes 46% (98/212) EAES meeting yes 71% (80/122) Consensus was reached on the recommendation, but not its likelihood to change practice
Topic 4: non-resectional management of complicated acute diverticulitis	Laparoscopic lavage has been shown to decrease stoma formation rate without impacting one-year mortality, although short-term morbidity may be increased. There is no consensus on an effective laparoscopic lavage technique Lavage may be considered in selected Hinchey III patients by surgeons with appropriate expertise and the ability to closely watch for and manage complications. The lower stoma rate should be weighed against the higher risk of complications and re-intervention. (LoE: high. Strength: weak)	High	79.97%	358 members (49.1%) already their current practice 130 (17.8%) agreed that this likely to change their practice 241 (33.1%) disagreed that it was likely to change their practice	Likelihood to change practice SAGES meeting yes 76% (152/200) EAES meetings yes 70.3% (83/118) Consensus achieved on likelihood to change practice with the 2nd round of voting

≥70% “yes” was categorized as agreement with a given recommendation. Agreement on the likelihood to change practice was defined as ≥ 70% stating their intent to change practice or that this was already their current practice. When agreement on a given recommendation and likelihood to change practice was both achieved, this was categorized as consensus. Re-voting outcomes are shown in the right-hand column

### Topic 1: Epidemiology of acute diverticulitis

The full systematic review for this topic and reference list are provided in supplementary materials.

Q1.1: *What is the incidence and prevalence of left*-*sided acute diverticulitis?*

**Statement:** Admission rates for left-sided acute diverticulitis are increasing. The highest rates of increase are occurring in those under 40 years of age. (Level of Evidence (LoE) moderate. No recommendation).

**Statement:** In patients over the age of 50, acute diverticulitis occurs more frequently in females; in those under 50 years of age, it occurs more commonly in males (LoE moderate. No recommendation).

**Statement:** Individuals from regions where the prevalence of diverticulitis is low experience a gradual increase in incidence following migration to the west and acculturation to the western lifestyle. (LoE low. No recommendation).

**Statement:** There is seasonal and geographic variation in the prevalence of acute diverticulitis which has been replicated in all hemispheres, with peak incidence occurring in the summer months. (LoE moderate. No recommendation).

*Q1.2: What factors are associated with an increased risk of developing acute diverticulitis?*

**Statement:** Long-term NSAID, corticosteroid, and opiate use have been associated with increased risk of perforation in the setting of acute left-sided diverticulitis. (LoE low. No recommendation).

**Statement:** Calcium channel blocker and statin therapy may lower the risk for colonic perforation and the need for emergency surgery in patients with symptomatic diverticular disease. (LoE moderate. No recommendation).

**Statement:** Increased BMI and increased visceral-to-subcutaneous fat ratio are associated with an increased risk for acute diverticulitis (LoE moderate. No recommendation).

**Statement:** Physical activity is associated with a diminished risk of complicated diverticulitis. (LoE moderate. No recommendation).

**Statement:** Smoking has been associated with increased risk of acute diverticulitis. (LoE moderate. No recommendation).

*Q1.3: Are there any other risk factors in specific patient groups?*

**Statement:** Ehler-Danlos, Marfan’s, and Williams-Beuren syndromes are associated with an increased risk of the development of acute diverticulitis. (LoE: low. No recommendation).

**Statement:** Patients with HIV and those undergoing chemotherapy are at increased risk for developing acute diverticulitis. (LoE: moderate. No recommendation).

*Q1.4: What is the microbiome profile in acute diverticulitis?*

**Statement:** Bifidobacteria and phylum proteobacteria are more abundant in patients presenting with acute diverticulitis (LoE: low. No recommendation).

**Statement:** H. pylori infection may confer protection against the development of complications in those with diverticular disease. (LoE: low. No recommendation).

### Topic 2: Diagnosis and classification of acute diverticulitis

The full systematic review for this topic and reference list are provided in supplementary materials.

*Q2.1 What are the classification systems for acute diverticulitis?*

**Statement:** There are multiple classification systems for acute diverticulitis. None has been conclusively demonstrated to be superior to another in predicting patient outcomes, and therefore, a specific recommendation cannot be provided. (LoE: low. No recommendation).

Given that the modified Hinchey classification has received the most attention in the literature and has been used in several clinical trials on diverticulitis it will be used for the purposes of these guidelines.

*Q2.2 How is the diagnosis of acute diverticulitis best established?*

**Statement:** Left-lower quadrant abdominal pain and tenderness in the absence of vomiting are the clinical features most consistent with diverticulitis.

**Recommendation:** A focused history and physical exam are recommended for all patients with suspected diverticulitis. (LoE: moderate. Strength of recommendation: strong).

Membership survey results: Yes 998 (97.75%).

743 members (72.7%) agreed that this recommendation was already their current practice; 67 (6.5%) agreed that this recommendation was likely to change their practice while 211 (20.6%) disagreed that it was likely to change their practice.

Consensus was reached with no further voting.

**Statement:** Numerous studies have demonstrated the diagnostic and prognostic value of C-reactive protein for patients with acute diverticulitis.

**Recommendation:** We recommend that CRP be included in the laboratory evaluation of a patient with acute diverticulitis. (LoE: moderate. Strength of recommendation: strong).

Membership survey results: Yes 776 (78.3%).

367 members (37%) agreed that this recommendation was already their current practice; 325 (32.8%) agreed that it was likely change their practice while 299 (30.1%) disagreed that it would change their practice. Since a consensus was not achieved, re-voting was carried out at SAGES and EAES meetings with 83.3% (175/210) and 75% (90/120) agreement respectively to change practice if not already practicing the recommendation. Consensus was reached with the second round of voting.

**Statement:** Patients with pain localized to the left lower quadrant, absence of vomiting, and a CRP > 50 mg/l are highly likely to have acute diverticulitis.

**Recommendation:** We recommend selective imaging in patients with pain localized to the left lower quadrant, absence of vomiting, a CRP > 50 mg/L, and/or a prior history of acute diverticulitis.

(LoE: moderate. Strength of recommendation: weak).

Membership survey results: Yes 719 (76.25%).

401 members (42.52%) agreed that this recommendation was already their current practice; 170 (18%) agreed that this recommendation was likely to change their practice while 372 (39.45%) disagreed that it was likely to change their practice. Since no consensus was obtained, re-voting was carried out at the SAGES and EAES meetings with only 53% (112/210) and 36% (42/114) positive responses received at both conferences, respectively. Consensus was achieved on the recommendation but not on the likelihood to change practice.

**Statement:** When imaging is deemed necessary, the recommended modality of choice is CT scan. Alternatively, ultrasound at centers with expertise in that modality could be used.

**Recommendation:** Our expert group rated this high-quality evidence with strong recommendation for using CT scan as the recommended modality of choice. Alternatively, ultrasound at centers with expertise in that modality could be used. (LoE: high. Strength of recommendation: strong).

Membership survey results: Yes 891 (96.74%).

736 members (79.9%) agreed that this recommendation was already their current practice; 66 (7.17%) agreed that his recommendation was likely to change their practice while 119 (12.9%) disagreed that it was likely to change their practice.

Consensus was reached with no further voting.

### Topic 3: Non-resection management of uncomplicated diverticulitis

The full systematic review for this topic and reference list are provided in supplementary materials.

*Q3.1 What are the risk factors for developing recurrent diverticulitis among patients with uncomplicated acute diverticulitis?*

**Statement:** In patients successfully treated for uncomplicated acute diverticulitis, the most commonly reported risk factors for the development of recurrent diverticulitis are young age (< 50) and previous history.

**Recommendation:** Patients with risk factors for recurrent diverticulitis should be counselled regarding an elevated risk for future episodes and the possible long-term treatment implications. (LoE: low. Strength of recommendation: strong).

Membership survey results: Yes 842 (91.87%).

649 members (72.35%) agreed that this recommendation was already their current practice, 114 (12.71%) agreed that this recommendation was likely to change their practice while 134 (14.94%) disagreed that it was likely to change their practice.

Consensus was reached with no further voting.

*Q3.2 What are the optimal non*-*operative strategies in the management of uncomplicated AD?*

**Statement:** In immunocompetent individuals presenting with uncomplicated acute diverticulitis, symptomatic treatment without antibiotics provides similar outcomes to treatment with antibiotics.

**Recommendation:** A trial of non-antibiotic therapy can be considered with appropriate follow-up in select immunocompetent individuals presenting with uncomplicated acute diverticulitis. (LoE: high. Strength of recommendation: weak).

Membership survey results: Yes 509 (59.12%).

228 members (26.48%) agreed that this recommendation was already their current practice; 199 (23.11%) agreed that this recommendation was likely to change their practice while 434 (50.41%) disagreed that it was likely to change their practice.

Since there was no majority agreement on either the statement of recommendation nor the likelihood that the recommendation would change practice, further voting was carried out at SAGES and EAES meetings. Agreement with the recommendation was achieved in 61.7% (129/209) and 70.8% (90/127) and with likelihood to change practice in 46% (98/212) and 71% (80/122) among SAGES and EAES members, respectively.

Consensus was not reached on the recommendation nor its likelihood to change practice.

**Statement:** Immunocompetent individuals presenting with uncomplicated acute diverticulitis may be managed in the outpatient setting.

**Recommendation:** Immunocompetent individuals presenting with uncomplicated acute diverticulitis and mild symptoms may be managed in the outpatient setting. (LoE: moderate. Strength of recommendation: weak).

Membership survey results: Yes 744 (87.94%).

552 members (65.25%) agreed that this recommendation was already their current practice; 118 (13.95%) agreed that this recommendation was likely to change their practice while 176 (20.80%) disagreed that it was likely to change their practice.

Consensus was reached with no further voting.

*Q3.3 What is the optimal follow*-*up following an episode of uncomplicated acute diverticulitis?*

**Statement:** The use of 5-ASA agents does not prevent recurrent diverticulitis or improve chronic gastrointestinal symptoms after successful treatment for an episode of uncomplicated acute diverticulitis. The role of Rifaximin, probiotics, and fiber is less well defined.

**Recommendation:** Our expert group did not support the use of 5-ASA to prevent recurrent diverticulitis or improve chronic gastrointestinal symptoms among patients successfully treated for an episode of uncomplicated acute diverticulitis. (LoE: high. Strength of recommendation: strong).

Membership survey results: Yes 798 (96.49%).

606 members (73.28%) agreed that this recommendation was already their current practice; 105 (12.70%) agreed that this recommendation was likely to change their practice while 116 (14.03%) disagreed that it was likely to change their practice.

Consensus was reached with no further voting.

*Q3.4 What is the role of interval endoscopy following an episode of acute diverticulitis?*

**Statement:** In the absence of high-risk features, the detection rate for advanced adenomas or malignant lesions with colonic evaluation after an episode of uncomplicated acute diverticulitis is very low.

**Recommendation:** Our expert group recommends against routine colonic evaluation after successfully treated uncomplicated acute diverticulitis, unless high-risk features are present. (LoE: moderate. Strength of recommendation: weak).

Membership survey results: Yes 588 (73.96%).

389 members (48.93%) agreed that this recommendation was already their current practice; 147 (18.49%) agreed that this recommendation was likely to change their practice while 259 (32.58%) disagreed that it was likely to change their practice.

Since no consensus was obtained, further voting was carried at the SAGES and EAES meetings where 67% (142/209) and 70% (87/124) agreed to change practice if the recommendation was not already their practice.

Consensus was reached on the recommendation, but not on the likelihood to change practice.

### Topic 4: Non-resectional management of complicated acute diverticulitis

The full systematic review for this topic and reference list are provided in supplementary materials.

*Q4.1 What is the non*-*operative management of complicated AD?*

**Statement:** Antibiotic therapy alone is associated with a very high treatment success rate for abscesses (< 4 cm). An association between size and success rate has been observed.

**Recommendation:** For all abscesses, we recommend antibiotics should be considered first line treatment. (LoE: moderate. Strength of recommendation: strong).

Membership survey results: Yes 741 (94.52%).

624 members (79.59%) agreed that this recommendation was already their current practice; 65 (8.29%) agreed that this recommendation was likely to change their practice while 95 (12.12%) disagreed that it was likely to change their practice.

Consensus was reached with no further voting.

**Statement:** There is no evidence to support a particular antibiotic regime, route, or duration for complicated acute diverticulitis. Antibiotics are indicated in all complicated acute diverticulitis cases. If a drainage procedure is indicated, there is no evidence to support a prolonged course of antibiotics after source control is achieved.

**Recommendation:** Our expert panel recommended that antibiotic use covers gram-negative and anaerobic bacteria based on institutional protocols and antibiotic stewardship principles. (LoE: very low. Strength of recommendation: strong).

Membership survey results: Yes 767 (99.1%).

644 members (83.2%) agreed that this recommendation was already their current practice; 67 (8.66%) agreed that this recommendation was likely to change their practice while 63 (8.14%) disagreed that it was likely to change their practice.

Consensus was reached with no further voting.

**Statement:** Percutaneously drained abscesses > 4 cm successfully resolved in 80% of patients with a low complication and re-intervention rate.

**Recommendation:** Our expert panel recommends that percutaneous drainage be considered for larger abscesses, those that do not resolve on antibiotics, and/or in the presence of patient deterioration. (LoE: low, Strength of recommendation: weak).

Membership survey results: Yes 655 (86.3%).

540 members (71.15%) agreed that this recommendation was already their current practice; 78 (10.28%) agreed that this recommendation was likely to change their practice while 141 (18.58%) disagreed that it was likely to change their practice.

Consensus was reached with no further voting.

**Statement:** The majority of stable patients with radiological evidence of extraluminal air and no extravasation of contrast can be successfully managed non-operatively. The presence of an associated abscess or distant air are predictors of failure of non-operative management.

**Recommendation:** In stable patients diagnosed with free air, we recommend initial non-operative management. (LoE: low. Strength of recommendation: weak).

Membership survey results: Yes 645 (86.93%).

518 members (69.81%) agreed that this recommendation was already their current practice; 77 (10.38%) agreed that this recommendation was likely to change their practice while 147 (19.81%) disagreed that it was likely to change their practice.

Consensus was reached with no further voting.

*Q4.2 What is the role of laparoscopic lavage in the management of diverticulitis?*

**Statement:** Laparoscopic lavage has been shown to decrease stoma formation rate without impacting 1-year mortality, although short-term morbidity may be increased. There was no consensus on an effective laparoscopic lavage technique.

**Recommendation:** Lavage may be considered in selected Hinchey III patients by surgeons with appropriate expertise and the ability to closely watch for and manage complications. The lower stoma rate should be weighed against the higher risk of complications and re-intervention. (LoE: high. Strength of recommendation: weak).

Membership survey results: Yes 583 (79.97%).

358 members (49.11%) agreed that this recommendation was already their current practice; 130 (17.83%) agreed that this recommendation was likely to change their practice while 241 (33.06%) disagreed that it was likely to change their practice.

Since no consensus was achieved on this recommendation, re-voting was carried out in SAGES and EAES meetings with 76% (152/200) and 70.3% (83/118) agreement to respectively for likelihood to change practice.

Consensus was reached with the second round of voting.

*Q4.3 When is surgical treatment indicated in acute complicated diverticulitis?*

**Statement:** The majority of Hinchey Ib-II abscesses and presence of peri-colonic air can successfully be managed non-operatively.

**Recommendation:** In Hinchey Ib-II abscesses or presence of peri-colonic air cases, acute surgery should be reserved to patients who have exhausted non-operative options without improvement of symptoms or remain systemically unwell. (LoE: moderate. Strength of recommendation: strong).

Membership survey results: Yes 697 (96.4%).

579 members (80.08%) agreed that this recommendation was already their current practice; 80 (11.07%) agreed that this recommendation was likely to change their practice while 64 (8.85%) disagreed that it was likely to change their practice.

Consensus was reached with no further voting.

**Statement:** Non-operative management of Hinchey III or IV disease has a low success rate.

**Recommendation:** When there is clinical and/or radiological suspicion of Hinchey III or IV diverticulitis, acute surgery should be considered. (LoE: low. Strength of recommendation: strong).

Membership survey results: Yes 709 (99.16%).

623 members (87.13%) agreed that this recommendation was already their current practice; 45 (6.29%) agreed that this recommendation was likely to change their practice while 47 (6.57%) disagreed that it was likely to change their practice.

Consensus was reached with no further voting.

**Statement:** The majority of patients with Hinchey Ib-II abscesses that are successfully managed non-operatively for a single episode of diverticulitis are unlikely to experience any further acute diverticulitis episode during long-term follow-up.

**Recommendation:** We recommend that following a single episode of successfully treated Hinchey I/II acute diverticulitis, surgery should not be routinely offered solely to avoid future episodes. (LoE: low. Strength of recommendation: weak).

Membership survey results: Yes 653 (92.23%).

529 members (74.75%) agreed that this recommendation was already their current practice; 79 (11.16%) agreed that this recommendation was likely to change their practice while 100 (14.12%) disagreed that it was likely to change their practice.

Consensus was reached with no further voting.

*Q4.4 How should complicated diverticulitis be managed in specific patient groups?*

**Statement:** Immunosuppressed patients are a high-risk group for early, frequent and severe relapses after complicated acute diverticulitis managed non-operatively.

**Recommendation:** In immunosuppressed patients with complicated diverticulitis, we recommend early elective resectional surgery. (LoE: very low. Strength of recommendation: weak).

Membership survey results: Yes 615 (87.86%).

446 members (63.71%) agreed that this recommendation was already their current practice; 121 (17.29%) agreed that this recommendation was likely to change their practice while 133 (19%) disagreed that it was likely to change their practice.

Consensus was reached with no further voting.

**Statement:** Diabetic patients presenting with acute diverticulitis have a higher incidence of complicated episodes but similar success with non-operative management compared to non-diabetic patients.

**Recommendation:** We recommend clinicians consider diabetes as a risk factor for complicated acute diverticulitis, but non-operative management remains appropriate. LoE: low. Strength of recommendation: weak).

Membership survey results: Yes 672 (96.69%).

537 members (77.27%) agreed that this recommendation was already their current practice; 76 (10.94%) agreed that this recommendation was likely to change their practice while 82 (11.8%) disagreed that it was likely to change their practice.

Consensus was reached with no further voting.

### Topic 5: Operative management of emergency surgery

The full systematic review for this topic and reference list are provided in supplementary materials.

*Q5.1: What are the indications and timing of emergency surgery in acute complicated diverticulitis?*

**Statement:** Patients with perforated diverticulitis and peritonitis should be evaluated early for operative intervention to control infection. There is little data to inform the timing of operative intervention, but the clinical status of the patient should guide urgency of surgical intervention.

**Recommendation:** Patients with perforated diverticulitis with diffuse peritonitis (Hinchey III and IV) should undergo emergent surgical intervention. (LoE: low. Strength of recommendation: strong).

Membership survey results: Yes 681 (98.84%).

609 members (88.39%) agreed that this recommendation was already their current practice; 38 (5.52%) agreed that this recommendation was likely to change their practice while 42 (6.1%) disagreed that it was likely to change their practice.

Consensus was reached with no further voting.

*Q5.2: What is the role of laparoscopic resection in emergency surgery for diverticulitis?*

**Statement:** Laparoscopic sigmoid resection with or without stoma in the emergency setting has been shown to decrease overall complications compared to open resections.

**Recommendations:** When resection is indicated, we recommend consideration of laparoscopic approach for perforated diverticulitis in the appropriate clinical setting. (LoE: low. Strength of recommendation: weak).

Membership survey results: Yes 615 (89.65%).

441 members (64.29%) agreed that this recommendation was already their current practice; 96 (13.56%) agreed that this recommendation was likely to change their practice while 152 (22.16%) disagreed that it was likely to change their practice.

Consensus was reached with no further voting.

*Q5.3: What is the optimal surgical strategy in the acute setting?*

**Statement:** In Hinchey III, diverticulitis sigmoid resection with primary anastomosis with proximal diversion has similar mortality, lower morbidity and lower stoma rate at 12 months compared to Hartmann procedure with reversal.

**Recommendation:** In the appropriate clinical setting, we recommend consideration of sigmoid resection with primary anastomosis and proximal diversion over HP in patients with Hinchey III/IV diverticulitis.

(LoE: moderate. Strength of recommendation: weak).

Membership survey results: Yes 607 (89%).

352 members (51.61%) agreed that this recommendation was already their current practice; 214 (31.38%) agreed that this recommendation was likely to change their practice while 116 (17.01%) disagreed that it was likely to change their practice.

Consensus was reached with no further voting.

**Recommendation:** Hartmann’s procedure is the preferred operation for hemodynamically unstable patients with perforated diverticulitis. (LoE: low. Strength of recommendation: strong).

Membership survey results: Yes 667 (98.09%).

583 members (85.74%) agreed that this recommendation was already their current practice; 45 (6.62%) agreed that this recommendation was likely to change their practice while 52 (7.65%) disagreed that it was likely to change their practice.

Consensus was reached with no further voting.

**Statement:** In unstable perforated diverticulitis damage control strategies (resection without anastomosis, temporary abdominal closure and second look) showed acceptable mortality and morbidity and lower stoma rates.

**Recommendation:** We recommend in unstable patients with perforated diverticulitis damage control strategies (resection without anastomosis, temporary abdominal closure and second look) be considered. (LoE: low. Strength of recommendation: strong).

Membership survey results: Yes 635 (93.66%).

462 members (68.14%) agreed that this recommendation was already their current practice; 124 (18.29%) agreed that this recommendation was likely to change their practice while 92 (13.57%) disagreed that it was likely to change their practice.

Consensus was reached with no further voting.

*Q5.4: What is the recommended extent of sigmoid resection and what is the best practice for splenic flexure mobilization?*

**Statement:** No evidence was is available to support a statement.

**Recommendation:** In the setting of an emergency HP, we recommend limiting the resection to the acutely affected segment and not mobilizing the splenic flexure unless necessary. (LoE: none. Strength of recommendation: strong).

Membership survey results: Yes 653 (96.74%).

565 members (83.7%) agreed that this recommendation already their current practice; 52 (7.7%) agreed that this recommendation was likely to change their practice while 58 (8.59%) disagreed that it was likely to change their practice.

Consensus was reached with no further voting.

*Q5.5: What is the incidence of postoperative complications?*

**Statement:** Emergency surgery for perforated diverticulitis is associated with increased morbidity and mortality compared to elective surgery. (LoE: low. No recommendation).

*Q5.6: How should complicated emergency diverticulitis be managed among specific patient groups?*

**Statement:** Immunosuppressed patients have increased mortality and morbidity following emergency surgery compared to immunocompetent individuals.

**Statement:** Elderly patients with perforated diverticulitis have a higher mortality rate following emergency surgery. (LoE: low. No recommendation).

### Topic 6: Operative management of elective surgery

The full systematic review for this topic and reference list are provided in supplementary materials.

*Q6.1: What is the role of laparoscopy in elective surgery for diverticulitis?*

**Statement:** Laparoscopy is safe in the setting of elective surgery for diverticulitis and is associated with reduced rates of morbidity and length of stay compared to open surgery.

**Recommendation:** A laparoscopic approach is recommended in elective surgery for diverticular disease, when feasible. (LoE: high. Strength of recommendation: strong).

Membership survey results: Yes 659 (98.21%).

561 members (83.61%) agreed that this recommendation was already their current practice; 65 (9.69%) agreed that this recommendation was likely to change their practice while 45 (6.71%) disagreed that it was likely to change their practice.

Consensus was reached with no further voting.

*Q6.2: When is elective interval sigmoid resection indicated following an episode(s) of complicated acute diverticulitis?*

**Statement:** Limited evidence suggests no difference in morbidity or mortality when comparing early (< 6 weeks) versus late (> 6 weeks) elective resection for diverticular disease; rate of conversion from laparoscopic to open may be higher in early surgery.

**Recommendation:** Consideration should be given to delaying elective interval sigmoid resection for minimum 6 weeks from the most recent episode of acute diverticulitis. (LoE: low. Strength of recommendation: weak).

Membership survey results: Yes 592 (88.62%).

449 members (67.22%) agreed that this recommendation was already their current practice; 104 (15.57%) agreed that this recommendation was likely to change their practice while 115 (17.22%) disagreed that it was likely to change their practice.

Consensus was reached with no further voting.

*Q6.3: When should prophylactic ureteral stents be used prior to elective surgery for diverticulitis?*

**Statement:** No evidence was available regarding the efficacy of prophylactic ureteric stenting in elective surgery for diverticulitis.

**Recommendation:** We recommend the utilization of a selective strategy based on imaging and patient characteristics for placement of prophylactic ureteral stents prior to elective surgery for diverticulitis.

(LoE: none. Strength of recommendation: weak).

Membership survey results: Yes 587 (88.14%).

449 members (67.42%) agreed that this recommendation was already their current practice; 73 (10.96%) agreed that this recommendation was likely to change their practice while 144 (21.62%) disagreed that it was likely to change their practice.

Consensus was reached with no further voting.

*Q6.4: What is the role of bowel preparation prior to surgery in the management of diverticulitis?*

**Statement:** Although we found no evidence specific to diverticular disease, in a general colorectal population, the use of mechanical bowel preparation is associated with decreased rates of SSI and anastomotic leak when combined with oral antibiotics.

**Recommendation:** While the evidence specific to diverticular disease is limited, evidence exists in the setting of elective colorectal surgery to recommend the use of an isosmotic mechanical bowel preparation with oral antibiotics prior to surgery. (LoE: moderate. Strength of recommendation: strong).

Membership survey results: Yes 584 (87.95%).

439 members (66.11%) agreed that this recommendation was already their current practice; 130 (19.58%) agreed that this recommendation was likely to change their practice while 95 (14.31%) disagreed that it was likely to change their practice.

Consensus was reached with no further voting.

*Q6.5: What is the optimal surgical strategy in the elective setting for complicated and uncomplicated diverticulitis?*

**Statement:** There is limited data to guide surgical strategy in the elective setting for complicated and uncomplicated diverticulitis. Data are mixed regarding the benefits of IMA preservation versus high ligation in preventing anastomotic leak.

**Recommendation:** Preservation of the inferior mesenteric artery should be considered to preserve vascular supply of the anastomosis (so long as this does not compromise formation of a tension-free anastomosis). (LoE: low. Strength of recommendation: weak).

Membership survey results: Yes 599 (90.48%).

462 members (69.79%) agreed that this recommendation was already their current practice; 80 (12.08%) agreed that this recommendation was likely to change their practice while 120 (18.13%) disagreed that it was likely to change their practice.

Consensus was reached with no further voting.

*Q6.6: When is a Hartmann’s procedure indicated in the elective setting?*

**Statement:** No evidence was available regarding the role of HP in elective surgery for diverticulitis.

**Recommendation:** Every effort should be made to construct a primary anastomosis in the elective setting for complicated and uncomplicated diverticulitis. (LoE: none. Strength of recommendation: weak).

Membership survey results: Yes 629 (95.16%).

545 members (82.45%) agreed that this recommendation was already their current practice; 49 (7.41%) agreed that this recommendation was likely to change their practice while 67 (10.14%) disagreed that it was likely to change their practice.

Consensus was reached with no further voting.

*Q6.7: What is the recommended extent of sigmoid resection, including mobilization of proximal bowel and mesentery/phlegmon dissection?*

**Statement:** No differences in leak, morbidity or mortality rates are reported when comparing elective diverticular resections with versus without splenic flexure mobilization.

**Recommendation:** Although routine mobilization of the splenic flexure is not supported by evidence, we recommend that the descending colon should be fully mobilized to provide sufficient colonic length to form a tension-free anastomosis. (LoE: low. Strength of recommendation: weak).

Membership survey results: Yes 602 (91.21%).

498 members (75.45%) agreed that this recommendation was already their current practice; 60 (9.09%) agreed that this recommendation was likely to change their practice while 102 (15.45%) disagreed that it was likely to change practice.

Consensus was reached with no further voting.

*Q6.8: What is the optimal level of resection proximally and distally, and how should the rectum be transected (as it relates to rectal preservation and defecatory function)?*

**Statement:** No evidence was available regarding the level of proximal and distal resection during elective surgery for diverticulitis.

**Recommendation:** We recommend transecting the colon proximal to the phlegmon in an area without gross evidence of inflammation. No attempt should be made to resect every diverticulum proximal to the phlegmon. (LoE: none. Strength of recommendation: weak).

Membership survey results: Yes 634 (96.21%).

540 members (81.94%) agreed that this recommendation was already their current practice; 55 (8.35%) agreed that this recommendation was likely to change their practice while 64 (9.71%) disagreed that it was likely to change their practice.

Consensus was reached with no further voting.

**Statement:** Colorectal anastomoses are associated with a decreased risk of recurrent diverticular disease relative to colo-sigmoid anastomoses in both laparoscopic and open elective resections.

**Recommendation:** Distal transection at or below the rectosigmoid junction (at the level of the sacral promontory where the tenia coli coalesce) is recommended to decrease the risk of recurrent diverticulitis. (LoE: low. Strength of recommendation: strong).

Membership survey results: Yes 646 (98.03%).

546 members (82.85%) agreed that this already their current practice; 63 (9.56%) agreed that this recommendation was likely to change their practice while 50 (7.59%) disagreed that it was likely to change their practice.

Consensus was reached with no further voting.

*Q6.9: What is the optimal strategy of colorectal anastomosis, and how could this be assessed?*

**Statement:** Postoperative morbidity is not impacted by choice of hand-sewn or stapled anastomosis for elective resection of diverticular disease.

**Recommendation:** We recommend the use of either a hand-sewn or stapled anastomosis based on individual surgeon preference. (LoE: low. Strength of recommendation: weak).

Membership survey results: Yes 620 (94.08%).

505 members (76.63%) agreed that this recommendation was already their current practice; 41 (6.22%) agreed that this recommendation was likely to change their practice while 113 (17.15%) disagreed that it was likely to change their practice.

Consensus was reached with no further voting.

**Recommendation:** While there was no evidence specific to diverticular disease, based on the evidence from the general colorectal population we recommend use of an air leak test to evaluate the integrity of the colorectal anastomosis and prevent anastomotic leak. (LoE: low. Strength of recommendation: strong).

Membership survey results: Yes 622 (94.67%).

537 members (81.74%) agreed that this recommendation was already their current practice; 49 (7.46%) agreed that this recommendation was likely to change their practice while 71 (10.81%) disagreed that it was likely to change their practice.

Consensus was reached with no further voting.

*Q6.10: What is the role of abdominal/pelvic drains following elective resection for complicated diverticulitis?*

**Statement:** No evidence was available to support a statement.

**Recommendation:** There is no evidence to support routine use of abdominal/pelvic drain in elective surgery for diverticulitis. We recommend the decision to place an abdominal or pelvic drain following elective resection for complicated diverticular disease be left to the surgeon’s discretion. (LoE: none. Strength of recommendation: weak).

Membership survey results: Yes 582 (88.72%).

472 members (71.95%) agreed that this recommendation was already their current practice; 50 (7.62%) agreed that this recommendation was likely to change their practice while 134 (20.43%) disagreed that it was likely to change their practice.

Consensus was reached with no further voting.

*Q6.11: What is the incidence of postoperative complications following elective surgery for diverticular disease?*

**Statement:** The incidence of postoperative complications following elective surgery for diverticular disease varies widely, ranging from 5 to 38%. Laparoscopic surgery conveys a lower risk of postoperative complications as compared to open resection. (LoE: high. no recommendation).

*Q6.12: What are the functional outcomes postoperatively (short*-*term), including defecatory and sexual function and quality of life following elective surgery for diverticular disease?*

**Statement:** Short-term functional outcomes and quality of life are improved in patients following elective resection for diverticular disease as compared to patients with conservatively managed disease.

**Recommendation:** We recommend elective resection in patients with symptomatic diverticular disease that is negatively impacting quality of life. (LoE: moderate. Strength of recommendation: strong).

Membership survey results: Yes 638 (97.7%).

532 members (81.47%) agreed that this recommendation was already their current practice; 71 (10.87%) agreed that this recommendation was likely to change their practice while 50 (7.66%) disagreed that it was likely to change their practice.

Consensus was reached with no further voting.

**Statement:** Short-term functional outcomes and quality of life are improved following laparoscopic elective resection of diverticular disease as compared to open resection.

**Recommendation:** We recommend a laparoscopic approach for elective resection of diverticular disease when feasible to improve short-term functional outcomes and quality of life. (LoE: moderate. Strength of recommendation: strong).

Membership survey results: Yes 639 (97.86%).

527 members (80.70%) agreed that this recommendation was already their current practice; 71 (10.87%) agreed that this recommendation was likely to change their practice while 55 (8.42%) disagreed that it was likely to change their practice.

Consensus was reached with no further voting.

*Q6.13: How should complicated elective diverticulitis be managed among specific patient groups?*

**Statement:** Complicated elective diverticulitis in overweight/obese patients can be safely managed with laparoscopic surgery with similar morbidity and rate of conversion to normal weight patients.

**Recommendation:** A laparoscopic approach is recommended for the elective resection of diverticular disease in obese patients, when feasible. (LoE: low. Strength of recommendation: weak).

Membership survey results: Yes 608 (93.25%).

497 members (76.23%) agreed that this recommendation was already their current practice; 63 (9.66%) agreed that this recommendation was likely to change their practice while 92 (14.11%) disagreed that it was likely to change their practice.

Consensus was reached with no further voting.

**Statement:** Elective surgery for complicated diverticular disease in immunosuppressed patients may be associated with an increased risk of complications. Surgical planning and patients counselling should be adapted to reflect this.

**Recommendation:** When considering elective resection of complicated diverticular disease in the immunocompromised patient, we recommend a lower threshold for stoma formation. (LoE: low. Strength of recommendation: weak).

Membership survey results: Yes 615 (95.05%).

468 members (72.33%) agreed that this recommendation was already their current practice; 84 (12.98%) agreed that this recommendation was likely to change their practice while 95 (14.68%) disagreed that it was likely to change their practice.

Consensus was achieved with no further voting.

## Discussion

The objective of this international DCC was to generate an updated review and evidence-based recommendations that could provide valuable guidance to our members, particularly acute care surgeons, on this common surgical problem for which the management has radically changed in the last 10 years. In this first collaborative project, we successfully engaged the wider membership of EAES and SAGES in order to ensure that the final output would reflect participants’ opinions, be relevant across both Societies’ diverse membership, and help bridge existing gaps in clinical practice upon evidence-based principles. Although a number of AD guidelines have been reported by national and specialty societies, significant discrepancies have resulted in differing levels of uptake and subsequent widespread variations in practice [1]. Involving the members of both societies, the DCC achieved broader consensus beyond that of an expert panel, ensuring relevance and applicability to clinical practice, with the hope of increasing adoption and unifying clinical practice for the benefit of our patients.

Through this effort, six systematic reviews of the contemporary evidence related to the epidemiology, diagnosis and classification of acute diverticulitis, elective and emergency treatment of uncomplicated and complicated acute diverticulitis were generated. These comprehensive reviews (presented in the supplementary materials) underpinned 51 consensus statements and 41 recommendations generated by the international steering group. The level of membership participation from both societies exceeded our expectations with over 1000 online survey responses and 300 audience members from both congresses actively engaged to reshape the final statements and recommendations.

Consensus was achieved on most of the recommendations which were also reported as likely to impact or change the clinical practice for participants not already applying them. This is perhaps not surprising as high-level evidence is now available for many acute diverticulitis topics and underpinned the basis of each statement and linked recommendation developed by this conference.

A number of areas of continuing controversy in the current diagnosis and management of AD were identified based on the lack of consensus amongst members’ survey responses. After the congress presentations, a few areas of disagreement persisted involving the selective use of imaging to guide AD diagnosis, recommendations against antibiotics in non-complicated AD and routine colonic evaluation after resolution of non-complicated diverticulitis.

The first area of disagreement was on selective imaging to guide the diagnosis of acute diverticulitis. Although a consensus was achieved on the moderate strength evidence statement and recommendation, consensus was not achieved on the likelihood to change practice. The weak recommendation to defer or eliminate routine imaging in patients with suspected AD based on physical examination, symptoms and C-reactive protein (CRP) levels also did not gain traction. This likely reflects the reluctance to dramatically change institutional diagnostic protocols for patients presenting with abdominal pain, from concerns regarding alternative diagnoses and potential diagnosis and treatment delays.

Although the use of CRP in the diagnosis and severity assessment of AD reached consensus in voting, willingness to adopt into clinical practice did not. After presenting the evidence in support for this strong recommendation, consensus was ultimately achieved upon live re-voting by both SAGES and EAES DCC participants. While 63% of surgeons surveyed reported not using CRP routinely in their practice, the majority were willing to implement this recommendation, which may be facilitated by the availability and low-cost of testing, and that it did not preclude replacing other diagnostic tests. On the other hand, the recommendation to use imaging selectively in the suspected diagnosis of AD, which was supported in voting, did not reach consensus with respect to change in practice, and even after two consecutive rounds of voting.

One notable topic of disagreement that also highlighted differences in opinion and clinical practice across both sides of the Atlantic is the role of antibiotics in non-complicated AD. Consensus was achieved by live re-voting among EAES members but not by SAGES members, even after presenting data from several multinational randomized controlled trials concluding that omission of antibiotics was safe and did not alter patient recovery nor complication rates. Reasons for this discrepancy that may be specific to North American surgeons including concerns over medico-legal repercussions of not “treating” patients with a diagnosis of AD, and reluctance to go against patient expectations with negative impact on future referrals. Another reason may be strong practice preferences that are difficult to change. Many physicians may consider that the benefits of antibiotics to largely outweigh the risks although in this age of increasing antibiotic resistance, public health considerations are of increasing importance. Similarly, although the recommendation against routine colonic evaluation after resolution of an uncomplicated episode of AD was supported in voting, consensus to adopt this recommendation in clinical practice was supported by EAES but not SAGES participants. While this recommendation follows typical practice in European nations, physicians may be reluctant to change practice in North America based on physician and patient expectations with respect to screening.

Perhaps most interesting was the fact that despite the current surgical debate on the use of laparoscopic lavage in Hinchey III AD, agreement with the weak recommendation to consider lavage in selected cases, reached consensus. While this recommendation was initially reported as unlikely to impact or change practice at initial voting, consensus was ultimately achieved upon live re-voting at both SAGES and EAES meetings after high level of evidence was presented on this topic that this recommendation was likely to change practice.

Overall, this project highlighted interesting trends and controversies related to surgeons’ willingness to alter standard practice based on evidence regarding modern AD management. One trend was clinicians rejecting new and high-level evidence, especially where it called for a radical change in established practice. Another more common pattern was when physicians agreed with the evidence in support of the given recommendation but were not willing to change their current practice. Given our sample size, it is likely that there are a variety of reasons and multi-factorial considerations responsible for the areas of discrepancy. As justified by the size of this consensus project and the high level of engagement observed, the survey and voting used a quantitative design. As a result, we are unable to definitively report the reasons behind the support or rejection of each statement and recommendation. However, our outputs help define the few areas of ongoing controversy which can now be investigated with further focused studies. Of particular interest are those where the membership disagreed with high level evidence and/or strong recommendations. It will be possible to revisit these questions and re-canvas the members views and assess whether practice actually changed.

This consensus conference and its outputs should be considered in the context of our limitations. The sample size of the live voting cohorts was significantly smaller than that of the original voting pool in the survey and may not be representative of each respective Society’s diverse opinions and practices. In addition, live voting may have been influenced by presentation of online membership polling results. Additionally, conference time constraints may have curtailed full discussion of complex topics and presentation of all relevant studies which risks further impacting the outcomes of subsequent voting. Limited, and occasionally no evidence was identified for a number of clinically relevant questions frequently encountered by our members. It is hoped that identification of these areas provides an agenda to stimulate future collaborative research between SAGES and EAES.

## Conclusion

This joint EAES and SAGES collaborative consensus conference updates surgeons on current and best evidence and provides a set of recommendations that can guide clinical practice on the management of acute diverticulitis. Strong membership engagement helped to highlight the clinical relevance of consensus recommendations and shed light on ongoing controversies related to diagnosis and management of acute diverticulitis.

## Electronic supplementary material

Below is the link to the electronic supplementary material.

Supplementary Appendix 1: Search strings and associated results for all six literature searches. Searches were performed between October 26^th^ 2017 and November 8th 2017. Supplementary material 1 (DOCX 240 kb)

Supplementary Fig. 1: PRISMA flow charts for all six acute diverticulitis topics.. Supplementary material 2 (TIFF 1480 kb)

Supplementary materials 1–6: Full text literature review results reporting the AD evidence base and rationale behind all statements and recommendations formulated in the consensus conference. Supplementary material 3 (DOCX 84 kb)

Supplementary material 4 (DOCX 229 kb)

Supplementary material 5 (DOC 71 kb)

Supplementary material 6 (DOCX 164 kb)

Supplementary material 7 (DOCX 57 kb)

Supplementary material 8 (DOCX 86 kb)
