# Supplementary material for: EAES and SAGES 2018 consensus conference on acute diverticulitis management: evidence-based recommendations for clinical practice
Source: Surg Endosc. 2019 Jun 27;33(9):2726–41. doi: 10.1007/s00464-019-06882-z (PMC6684540; doi:10.1007/s00464-019-06882-z)
Supplement: Supplementary file 1 — Supplementary Appendix 1: Search strings and associated results for all six literature searches. Searches were performed between October 26th 2017 and November 8th 2017. Supplementary material 1 (DOCX 240 kb) [file 464_2019_6882_MOESM1_ESM.docx]

Search terms and EAES SAGES Acute Diverticulitis Guideline

Contents

[PubMed 26 Oct 2017 4](#_Toc1492541)

[Topic 1: Epidemiology and natural history (1+2-9) 4](#_Toc1492542)

[Topic 2: Diagnosis and classifications (1+11-12) 5](#_Toc1492543)

[Topic 3: Uncomplicated diverticulitis 7](#_Toc1492544)

[Topic 4: Complicated diverticulitis 7](#_Toc1492545)

[PubMed 31 Oct 2017 (440+808) 7](#_Toc1492546)

[Bostian search terms 1 Nov 2017 8](#_Toc1492547)

[PubMed 8 Nov 2017 9](#_Toc1492548)

[Topic 1: Epidemiology and natural history 9](#_Toc1492549)

[Topic 2: Diagnosis and classifications 11](#_Toc1492550)

[Topic 3: Nonsurgical intervention of uncomplicated diverticulitis 13](#_Toc1492551)

[Topic 4: Nonsurgical intervention of complicated diverticulitis 14](#_Toc1492552)

[Topic 5: Emergency surgery 14](#_Toc1492553)

[Topic 6: Elective surgery 15](#_Toc1492554)

[Limits 15](#_Toc1492555)

| **Topic** | **Questions** | **Key search** |
| --- | --- | --- |
| 1. **Epidemiology and natural history** | - 1. What is the incidence and prevalence of left sided acute diverticulitis?   2. What factors are associated with an increased risk of developing acute diverticulitis   3. Are there any other risk factors in specific patient groups?   4. What is microbiome profile in acute diverticulitis? | **Race, ethnicity**  **Gender, age**  **Diet**  **Constipation**  **BMI** |
| 1. **Diagnosis and classi­fications** | 2.1 What are the classifications of acute diverticulitis?  2.2 How is the diagnosis of acute diverticulitis best established?  2.3 How is the severity of perforated diverticulitis best assessed? | **Acute vs. chronic vs. recurrent, uncomplicated vs. complicated**  **complicated: phlegmon, perforation, stricture, colovisceral fistula, obstruction**  **Imaging (CT, MRI, US, BE)**  **Physical examination**  **Biochemical profile, Endoscopy**  **Adjunct tests : Barium enema, cystoscopy**  **CT with Hinchey classification for perforated diverticulitis** |
| 1. **Non-resection manage­­ment of uncompli­cated** | - 1. What are the risk factors for developing recurrent or ongoing diverticulitis among patients with uncomplicated AD?   2. What is the optimum non-operative measures of uncomplicated acute Diverticulitis   3. What is the optimum follow up following uncomplicated AD?   4. What is the optimal timing for interval endoscopy following acute episode of diverticulitis?   5. What are the indications for surgical intervention in uncomplicated DD?   6. When is interval sigmoid resection indicated following episode(s) of uncomplicated acute diverticulitis?   7. What is the optimum management of uncomplicated AD among specific patients gp? | **Hospitalization and discharge criteria; Bowel rest; IV ABX**  **PO ABX; stool softener; diet**  **Probiotics; other pharmacological treatment; any other specific diet**  **Frequency of episodes**  **Symptoms/QOL**  **specific comorbidities**  **Young <40**  **Transplant, immunosuppressive medications**  **frail and elder** |
| 1. **Non-resection management of complicated** | - 1. What is the non-operative management of complicated AD; abscess; localized perforation; bleeding and colovesical fistula   2. What is the role of laparoscopic lavage in the management of diverticulitis?" What are the recommended criteria for discharge following hospitalization for complicated diverticulitis?   3. When is surgical treatment indicated in acute complicated diverticulitis?   4. How should complicated diverticulitis be managed in specific patients group? | **PO ABX**  **Percutaneous drainage (perforation)**  **colon stenting (obstruction)**  **Endoscopy (bleeding)**  **Vascular embolization (bleeding)**  **Routine**  **Symptoms/QOL**  **specific comorbidities**  **Patient choice?**  **Young <40**  **Transplant, immunosuppressive medications**  **frail and elder** |
| 1. **Perioperative management of emergency surgery** | - 1. what are the indication and timing of surgery in acute complicated diverticulitis   2. "What is the role of laparoscopic resection in emergency surgery for diverticulitis?"   3. What is the optimal surgical strategy in the acute setting?   4. When is primary anastomosis with or without proximal diversion indicated?   5. When is Hartman's procedure indicated?   6. What is the recommended extent of sigmoid resection?   7. What are the best practices to mobilizing the mesentery/phlegmon during resection of diverticulitis?   8. What is the best practice re splenic flexure mobilization?   9. what is the optimal level of resection proximally and distally and how should the rectum be transected (as it relates to rectal preservation and defecatory function)   5.10 How should the rectal stump be managed during Hartmann's procedure?  5.11 What are the optimal abdominal incision and extraction sites?  5.12 What is the optimal strategy of colorectal anastomosis and how this could be assessed?  5. 13 What are the indications for conversion to open surgery?  5.14 What is the role and management of abdominal/pelvic drains following resection for complicated diverticulitis?  5.15 What type and duration of ABX should be used in the postoperative period?  5.16 Does Enhanced recovery have a role in postop management of acute diverticulitis?  5.17 What are the functional outcomes postoperatively (short-long-term) re defecatory, sexual, QOL function?  5.18 What is the incidence of postop complications?  5.19 How should complicated emergency diverticulitis be managed among specific patients gp? | **Hinchey 3 or 4** |
| 1. **Perioperative management for elective surgery** | - 1. What is the optimal surgical strategy in the elective setting?   2. "What is the role of laparoscopy in diverticulitis?" Broad question. Will need subgroups   3. When is interval sigmoid resection indicated following episode(s) of complicated acute or chronic diverticulitis?   4. When should prophylactic ureteral stents be used for surgery for acute diverticulitis?   5. What is the role of bowel preparation in the management of Diverticulitis?   6. What is the optimal surgical strategy in the elective setting?   7. When is Hartman's procedure indicated in the elective setting?   8. What is the recommended extent of sigmoid resection?   9. What are the best practices to mobilizing the mesentery/phlegmon during resection of diverticulitis?   10. What is the best practice re splenic flexure mobilization?   11. what is the optimal level of resection proximally and distally and how should the rectum be transected (as it relates to rectal preservation and defecatory function)   12. What are the optimal abdominal incision and extraction sites after elective surgery?   13. What is the optimal strategy of colorectal anastomosis and how this could be assessed?   14. What are the indications for conversion to open surgery in the elective setting?   15. What is the role and management of abdominal/pelvic drains following elective resection for complicated diverticulitis?   16. What is the incidence of postop complications?   17. How should complicated elective diverticulitis be managed in vulnerable population?   5.22 What are the functional outcomes postoperatively (short-long-term) re defecatory, sexual, QOL function?   - 1. What is the optimum follow up after elective resection for DD?   2. How should complicated emergency diverticulitis be managed among specific patients gpin vulnerable population? | **Hinchey 1 of 2** |

# PubMed 26 Oct 2017

**Topics + searches per question**

# Topic 1: Epidemiology and natural history (1+2-9)

1. **Diverticulitis**

"Diverticulosis, Colonic"[Mesh] OR "Diverticulum, Colon"[Mesh] OR ((“Colon"[Mesh:NoExp] OR "Colon, Descending"[Mesh] OR "Colon, Sigmoid"[Mesh] OR "Colon, Transverse"[Mesh] OR sigmoid*[tiab] OR colon*[tiab] OR transvers*[tiab] OR descend*[tiab] OR left*[tiab]) AND diverticul*[tiab]) NOT right[ti]

"Diverticulosis, Colonic"[Mesh] OR "Diverticulum, Colon"[Mesh] OR ((“Colon"[Mesh:NoExp] OR "Colon, Descending"[Mesh] OR "Colon, Sigmoid"[Mesh] OR "Colon, Transverse"[Mesh] OR sigmoid*[tiab] OR colon*[tiab] OR transvers*[tiab] OR descend*[tiab] OR left*[tiab]) AND ("Diverticulitis"[Mesh] OR diverticul*[tiab])) NOT right[ti]

1. **Epidemiology broad**

"Morbidity"[Mesh] OR "Mortality"[Mesh] OR "Epidemiologic Studies"[Mesh] OR cohort[tiab] OR (case[tiab] AND (control[tiab] OR controll*[tiab] OR comparison[tiab] OR referent[tiab])) OR risk[tiab] OR causation[tiab] OR causal[tiab] OR "odds ratio"[tiab] OR etiol*[tiab] OR aetiol*[tiab] OR "natural history"[tiab] OR predict*[tiab] OR prognos*[tiab] OR outcome[tiab] OR course[tiab] OR retrospect*[tiab] OR "comparative study"[pt] OR "risk factors"[mesh] OR "cohort"[tw] OR "compared"[tw] OR "groups"[tw] OR "multivariate"[tw] OR inciden*[tiab] OR prevalen*[tiab] OR morbid*[tiab] OR mortal*[tiab] OR epidemiol*[tiab]

- 1. **Epidemiology without morbidity/mortality**

"Epidemiologic Studies"[Mesh] OR cohort[tiab] OR (case[tiab] AND (control[tiab] OR controll*[tiab] OR comparison[tiab] OR referent[tiab])) OR risk[tiab] OR causation[tiab] OR causal[tiab] OR "odds ratio"[tiab] OR etiol*[tiab] OR aetiol*[tiab] OR "natural history"[tiab] OR predict*[tiab] OR prognos*[tiab] OR outcome[tiab] OR course[tiab] OR retrospect*[tiab] OR "comparative study"[pt] OR "risk factors"[mesh] OR "cohort"[tw] OR "compared"[tw] OR "groups"[tw] OR "multivariate"[tw] OR inciden*[tiab] OR prevalen*[tiab] OR epidemiol*[tiab]

- 1. **Epidemiology specific: Majr and [ti]**

"Morbidity"[Majr] OR "Mortality"[Majr] OR "Epidemiologic Studies"[Majr] OR cohort[ti] OR (case[ti] AND (control[ti] OR controll*[ti] OR comparison[ti] OR referent[ti])) OR risk[ti] OR causation[ti] OR causal[ti] OR "odds ratio"[ti] OR etiol*[ti] OR aetiol*[ti] OR "natural history"[ti] OR predict*[ti] OR prognos*[ti] OR outcome[ti] OR course[ti] OR retrospect*[ti] OR "comparative study"[pt] OR "risk factors"[Majr] OR "cohort"[ti] OR "compared"[ti] OR "groups"[ti] OR "multivariate"[ti] OR inciden*[ti] OR prevalen*[ti] OR morbid*[ti] OR mortal*[ti] OR epidemiol*[ti] OR cohort[ot] OR (case[ot] AND (control[ot] OR controll*[ot] OR comparison[ot] OR referent[ot])) OR risk[ot] OR causation[ot] OR causal[ot] OR "odds ratio"[ot] OR etiol*[ot] OR aetiol*[ot] OR "natural history"[ot] OR predict*[ot] OR prognos*[ot] OR outcome[ot] OR course[ot] OR retrospect*[ot] OR "comparative study"[pt] OR "risk factors"[Majr] OR "cohort"[ot] OR "compared"[ot] OR "groups"[ot] OR "multivariate"[ot] OR inciden*[ot] OR prevalen*[ot] OR morbid*[ot] OR mortal*[ot] OR epidemiol*[ot]

1. **Ethnicity**

"Ethnic Groups"[Mesh] OR "Continental Population Groups"[Mesh] OR racial*[tiab] OR race[tiab] OR races[tiab] OR ethnic*[tiab] OR population group*[tiab] OR nationalit*[tiab]

"Ethnic Groups"[Mesh] OR "Continental Population Groups"[Mesh] OR racial*[tiab] OR race[tiab] OR races[tiab] OR ethnic*[tiab] OR population group*[tiab] OR nationalit*[tiab] **OR ethnology[sh]**

1. **Gender**

"Sex"[Mesh] OR "Men"[Mesh:NoExp] OR "Male"[Mesh] OR man[tiab] OR men[tiab] OR male[tiab] OR males[tiab] OR sex[tiab] OR "Female"[Mesh] OR "Women"[Mesh] OR female*[tiab] OR woman[tiab] OR women[tiab] OR feminin*[tiab]

1. **Age**

"Aging"[Mesh] OR "Adult"[Mesh] OR aging*[tiab] OR age[tiab] OR ages[tiab] OR adult*[tiab] OR "Aged"[Mesh] OR "Aged, 80 and over"[Mesh] OR "Frail Elderly"[Mesh] OR "Geriatrics"[Mesh] OR "Geriatric Psychiatry"[Mesh] OR "Geriatric Nursing"[Mesh] OR "Geriatric Dentistry"[Mesh] OR "Dental Care for Aged"[Mesh] OR "Health Services for the Aged"[Mesh] OR elder*[tw] OR eldest[tw] OR frail*[tw] OR geriatri*[tw] OR old age*[tw] OR oldest old*[tw] OR senior*[tw] OR senium[tw] OR very old*[tw] OR septuagenarian*[tw] OR octagenarian*[tw] OR octogenarian*[tw] OR nonagenarian*[tw] OR centarian*[tw] OR centenarian*[tw] OR supercentenarian*[tw] OR older people[tw] OR older subject*[tw] OR older patient*[tw] OR older age*[tw] OR older adult*[tw] OR older man[tw] OR older men[tw] OR older male*[tw] OR older woman[tw] OR older women[tw] OR older female*[tw] OR older population*[tw] OR older person*[tw]

1. **Diet**

"Diet"[Mesh] OR "Dietary Supplements"[Mesh] OR "Food and Beverages"[Mesh] OR diet[tiab] OR diets[tiab] OR supplements[tiab] OR supplement[tiab] OR nutraceutical[tiab] OR neutraceutical[tiab] OR nuts[tiab] OR seeds[tiab] OR popcorn[tiab] OR tomato*[tiab] OR meat[tiab] OR meats[tiab] OR pork[tiab] OR beef[tiab]

**6.1 Dietary fiber**

"Dietary Fiber"[Mesh] OR dietary fiber*[tiab] OR dietary fibre*[tiab] OR diet fiber*[tiab] OR wheat bran*[tiab] OR roughage*[tiab] OR high fiber diet*[tiab] OR high fibre diet*[tiab] OR alimentary fiber*[tiab] OR alimentary fibre*[tiab]

1. **Stool**

"Constipation"[Mesh] OR "Diarrhea"[Mesh] OR "Feces"[Mesh] OR bowel habit*[tiab] OR constipat*[tiab] OR diarrh*[tiab] OR feces[tiab] OR faeces[tiab] OR fecal*[tiab] OR faecal*[tiab] OR stool[tiab]

1. **BMI**

"Overweight"[Mesh] OR obese*[tiab] OR overweight*[tiab] OR obesit*[tiab] OR "Body Mass Index"[Mesh] OR "body mass index"[tiab] OR bmi[tiab] OR adipos*[tiab] OR stout[tiab] OR bulky[tiab] OR sizeable[tiab] OR heavy[tiab] OR fat[tiab] OR fatty[tiab] OR greasy[tiab]

1. **Microbiome**

"Gastrointestinal Microbiome"[Mesh] OR microbio*[tiab] OR flora[tiab] OR floras[tiab] OR microflora*[tiab]

1. **Diagnostic accuracy**

"Sensitivity and Specificity"[MeSH] OR specificit*[tw] OR screening[tw] OR accura*[tw] OR reference value*[tw] OR false positive[tw] OR false negative[tw] OR predictive value*[tw] OR roc[tw] OR likelyhood*[tw] OR likelihood*[tw]

# Topic 2: Diagnosis and classi­fications (1+11-12)

1. **Diagnosis**

"Diagnosis"[Mesh] OR "diagnosis" [Subheading] OR "Diagnostic Techniques and Procedures"[Mesh] OR diagnos*[tiab]

"Diagnosis"[Mesh] OR "diagnosis" [Subheading] OR "Diagnostic Techniques and Procedures"[Mesh] OR diagnos*[tiab] **OR severit*[tiab] OR assessment*[tiab]**

- 1. **Diagnostic imaging (CT, MRI, US, BE)**

"Tomography, X-Ray Computed"[Mesh] OR computed tomograph*[tiab] OR ct[tiab] OR cts[tiab] OR cat scan*[tiab] OR computer assisted tomograph*[tiab] OR computerized tomograph*[tiab] OR computed x ray tomograph*[tiab] OR computed xray tomograph*[tiab] OR "Magnetic Resonance Imaging"[Mesh] OR ("magnetic resonance"[tiab] AND (image[tiab] OR images[tiab] OR imaging[tiab])) OR mri[tiab] OR mris[tiab] OR nmr[tiab] OR mra[tiab] OR mras[tiab] OR zeugmatograph*[tiab] OR "mr tomography"[tiab] OR "mr tomographies"[tiab] OR "mr tomographic"[tiab] OR "proton spin"[tiab] OR ((magneti*[tiab] OR "chemical shift"[tiab]) AND imaging[tiab]) OR fmri[tiab] OR fmris[tiab] OR "Ultrasonography"[Mesh] OR "diagnostic imaging"[Subheading] OR ultraso*[tiab] OR sonograph*[tiab] OR echograph*[tiab] OR echocardiograph*[tiab] OR echotomograph*[tiab] OR "Barium Enema"[Mesh] OR barium enema*[tiab] OR "Endoscopy, Digestive System"[Mesh] OR "Colonoscopy"[Mesh] OR colonoscop*[tiab] OR endoscop*[tiab] OR "Physical Examination"[Mesh] OR physical examin*[tiab] OR palpati* OR percussi*[tiab] OR "C-Reactive Protein"[Mesh] OR c reactive protein*[tiab] OR crp[tiab] OR "Leukocyte Count"[Mesh] OR leukocyte count*[tiab] OR white blood cell count*[tiab] OR leukocyte number*[tiab] OR biochemical*[tiab] OR laboratory[tiab] OR blood work[tiab] OR "Cystoscopy"[Mesh] OR cystoscop*[tiab]

1. **Classification**

"Classification"[Mesh] OR "classification" [Subheading] OR classificat*[tiab] OR taxonom*[tiab] OR hierarch*[tiab]

**Explanation of search**

#3 = 1 + 2.2 = diverticulitis + epidemiology specific

#11 = 1 + 2 + (3 | 4 | 5 | 6 | 6.1 | 7 | 8) = diverticulitis + epidemiology broad + risk factors

#14 = 1 + 9 = diverticulitis + mircobiome

#19 = 1 + 10 + (11 | 11.1) = diverticulitis + diagnosis + diagnostic accuracy

#22 = 1 + 12 = diverticulitis + classification

Limits: entrez date in PubMed from 1998.

Exclusion of previous search results; to eliminate duplicates within this topic.

| **Search** | **Query** | **Items found** |
| --- | --- | --- |
| [#23](https://www.ncbi.nlm.nih.gov/pubmed/advanced) | (#22 NOT (#11 OR #3 OR #14 OR #19)) | [60](https://www.ncbi.nlm.nih.gov/pubmed/?cmd=HistorySearch&querykey=23) |
| [#22](https://www.ncbi.nlm.nih.gov/pubmed/advanced) | (#1 AND #21 AND ("1998"[Date - Entrez] : "3000"[Date - Entrez])) | [256](https://www.ncbi.nlm.nih.gov/pubmed/?cmd=HistorySearch&querykey=22) |
| [#21](https://www.ncbi.nlm.nih.gov/pubmed/advanced) | "Classification"[Mesh] OR "classification" [Subheading] OR classificat*[tiab] OR taxonom*[tiab] OR hierarch*[tiab] | [914527](https://www.ncbi.nlm.nih.gov/pubmed/?cmd=HistorySearch&querykey=21) |
| [#20](https://www.ncbi.nlm.nih.gov/pubmed/advanced) | (#19 NOT (#11 OR #3 OR #14)) | [138](https://www.ncbi.nlm.nih.gov/pubmed/?cmd=HistorySearch&querykey=20) |
| [#19](https://www.ncbi.nlm.nih.gov/pubmed/advanced) | (#1 AND #16 AND (#17 OR #18) AND ("1998"[Date - Entrez] : "3000"[Date - Entrez])) | [574](https://www.ncbi.nlm.nih.gov/pubmed/?cmd=HistorySearch&querykey=19) |
| [#18](https://www.ncbi.nlm.nih.gov/pubmed/advanced) | ("Tomography, X-Ray Computed"[Mesh] OR computed tomograph*[tiab] OR ct[tiab] OR cts[tiab] OR cat scan*[tiab] OR computer assisted tomograph*[tiab] OR computerized tomograph*[tiab] OR computed x ray tomograph*[tiab] OR computed xray tomograph*[tiab] OR "Magnetic Resonance Imaging"[Mesh] OR ("magnetic resonance"[tiab] AND (image[tiab] OR images[tiab] OR imaging[tiab])) OR mri[tiab] OR mris[tiab] OR nmr[tiab] OR mra[tiab] OR mras[tiab] OR zeugmatograph*[tiab] OR "mr tomography"[tiab] OR "mr tomographies"[tiab] OR "mr tomographic"[tiab] OR "proton spin"[tiab] OR ((magneti*[tiab] OR "chemical shift"[tiab]) AND imaging[tiab]) OR fmri[tiab] OR fmris[tiab] OR "Ultrasonography"[Mesh] OR "diagnostic imaging"[Subheading] OR ultraso*[tiab] OR sonograph*[tiab] OR echograph*[tiab] OR echocardiograph*[tiab] OR echotomograph*[tiab] OR "Barium Enema"[Mesh] OR barium enema*[tiab] OR "Endoscopy, Digestive System"[Mesh] OR "Colonoscopy"[Mesh] OR colonoscop*[tiab] OR endoscop*[tiab] OR "Physical Examination"[Mesh] OR physical examin*[tiab] OR palpati* OR percussi*[tiab] OR "C-Reactive Protein"[Mesh] OR c reactive protein*[tiab] OR crp[tiab] OR "Leukocyte Count"[Mesh] OR leukocyte count*[tiab] OR white blood cell count*[tiab] OR leukocyte number*[tiab] OR biochemical*[tiab] OR laboratory[tiab] OR blood work[tiab] OR "Cystoscopy"[Mesh] OR cystoscop*[tiab]) | [4172215](https://www.ncbi.nlm.nih.gov/pubmed/?cmd=HistorySearch&querykey=18) |
| [#17](https://www.ncbi.nlm.nih.gov/pubmed/advanced) | ("Diagnosis"[Mesh] OR "diagnosis" [Subheading] OR "Diagnostic Techniques and Procedures"[Mesh] OR diagnos*[tiab]) | [9180672](https://www.ncbi.nlm.nih.gov/pubmed/?cmd=HistorySearch&querykey=17) |
| [#16](https://www.ncbi.nlm.nih.gov/pubmed/advanced) | ("Sensitivity and Specificity"[MeSH] OR specificit*[tw] OR screening[tw] OR accura*[tw] OR reference value*[tw] OR false positive[tw] OR false negative[tw] OR predictive value*[tw] OR roc[tw] OR likelyhood*[tw] OR likelihood*[tw]) | [2336702](https://www.ncbi.nlm.nih.gov/pubmed/?cmd=HistorySearch&querykey=16) |
| [#15](https://www.ncbi.nlm.nih.gov/pubmed/advanced) | (#14 NOT (#11 OR #3)) | [29](https://www.ncbi.nlm.nih.gov/pubmed/?cmd=HistorySearch&querykey=15) |
| [#14](https://www.ncbi.nlm.nih.gov/pubmed/advanced) | (#1 AND #13 AND ("1998"[Date - Entrez] : "3000"[Date - Entrez])) | [61](https://www.ncbi.nlm.nih.gov/pubmed/?cmd=HistorySearch&querykey=14) |
| [#13](https://www.ncbi.nlm.nih.gov/pubmed/advanced) | (("Gastrointestinal Microbiome"[Mesh] OR microbio*[tiab] OR flora[tiab] OR floras[tiab] OR microflora*[tiab])) | [143647](https://www.ncbi.nlm.nih.gov/pubmed/?cmd=HistorySearch&querykey=13) |
| [#12](https://www.ncbi.nlm.nih.gov/pubmed/advanced) | (#11 NOT #3) | [2010](https://www.ncbi.nlm.nih.gov/pubmed/?cmd=HistorySearch&querykey=12) |
| [#11](https://www.ncbi.nlm.nih.gov/pubmed/advanced) | (#1 AND #4 AND (#5 OR #6 OR #7 OR #8 OR #9 OR #10) AND ("1998"[Date - Entrez] : "3000"[Date - Entrez])) | [2956](https://www.ncbi.nlm.nih.gov/pubmed/?cmd=HistorySearch&querykey=11) |
| [#10](https://www.ncbi.nlm.nih.gov/pubmed/advanced) | (("Overweight"[Mesh] OR obese*[tiab] OR overweight*[tiab] OR obesit*[tiab] OR "Body Mass Index"[Mesh] OR "body mass index"[tiab] OR bmi[tiab] OR adipos*[tiab] OR stout[tiab] OR bulky[tiab] OR sizeable[tiab] OR heavy[tiab] OR fat[tiab] OR fatty[tiab] OR greasy[tiab])) | [941245](https://www.ncbi.nlm.nih.gov/pubmed/?cmd=HistorySearch&querykey=10) |
| [#9](https://www.ncbi.nlm.nih.gov/pubmed/advanced) | (("Diet"[Mesh] OR "Dietary Supplements"[Mesh] OR "Food and Beverages"[Mesh] OR diet[tiab] OR diets[tiab] OR supplements[tiab] OR supplement[tiab] OR nutraceutical[tiab] OR neutraceutical[tiab] OR nuts[tiab] OR seeds[tiab] OR popcorn[tiab] OR tomato*[tiab] OR meat[tiab] OR meats[tiab] OR pork[tiab] OR beef[tiab])) | [984690](https://www.ncbi.nlm.nih.gov/pubmed/?cmd=HistorySearch&querykey=9) |
| [#8](https://www.ncbi.nlm.nih.gov/pubmed/advanced) | (("Dietary Fiber"[Mesh] OR dietary fiber*[tiab] OR dietary fibre*[tiab] OR diet fiber*[tiab] OR wheat bran*[tiab] OR roughage*[tiab] OR high fiber diet*[tiab] OR high fibre diet*[tiab] OR alimentary fiber*[tiab] OR alimentary fibre*[tiab])) | [22164](https://www.ncbi.nlm.nih.gov/pubmed/?cmd=HistorySearch&querykey=8) |
| [#7](https://www.ncbi.nlm.nih.gov/pubmed/advanced) | (("Aging"[Mesh] OR "Adult"[Mesh] OR aging*[tiab] OR age[tiab] OR ages[tiab] OR adult*[tiab] OR "Aged"[Mesh] OR "Aged, 80 and over"[Mesh] OR "Frail Elderly"[Mesh] OR "Geriatrics"[Mesh] OR "Geriatric Psychiatry"[Mesh] OR "Geriatric Nursing"[Mesh] OR "Geriatric Dentistry"[Mesh] OR "Dental Care for Aged"[Mesh] OR "Health Services for the Aged"[Mesh] OR elder*[tw] OR eldest[tw] OR frail*[tw] OR geriatri*[tw] OR old age*[tw] OR oldest old*[tw] OR senior*[tw] OR senium[tw] OR very old*[tw] OR septuagenarian*[tw] OR octagenarian*[tw] OR octogenarian*[tw] OR nonagenarian*[tw] OR centarian*[tw] OR centenarian*[tw] OR supercentenarian*[tw] OR older people[tw] OR older subject*[tw] OR older patient*[tw] OR older age*[tw] OR older adult*[tw] OR older man[tw] OR older men[tw] OR older male*[tw] OR older woman[tw] OR older women[tw] OR older female*[tw] OR older population*[tw] OR older person*[tw])) | [7833136](https://www.ncbi.nlm.nih.gov/pubmed/?cmd=HistorySearch&querykey=7) |
| [#6](https://www.ncbi.nlm.nih.gov/pubmed/advanced) | (("Sex"[Mesh] OR "Men"[Mesh:NoExp] OR "Male"[Mesh] OR man[tiab] OR men[tiab] OR male[tiab] OR males[tiab] OR sex[tiab] OR "Female"[Mesh] OR "Women"[Mesh] OR female*[tiab] OR woman[tiab] OR women[tiab] OR feminin*[tiab])) | [10761371](https://www.ncbi.nlm.nih.gov/pubmed/?cmd=HistorySearch&querykey=6) |
| [#5](https://www.ncbi.nlm.nih.gov/pubmed/advanced) | (("Ethnic Groups"[Mesh] OR "Continental Population Groups"[Mesh] OR racial*[tiab] OR race[tiab] OR races[tiab] OR ethnic*[tiab] OR population group*[tiab] OR nationalit*[tiab])) | [394356](https://www.ncbi.nlm.nih.gov/pubmed/?cmd=HistorySearch&querykey=5) |
| [#4](https://www.ncbi.nlm.nih.gov/pubmed/advanced) | ("Morbidity"[Mesh] OR "Mortality"[Mesh] OR "Epidemiologic Studies"[Mesh] OR cohort[tiab] OR (case[tiab] AND (control[tiab] OR controll*[tiab] OR comparison[tiab] OR referent[tiab])) OR risk[tiab] OR causation[tiab] OR causal[tiab] OR "odds ratio"[tiab] OR etiol*[tiab] OR aetiol*[tiab] OR "natural history"[tiab] OR predict*[tiab] OR prognos*[tiab] OR outcome[tiab] OR course[tiab] OR retrospect*[tiab] OR "comparative study"[pt] OR "risk factors"[mesh] OR "cohort"[tw] OR "compared"[tw] OR "groups"[tw] OR "multivariate"[tw] OR inciden*[tiab] OR prevalen*[tiab] OR morbid*[tiab] OR mortal*[tiab] OR epidemiol*[tiab]) | [9818588](https://www.ncbi.nlm.nih.gov/pubmed/?cmd=HistorySearch&querykey=4) |
| [#3](https://www.ncbi.nlm.nih.gov/pubmed/advanced) | (#1 AND #2 AND ("1998"[Date - Entrez] : "3000"[Date - Entrez])) | [1044](https://www.ncbi.nlm.nih.gov/pubmed/?cmd=HistorySearch&querykey=3) |
| [#2](https://www.ncbi.nlm.nih.gov/pubmed/advanced) | ("Morbidity"[Majr] OR "Mortality"[Majr] OR "Epidemiologic Studies"[Majr] OR cohort[ti] OR (case[ti] AND (control[ti] OR controll*[ti] OR comparison[ti] OR referent[ti])) OR risk[ti] OR causation[ti] OR causal[ti] OR "odds ratio"[ti] OR etiol*[ti] OR aetiol*[ti] OR "natural history"[ti] OR predict*[ti] OR prognos*[ti] OR outcome[ti] OR course[ti] OR retrospect*[ti] OR "comparative study"[pt] OR "risk factors"[Majr] OR "cohort"[ti] OR "compared"[ti] OR "groups"[ti] OR "multivariate"[ti] OR inciden*[ti] OR prevalen*[ti] OR morbid*[ti] OR mortal*[ti] OR epidemiol*[ti] OR cohort[ot] OR (case[ot] AND (control[ot] OR controll*[ot] OR comparison[ot] OR referent[ot])) OR risk[ot] OR causation[ot] OR causal[ot] OR "odds ratio"[ot] OR etiol*[ot] OR aetiol*[ot] OR "natural history"[ot] OR predict*[ot] OR prognos*[ot] OR outcome[ot] OR course[ot] OR retrospect*[ot] OR "comparative study"[pt] OR "risk factors"[Majr] OR "cohort"[ot] OR "compared"[ot] OR "groups"[ot] OR "multivariate"[ot] OR inciden*[ot] OR prevalen*[ot] OR morbid*[ot] OR mortal*[ot] OR epidemiol*[ot]) | [3344843](https://www.ncbi.nlm.nih.gov/pubmed/?cmd=HistorySearch&querykey=2) |
| [#1](https://www.ncbi.nlm.nih.gov/pubmed/advanced) | ("Diverticulosis, Colonic"[Mesh] OR "Diverticulum, Colon"[Mesh] OR ((“Colon"[Mesh:NoExp] OR "Colon, Descending"[Mesh] OR "Colon, Sigmoid"[Mesh] OR "Colon, Transverse"[Mesh] OR sigmoid*[tiab] OR colon*[tiab] OR transvers*[tiab] OR descend*[tiab] OR left*[tiab]) AND diverticul*[tiab]) NOT right[ti]) | [10309](https://www.ncbi.nlm.nih.gov/pubmed/?cmd=HistorySearch&querykey=1) |

# Topic 3: Uncomplicated diverticulitis

((nonsurgical*[tiab] OR nonresecti*[tiab] OR nonoperati*[tiab]) AND manag*[tiab]) OR

uncomplicated OR recurren* OR ongoing OR chronic* AND div AND follow up

**(uncomplicated[tiab] OR "hinchey 1"[tiab] OR "hinchey 2"[tiab] OR "hinchey i"[tiab] OR "hinchey ii"[tiab]** OR recurren*[tiab] OR ongoing[tiab] OR chronic*[tiab]**)**

# Topic 4: Complicated diverticulitis

(complicated[tiab] OR "hinchey 3"[tiab] OR "hinchey 4"[tiab] OR “hinchey iii”[tiab] OR “hinchey iv”[tiab] OR acute*[tiab] OR fistula*[tiab] OR stenos* OR strictur* OR peritonitis OR purulent OR fecal OR sepsis OR obstructi* OR bleeding OR stent* OR drainag* OR abscess*)

((nonsurgical*[tiab] OR nonresecti*[tiab] OR nonoperati*[tiab]) AND manag*[tiab]) OR lavage* OR diet ther* OR abx OR pharmacolog* OR mesalazin*

# PubMed 31 Oct 2017 (440+808)

#3 = uncomplicated + diverticulitis

#5 = complicated + diverticulitis

| **Search** | **Query** | **Items found** |
| --- | --- | --- |
| [#5](https://www.ncbi.nlm.nih.gov/pubmed/advanced) | (#1 AND #4) | [808](https://www.ncbi.nlm.nih.gov/pubmed/?cmd=HistorySearch&querykey=5) |
| [#4](https://www.ncbi.nlm.nih.gov/pubmed/advanced) | (complicated[tiab] OR "hinchey 3"[tiab] OR "hinchey 4"[tiab]) | [138503](https://www.ncbi.nlm.nih.gov/pubmed/?cmd=HistorySearch&querykey=4) |
| [#3](https://www.ncbi.nlm.nih.gov/pubmed/advanced) | (#1 AND #2) | [440](https://www.ncbi.nlm.nih.gov/pubmed/?cmd=HistorySearch&querykey=3) |
| [#2](https://www.ncbi.nlm.nih.gov/pubmed/advanced) | (uncomplicated[tiab] OR "hinchey 1"[tiab] OR "hinchey 2"[tiab]) | [31382](https://www.ncbi.nlm.nih.gov/pubmed/?cmd=HistorySearch&querykey=2) |
| [#1](https://www.ncbi.nlm.nih.gov/pubmed/advanced) | ("Diverticulosis, Colonic"[Mesh] OR "Diverticulum, Colon"[Mesh] OR ((“Colon"[Mesh:NoExp] OR "Colon, Descending"[Mesh] OR "Colon, Sigmoid"[Mesh] OR "Colon, Transverse"[Mesh] OR sigmoid*[tiab] OR colon*[tiab] OR transvers*[tiab] OR descend*[tiab] OR left*[tiab]) AND diverticul*[tiab]) NOT right[ti]) | [10318](https://www.ncbi.nlm.nih.gov/pubmed/?cmd=HistorySearch&querykey=1) |

# Bostian search terms 1 Nov 2017

| **Question #** | **MESH terms** | **Textwords** |
| --- | --- | --- |
| **3 (pertinent for all sub-questions)** | diverticulitis [Majr] OR diverticulitis, colonic [Majr] OR ( "Diverticulitis/classification"[Majr] OR "Diverticulitis/complications"[Majr] OR "Diverticulitis/diagnosis"[Majr] OR "Diverticulitis/diet therapy"[Majr] OR "Diverticulitis/drug therapy"[Majr] OR "Diverticulitis/epidemiology"[Majr] OR "Diverticulitis/ethnology"[Majr] OR "Diverticulitis/surgery"[Majr] OR "Diverticulitis/therapy"[Majr] ) |  |
|  | hospitalization |  |
|  |  | bowel rest |
|  | antibiotic prophylaxis | antibiotics |
|  | feces | stool softener |
|  | episode of care | frequency |
|  | signs and symptoms, digestive AND quality of life |  |
|  | frail elderly OR young adult |  |
|  |  | comorbidities |
|  | (infusions, intravenous OR injections, intravenous) OR oral medicine OR pharmacology | pharmacological treatment |
|  | patient discharge OR patient discharge summaries |  |
|  | (diet or diet therapy) OR probiotics |  |
|  | transplants OR transplantation OR transplant recipients) AND (immunosuppressive agents OR immunosuppression) | immunosuppresive medications |
| **3,1** |  | risk factors AND (recurrent diverticulits [tiab] OR chronic diverticulitis [tiab] OR uncomplicated, acute diverticulitis [tiab]) |
| **3,2** |  | non-operative management AND uncomplicated, acute diverticulitis [tiab] |
| **3,3** | follow-up studies | uncomplicated, acute diverticulitis [tiab] |
| **3,4** | (endoscopy [Majr] OR capsule endoscopy [Majr] OR endoscopy, digestive system [Majr] OR endoscopy, gastrointestinal [Majr]) | interval endoscopy AND acute diverticulitis [tiab] |
| **3,5** | surgical procedures, operative | surgical intervention AND uncomplicated diverticular disease [tiab] |
| **3,6** |  | interval sigmoid resection |
|  |  | uncomplicated diverticular disease [tiab] |
| **3,7** |  | non-operative management |
|  |  | uncomplicated, acute diverticulitis [tiab] |
| **4 (pertinent for all sub-questions)** | diverticulitis [Majr] OR diverticulitis, colonic [Majr] OR ( "Diverticulitis/classification"[Majr] OR "Diverticulitis/complications"[Majr] OR "Diverticulitis/diagnosis"[Majr] OR "Diverticulitis/diet therapy"[Majr] OR "Diverticulitis/drug therapy"[Majr] OR "Diverticulitis/epidemiology"[Majr] OR "Diverticulitis/ethnology"[Majr] OR "Diverticulitis/surgery"[Majr] OR "Diverticulitis/therapy"[Majr] ) |  |
|  | antibiotic prophylaxis | antibiotics |
|  | drainage | percutaneous drainage |
|  | stents AND intestinal obstruction | colon stenting AND colon obstruction |
|  | (endoscopy [Majr] OR capsule endoscopy [Majr] OR endoscopy, digestive system [Majr] OR endoscopy, gastrointestinal [Majr]) AND gastrointestinal hemorrhage | endoscopy AND gastrointestinal bleeding |
|  | embolization, therapeutic AND gastrointestinal hemmorrhage | vascular embolization AND gastrointestinal bleeding |
|  | signs and symptoms, digestive AND quality of life |  |
|  |  | comorbidities |
|  | patient preference |  |
|  | frail elderly OR young adult |  |
|  | transplants OR transplantation OR transplant recipients) AND (immunosuppressive agents OR immunosuppression) | immunosuppresive medications |
| **4,1** | intestinal perforation OR abdominal abscess | (non-operative management AND complicated, acute diverticulitis [tiab]) AND localized perforation |
|  | gastrointestinal hemorrhage | gastrointestinal bleeding |
|  | intestinal fistula | colovesical fistula |
| **4,2** | (patient discharge OR patient discharge summaries) AND hospitalization | (recommendations AND discharge) AND complicated diverticulitis [tiab] |
| **4,3** | surgical procedures, operative | (surgical treatment OR surgical intervention) AND acute, complicated diverticulitis [tiab] |
| **4,4** |  | non-operative management AND complicated diverticulitis [tiab] |
| **5 (pertinent for all subquestions)** | diverticulitis [Majr] OR diverticulitis, colonic [Majr] OR ( "Diverticulitis/classification"[Majr] OR "Diverticulitis/complications"[Majr] OR "Diverticulitis/diagnosis"[Majr] OR "Diverticulitis/diet therapy"[Majr] OR "Diverticulitis/drug therapy"[Majr] OR "Diverticulitis/epidemiology"[Majr] OR "Diverticulitis/ethnology"[Majr] OR "Diverticulitis/surgery"[Majr] OR "Diverticulitis/therapy"[Majr] ) |  |
|  | perioperative period AND colorectal surgery | perioperative management AND emergency surgery |
|  | stents | ureteric stents |
|  |  | bowel prep OR intestinal prep |

# PubMed 8 Nov 2017

1. **Diverticulitis**

"Diverticulosis, Colonic"[Mesh] OR "Diverticulum, Colon"[Mesh] OR ((“Colon"[Mesh:NoExp] OR "Colon, Descending"[Mesh] OR "Colon, Sigmoid"[Mesh] OR "Colon, Transverse"[Mesh] OR sigmoid*[tiab] OR colon*[tiab] OR transvers*[tiab] OR descend*[tiab] OR left*[tiab]) AND (**"Diverticulitis"[Mesh] OR** diverticul*[tiab])) NOT right[ti]

# Topic 1: Epidemiology and natural history

**#46 = 0 + 1.1 = diverticulitis + epidemiology specific**

**#58 = 0 + 1 + (2 | 3 | 4 | 5 | 6 | 6.1 | 7) = diverticulitis + epidemiology broad + risk factors**

**#63 = 0 + 8 = diverticulitis + mircobiome**

1. **Epidemiology broad**

"Morbidity"[Mesh] OR "Mortality"[Mesh] OR "Epidemiologic Studies"[Mesh] OR cohort[tiab] OR (case[tiab] AND (control[tiab] OR controll*[tiab] OR comparison[tiab] OR referent[tiab])) OR risk[tiab] OR causation[tiab] OR causal[tiab] OR "odds ratio"[tiab] OR etiol*[tiab] OR aetiol*[tiab] OR "natural history"[tiab] OR predict*[tiab] OR prognos*[tiab] OR outcome[tiab] OR course[tiab] OR retrospect*[tiab] OR "comparative study"[pt] OR "risk factors"[mesh] OR "cohort"[tw] OR "compared"[tw] OR "groups"[tw] OR "multivariate"[tw] OR inciden*[tiab] OR prevalen*[tiab] OR morbid*[tiab] OR mortal*[tiab] OR epidemiol*[tiab]

- 1. **Epidemiology specific: Majr and [ti]**

"Morbidity"[Majr] OR "Mortality"[Majr] OR "Epidemiologic Studies"[Majr] OR cohort[ti] OR (case[ti] AND (control[ti] OR controll*[ti] OR comparison[ti] OR referent[ti])) OR risk[ti] OR causation[ti] OR causal[ti] OR "odds ratio"[ti] OR etiol*[ti] OR aetiol*[ti] OR "natural history"[ti] OR predict*[ti] OR prognos*[ti] OR outcome[ti] OR course[ti] OR retrospect*[ti] OR "comparative study"[pt] OR "risk factors"[Majr] OR "cohort"[ti] OR "compared"[ti] OR "groups"[ti] OR "multivariate"[ti] OR inciden*[ti] OR prevalen*[ti] OR morbid*[ti] OR mortal*[ti] OR epidemiol*[ti] OR cohort[ot] OR (case[ot] AND (control[ot] OR controll*[ot] OR comparison[ot] OR referent[ot])) OR risk[ot] OR causation[ot] OR causal[ot] OR "odds ratio"[ot] OR etiol*[ot] OR aetiol*[ot] OR "natural history"[ot] OR predict*[ot] OR prognos*[ot] OR outcome[ot] OR course[ot] OR retrospect*[ot] OR "comparative study"[pt] OR "risk factors"[Majr] OR "cohort"[ot] OR "compared"[ot] OR "groups"[ot] OR "multivariate"[ot] OR inciden*[ot] OR prevalen*[ot] OR morbid*[ot] OR mortal*[ot] OR epidemiol*[ot]

1. **Ethnicity**

"Ethnic Groups"[Mesh] OR "Continental Population Groups"[Mesh] OR racial*[tiab] OR race[tiab] OR races[tiab] OR ethnic*[tiab] OR population group*[tiab] OR nationalit*[tiab] **OR ethnology[sh]**

1. **Gender**

"Sex"[Mesh] OR "Men"[Mesh:NoExp] OR "Male"[Mesh] OR man[tiab] OR men[tiab] OR male[tiab] OR males[tiab] OR sex[tiab] OR "Female"[Mesh] OR "Women"[Mesh] OR female*[tiab] OR woman[tiab] OR women[tiab] OR feminin*[tiab]

1. **Age**

"Aging"[Mesh] OR "Adult"[Mesh] OR aging*[tiab] OR **ageing*[tiab]** OR age[tiab] OR ages[tiab] OR adult*[tiab] OR "Aged"[Mesh] OR "Aged, 80 and over"[Mesh] OR "Frail Elderly"[Mesh] OR "Geriatrics"[Mesh] OR "Geriatric Psychiatry"[Mesh] OR "Geriatric Nursing"[Mesh] OR "Geriatric Dentistry"[Mesh] OR "Dental Care for Aged"[Mesh] OR "Health Services for the Aged"[Mesh] OR elder*[tw] OR eldest[tw] OR frail*[tw] OR geriatri*[tw] OR old age*[tw] OR oldest old*[tw] OR senior*[tw] OR senium[tw] OR very old*[tw] OR septuagenarian*[tw] OR octagenarian*[tw] OR octogenarian*[tw] OR nonagenarian*[tw] OR centarian*[tw] OR centenarian*[tw] OR supercentenarian*[tw] OR older people[tw] OR older subject*[tw] OR older patient*[tw] OR older age*[tw] OR older adult*[tw] OR older man[tw] OR older men[tw] OR older male*[tw] OR older woman[tw] OR older women[tw] OR older female*[tw] OR older population*[tw] OR older person*[tw]

1. **Diet**

"Diet"[Mesh] OR "Dietary Supplements"[Mesh] OR "Food and Beverages"[Mesh] OR diet[tiab] OR diets[tiab] OR supplements[tiab] OR supplement[tiab] OR nutraceutical[tiab] OR neutraceutical[tiab] OR nuts[tiab] OR seeds[tiab] OR popcorn[tiab] OR tomato*[tiab] OR meat[tiab] OR meats[tiab] OR pork[tiab] OR beef[tiab]

**6.1 Dietary fiber**

"Dietary Fiber"[Mesh] OR dietary fiber*[tiab] OR dietary fibre*[tiab] OR diet fiber*[tiab] OR wheat bran*[tiab] OR roughage*[tiab] OR high fiber diet*[tiab] OR high fibre diet*[tiab] OR alimentary fiber*[tiab] OR alimentary fibre*[tiab]

1. **Stool**

"Constipation"[Mesh] OR "Diarrhea"[Mesh] OR "Feces"[Mesh] OR bowel habit*[tiab] OR constipat*[tiab] OR diarrh*[tiab] OR feces[tiab] OR faeces[tiab] OR fecal*[tiab] OR faecal*[tiab] OR stool[tiab]

1. **BMI**

"Overweight"[Mesh] OR obese*[tiab] OR overweight*[tiab] OR obesit*[tiab] OR "Body Mass Index"[Mesh] OR "body mass index"[tiab] OR bmi[tiab] OR adipos*[tiab] OR stout[tiab] OR bulky[tiab] OR sizeable[tiab] OR heavy[tiab] OR fat[tiab] OR fatty[tiab] OR greasy[tiab]

1. **Microbiome**

"Gastrointestinal Microbiome"[Mesh] OR microbio*[tiab] OR flora[tiab] OR floras[tiab] OR microflora*[tiab]

# Topic 2: Diagnosis and classi­fications

**#69 = 0 + (9 | 9.1) + 10 = diverticulitis + diagnosis + diagnostic accuracy**

**#74 = 0 + 11 = diverticulitis + classification**

1. **Diagnosis**

"Diagnosis"[Mesh] OR "diagnosis" [Subheading] OR "Diagnostic Techniques and Procedures"[Mesh] OR diagnos*[tiab] **OR severit*[tiab] OR assessment*[tiab]**

- 1. **Diagnostic imaging (CT, MRI, US, BE)**

"Tomography, X-Ray Computed"[Mesh] OR computed tomograph*[tiab] OR ct[tiab] OR cts[tiab] OR cat scan*[tiab] OR computer assisted tomograph*[tiab] OR computerized tomograph*[tiab] OR computed x ray tomograph*[tiab] OR computed xray tomograph*[tiab] OR "Magnetic Resonance Imaging"[Mesh] OR ("magnetic resonance"[tiab] AND (image[tiab] OR images[tiab] OR imaging[tiab])) OR mri[tiab] OR mris[tiab] OR nmr[tiab] OR mra[tiab] OR mras[tiab] OR zeugmatograph*[tiab] OR "mr tomography"[tiab] OR "mr tomographies"[tiab] OR "mr tomographic"[tiab] OR "proton spin"[tiab] OR ((magneti*[tiab] OR "chemical shift"[tiab]) AND imaging[tiab]) OR fmri[tiab] OR fmris[tiab] OR "Ultrasonography"[Mesh] OR "diagnostic imaging"[Subheading] OR ultraso*[tiab] OR sonograph*[tiab] OR echograph*[tiab] OR echocardiograph*[tiab] OR echotomograph*[tiab] OR "Barium Enema"[Mesh] OR barium enema*[tiab] OR "Endoscopy, Digestive System"[Mesh] OR "Colonoscopy"[Mesh] OR colonoscop*[tiab] OR endoscop*[tiab] OR "Physical Examination"[Mesh] OR physical examin*[tiab] OR palpati* OR percussi*[tiab] OR "C-Reactive Protein"[Mesh] OR c reactive protein*[tiab] OR crp[tiab] OR "Leukocyte Count"[Mesh] OR leukocyte count*[tiab] OR white blood cell count*[tiab] OR leukocyte number*[tiab] OR biochemical*[tiab] OR laboratory[tiab] OR blood work[tiab] OR "Cystoscopy"[Mesh] OR cystoscop*[tiab]

1. **Diagnostic accuracy**

"Sensitivity and Specificity"[MeSH] OR specificit*[tw] OR screening[tw] OR accura*[tw] OR reference value*[tw] OR false positive[tw] OR false negative[tw] OR predictive value*[tw] OR roc[tw] OR likelyhood*[tw] OR likelihood*[tw]

1. **Classification**

"Classification"[Mesh] OR "classification" [Subheading] OR classificat*[tiab] OR taxonom*[tiab] OR hierarch*[tiab]

| **Search** | **Query** | **Items found** |
| --- | --- | --- |
| [#74](https://www.ncbi.nlm.nih.gov/pubmed/advanced) | (#73 NOT #69) | [185](https://www.ncbi.nlm.nih.gov/pubmed/?cmd=HistorySearch&querykey=74) |
| [#73](https://www.ncbi.nlm.nih.gov/pubmed/advanced) | ((#72 NOT case reports[pt]) AND ("1998"[Date - Entrez] : "3000"[Date - Entrez])) | [233](https://www.ncbi.nlm.nih.gov/pubmed/?cmd=HistorySearch&querykey=73) |
| [#72](https://www.ncbi.nlm.nih.gov/pubmed/advanced) | (#71 NOT (animals[mh] NOT humans[mh])) | [310](https://www.ncbi.nlm.nih.gov/pubmed/?cmd=HistorySearch&querykey=72) |
| [#71](https://www.ncbi.nlm.nih.gov/pubmed/advanced) | (#42 AND #70) | [318](https://www.ncbi.nlm.nih.gov/pubmed/?cmd=HistorySearch&querykey=71) |
| [#70](https://www.ncbi.nlm.nih.gov/pubmed/advanced) | ("Classification"[Mesh] OR "classification" [Subheading] OR classificat*[tiab] OR taxonom*[tiab] OR hierarch*[tiab]) | [916739](https://www.ncbi.nlm.nih.gov/pubmed/?cmd=HistorySearch&querykey=70) |
| [#69](https://www.ncbi.nlm.nih.gov/pubmed/advanced) | ((#68 NOT case reports[pt]) AND ("1998"[Date - Entrez] : "3000"[Date - Entrez])) | [522](https://www.ncbi.nlm.nih.gov/pubmed/?cmd=HistorySearch&querykey=69) |
| [#68](https://www.ncbi.nlm.nih.gov/pubmed/advanced) | (#67 NOT (animals[mh] NOT humans[mh])) | [756](https://www.ncbi.nlm.nih.gov/pubmed/?cmd=HistorySearch&querykey=68) |
| [#67](https://www.ncbi.nlm.nih.gov/pubmed/advanced) | (#42 AND (#64 OR #65) AND #66) | [759](https://www.ncbi.nlm.nih.gov/pubmed/?cmd=HistorySearch&querykey=67) |
| [#66](https://www.ncbi.nlm.nih.gov/pubmed/advanced) | ("Sensitivity and Specificity"[MeSH] OR specificit*[tw] OR screening[tw] OR accura*[tw] OR reference value*[tw] OR false positive[tw] OR false negative[tw] OR predictive value*[tw] OR roc[tw] OR likelyhood*[tw] OR likelihood*[tw]) | [2342024](https://www.ncbi.nlm.nih.gov/pubmed/?cmd=HistorySearch&querykey=66) |
| [#65](https://www.ncbi.nlm.nih.gov/pubmed/advanced) | ("Tomography, X-Ray Computed"[Mesh] OR computed tomograph*[tiab] OR ct[tiab] OR cts[tiab] OR cat scan*[tiab] OR computer assisted tomograph*[tiab] OR computerized tomograph*[tiab] OR computed x ray tomograph*[tiab] OR computed xray tomograph*[tiab] OR "Magnetic Resonance Imaging"[Mesh] OR ("magnetic resonance"[tiab] AND (image[tiab] OR images[tiab] OR imaging[tiab])) OR mri[tiab] OR mris[tiab] OR nmr[tiab] OR mra[tiab] OR mras[tiab] OR zeugmatograph*[tiab] OR "mr tomography"[tiab] OR "mr tomographies"[tiab] OR "mr tomographic"[tiab] OR "proton spin"[tiab] OR ((magneti*[tiab] OR "chemical shift"[tiab]) AND imaging[tiab]) OR fmri[tiab] OR fmris[tiab] OR "Ultrasonography"[Mesh] OR "diagnostic imaging"[Subheading] OR ultraso*[tiab] OR sonograph*[tiab] OR echograph*[tiab] OR echocardiograph*[tiab] OR echotomograph*[tiab] OR "Barium Enema"[Mesh] OR barium enema*[tiab] OR "Endoscopy, Digestive System"[Mesh] OR "Colonoscopy"[Mesh] OR colonoscop*[tiab] OR endoscop*[tiab] OR "Physical Examination"[Mesh] OR physical examin*[tiab] OR palpati* OR percussi*[tiab] OR "C-Reactive Protein"[Mesh] OR c reactive protein*[tiab] OR crp[tiab] OR "Leukocyte Count"[Mesh] OR leukocyte count*[tiab] OR white blood cell count*[tiab] OR leukocyte number*[tiab] OR biochemical*[tiab] OR laboratory[tiab] OR blood work[tiab] OR "Cystoscopy"[Mesh] OR cystoscop*[tiab]) | [4180791](https://www.ncbi.nlm.nih.gov/pubmed/?cmd=HistorySearch&querykey=65) |
| [#64](https://www.ncbi.nlm.nih.gov/pubmed/advanced) | ("Diagnosis"[Mesh] OR "diagnosis" [Subheading] OR "Diagnostic Techniques and Procedures"[Mesh] OR diagnos*[tiab] OR severit*[tiab] OR assessment*[tiab]) | [9738685](https://www.ncbi.nlm.nih.gov/pubmed/?cmd=HistorySearch&querykey=64) |
| [#63](https://www.ncbi.nlm.nih.gov/pubmed/advanced) | (#62 NOT (#57 OR #46)) | [25](https://www.ncbi.nlm.nih.gov/pubmed/?cmd=HistorySearch&querykey=63) |
| [#62](https://www.ncbi.nlm.nih.gov/pubmed/advanced) | ((#61 NOT case reports[pt]) AND ("1998"[Date - Entrez] : "3000"[Date - Entrez])) | [52](https://www.ncbi.nlm.nih.gov/pubmed/?cmd=HistorySearch&querykey=62) |
| [#61](https://www.ncbi.nlm.nih.gov/pubmed/advanced) | (#60 NOT (animals[mh] NOT humans[mh])) | [82](https://www.ncbi.nlm.nih.gov/pubmed/?cmd=HistorySearch&querykey=61) |
| [#60](https://www.ncbi.nlm.nih.gov/pubmed/advanced) | (#42 AND #59) | [83](https://www.ncbi.nlm.nih.gov/pubmed/?cmd=HistorySearch&querykey=60) |
| [#59](https://www.ncbi.nlm.nih.gov/pubmed/advanced) | ("Gastrointestinal Microbiome"[Mesh] OR microbio*[tiab] OR flora[tiab] OR floras[tiab] OR microflora*[tiab]) | [144189](https://www.ncbi.nlm.nih.gov/pubmed/?cmd=HistorySearch&querykey=59) |
| [#58](https://www.ncbi.nlm.nih.gov/pubmed/advanced) | (#57 NOT #46) | [1566](https://www.ncbi.nlm.nih.gov/pubmed/?cmd=HistorySearch&querykey=58) |
| [#57](https://www.ncbi.nlm.nih.gov/pubmed/advanced) | ((#56 NOT case reports[pt]) AND ("1998"[Date - Entrez] : "3000"[Date - Entrez])) | [2478](https://www.ncbi.nlm.nih.gov/pubmed/?cmd=HistorySearch&querykey=57) |
| [#56](https://www.ncbi.nlm.nih.gov/pubmed/advanced) | (#55 NOT (animals[mh] NOT humans[mh])) | [4248](https://www.ncbi.nlm.nih.gov/pubmed/?cmd=HistorySearch&querykey=56) |
| [#55](https://www.ncbi.nlm.nih.gov/pubmed/advanced) | (#42 AND #47 AND (#48 OR #49 OR #50 OR #51 OR #52 OR #53 OR #54)) | [4288](https://www.ncbi.nlm.nih.gov/pubmed/?cmd=HistorySearch&querykey=55) |
| [#54](https://www.ncbi.nlm.nih.gov/pubmed/advanced) | ("Overweight"[Mesh] OR obese*[tiab] OR overweight*[tiab] OR obesit*[tiab] OR "Body Mass Index"[Mesh] OR "body mass index"[tiab] OR bmi[tiab] OR adipos*[tiab] OR stout[tiab] OR bulky[tiab] OR sizeable[tiab] OR heavy[tiab] OR fat[tiab] OR fatty[tiab] OR greasy[tiab]) | [943881](https://www.ncbi.nlm.nih.gov/pubmed/?cmd=HistorySearch&querykey=54) |
| [#53](https://www.ncbi.nlm.nih.gov/pubmed/advanced) | ("Constipation"[Mesh] OR "Diarrhea"[Mesh] OR "Feces"[Mesh] OR bowel habit*[tiab] OR constipat*[tiab] OR diarrh*[tiab] OR feces[tiab] OR faeces[tiab] OR fecal*[tiab] OR faecal*[tiab] OR stool[tiab]) | [272752](https://www.ncbi.nlm.nih.gov/pubmed/?cmd=HistorySearch&querykey=53) |
| [#52](https://www.ncbi.nlm.nih.gov/pubmed/advanced) | ("Dietary Fiber"[Mesh] OR dietary fiber*[tiab] OR dietary fibre*[tiab] OR diet fiber*[tiab] OR wheat bran*[tiab] OR roughage*[tiab] OR high fiber diet*[tiab] OR high fibre diet*[tiab] OR alimentary fiber*[tiab] OR alimentary fibre*[tiab]) | [22208](https://www.ncbi.nlm.nih.gov/pubmed/?cmd=HistorySearch&querykey=52) |
| [#51](https://www.ncbi.nlm.nih.gov/pubmed/advanced) | ("Diet"[Mesh] OR "Dietary Supplements"[Mesh] OR "Food and Beverages"[Mesh] OR diet[tiab] OR diets[tiab] OR supplements[tiab] OR supplement[tiab] OR nutraceutical[tiab] OR neutraceutical[tiab] OR nuts[tiab] OR seeds[tiab] OR popcorn[tiab] OR tomato*[tiab] OR meat[tiab] OR meats[tiab] OR pork[tiab] OR beef[tiab]) | [986748](https://www.ncbi.nlm.nih.gov/pubmed/?cmd=HistorySearch&querykey=51) |
| [#50](https://www.ncbi.nlm.nih.gov/pubmed/advanced) | ("Aging"[Mesh] OR "Adult"[Mesh] OR aging*[tiab] OR ageing*[tiab] OR age[tiab] OR ages[tiab] OR adult*[tiab] OR "Aged"[Mesh] OR "Aged, 80 and over"[Mesh] OR "Frail Elderly"[Mesh] OR "Geriatrics"[Mesh] OR "Geriatric Psychiatry"[Mesh] OR "Geriatric Nursing"[Mesh] OR "Geriatric Dentistry"[Mesh] OR "Dental Care for Aged"[Mesh] OR "Health Services for the Aged"[Mesh] OR elder*[tw] OR eldest[tw] OR frail*[tw] OR geriatri*[tw] OR old age*[tw] OR oldest old*[tw] OR senior*[tw] OR senium[tw] OR very old*[tw] OR septuagenarian*[tw] OR octagenarian*[tw] OR octogenarian*[tw] OR nonagenarian*[tw] OR centarian*[tw] OR centenarian*[tw] OR supercentenarian*[tw] OR older people[tw] OR older subject*[tw] OR older patient*[tw] OR older age*[tw] OR older adult*[tw] OR older man[tw] OR older men[tw] OR older male*[tw] OR older woman[tw] OR older women[tw] OR older female*[tw] OR older population*[tw] OR older person*[tw]) | [7855676](https://www.ncbi.nlm.nih.gov/pubmed/?cmd=HistorySearch&querykey=50) |
| [#49](https://www.ncbi.nlm.nih.gov/pubmed/advanced) | ("Sex"[Mesh] OR "Men"[Mesh:NoExp] OR "Male"[Mesh] OR man[tiab] OR men[tiab] OR male[tiab] OR males[tiab] OR sex[tiab] OR "Female"[Mesh] OR "Women"[Mesh] OR female*[tiab] OR woman[tiab] OR women[tiab] OR feminin*[tiab]) | [10779587](https://www.ncbi.nlm.nih.gov/pubmed/?cmd=HistorySearch&querykey=49) |
| [#48](https://www.ncbi.nlm.nih.gov/pubmed/advanced) | ("Ethnic Groups"[Mesh] OR "Continental Population Groups"[Mesh] OR racial*[tiab] OR race[tiab] OR races[tiab] OR ethnic*[tiab] OR population group*[tiab] OR nationalit*[tiab] OR ethnology[sh]) | [450721](https://www.ncbi.nlm.nih.gov/pubmed/?cmd=HistorySearch&querykey=48) |
| [#47](https://www.ncbi.nlm.nih.gov/pubmed/advanced) | ("Morbidity"[Mesh] OR "Mortality"[Mesh] OR "Epidemiologic Studies"[Mesh] OR cohort[tiab] OR (case[tiab] AND (control[tiab] OR controll*[tiab] OR comparison[tiab] OR referent[tiab])) OR risk[tiab] OR causation[tiab] OR causal[tiab] OR "odds ratio"[tiab] OR etiol*[tiab] OR aetiol*[tiab] OR "natural history"[tiab] OR predict*[tiab] OR prognos*[tiab] OR outcome[tiab] OR course[tiab] OR retrospect*[tiab] OR "comparative study"[pt] OR "risk factors"[mesh] OR "cohort"[tw] OR "compared"[tw] OR "groups"[tw] OR "multivariate"[tw] OR inciden*[tiab] OR prevalen*[tiab] OR morbid*[tiab] OR mortal*[tiab] OR epidemiol*[tiab]) | [9841402](https://www.ncbi.nlm.nih.gov/pubmed/?cmd=HistorySearch&querykey=47) |
| [#46](https://www.ncbi.nlm.nih.gov/pubmed/advanced) | ((#45 NOT case reports[pt])) AND ("1998"[Date - Entrez] : "3000"[Date - Entrez]) | [1000](https://www.ncbi.nlm.nih.gov/pubmed/?cmd=HistorySearch&querykey=46) |
| [#45](https://www.ncbi.nlm.nih.gov/pubmed/advanced) | (#44 NOT (animals[mh] NOT humans[mh])) | [1487](https://www.ncbi.nlm.nih.gov/pubmed/?cmd=HistorySearch&querykey=45) |
| [#44](https://www.ncbi.nlm.nih.gov/pubmed/advanced) | (#42 AND #43) | [1499](https://www.ncbi.nlm.nih.gov/pubmed/?cmd=HistorySearch&querykey=44) |
| [#43](https://www.ncbi.nlm.nih.gov/pubmed/advanced) | ("Morbidity"[Majr] OR "Mortality"[Majr] OR "Epidemiologic Studies"[Majr] OR cohort[ti] OR (case[ti] AND (control[ti] OR controll*[ti] OR comparison[ti] OR referent[ti])) OR risk[ti] OR causation[ti] OR causal[ti] OR "odds ratio"[ti] OR etiol*[ti] OR aetiol*[ti] OR "natural history"[ti] OR predict*[ti] OR prognos*[ti] OR outcome[ti] OR course[ti] OR retrospect*[ti] OR "comparative study"[pt] OR "risk factors"[Majr] OR "cohort"[ti] OR "compared"[ti] OR "groups"[ti] OR "multivariate"[ti] OR inciden*[ti] OR prevalen*[ti] OR morbid*[ti] OR mortal*[ti] OR epidemiol*[ti] OR cohort[ot] OR (case[ot] AND (control[ot] OR controll*[ot] OR comparison[ot] OR referent[ot])) OR risk[ot] OR causation[ot] OR causal[ot] OR "odds ratio"[ot] OR etiol*[ot] OR aetiol*[ot] OR "natural history"[ot] OR predict*[ot] OR prognos*[ot] OR outcome[ot] OR course[ot] OR retrospect*[ot] OR "comparative study"[pt] OR "risk factors"[Majr] OR "cohort"[ot] OR "compared"[ot] OR "groups"[ot] OR "multivariate"[ot] OR inciden*[ot] OR prevalen*[ot] OR morbid*[ot] OR mortal*[ot] OR epidemiol*[ot]) | [3350815](https://www.ncbi.nlm.nih.gov/pubmed/?cmd=HistorySearch&querykey=43) |
| [#42](https://www.ncbi.nlm.nih.gov/pubmed/advanced) | ("Diverticulosis, Colonic"[Mesh] OR "Diverticulum, Colon"[Mesh] OR ((“Colon"[Mesh:NoExp] OR "Colon, Descending"[Mesh] OR "Colon, Sigmoid"[Mesh] OR "Colon, Transverse"[Mesh] OR sigmoid*[tiab] OR colon*[tiab] OR transvers*[tiab] OR descend*[tiab] OR left*[tiab]) AND ("Diverticulitis"[Mesh] OR diverticul*[tiab])) NOT right[ti]) | [10393](https://www.ncbi.nlm.nih.gov/pubmed/?cmd=HistorySearch&querykey=42) |

# Topic 3: Nonsurgical intervention of uncomplicated diverticulitis

**#59 = 0 + 12 + (13 | 13.1)**

1. **Uncomplicated**

uncomplicated[tiab] OR "hinchey 1"[tiab] OR "hinchey 2"[tiab] OR "hinchey i"[tiab] OR "hinchey ii"[tiab] OR recurr*[tiab] OR ongoing[tiab] OR chronic*[tiab]

1. **Nonsurgical intervention**

((nonsurgical*[tiab] OR non surgical*[tiab] OR nonresecti*[tiab] OR non resecti*[tiab] OR nonoperati*[tiab]) AND manag*[tiab]) OR "Diet Therapy"[Mesh] OR "Mesalamine"[Mesh] OR "Pharmaceutical Preparations"[Mesh] OR lavag*[tiab] OR diet[tiab] OR diets[tiab] OR dietary[tiab] OR pharmacolog*[tiab] OR pharmaceutical*[tiab] OR mesalazin*[tiab] OR mesalamin*[tiab]

**13.1 ABX**

"Anti-Bacterial Agents"[Mesh] OR "Anti-Bacterial Agents" [Pharmacological Action] OR "Penicillins"[Mesh] OR antibacterial*[tiab] OR anti bacterial*[tiab] OR antibiotic*[tiab] OR acedapson*[tiab] OR aconiazide*[tiab] OR actinonin*[tiab] OR actinorhodin*[tiab] OR alamethicin*[tiab] OR albomycin*[tiab] OR amdinocillin*[tiab] OR amifloxacin*[tiab] OR amikacin*[tiab] OR aminosalicylic acid*[tiab] OR amoxicillin*[tiab] OR amphomycin*[tiab] OR amphotericin B*[tiab] OR ampicillin*[tiab] OR amprenavir*[tiab] OR angustmycin*[tiab] OR anisomycin*[tiab] OR antimycin*[tiab] OR antofloxacin*[tiab] OR apramycin*[tiab] OR arsphenamin*[tiab] OR aurodox[tiab] OR avibactam*[tiab] OR avilamycin*[tiab] OR azithromycin*[tiab] OR azlocillin*[tiab] OR aztreonam*[tiab] OR bacampicillin*[tiab] OR bacitracin*[tiab] OR bacteriocins*[tiab] OR balofloxacin*[tiab] OR bambermycins*[tiab] OR bedaquilin*[tiab] OR bekanamycin*[tiab] OR benzathine cloxacillin*[tiab] OR berythromycin*[tiab] OR beta lactam*[tiab] OR bialaphos*[tiab] OR bicozamycin*[tiab] OR blasticidin*[tiab] OR bongkrekic Acid*[tiab] OR bredinin*[tiab] OR brefeldin*[tiab] OR broadcillin*[tiab] OR brobactam*[tiab] OR butirosin sulfate*[tiab] OR cactinomycin*[tiab] OR calcimycin*[tiab] OR candicidin*[tiab] OR capreomycin*[tiab] OR carbenicillin*[tiab] OR carfecillin*[tiab] OR cefaclor*[tiab] OR cefadroxil*[tiab] OR cefamandol*[tiab] OR cefatrizin*[tiab] OR cefazedon*[tiab] OR cefazolin*[tiab] OR cefdinir*[tiab] OR cefditoren*[tiab] OR cefepim*[tiab] OR cefetamet*[tiab] OR cefixim*[tiab] OR cefmenoxim*[tiab] OR cefmetazole*[tiab] OR cefminox*[tiab] OR cefodizim*[tiab] OR cefonicid*[tiab] OR cefoperazon*[tiab] OR ceforanide*[tiab] OR cefoselis*[tiab] OR cefotaxim*[tiab] OR cefotetan*[tiab] OR cefotiam*[tiab] OR cefoxitin*[tiab] OR cefpimizol*[tiab] OR cefpiramid*[tiab] OR cefpirom*[tiab] OR cefpodoxim*[tiab] OR cefprozil*[tiab] OR cefsulodin*[tiab] OR ceftazidim*[tiab] OR cefteram pivoxil*[tiab] OR ceftezol*[tiab] OR ceftibuten*[tiab] OR ceftiofur*[tiab] OR ceftizoxime*[tiab] OR ceftobiprole*[tiab] OR ceftriaxon*[tiab] OR cefuroxim*[tiab] OR cefuroxime axetil*[tiab] OR cephacetrile*[tiab] OR cephalexin*[tiab] OR cephaloglycin*[tiab] OR cephaloridin*[tiab] OR cephalosporin*[tiab] OR cephalothin*[tiab] OR cephamycin*[tiab] OR cephapirin*[tiab] OR cephradin*[tiab] OR cethromycin*[tiab] OR chelerythrine*[tiab] OR chloramphenicol*[tiab] OR chloroxin*[tiab] OR chlortetracyclin*[tiab] OR ciprofloxacin*[tiab] OR citrinin*[tiab] OR clarithromycin*[tiab] OR clavulanic acid*[tiab] OR clinafloxacin*[tiab] OR clindamycin*[tiab] OR clofazimin*[tiab] OR cloxacillin*[tiab] OR colistin*[tiab] OR cyclacillin*[tiab] OR cycloserin*[tiab] OR dactinomycin*[tiab] OR dalbavancin*[tiab] OR dalfopristin*[tiab] OR dapson*[tiab] OR daptomycin*[tiab] OR decamethoxin*[tiab] OR demeclocyclin*[tiab] OR desoxyfructo serotonin*[tiab] OR diarylquinoline*[tiab] OR dibekacin*[tiab] OR dicloxacillin*[tiab] OR dihydrostreptomycin sulfate*[tiab] OR diketopiperazin*[tiab] OR dirithromycin*[tiab] OR distamycin*[tiab] OR diucifon*[tiab] OR doxycycline*[tiab] OR dynemicin a[tiab] OR echinomycin*[tiab] OR edeine*[tiab] OR efrotomycin*[tiab] OR emiglitate*[tiab] OR enoxacin*[tiab] OR enviomycin*[tiab] OR epicillin*[tiab] OR ertapenem*[tiab] OR erythromycin*[tiab] OR ethambutol*[tiab] OR ethionamid*[tiab] OR filipin*[tiab] OR florfenicol*[tiab] OR floxacillin*[tiab] OR fluoroquinolon*[tiab] OR forphenicinol*[tiab] OR fosfomycin*[tiab] OR framycetin*[tiab] OR fumagillin*[tiab] OR fusafungin*[tiab] OR fusidic acid*[tiab] OR gamithromycin*[tiab] OR garenoxacin*[tiab] OR gatifloxacin*[tiab] OR gemifloxacin*[tiab] OR gentamicin*[tiab] OR gramicidin*[tiab] OR grepafloxacin*[tiab] OR herbimycin*[tiab] OR hygromycin b[tiab] OR imipenem*[tiab] OR immunomycin*[tiab] OR isatoic anhydride*[tiab] OR isepamicin*[tiab] OR isoniazid*[tiab] OR izumenolid*[tiab] OR josamycin*[tiab] OR kanamycin*[tiab] OR kitasamycin*[tiab] OR lactacystin*[tiab] OR lactam*[tiab] OR lacticin 481[tiab] OR lactoferricin b[tiab] OR lasalocid*[tiab] OR leucomycin*[tiab] OR levofloxacin*[tiab] OR lincomycin*[tiab] OR lincosamid*[tiab] OR lomefloxacin*[tiab] OR loracarbef*[tiab] OR lucensomycin*[tiab] OR lydiamycin a[tiab] OR lymecyclin*[tiab] OR maduramicin*[tiab] OR maltotetraos*[tiab] OR manoalid*[tiab] OR manumycin*[tiab] OR marbofloxacin*[tiab] OR meclocycline*[tiab] OR mepartricin*[tiab] OR meropenem*[tiab] OR methacyclin*[tiab] OR methampicillin*[tiab] OR methicillin*[tiab] OR mevastatin*[tiab] OR mezlocillin*[tiab] OR micronomicin*[tiab] OR midecamycin*[tiab] OR mikamycin*[tiab] OR minocyclin*[tiab] OR miocamycin*[tiab] OR mirincamycin*[tiab] OR mocimycin*[tiab] OR moxalactam*[tiab] OR moxifloxacin*[tiab] OR muconomycin a[tiab] OR mupirocin*[tiab] OR mycobacillin*[tiab] OR nadifloxacin*[tiab] OR nafcillin*[tiab] OR nalidixic acid*[tiab] OR narasin*[tiab] OR natamycin*[tiab] OR nebacetin*[tiab] OR nebramycin*[tiab] OR nebularine*[tiab] OR neomycin*[tiab] OR netilmicin*[tiab] OR netropsin*[tiab] OR nigericin*[tiab] OR nisin[tiab] OR nojirimycin*[tiab] OR norfloxacin*[tiab] OR novobiocin*[tiab] OR nystatin*[tiab] OR ofloxacin*[tiab] OR oleandomycin*[tiab] OR oligomycin*[tiab] OR oxacillin*[tiab] OR oxetanocin*[tiab] OR oxolinic acid*[tiab] OR oxytetracycline*[tiab] OR panipenem betamipron*[tiab] OR paromomycin*[tiab] OR pazufloxacin*[tiab] OR pediocin pa 1[tiab] OR pefloxacin*[tiab] OR penicill*[tiab] OR penimepicyclin*[tiab] OR phenethicillin*[tiab] OR phosphoramidon*[tiab] OR piericidin a[tiab] OR pipemidic acid*[tiab] OR piperacillin*[tiab] OR pivampicillin*[tiab] OR pluracidomycin*[tiab] OR polymyxins*[tiab] OR polyoxorim*[tiab] OR pristinamycin*[tiab] OR prodigiosin*[tiab] OR propicillin*[tiab] OR prothionamid*[tiab] OR prulifloxacin*[tiab] OR pyrazinamid*[tiab] OR pyrazofurin*[tiab] OR quinupristin*[tiab] OR radezolid*[tiab] OR ramoplanin*[tiab] OR ribostamycin*[tiab] OR rifabutin*[tiab] OR rifamexil*[tiab] OR rifampin*[tiab] OR rifamycins*[tiab] OR rifapentine*[tiab] OR ristocetin*[tiab] OR rolitetracyclin*[tiab] OR roxarson*[tiab] OR roxithromycin*[tiab] OR rutamycin*[tiab] OR saframycin a[tiab] OR salinomycin*[tiab] OR sangivamycin*[tiab] OR sirolimus[tiab] OR sisomicin*[tiab] OR sitafloxacin*[tiab] OR sodium thiosulfat*[tiab] OR sparfloxacin*[tiab] OR spectinomycin*[tiab] OR spiramycin*[tiab] OR squalamin*[tiab] OR staphylococcin*[tiab] OR stigmatellin*[tiab] OR streptogramin*[tiab] OR streptomycin*[tiab] OR streptovaricin*[tiab] OR sulbactam*[tiab] OR sulbenicillin*[tiab] OR sulfaguanol*[tiab] OR sulfamerazin*[tiab] OR sulfameter*[tiab] OR sulfamethoxypyridazin*[tiab] OR sulfanilamid*[tiab] OR sultamicillin*[tiab] OR suncillin*[tiab] OR syringomycin*[tiab] OR talampicillin*[tiab] OR tazobactam*[tiab] OR teicoplanin*[tiab] OR telavancin*[tiab] OR telithromycin*[tiab] OR temafloxacin*[tiab] OR temocillin*[tiab] OR tetarimycin*[tiab] OR tetracenomycin*[tiab] OR tetracyclin*[tiab] OR thalidomid*[tiab] OR thiamphenicol*[tiab] OR thienamycin*[tiab] OR thienamycin*[tiab] OR thioacetazon*[tiab] OR thiobenzamid*[tiab] OR thiocarlid*[tiab] OR thiolactomycin*[tiab] OR thiomandelic acid*[tiab] OR thiostrepton*[tiab] OR thymopoietin*[tiab] OR tiamulin*[tiab] OR ticarcillin*[tiab] OR tigecyclin*[tiab] OR tilmicosin*[tiab] OR tobramycin*[tiab] OR tomaymycin*[tiab] OR torezolid*[tiab] OR triostin a[tiab] OR troleandomycin*[tiab] OR tunicamycin*[tiab] OR tylosin*[tiab] OR tyrocidin*[tiab] OR tyrothricin*[tiab] OR ubenimex[tiab] OR ulifloxacin*[tiab] OR undecylprodigiosin*[tiab] OR valinomycin*[tiab] OR vancomycin*[tiab] OR vernamycin b[tiab] OR viomycin*[tiab] OR virginiamycin*[tiab]

# Topic 4: Nonsurgical intervention of complicated diverticulitis

**#63 = 0 + 14 + (15 | 16.1)**

1. **Complicated**

complicated[tiab] OR "hinchey 3"[tiab] OR "hinchey 4"[tiab] OR “hinchey iii”[tiab] OR “hinchey iv”[tiab] OR acute*[tiab] OR "Peritonitis"[Mesh] OR "Fistula"[Mesh] OR "Feces"[Mesh] OR "Sepsis"[Mesh] OR "Hemorrhage"[Mesh] OR "Abscess"[Mesh] OR fistula*[tiab] OR stenos*[tiab] OR strictur*[tiab] OR peritoniti*[tiab] OR purulen*[tiab] OR fecal*[tiab] OR faecal*[tiab] OR feces[tiab] OR faeces[tiab] OR sepsis[tiab] OR obstructi*[tiab] OR bleeding[tiab] OR hemorrhag*[tiab] OR haemorrhag*[tiab] OR stent*[tiab] OR drainag*[tiab] OR abscess*[tiab]

1. **Nonsurgical intervention**

((nonsurgical*[tiab] OR non surgical*[tiab] OR nonresecti*[tiab] OR non resecti*[tiab] OR nonoperati*[tiab]) AND manag*[tiab]) OR "Diet Therapy"[Mesh] OR "Mesalamine"[Mesh] OR "Pharmaceutical Preparations"[Mesh] OR lavag*[tiab] OR diet[tiab] OR diets[tiab] OR dietary[tiab] OR pharmacolog*[tiab] OR pharmaceutical*[tiab] OR mesalazin*[tiab] OR mesalamin*[tiab]

**16.1 ABX**

See: 13.1

# Topic 5: Emergency surgery

**#68 = 0 + 16 + 17**

1. **Emergency**

emergenc*[tiab] OR acute*[tiab] OR complicated[tiab] OR "hinchey 3"[tiab] OR "hinchey 4"[tiab] OR “hinchey iii”[tiab] OR “hinchey iv”[tiab] OR acute*[tiab] OR "Peritonitis"[Mesh] OR "Fistula"[Mesh] OR "Feces"[Mesh] OR "Sepsis"[Mesh] OR "Hemorrhage"[Mesh] OR "Abscess"[Mesh] OR fistula*[tiab] OR stenos*[tiab] OR strictur*[tiab] OR peritoniti*[tiab] OR purulen*[tiab] OR fecal*[tiab] OR faecal*[tiab] OR feces[tiab] OR faeces[tiab] OR sepsis[tiab] OR obstructi*[tiab] OR bleeding[tiab] OR hemorrhag*[tiab] OR haemorrhag*[tiab] OR stent*[tiab] OR drainag*[tiab] OR abscess*[tiab]

1. **Surgery**

"surgery" [Subheading] OR "Surgical Procedures, Operative"[Mesh] OR "Surgeons"[Mesh] OR "Perioperative Period"[Mesh] OR "Perioperative Care"[Mesh] OR "Anesthesia"[Mesh] OR surger*[tiab] OR surgical*[tiab] OR surgeon*[tiab] OR operation*[tiab] OR operative*[tiab] OR perioperati*[tiab] OR anesthe*[tiab] OR anaesthe*[tiab] OR incisi*[tiab] OR extracti*[tiab] OR excisi*[tiab] OR invasive*[tiab] OR restorati*[tiab]

# Topic 6: Elective surgery

**#72 = 0 + 18 + 19**

1. **Elective**

elective*[tiab] OR nonacute*[tiab] OR non acute*[tiab] OR planned[tiab] OR schedule*[tiab] OR uncomplicated[tiab] OR "hinchey 1"[tiab] OR "hinchey 2"[tiab] OR "hinchey i"[tiab] OR "hinchey ii"[tiab] OR recurr*[tiab] OR ongoing[tiab] OR chronic*[tiab]

1. **Surgery**

"surgery" [Subheading] OR "Surgical Procedures, Operative"[Mesh] OR "Surgeons"[Mesh] OR "Perioperative Period"[Mesh] OR "Perioperative Care"[Mesh] OR "Anesthesia"[Mesh] OR surger*[tiab] OR surgical*[tiab] OR surgeon*[tiab] OR operation*[tiab] OR operative*[tiab] OR perioperati*[tiab] OR anesthe*[tiab] OR anaesthe*[tiab] OR incisi*[tiab] OR extracti*[tiab] OR excisi*[tiab] OR invasive*[tiab] OR restorati*[tiab]

# Limits

1. **NOT animal studies**

NOT (animals[mh] NOT humans[mh])

1. **NOT case reports[pt]**
2. **NOT conference abstracts (in Embase)**
3. **> 1998**

("1998"[Date - Entrez] : "3000"[Date - Entrez])

| **Search** | **Query** | **Items found** |
| --- | --- | --- |
| [#72](https://www.ncbi.nlm.nih.gov/pubmed/advanced) | ((#70 NOT case reports[pt]) AND ("1998"[Date - Entrez] : "3000"[Date - Entrez])) | [1089](https://www.ncbi.nlm.nih.gov/pubmed/?cmd=HistorySearch&querykey=72) |
| [#71](https://www.ncbi.nlm.nih.gov/pubmed/advanced) | (#70 NOT (animals[mh] NOT humans[mh])) | [1721](https://www.ncbi.nlm.nih.gov/pubmed/?cmd=HistorySearch&querykey=71) |
| [#70](https://www.ncbi.nlm.nih.gov/pubmed/advanced) | (#42 AND #69 AND #65) | [1727](https://www.ncbi.nlm.nih.gov/pubmed/?cmd=HistorySearch&querykey=70) |
| [#69](https://www.ncbi.nlm.nih.gov/pubmed/advanced) | (elective*[tiab] OR nonacute*[tiab] OR non acute*[tiab] OR planned[tiab] OR schedule*[tiab] OR uncomplicated[tiab] OR "hinchey 1"[tiab] OR "hinchey 2"[tiab] OR "hinchey i"[tiab] OR "hinchey ii"[tiab] OR recurr*[tiab] OR ongoing[tiab] OR chronic*[tiab]) | [1841870](https://www.ncbi.nlm.nih.gov/pubmed/?cmd=HistorySearch&querykey=69) |
| [#68](https://www.ncbi.nlm.nih.gov/pubmed/advanced) | ((#67 NOT case reports[pt]) AND ("1998"[Date - Entrez] : "3000"[Date - Entrez])) | [1798](https://www.ncbi.nlm.nih.gov/pubmed/?cmd=HistorySearch&querykey=68) |
| [#67](https://www.ncbi.nlm.nih.gov/pubmed/advanced) | (#66 NOT (animals[mh] NOT humans[mh])) | [3975](https://www.ncbi.nlm.nih.gov/pubmed/?cmd=HistorySearch&querykey=67) |
| [#66](https://www.ncbi.nlm.nih.gov/pubmed/advanced) | (#42 AND #64 AND #65) | [3990](https://www.ncbi.nlm.nih.gov/pubmed/?cmd=HistorySearch&querykey=66) |
| [#65](https://www.ncbi.nlm.nih.gov/pubmed/advanced) | ("surgery" [Subheading] OR "Surgical Procedures, Operative"[Mesh] OR "Surgeons"[Mesh] OR "Perioperative Period"[Mesh] OR "Perioperative Care"[Mesh] OR "Anesthesia"[Mesh] OR surger*[tiab] OR surgical*[tiab] OR surgeon*[tiab] OR operation*[tiab] OR operative*[tiab] OR perioperati*[tiab] OR anesthe*[tiab] OR anaesthe*[tiab] OR incisi*[tiab] OR extracti*[tiab] OR excisi*[tiab] OR invasive*[tiab] OR restorati*[tiab]) | [4858166](https://www.ncbi.nlm.nih.gov/pubmed/?cmd=HistorySearch&querykey=65) |
| [#64](https://www.ncbi.nlm.nih.gov/pubmed/advanced) | (emergenc*[tiab] OR acute*[tiab] OR complicated[tiab] OR "hinchey 3"[tiab] OR "hinchey 4"[tiab] OR “hinchey iii”[tiab] OR “hinchey iv”[tiab] OR acute*[tiab] OR "Peritonitis"[Mesh] OR "Fistula"[Mesh] OR "Feces"[Mesh] OR "Sepsis"[Mesh] OR "Hemorrhage"[Mesh] OR "Abscess"[Mesh] OR fistula*[tiab] OR stenos*[tiab] OR strictur*[tiab] OR peritoniti*[tiab] OR purulen*[tiab] OR fecal*[tiab] OR faecal*[tiab] OR feces[tiab] OR faeces[tiab] OR sepsis[tiab] OR obstructi*[tiab] OR bleeding[tiab] OR hemorrhag*[tiab] OR haemorrhag*[tiab] OR stent*[tiab] OR drainag*[tiab] OR abscess*[tiab]) | [2656094](https://www.ncbi.nlm.nih.gov/pubmed/?cmd=HistorySearch&querykey=64) |
| [#63](https://www.ncbi.nlm.nih.gov/pubmed/advanced) | ((#62 NOT case reports[pt]) AND ("1998"[Date - Entrez] : "3000"[Date - Entrez])) | [538](https://www.ncbi.nlm.nih.gov/pubmed/?cmd=HistorySearch&querykey=63) |
| [#62](https://www.ncbi.nlm.nih.gov/pubmed/advanced) | (#61 NOT (animals[mh] NOT humans[mh])) | [901](https://www.ncbi.nlm.nih.gov/pubmed/?cmd=HistorySearch&querykey=62) |
| [#61](https://www.ncbi.nlm.nih.gov/pubmed/advanced) | (#42 AND #60 AND (#55 OR #56)) | [909](https://www.ncbi.nlm.nih.gov/pubmed/?cmd=HistorySearch&querykey=61) |
| [#60](https://www.ncbi.nlm.nih.gov/pubmed/advanced) | (complicated[tiab] OR "hinchey 3"[tiab] OR "hinchey 4"[tiab] OR “hinchey iii”[tiab] OR “hinchey iv”[tiab] OR acute*[tiab] OR "Peritonitis"[Mesh] OR "Fistula"[Mesh] OR "Feces"[Mesh] OR "Sepsis"[Mesh] OR "Hemorrhage"[Mesh] OR "Abscess"[Mesh] OR fistula*[tiab] OR stenos*[tiab] OR strictur*[tiab] OR peritoniti*[tiab] OR purulen*[tiab] OR fecal*[tiab] OR faecal*[tiab] OR feces[tiab] OR faeces[tiab] OR sepsis[tiab] OR obstructi*[tiab] OR bleeding[tiab] OR hemorrhag*[tiab] OR haemorrhag*[tiab] OR stent*[tiab] OR drainag*[tiab] OR abscess*[tiab]) | [2435934](https://www.ncbi.nlm.nih.gov/pubmed/?cmd=HistorySearch&querykey=60) |
| [#59](https://www.ncbi.nlm.nih.gov/pubmed/advanced) | ((#58 NOT case reports[pt]) AND ("1998"[Date - Entrez] : "3000"[Date - Entrez])) | [368](https://www.ncbi.nlm.nih.gov/pubmed/?cmd=HistorySearch&querykey=59) |
| [#58](https://www.ncbi.nlm.nih.gov/pubmed/advanced) | (#57 NOT (animals[mh] NOT humans[mh])) | [473](https://www.ncbi.nlm.nih.gov/pubmed/?cmd=HistorySearch&querykey=58) |
| [#57](https://www.ncbi.nlm.nih.gov/pubmed/advanced) | (#42 AND #54 AND (#55 OR #56)) | [474](https://www.ncbi.nlm.nih.gov/pubmed/?cmd=HistorySearch&querykey=57) |
| [#56](https://www.ncbi.nlm.nih.gov/pubmed/advanced) | ("Anti-Bacterial Agents"[Mesh] OR "Anti-Bacterial Agents" [Pharmacological Action] OR "Penicillins"[Mesh] OR antibacterial*[tiab] OR anti bacterial*[tiab] OR antibiotic*[tiab] OR acedapson*[tiab] OR aconiazide*[tiab] OR actinonin*[tiab] OR actinorhodin*[tiab] OR alamethicin*[tiab] OR albomycin*[tiab] OR amdinocillin*[tiab] OR amifloxacin*[tiab] OR amikacin*[tiab] OR aminosalicylic acid*[tiab] OR amoxicillin*[tiab] OR amphomycin*[tiab] OR amphotericin B*[tiab] OR ampicillin*[tiab] OR amprenavir*[tiab] OR angustmycin*[tiab] OR anisomycin*[tiab] OR antimycin*[tiab] OR antofloxacin*[tiab] OR apramycin*[tiab] OR arsphenamin*[tiab] OR aurodox[tiab] OR avibactam*[tiab] OR avilamycin*[tiab] OR azithromycin*[tiab] OR azlocillin*[tiab] OR aztreonam*[tiab] OR bacampicillin*[tiab] OR bacitracin*[tiab] OR bacteriocins*[tiab] OR balofloxacin*[tiab] OR bambermycins*[tiab] OR bedaquilin*[tiab] OR bekanamycin*[tiab] OR benzathine cloxacillin*[tiab] OR berythromycin*[tiab] OR beta lactam*[tiab] OR bialaphos*[tiab] OR bicozamycin*[tiab] OR blasticidin*[tiab] OR bongkrekic Acid*[tiab] OR bredinin*[tiab] OR brefeldin*[tiab] OR broadcillin*[tiab] OR brobactam*[tiab] OR butirosin sulfate*[tiab] OR cactinomycin*[tiab] OR calcimycin*[tiab] OR candicidin*[tiab] OR capreomycin*[tiab] OR carbenicillin*[tiab] OR carfecillin*[tiab] OR cefaclor*[tiab] OR cefadroxil*[tiab] OR cefamandol*[tiab] OR cefatrizin*[tiab] OR cefazedon*[tiab] OR cefazolin*[tiab] OR cefdinir*[tiab] OR cefditoren*[tiab] OR cefepim*[tiab] OR cefetamet*[tiab] OR cefixim*[tiab] OR cefmenoxim*[tiab] OR cefmetazole*[tiab] OR cefminox*[tiab] OR cefodizim*[tiab] OR cefonicid*[tiab] OR cefoperazon*[tiab] OR ceforanide*[tiab] OR cefoselis*[tiab] OR cefotaxim*[tiab] OR cefotetan*[tiab] OR cefotiam*[tiab] OR cefoxitin*[tiab] OR cefpimizol*[tiab] OR cefpiramid*[tiab] OR cefpirom*[tiab] OR cefpodoxim*[tiab] OR cefprozil*[tiab] OR cefsulodin*[tiab] OR ceftazidim*[tiab] OR cefteram pivoxil*[tiab] OR ceftezol*[tiab] OR ceftibuten*[tiab] OR ceftiofur*[tiab] OR ceftizoxime*[tiab] OR ceftobiprole*[tiab] OR ceftriaxon*[tiab] OR cefuroxim*[tiab] OR cefuroxime axetil*[tiab] OR cephacetrile*[tiab] OR cephalexin*[tiab] OR cephaloglycin*[tiab] OR cephaloridin*[tiab] OR cephalosporin*[tiab] OR cephalothin*[tiab] OR cephamycin*[tiab] OR cephapirin*[tiab] OR cephradin*[tiab] OR cethromycin*[tiab] OR chelerythrine*[tiab] OR chloramphenicol*[tiab] OR chloroxin*[tiab] OR chlortetracyclin*[tiab] OR ciprofloxacin*[tiab] OR citrinin*[tiab] OR clarithromycin*[tiab] OR clavulanic acid*[tiab] OR clinafloxacin*[tiab] OR clindamycin*[tiab] OR clofazimin*[tiab] OR cloxacillin*[tiab] OR colistin*[tiab] OR cyclacillin*[tiab] OR cycloserin*[tiab] OR dactinomycin*[tiab] OR dalbavancin*[tiab] OR dalfopristin*[tiab] OR dapson*[tiab] OR daptomycin*[tiab] OR decamethoxin*[tiab] OR demeclocyclin*[tiab] OR desoxyfructo serotonin*[tiab] OR diarylquinoline*[tiab] OR dibekacin*[tiab] OR dicloxacillin*[tiab] OR dihydrostreptomycin sulfate*[tiab] OR diketopiperazin*[tiab] OR dirithromycin*[tiab] OR distamycin*[tiab] OR diucifon*[tiab] OR doxycycline*[tiab] OR dynemicin a[tiab] OR echinomycin*[tiab] OR edeine*[tiab] OR efrotomycin*[tiab] OR emiglitate*[tiab] OR enoxacin*[tiab] OR enviomycin*[tiab] OR epicillin*[tiab] OR ertapenem*[tiab] OR erythromycin*[tiab] OR ethambutol*[tiab] OR ethionamid*[tiab] OR filipin*[tiab] OR florfenicol*[tiab] OR floxacillin*[tiab] OR fluoroquinolon*[tiab] OR forphenicinol*[tiab] OR fosfomycin*[tiab] OR framycetin*[tiab] OR fumagillin*[tiab] OR fusafungin*[tiab] OR fusidic acid*[tiab] OR gamithromycin*[tiab] OR garenoxacin*[tiab] OR gatifloxacin*[tiab] OR gemifloxacin*[tiab] OR gentamicin*[tiab] OR gramicidin*[tiab] OR grepafloxacin*[tiab] OR herbimycin*[tiab] OR hygromycin b[tiab] OR imipenem*[tiab] OR immunomycin*[tiab] OR isatoic anhydride*[tiab] OR isepamicin*[tiab] OR isoniazid*[tiab] OR izumenolid*[tiab] OR josamycin*[tiab] OR kanamycin*[tiab] OR kitasamycin*[tiab] OR lactacystin*[tiab] OR lactam*[tiab] OR lacticin 481[tiab] OR lactoferricin b[tiab] OR lasalocid*[tiab] OR leucomycin*[tiab] OR levofloxacin*[tiab] OR lincomycin*[tiab] OR lincosamid*[tiab] OR lomefloxacin*[tiab] OR loracarbef*[tiab] OR lucensomycin*[tiab] OR lydiamycin a[tiab] OR lymecyclin*[tiab] OR maduramicin*[tiab] OR maltotetraos*[tiab] OR manoalid*[tiab] OR manumycin*[tiab] OR marbofloxacin*[tiab] OR meclocycline*[tiab] OR mepartricin*[tiab] OR meropenem*[tiab] OR methacyclin*[tiab] OR methampicillin*[tiab] OR methicillin*[tiab] OR mevastatin*[tiab] OR mezlocillin*[tiab] OR micronomicin*[tiab] OR midecamycin*[tiab] OR mikamycin*[tiab] OR minocyclin*[tiab] OR miocamycin*[tiab] OR mirincamycin*[tiab] OR mocimycin*[tiab] OR moxalactam*[tiab] OR moxifloxacin*[tiab] OR muconomycin a[tiab] OR mupirocin*[tiab] OR mycobacillin*[tiab] OR nadifloxacin*[tiab] OR nafcillin*[tiab] OR nalidixic acid*[tiab] OR narasin*[tiab] OR natamycin*[tiab] OR nebacetin*[tiab] OR nebramycin*[tiab] OR nebularine*[tiab] OR neomycin*[tiab] OR netilmicin*[tiab] OR netropsin*[tiab] OR nigericin*[tiab] OR nisin[tiab] OR nojirimycin*[tiab] OR norfloxacin*[tiab] OR novobiocin*[tiab] OR nystatin*[tiab] OR ofloxacin*[tiab] OR oleandomycin*[tiab] OR oligomycin*[tiab] OR oxacillin*[tiab] OR oxetanocin*[tiab] OR oxolinic acid*[tiab] OR oxytetracycline*[tiab] OR panipenem betamipron*[tiab] OR paromomycin*[tiab] OR pazufloxacin*[tiab] OR pediocin pa 1[tiab] OR pefloxacin*[tiab] OR penicill*[tiab] OR penimepicyclin*[tiab] OR phenethicillin*[tiab] OR phosphoramidon*[tiab] OR piericidin a[tiab] OR pipemidic acid*[tiab] OR piperacillin*[tiab] OR pivampicillin*[tiab] OR pluracidomycin*[tiab] OR polymyxins*[tiab] OR polyoxorim*[tiab] OR pristinamycin*[tiab] OR prodigiosin*[tiab] OR propicillin*[tiab] OR prothionamid*[tiab] OR prulifloxacin*[tiab] OR pyrazinamid*[tiab] OR pyrazofurin*[tiab] OR quinupristin*[tiab] OR radezolid*[tiab] OR ramoplanin*[tiab] OR ribostamycin*[tiab] OR rifabutin*[tiab] OR rifamexil*[tiab] OR rifampin*[tiab] OR rifamycins*[tiab] OR rifapentine*[tiab] OR ristocetin*[tiab] OR rolitetracyclin*[tiab] OR roxarson*[tiab] OR roxithromycin*[tiab] OR rutamycin*[tiab] OR saframycin a[tiab] OR salinomycin*[tiab] OR sangivamycin*[tiab] OR sirolimus[tiab] OR sisomicin*[tiab] OR sitafloxacin*[tiab] OR sodium thiosulfat*[tiab] OR sparfloxacin*[tiab] OR spectinomycin*[tiab] OR spiramycin*[tiab] OR squalamin*[tiab] OR staphylococcin*[tiab] OR stigmatellin*[tiab] OR streptogramin*[tiab] OR streptomycin*[tiab] OR streptovaricin*[tiab] OR sulbactam*[tiab] OR sulbenicillin*[tiab] OR sulfaguanol*[tiab] OR sulfamerazin*[tiab] OR sulfameter*[tiab] OR sulfamethoxypyridazin*[tiab] OR sulfanilamid*[tiab] OR sultamicillin*[tiab] OR suncillin*[tiab] OR syringomycin*[tiab] OR talampicillin*[tiab] OR tazobactam*[tiab] OR teicoplanin*[tiab] OR telavancin*[tiab] OR telithromycin*[tiab] OR temafloxacin*[tiab] OR temocillin*[tiab] OR tetarimycin*[tiab] OR tetracenomycin*[tiab] OR tetracyclin*[tiab] OR thalidomid*[tiab] OR thiamphenicol*[tiab] OR thienamycin*[tiab] OR thienamycin*[tiab] OR thioacetazon*[tiab] OR thiobenzamid*[tiab] OR thiocarlid*[tiab] OR thiolactomycin*[tiab] OR thiomandelic acid*[tiab] OR thiostrepton*[tiab] OR thymopoietin*[tiab] OR tiamulin*[tiab] OR ticarcillin*[tiab] OR tigecyclin*[tiab] OR tilmicosin*[tiab] OR tobramycin*[tiab] OR tomaymycin*[tiab] OR torezolid*[tiab] OR triostin a[tiab] OR troleandomycin*[tiab] OR tunicamycin*[tiab] OR tylosin*[tiab] OR tyrocidin*[tiab] OR tyrothricin*[tiab] OR ubenimex[tiab] OR ulifloxacin*[tiab] OR undecylprodigiosin*[tiab] OR valinomycin*[tiab] OR vancomycin*[tiab] OR vernamycin b[tiab] OR viomycin*[tiab] OR virginiamycin*[tiab]) | [930752](https://www.ncbi.nlm.nih.gov/pubmed/?cmd=HistorySearch&querykey=56) |
| [#55](https://www.ncbi.nlm.nih.gov/pubmed/advanced) | (((nonsurgical*[tiab] OR non surgical*[tiab] OR nonresecti*[tiab] OR non resecti*[tiab] OR nonoperati*[tiab]) AND manag*[tiab]) OR "Diet Therapy"[Mesh] OR "Mesalamine"[Mesh] OR "Pharmaceutical Preparations"[Mesh] OR lavag*[tiab] OR diet[tiab] OR diets[tiab] OR dietary[tiab] OR pharmacolog*[tiab] OR pharmaceutical*[tiab] OR mesalazin*[tiab] OR mesalamin*[tiab]) | [1579714](https://www.ncbi.nlm.nih.gov/pubmed/?cmd=HistorySearch&querykey=55) |
| [#54](https://www.ncbi.nlm.nih.gov/pubmed/advanced) | (uncomplicated[tiab] OR "hinchey 1"[tiab] OR "hinchey 2"[tiab] OR "hinchey i"[tiab] OR "hinchey ii"[tiab] OR recurr*[tiab] OR ongoing[tiab] OR chronic*[tiab]) | [1626221](https://www.ncbi.nlm.nih.gov/pubmed/?cmd=HistorySearch&querykey=54) |
| [#53](https://www.ncbi.nlm.nih.gov/pubmed/advanced) | (#52 NOT #48) | [185](https://www.ncbi.nlm.nih.gov/pubmed/?cmd=HistorySearch&querykey=53) |
| [#48](https://www.ncbi.nlm.nih.gov/pubmed/advanced) | ((#47 NOT case reports[pt])) AND ("1998"[Date - Entrez] : "3000"[Date - Entrez]) | [522](https://www.ncbi.nlm.nih.gov/pubmed/?cmd=HistorySearch&querykey=48) |
| [#52](https://www.ncbi.nlm.nih.gov/pubmed/advanced) | ((#51 NOT case reports[pt]) AND ("1998"[Date - Entrez] : "3000"[Date - Entrez])) | [233](https://www.ncbi.nlm.nih.gov/pubmed/?cmd=HistorySearch&querykey=52) |
| [#51](https://www.ncbi.nlm.nih.gov/pubmed/advanced) | (#50 NOT (animals[mh] NOT humans[mh])) | [310](https://www.ncbi.nlm.nih.gov/pubmed/?cmd=HistorySearch&querykey=51) |
| [#50](https://www.ncbi.nlm.nih.gov/pubmed/advanced) | (#42 AND #49) | [318](https://www.ncbi.nlm.nih.gov/pubmed/?cmd=HistorySearch&querykey=50) |
| [#49](https://www.ncbi.nlm.nih.gov/pubmed/advanced) | ("Classification"[Mesh] OR "classification" [Subheading] OR classificat*[tiab] OR taxonom*[tiab] OR hierarch*[tiab]) | [916739](https://www.ncbi.nlm.nih.gov/pubmed/?cmd=HistorySearch&querykey=49) |
| [#47](https://www.ncbi.nlm.nih.gov/pubmed/advanced) | (#46 NOT (animals[mh] NOT humans[mh])) | [756](https://www.ncbi.nlm.nih.gov/pubmed/?cmd=HistorySearch&querykey=47) |
| [#46](https://www.ncbi.nlm.nih.gov/pubmed/advanced) | (#42 AND (#43 OR #44) AND #45) | [759](https://www.ncbi.nlm.nih.gov/pubmed/?cmd=HistorySearch&querykey=46) |
| [#45](https://www.ncbi.nlm.nih.gov/pubmed/advanced) | ("Sensitivity and Specificity"[MeSH] OR specificit*[tw] OR screening[tw] OR accura*[tw] OR reference value*[tw] OR false positive[tw] OR false negative[tw] OR predictive value*[tw] OR roc[tw] OR likelyhood*[tw] OR likelihood*[tw]) | [2342024](https://www.ncbi.nlm.nih.gov/pubmed/?cmd=HistorySearch&querykey=45) |
| [#44](https://www.ncbi.nlm.nih.gov/pubmed/advanced) | ("Tomography, X-Ray Computed"[Mesh] OR computed tomograph*[tiab] OR ct[tiab] OR cts[tiab] OR cat scan*[tiab] OR computer assisted tomograph*[tiab] OR computerized tomograph*[tiab] OR computed x ray tomograph*[tiab] OR computed xray tomograph*[tiab] OR "Magnetic Resonance Imaging"[Mesh] OR ("magnetic resonance"[tiab] AND (image[tiab] OR images[tiab] OR imaging[tiab])) OR mri[tiab] OR mris[tiab] OR nmr[tiab] OR mra[tiab] OR mras[tiab] OR zeugmatograph*[tiab] OR "mr tomography"[tiab] OR "mr tomographies"[tiab] OR "mr tomographic"[tiab] OR "proton spin"[tiab] OR ((magneti*[tiab] OR "chemical shift"[tiab]) AND imaging[tiab]) OR fmri[tiab] OR fmris[tiab] OR "Ultrasonography"[Mesh] OR "diagnostic imaging"[Subheading] OR ultraso*[tiab] OR sonograph*[tiab] OR echograph*[tiab] OR echocardiograph*[tiab] OR echotomograph*[tiab] OR "Barium Enema"[Mesh] OR barium enema*[tiab] OR "Endoscopy, Digestive System"[Mesh] OR "Colonoscopy"[Mesh] OR colonoscop*[tiab] OR endoscop*[tiab] OR "Physical Examination"[Mesh] OR physical examin*[tiab] OR palpati* OR percussi*[tiab] OR "C-Reactive Protein"[Mesh] OR c reactive protein*[tiab] OR crp[tiab] OR "Leukocyte Count"[Mesh] OR leukocyte count*[tiab] OR white blood cell count*[tiab] OR leukocyte number*[tiab] OR biochemical*[tiab] OR laboratory[tiab] OR blood work[tiab] OR "Cystoscopy"[Mesh] OR cystoscop*[tiab]) | [4180791](https://www.ncbi.nlm.nih.gov/pubmed/?cmd=HistorySearch&querykey=44) |
| [#43](https://www.ncbi.nlm.nih.gov/pubmed/advanced) | ("Diagnosis"[Mesh] OR "diagnosis" [Subheading] OR "Diagnostic Techniques and Procedures"[Mesh] OR diagnos*[tiab] OR severit*[tiab] OR assessment*[tiab]) | [9738685](https://www.ncbi.nlm.nih.gov/pubmed/?cmd=HistorySearch&querykey=43) |
| [#42](https://www.ncbi.nlm.nih.gov/pubmed/advanced) | ("Diverticulosis, Colonic"[Mesh] OR "Diverticulum, Colon"[Mesh] OR ((“Colon"[Mesh:NoExp] OR "Colon, Descending"[Mesh] OR "Colon, Sigmoid"[Mesh] OR "Colon, Transverse"[Mesh] OR sigmoid*[tiab] OR colon*[tiab] OR transvers*[tiab] OR descend*[tiab] OR left*[tiab]) AND ("Diverticulitis"[Mesh] OR diverticul*[tiab])) NOT right[ti]) | [10393](https://www.ncbi.nlm.nih.gov/pubmed/?cmd=HistorySearch&querykey=42) |
