# Supplementary figures and images for: EAES and SAGES 2018 consensus conference on acute diverticulitis management: evidence-based recommendations for clinical practice
Source: Surg Endosc. 2019 Jun 27;33(9):2726–41. doi: 10.1007/s00464-019-06882-z (PMC6684540; doi:10.1007/s00464-019-06882-z)

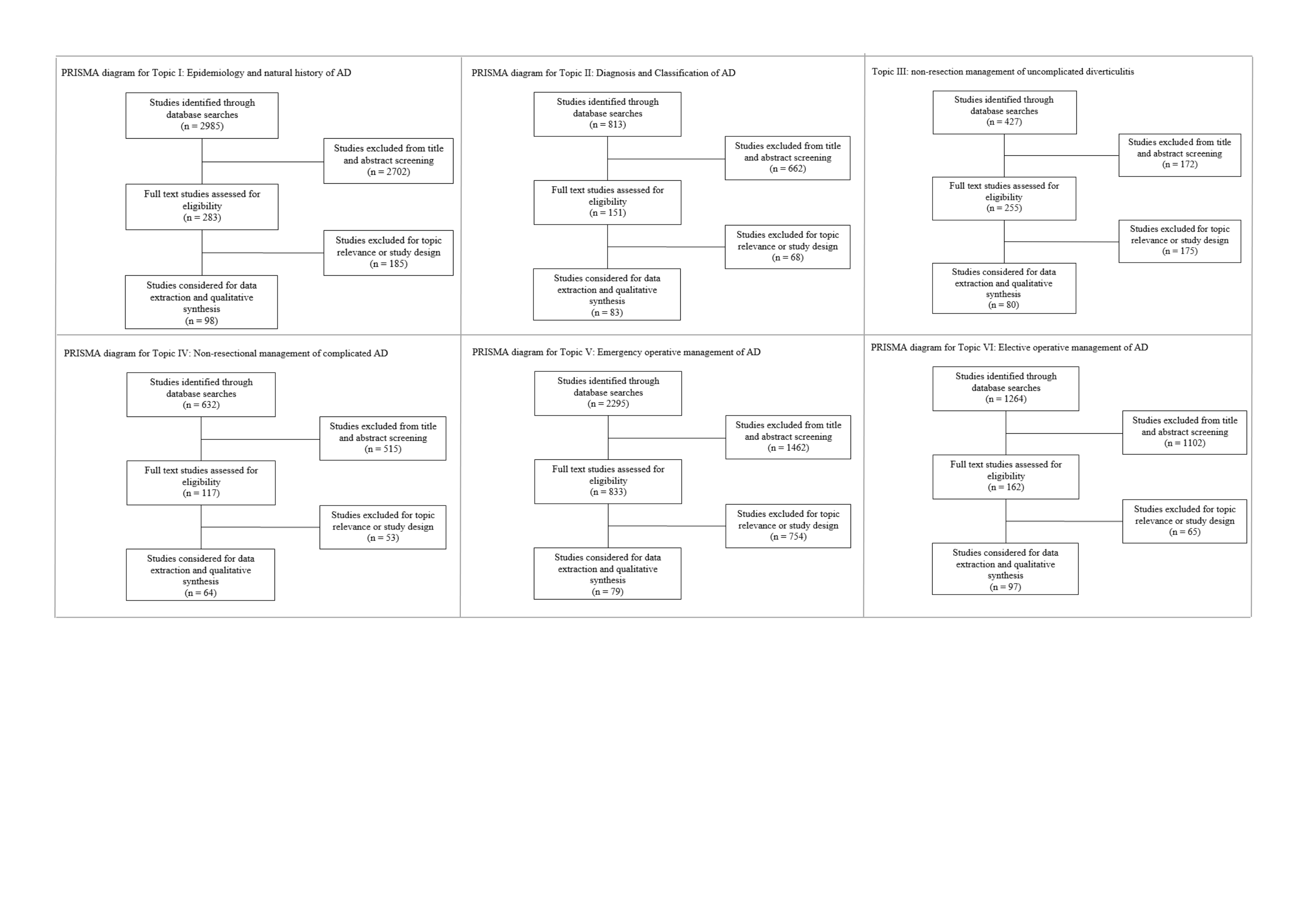

Supplement: Supplementary file 2 — Supplementary Fig. 1: PRISMA flow charts for all six acute diverticulitis topics.. Supplementary material 2 (TIFF 1480 kb) [file 464_2019_6882_MOESM2_ESM.tif]
