# Supplementary material for: EAES and SAGES 2018 consensus conference on acute diverticulitis management: evidence-based recommendations for clinical practice
Source: Surg Endosc. 2019 Jun 27;33(9):2726–41. doi: 10.1007/s00464-019-06882-z (PMC6684540; doi:10.1007/s00464-019-06882-z)
Supplement: Supplementary file 3 — Supplementary materials 1–6: Full text literature review results reporting the AD evidence base and rationale behind all statements and recommendations formulated in the consensus conference. Supplementary material 3 (DOCX 84 kb) [file 464_2019_6882_MOESM3_ESM.docx]

**Topic I: Epidemiology and natural history of acute diverticulitis**

**Introduction**

Topic 1 pertains to the epidemiology and natural history of diverticulitis and is further divided into four areas concerning the epidemiology, risk factors, and the role of the microbiome in the development of acute diverticulitis. A total of 2985 articles resulted from the search of our topic. The search was based on the following terms: age, ethnicity, gender, age, diet, constipation and body mass index (BMI).

We ultimately included 98 publications that were relevant toward supporting the statements formulated concerning this topic. These were largely in the shape of well-powered, prospective and retrospective studies concerning acute left-sided diverticulitis. Case reports, animal studies, narrative reviews, expert opinion pieces, and editorials were excluded.

**Q1.1:  *What is the incidence and prevalence of left-sided acute diverticulitis?***

**Statement: Admission rates for left-sided acute diverticulitis are increasing. The highest rates of increase are occurring in those under 40-years of age.**

Numerous retrospective studies from North America, Europe, and eastern Asia have suggested that acute diverticulitis as well as its complications have become increasingly more prevalent in the new millennium. Within the United States, Bharucha et al found that the incidence of acute diverticulitis increased by 50% in the period between 2000-2007 when compared to that between 1990-1999[1]. The increase was most apparent in those under fifty years of age. While diverticulosis and its complications have historically been considered ailments of the elderly, the increasing prevalence of symptomatic diverticular disease in younger individuals has significantly altered the face of the disease and its corresponding management. Etzioni examined admission rates for acute diverticulitis in California between 1995 and 2006 and found significant increases in rates of hospitalization and elective surgical management for diverticulitis; the changes in these rates over the study period were most pronounced in those between twenty and thirty-four years old (estimated percent annual change 8.6%) and those between thirty-five and forty years old (estimated percent annual change 5.7%) [2]. Similar trends have been noted by other groups in North America, including West [3], Adler [4], and Razik [5]. Vather and colleagues described similar epidemiologic data in New Zealand, where patients under the age of sixty comprised 38% of admissions for acute diverticulitis between 2000 and 2012 [6]. In the Netherlands, Unlu found that 32% of patients admitted for four hospitals for acute diverticulitis were under fifty years of age [7].

The same findings have been observed in Europe and even in Asia, where the western diet and lifestyle have become a new-normal in the era of modernity and globalization. In the United Kingdom, Jejayarah and colleagues found a doubling in admission rates from 0.56 to 1.20 per 1000 patient years between 1996 and 2006 [8]. Similar findings were illustrated in Scotland by Paterson [9] and in Norway by Jamal [10]. Manabe et al describe significant increases in admissions for acute diverticulitis to various hospitals within Japan [11]. Finally, while the prevalence of colonic diverticulosis in Africa has been shown to be on the rise as well, data on the development of acute diverticulitis throughout the continent remains largely anecdotal, limited to small case series and case reports.

*→ LoE: Moderate*

*→ Strength of recommendation: n.a.*

**Statement: In patients over the age of 50, acute diverticulitis occurs more frequently in females; in those under 50 years of age, it occurs more commonly in males.**

As we have noted above, various retrospective and prospective cohort studies support the argument that acute diverticulitis is no longer strictly a disease of the elderly. Within the younger population, complications related to diverticular disease are more likely to afflict males. Several groups have illustrated this finding. Lahat and colleagues prospectively followed 207 patients who were hospitalized for acute diverticulitis; in those under forty-five years of age, there was a male predominance (3:1 male-to-female ratio), whereas in those over forty-five years old there was a female predominance (2:1 female-to-male ratio). It is important to note that younger patients did not have significantly more severe disease [12].

Faria et al replicated this in Portugal, in a retrospective review of 157 patients admitted with acute diverticulitis at a single hospital between 2004 and 2007 [13]. There was a striking male predominance in those under fifty-years-old, with a male:female ratio of 1.8:1. Those over the age of fifty showed a female predominance with a male:female ratio of 0.61:1. The difference between both groups according to gender was significant (P= 0.008). Lopez-Boraro also demonstrated this epidemiologic finding in Spain, where a retrospective analysis was conducted on 686 patients admitted with a first episode of diverticulitis between 1998-2008. Males had decreasing incidence of admissions for diverticulitis as they aged. They comprised 78% of patients under forty-five years of age, 50.4% of patients aged between forty-five and seventy years, and 33.5% of patients over seventy-years-old (p<0.001) [14]. Makela, Schneider, and Reisman showed similar findings in prospective studies in Finland, the United States, and Israel, respectively [15, 16, 17].

*→ LoE: Moderate*

*→ Strength of recommendation: n.a.*

**Statement: Individuals from regions where the prevalence of diverticulitis is low experience a gradual increase in incidence following migration to the west and acculturation to the western lifestyle.**

Historically, the prevalence of diverticular disease and its associated complications in Eastern Asia and Africa have been far overshadowed by those in the West. The western diet (comparatively rich in refined sugar and saturated fat, and low in fiber) and lifestyle have long been implicated as the underlying root causes of this finding. With increasingly lightening constraints on international travel and the influences of globalization, however, a migratory effect on the prevalence of diverticular disease has been noted. Hjern and colleagues conducted a prospective cohort study comparing rates of acute diverticulitis admissions amongst native Swedes to those amongst non-Western immigrants to Sweden. After adjustment for age, sex and socioeconomic indicators, admission rates were lower in non-Western immigrants (RRs=0.5–0.7) compared with those of natives. However, the risk for inpatient admission in the latter group was found to increase in a stepwise fashion with time after settlement [18]. Ostensibly, acculturation to the so-called western lifestyle has at least some influence on this phenomenon. Similar trends illustrating the propensity of those from low-risk areas who migrate to high-risk regions to develop symptomatic diverticular disease have been reproduced in studies involving other traditionally low-risk ethnic groups, including Bangladeshi immigrants to the United Kingdom and Middle Eastern, African, and South American migrants to other countries within western Europe.

*→ LoE: Low*

*→ Strength of recommendation: n.a.*

**Statement: There is seasonal and geographic variation in the prevalence of acute diverticulitis which has been replicated in all hemispheres, with peak incidence occurring in the summer months**.

Several retrospective, epidemiologic studies have illustrated a correlation between southern latitude and the summer season and admission rates for acute diverticulitis. A corresponding decline in admission rates during winter months has also been observed. Relative dehydration, dietary changes, and fluctuating vitamin D levels may play a substantive role in this phenomenon, which has been duplicated in other inflammatory illnesses of the bowel such as acute appendicitis. Adler et al reviewed hospital admissions for diverticulitis in the US, UK, and Australia between 2008 and 2013, with summer peaks being present in all three countries. On regression analysis, the seasonal association proved significant in both hemispheres [19]. Furthermore, within the United States, admission rates for acute diverticulitis are significantly higher in southern states when compared to those in states in the northeast, midwest, and west. While cultural and dietary differences may play roles in these findings, climate differences have been hypothesized to contribute as well. However, the effect and ramifications of local climate on the risk to developing acute diverticulitis may not be as straightforward as it would appear. Regions with low UV exposure displayed significantly higher admission rates for perforated disease and higher rates of emergent colectomy in a retrospective study by Maguire and colleagues [20]. UV fluctuation may also have a secondary effect on seasonal influence, with the increased summer admission trend being more pronounced in areas with high annual UV variability.

*→ LoE: Moderate*

*→ Strength of recommendation: n.a.*

**Q1.2:  *What factors are associated with an increased risk of developing acute diverticulitis*?**

**Statement: Long term NSAID, corticosteroid, and opiate use has been associated with increased risk of perforation in the setting of acute left-sided diverticulitis.**

Perforation may result from a combination of increased intracolonic pressure and impairment of the colonic mucosal barrier. Drugs that either increase intracolonic pressure or weaken the mucosal barrier might therefore represent potential risk factors [21].

Several retrospective cohort studies have shown a strong association between the use of NSAIDs, corticosteroids and opioids and the risk of perforation [22-27]. Use of corticosteroids and opioids have shown a risk which is two- to threefold greater compared to controls, even when correcting for comorbidities and confounding factors [22]. The risk of developing other complications were not described.  It is hypothesized that opioids slow the colonic transit time and may therefore cause fecal stasis and thus raise intraluminal pressure which may increase the risk on inflammation and complications [21, 28]. NSAID use causes a decrease in prostaglandin synthesis and alterations in large intestine permeability which serve to decrease colonic wall integrity [22, 23]. Corticosteroids are powerful immunosuppressive drugs that may impair the normal protective responses to inflammation and infection. Chronic corticosteroid use also reduces collagen turnover and might weaken the colonic wall [21]. The risk of both inhaled and oral steroid use has been studied in rheumatologic patient cohorts [24] and has shown a strong dose-response association with the development of perforation [26]. No association has been found between steroid use and the development of diverticulitis in diverticulosis patients. Particularly when used on a daily basis, opioids are associated with an increased risk of diverticular perforation [21]. Furthermore, this increased risk of perforation with medication use has been demonstrated in patients in the west but has not been duplicated in patients in eastern Asia [25, 29]. Therefore, it has been hypothesized that ‘diverticulitis,’ as an entity, may denote distinct disease processes in the east and west.

*Level of evidence: Low*

*Strength of recommendation: n/a*

**Statement:** **Calcium channel blocker and statin therapy may lower the risk for colonic perforation and the need for emergency surgery in patients with symptomatic diverticular disease.**

Four case-control studies have investigated the hypothesized protective effect of Calcium-channel blocker therapy and statin therapy on the development of colonic perforation in acute diverticulitis [22, 28, 30, 31].

Both studies of Morris et al and Piekarek et al have shown reduced rates of diverticular perforation in patient groups using Ca-Channel blockers. Humes et al have not identified an increased risk of diverticular perforation in Ca-antagonist and aspirin users, though have not shown a reduced rate either.  Calcium channel blockers have been reported to decrease the frequency of high-pressure colonic contractions and improve mucosal blood flow, this potential mechanism was thought responsible for the prior finding of a reduced association between users of these drugs and diverticular perforation [22, 30].

Statins have emerged as potential anti-inflammatory agents as they apparently reduce the pro-inflammatory tendencies of macrophages and neutrophils [22, 32] . It has also been shown that statins may have an anti-inflammatory effect through HMG-CoA reductase inhibitor Fluvastatin, which reduces the severity of colitis in dextransodium sulphate-treated rodents in a model for ulcerative colitis [22, 33]. Humes et al has shown that statin use was associated with a reduction in the risk of perforation, this was confirmed by Skoldberg et al, who have shown that current statin use reduces the risk on emergency surgery, which is linked to diverticular complications. Statin use does not seem to influence the development of diverticular disease in general.

*Level of evidence: Moderate*

*Strength of recommendation: n.a.*

**Statement: Increased BMI and increased visceral-to-subcutaneous fat ratio are associated with an increased risk for acute diverticulitis.**

Multiple studies have shown an association between obesity, increased BMI, and/or visceral fat ratio to the development of acute diverticulitis in those with diverticulosis compared to healthy controls [34-36].

Hjern et al found that women with a BMI > 30 had an increased risk of 33% on diverticular disease and have a twofold higher risk for complicated disease [37]. Rodriguez-Wong et al have found any BMI greater than 25 to be associated as a risk factor for complicated diverticular disease.  Lee et al have compared visceral to subcutaneous fat ratio in healthy controls to patients with diverticulosis to patients with diverticulitis, though have not found significant differences.

The pathophysiology remains poorly understood but is thought to be secondary to high baseline levels of circulating inflammatory mediators as the lipid catabolism is increased in the acute inflammatory phase. Serum receptor for advanced glycation end products (sRAGE) is a receptor in serum which competes with gut-receptors for AGEs. sRAGE was significantly reduced in obese male and in males with diverticulosis. As higher quantities are known to limit inflammation, this reduction might thus be correlated with the development of diverticulitis [38].

Some studies have shown that a higher BMI is also associated with higher rates of complications of diverticulitis including perforation and recurrence and higher rates of surgical treatment [34, 35, 39].

On the contrary, Hjern et al have also shown that a low BMI, defined as below 20, also seems to be associated with a significantly increased risk for complicated disease. Although this does not account for diverticular disease in general. The mechanism for this remains unknown but might be associated with general weakness and therefore higher chance on complications.

*Level of evidence: Moderate*

*Strength of recommendation: n.a.*

**Statement: Physical activity is associated with a diminished risk of complicated diverticulitis**

Two studies have shown a clear association between physical inactivity and development of complicated diverticulitis [37, 40]. No relationships were found between the development of uncomplicated diverticulosis and physical inactivity. It is thought to be linked to the development of diverticular disease by effects on gastrointestinal motor function, decreased intracolonic pressure, reduced transit time which in turn may reduce fecal stasis and bacterial overgrowth and colonization. Moreover, neuroendocrine changes with effect on the gastrointestinal tract through regulation of motilin, prostaglandins and catecholamine’s are thought to play a role.

Hjern et al [37] have shown that physical inactivity, which the authors define as less than 30 minutes per day, is associated with higher rates of hospital admission. Still, no associations with the rate of perforation or the development of intraabdominal abscesses were found.

A linear association between increase in physical activity and reduction of diverticulitis and its complication rates was found in the study of Strate et al [40]. Those performing vigorous activity, running specifically, regularly seemed to have the lowest incidence on both diverticulitis and its complications.  Of particular relevance to these findings, it was hypothesized that the up and down motion of jogging and running may impart distinct benefits to the colon perhaps by stimulating defecation [41].  A statistically significant inverse association was seen between total physical activity and the risk of diverticulitis and diverticular bleeding. However, non-vigorous activity was not significantly associated with either outcome.

*Level of evidence: Moderate*

*Strength of recommendation: n.a.*

**Statement: Smoking has been associated with increased risk of acute diverticulitis.**

The pathophysiology of diverticular disease as well as the possible effect of smoking on the development of the disease and its complications is poorly understood.

Controversial results are presented regarding the relationship between smoking and the development of acute diverticulitis.

Bahadursingh et al [42] described 24% of diverticulitis patients as being smokers, of which the authors infer that this is the average percentage of smokers in the region’s population and that smoking in and of itself likely does not confer additional risk. Papagrigoriadis [43] et al have inferred that more diverticulitis patients have a history of smoking, but their results did not reach significance. Several other studies have shown that a history of smoking is positively associated with the diagnosis of diverticulitis [35, 44], others have shown a positive association with the development of complicated diverticulitis [45-47]. This increased risk associated with smoking might be due to increases in VIP (vasointestinal polypeptide) levels in the colonic mucosa [48] and thus colonic motility and intraluminal pressure [49] and may thereby enhance the bulging of colonic mucosa. Smoking also impairs blood supply to the colonic mucosa [47, 50, 51] which can lead to weakening of the colon wall and subsequent damage.

Kikuchi et al [52] observed that chronic nicotine stimulation affects the immune response of lamina propria T cells to the Th1-dominant pattern via nAChR by up-regulation of the Th1-specific transcriptional factor. Therefore, the nicotine-dependent Th1 modulation, as found in Th1-type enteritis such as Crohn’s disease, could increase the risk of mucosal inflammation in patients with diverticulosis.

*Level of evidence: Moderate*

*Strength of recommendation: n.a*

**Q1.3:  *Are there any other risk factors in specific patient groups?***

**Statement: Ehler-Danlos, Marfan’s, and Williams-Beuren syndromes are associated with an increased risk of the development of acute diverticulitis.**

Many studies report Ehler-Danlos, Marfan’s and Williams-Beuren syndromes to be associated with the development of diverticulosis, but only some describe the development acute diverticulitis in these subgroups. The hypothesis behind this increased risk for both diverticulosis and diverticulitis is due to a disrupted collagen metabolism [53]. Bode et al [54] have shown that collagen III ratio is increased in diverticulosis, which led to investigating the link between collagen I:III ratios in relation to age and the development of diverticulitis.  Brown et al [55] found that diverticulitis in the younger patient was not associated with a lower type I:III collagen ratio. It appears that the decrease in collagen ratio of the colon with age is associated with an increase in type III collagen deposition. The study of Ulmer et al [56] revealed a significantly higher collagen type I/III ratio for the control group compared to patients with complicated diverticular disease. This indicates that changes in collagen ratio does seem to be responsible for the development of diverticulosis and diverticulitis, which might explain the predisposition of patients with certain syndromes to diverticular disease.

Both Connelly [53]and Leganger [57] presented data of subgroups of patients with Ehler-Danlos Syndrome, a known disorder secondary to collagen 5A1 or 5A2 gene mutations, which show that a higher occurrence of overall diverticular events and admissions with diverticulitis were found compared to the general population.

Partsch et al [58] have investigated a large group of Williams-Beuren patients, in which 10.9% had proven diverticulosis, whereas in the general population this is around 2%. Of these, 71% developed diverticulitis, this reported incidence of development of acute diverticulitis is three times higher than in the normal population.

*Level of evidence: low*

*Strength of recommendation: n.a.*

**Statement: Patients with HIV and those undergoing chemotherapy are at increased risk for developing acute diverticulitis.**

Traditionally, patients on chronic immunosuppression therapy have been identified as a high-risk population for increased risk of complicated and recurrent diverticulitis.

Several series report increased morbidity and mortality from acute diverticulitis in immunosuppressed patients and a high likelihood that non-operative management will fail in this population. In a study by Samdani et al [59] a cohort of patients currently undergoing chemotherapy did not differ significantly from the control group in terms of severity of index episode of acute diverticulitis, failure of non-operative management, and rate of recurrence. Chemotherapy patients did recur with more severe disease and were more likely to undergo emergent surgery, and they were significantly more likely to have postoperative complications following interval resection. Perioperative morbidity and mortality were not increased relative to control patients. These data argue against routine surgical intervention in patients undergoing chemotherapy who present with diverticulitis, in both the acute and recurrent settings. It is still unclear whether this risk can be attributed to the history of malignancy and higher rates of corticosteroid use in chemotherapy patients, or to chemotherapy alone.

As the association with diverticulitis and immunosuppression has been made multiple times, HIV might also be associated with an increased risk for diverticulitis. Cronley et al [60] is the only group to have investigated this risk thus far. As the life-expectancy of HIV patients is consistently rising, the prevalence of diverticulitis in this population of patients has accordingly increased. Moreover, patients with HIV infection that are hospitalized for diverticulitis had a mortality rate almost four times higher than those who were HIV negative and hospitalized for diverticulitis.

Both for the association of diverticulitis with HIV and with chemotherapy, more research needs to be done to determine the associated risks. However, it seems likely that immunosuppression plays a significant role in the development of diverticulitis and its complications.

*Level of evidence: moderate*

*Strength of recommendation: n.a.*

**Q1.4:  What is the microbiome profile in acute diverticulitis?**

**Statement: Bifidobacteria and phylum proteobacteria are more abundant in patients presenting with acute diverticulitis.**

The local milieu of the gut, more specifically the microbiome, has become an area of strong interest in understanding the pathophysiology of a wide spectrum of gastrointestinal maladies. The available literature on this subject and its role in the pathophysiology of acute diverticulitis is sparse and limited to low-powered prospective series with small sample sizes, though continues to evolve. Examination of stool samples demonstrates that patients with acute diverticulitis possess a higher diversity of fecal bacteria when compared to healthy controls. Proteobacteria and bifidobacteria make up this difference in several papers. It is thought that such organisms continuously induce immune responses and secrete proinflammatory mediators, and in turn put the patient with diverticulosis at risk for the development of its inflammatory complications thereof. Moreover, the anaerobic bifidobacteria lead to excessive deposition of elastin within the colonic wall, leading to its weakening and a corresponding predisposal to diverticular disease. The role of small bowel bacterial overgrowth and its potential implications on the development of acute diverticulitis in those with preexisting colonic diverticular disease, has also been illustrated by Tursi and colleagues [61].

*→LoE: Low*

*→ Strength of recommendation: n.a.*

**Statement: H. pylori infection may confer protection against the development of complications in those with diverticular disease.**

The role of H. pylori in the development of peptic ulcer disease was established by Marshall and Warren in 1984 [62]. Recent work on the gram negative bacteria has largely focused on its effects on the risk for the development of inflammatory bowel disease, as well as allergy and asthma in urban populations. The relevance of H. pylori infection, and its potential effect on the risk for developing acute diverticulitis, largely remain as an area of ongoing investigation. A 2017 prospective study of 56,000 patients by Bartels et al examined this relationship [63]. Individuals underwent a urea breath test (UBT) and were then followed for six years and monitored for the development of diverticular disease and its complications. Those born between 1910-1920 were more likely to have a positive UBT than those born between 1980-1990 (34% vs. 11%); those with a positive UBT were significantly less likely to have symptomatic diverticular disease, including acute diverticulitis with and without perforation (0.87% vs. 1.14%, OR 0.62, 95% CI 0.50-0.78). This inverse association did not exist following treatment for H. pylori amongst subjects who had a positive UBT, suggesting that H. pylori may confer protection against the development of acute diverticulitis. An explanation for this finding is unknown, however the authors posit that it may result from direct mucosal immune modulation by the bacteria. More work is needed before a definitive role can be elucidated between H. pylori infection and its risk on the development of acute diverticulitis in those with known colonic diverticular disease.

*→ LoE: low*

*→ Strength of recommendation: n.a.*

**References**

1. Bharucha AE, Parthasarathy G, Ditah I, et al. Temporal Trends in the Incidence and Natural History of Diverticulitis: A Population-Based Study. Am J Gastroenterol. 2015;110(11):1589-96.
2. Etzioni DA, Cannom RR, Ault GT, Beart RW, Kaiser AM. Diverticulitis in California from 1995 to 2006: increased rates of treatment for younger patients. Am Surg. 2009;75(10):981-5.
3. West SD, Robinson EK, Delu AN, Ligon RE, Kao LS, Mercer DW. Diverticulitis in the younger patient. Am J Surg. 2003;186(6):743-6.
4. Adler JT, Chang DC, Chan AT, Faiz O, Maguire LH. Seasonal Variation in Diverticulitis: Evidence From Both Hemispheres. Dis Colon Rectum. 2016;59(9):870-7.
5. Razik R, Chong CA, Nguyen GC. Younger age and prognosis in diverticulitis: a nationwide retrospective cohort study. Can J Gastroenterol. 2013;27(2):95-8.
6. Vather R, Broad JB, Jaung R, Robertson J, Bissett IP. Demographics and trends in the acute presentation of diverticular disease: a national study. ANZ J Surg. 2015;85(10):744-8.
7. Ünlü C, Daniels L, Vrouenraets BC, Boermeester MA. A systematic review of high-fibre dietary therapy in diverticular disease. Int J Colorectal Dis. 2012;27(4):419-27.
8. Jeyarajah S, Faiz O, Bottle A, et al. Diverticular disease hospital admissions are increasing, with poor outcomes in the elderly and emergency admissions. Aliment Pharmacol Ther. 2009;30(11-12):1171-82.
9. Paterson HM, Arnott ID, Nicholls RJ, et al. Diverticular disease in Scotland: 2000-2010. Colorectal Dis. 2015;17(4):329-34.
10. Jamal talabani A, Lydersen S, Endreseth BH, Edna TH. Major increase in admission- and incidence rates of acute colonic diverticulitis. Int J Colorectal Dis. 2014;29(8):937-45.
11. Manabe N, Haruma K, Nakajima A, et al. Characteristics of Colonic Diverticulitis and Factors Associated With Complications: A Japanese Multicenter, Retrospective, Cross-Sectional Study. Dis Colon Rectum. 2015;58(12):1174-81.
12. Lahat A, Avidan B, Sakhnini E, Katz L, Fidder HH, Meir SB. Acute diverticulitis: a decade of prospective follow-up. J Clin Gastroenterol. 2013;47(5):415-9.
13. Faria GR, Almeida AB, Moreira H, Pinto-de-sousa J, Correia-da-silva P, Pimenta AP. Acute diverticulitis in younger patients: any rationale for a different approach?. World J Gastroenterol. 2011;17(2):207-12.
14. Lopez-borao J, Kreisler E, Millan M, et al. Impact of age on recurrence and severity of left colonic diverticulitis. Colorectal Dis. 2012;14(7):e407-12.
15. Mäkelä JT, Kiviniemi HO, Laitinen ST. Spectrum of disease and outcome among patients with acute diverticulitis. Dig Surg. 2010;27(3):190-6.
16. Schneider EB, Singh A, Sung J, et al. Emergency department presentation, admission, and surgical intervention for colonic diverticulitis in the United States. Am J Surg. 2015;210(2):404-7.
17. Reisman Y, Ziv Y, Kravrovitc D, Negri M, Wolloch Y, Halevy A. Diverticulitis: the effect of age and location on the course of disease. Int J Colorectal Dis. 1999;14(4-5):250-4.
18. Hjern F, Johansson C, Mellgren A, Baxter NN, Hjern A. Diverticular disease and migration--the influence of acculturation to a Western lifestyle on diverticular disease. Aliment Pharmacol Ther. 2006;23(6):797-805.
19. Adler JT, Chang DC, Chan AT, Faiz O, Maguire LH. Seasonal Variation in Diverticulitis: Evidence From Both Hemispheres. Dis Colon Rectum. 2016;59(9):870-7.
20. Maguire LH, Song M, Strate LL, Giovannucci EL, Chan AT. Association of geographic and seasonal variation with diverticulitis admissions. JAMA Surg. 2015;150(1):74-7.
21. Morris CR, Harvey IM, Stebbings WS, Speakman CT, Kennedy HJ, Hart AR. Anti-inflammatory drugs, analgesics and the risk of perforated colonic diverticular disease. Br J Surg. 2003;90(10):1267-72.
22. Humes DJ, Fleming KM, Spiller RC, West J. Concurrent drug use and the risk of perforated colonic diverticular disease: a population-based case-control study. Gut. 2011;60(2):219-24.
23. Goh H, Bourne R. Non-steroidal anti-inflammatory drugs and perforated diverticular disease: a case-control study. Ann R Coll Surg Engl. 2002;84(2):93-6.
24. Mpofu S, Mpofu CM, Hutchinson D, Maier AE, Dodd SR, Moots RJ. Steroids, non-steroidal anti-inflammatory drugs, and sigmoid diverticular abscess perforation in rheumatic conditions. Ann Rheum Dis. 2004;63(5):588-90.
25. Chang SS, Hu HY. Long-term use of steroids protects from the development of symptomatic diverticulitis requiring hospitalization in the Asian population. PLoS One. 2015;10(4):e0124598.
26. Hjern F, Mahmood MW, Abraham-Nordling M, Wolk A, Hakansson N. Cohort study of corticosteroid use and risk of hospital admission for diverticular disease. Br J Surg. 2015;102(1):119-24.
27. von Rahden BH, Kircher S, Thiery S, Landmann D, Jurowich CF, Germer CT, et al. Association of steroid use with complicated sigmoid diverticulitis: potential role of activated CD68+/CD163+ macrophages. Langenbecks Arch Surg. 2011;396(6):759-68
28. Piekarek K, Israelsson LA. Perforated colonic diverticular disease: the importance of NSAIDs, opioids, corticosteroids, and calcium channel blockers. Int J Colorectal Dis. 2008;23(12):1193-7.
29. Ho BL, Hu HY, Chang SS. Association between use of proton pump inhibitors and occurrence of colon diverticulitis. J Chin Med Assoc. 2016;79(1):5-10.
30. Morris CR, Harvey IM, Stebbings WS, Speakman CT, Kennedy HJ, Hart AR. Do calcium channel blockers and antimuscarinics protect against perforated colonic diverticular disease? A case control study. Gut. 2003;52(12):1734-7.
31. Skoldberg F, Svensson T, Olen O, Hjern F, Schmidt PT, Ljung R. A population-based case-control study on statin exposure and risk of acute diverticular disease. Scand J Gastroenterol. 2016;51(2):203-10.
32. Terblanche M, Almog Y, Rosenson RS, Smith TS, Hackam DG. Statins and sepsis: multiple modifications at multiple levels. The Lancet Infectious diseases. 2007;7(5):358-68.
33. Suzuki S, Tajima T, Sassa S, Kudo H, Okayasu I, Sakamoto S. Preventive effect of fluvastatin on ulcerative colitis-associated carcinogenesis in mice. Anticancer research. 2006;26(6b):4223-8.
34. Dobbins C, Defontgalland D, Duthie G, Wattchow DA. The relationship of obesity to the complications of diverticular disease. Colorectal Dis. 2006;8(1):37-40.
35. Harvey J, Roberts PL, Schoetz DJ, Hall JF, Read TE, Marcello PW, et al. Do Appendicitis and Diverticulitis Share a Common Pathological Link? Dis Colon Rectum. 2016;59(7):656-61.
36. Wolf C. Diverticulitis: the relationship between body mass index and disease location, recurrence, and complications. Gastroenterol Nurs. 2012;35(1):46-51.
37. Hjern F, Wolk A, Hakansson N. Obesity, physical inactivity, and colonic diverticular disease requiring hospitalization in women: a prospective cohort study. Am J Gastroenterol. 2012;107(2):296-302.
38. Comstock SS, Lewis MM, Pathak DR, Hortos K, Kovan B, Fenton JI. Cross-sectional analysis of obesity and serum analytes in males identifies sRAGE as a novel biomarker inversely associated with diverticulosis. PLoS One. 2014;9(4).
39. Rodriguez-Wong U, Cruz-Rubin C, Pinto-Angulo VM, Garcia Alvarez J. [Obesity and complicated diverticular disease of the colon]. Cir Cir. 2015;83(4):292-6.
40. Strate LL, Liu YL, Aldoori WH, Giovannucci EL. Physical activity decreases diverticular complications. Am J Gastroenterol. 2009;104(5):1221-30.
41. Sullivan SN. The effect of running on the gastrointestinal tract. J Clin Gastroenterol. 1984;6(5):461-5.
42. Bahadursingh AM, Virgo KS, Kaminski DL, Longo WE. Spectrum of disease and outcome of complicated diverticular disease. Am J Surg. 2003;186(6):696-701
43. Papagrigoriadis S, Macey L, Bourantas N, Rennie JA. Smoking may be associated with complications in diverticular disease. Br J Surg. 1999;86(7):923-6.
44. Usai P, Ibba I, Lai M, Boi MF, Savarese MF, Cuomo R, et al. Cigarette smoking and appendectomy: effect on clinical course of diverticulosis. Dig Liver Dis. 2011;43(2):98-101.
45. Park NS, Jeen YT, Choi HS, Kim ES, Kim YJ, Keum B, et al. Risk factors for severe diverticulitis in computed tomography-confirmed acute diverticulitis in Korea. Gut Liver. 2013;7(4):443-9.
46. Yoo PS, Garg R, Salamone LF, Floch MH, Rosenthal R, Longo WE. Medical comorbidities predict the need for colectomy for complicated and recurrent diverticulitis. Am J Surg. 2008;196(5):710-4.
47. Turunen P, Wikstrom H, Carpelan-Holmstrom M, Kairaluoma P, Kruuna O, Scheinin T. Smoking increases the incidence of complicated diverticular disease of the sigmoid colon. Scand J Surg. 2010;99(1):14-7.
48. Miotto D, Boschetto P, Bononi I, Zeni E, Cavallesco G, Fabbri LM, et al. Vasoactive intestinal peptide receptors in the airways of smokers with chronic bronchitis. The European respiratory journal. 2004;24(6):958-63.
49. Milner P, Crowe R, Kamm MA, Lennard-Jones JE, Burnstock G. Vasoactive intestinal polypeptide levels in sigmoid colon in idiopathic constipation and diverticular disease. Gastroenterology. 1990;99(3):666-75.
50. Fawcett A, Shembekar M, Church JS, Vashisht R, Springall RG, Nott DM. Smoking, hypertension, and colonic anastomotic healing; a combined clinical and histopathological study. Gut. 1996;38(5):714-8.
51. Zimmerman DD, Gosselink MP, Mitalas LE, Delemarre JB, Hop WJ, Briel JW, et al. Smoking impairs rectal mucosal bloodflow--a pilot study: possible implications for transanal advancement flap repair. Dis Colon Rectum. 2005;48(6):1228.
52. Kikuchi H, Itoh J, Fukuda S. Chronic nicotine stimulation modulates the immune response of mucosal T cells to Th1-dominant pattern via nAChR by upregulation of Th1-specific transcriptional factor. Neuroscience letters. 2008;432(3):217-21.
53. Connelly TM, Choi CS, Berg AS, Harris L, 3rd, Coble J, Koltun WA. Diverticulitis and Crohn's disease have distinct but overlapping tumor necrosis superfamily 15 haplotypes. J Surg Res. 2017;214:262-9.
54. Bode MK, Karttunen TJ, Makela J, Risteli L, Risteli J. Type I and III collagens in human colon cancer and diverticulosis. Scand J Gastroenterol. 2000;35(7):747-52.
55. Brown SR, Cleveland EM, Deeken CR, Huitron SS, Aluka KJ, Davis KG. Type I/type III collagen ratio associated with diverticulitis of the colon in young patients. J Surg Res. 2017;207:229-34.
56. Ulmer TF, Rosch R, Mossdorf A, Alizai H, Binnebosel M, Neumann U. Colonic wall changes in patients with diverticular disease - is there a predisposition for a complicated course? Int J Surg. 2014;12(5):426-31.
57. Leganger J, Soborg MK, Mortensen LQ, Gregersen R, Rosenberg J, Burcharth J. Association between diverticular disease and Ehlers-Danlos syndrome: a 13-year nationwide population-based cohort study. Int J Colorectal Dis. 2016;31(12):1863-7.
58. Partsch CJ, Siebert R, Caliebe A, Gosch A, Wessel A, Pankau R. Sigmoid diverticulitis in patients with Williams-Beuren syndrome: relatively high prevalence and high complication rate in young adults with the syndrome. Am J Med Genet A. 2005;137(1):52-4.
59. Samdani T, Pieracci FM, Eachempati SR, Benarroch-Gampel J, Weiss A, Pietanza MC, et al. Colonic diverticulitis in chemotherapy patients: should operative indications change? A retrospective cohort study. Int J Surg. 2014;12(12):1489-94.
60. Cronley K, Wenzke J, Hussan H, Vasquez AM, Hinton A, El-Dika S, et al. Diverticulitis in HIV-infected patients within the United States. HIV Med. 2016;17(3):216-21.
61. Tursi A, Brandimarte G, Giorgetti GM, Elisei W. Assessment of small intestinal bacterial overgrowth in uncomplicated acute diverticulitis of the colon. World J Gastroenterol. 2005;11(18):2773-6.
62. Marshall BJ, Warren RM. Unidentified curved bacilli in the stomach of patients with gastritis and peptic ulceration. Lancet. 1984;16:1311–1315.
63. Bartels LE, Jepsen P, Tøttrup A, Vilstrup H, Dahlerup JF. Helicobacter pylori infection is associated with reduced prevalence of colonic diverticular disease. Helicobacter. 2017;22(4).
