# Supplementary material for: EAES and SAGES 2018 consensus conference on acute diverticulitis management: evidence-based recommendations for clinical practice
Source: Surg Endosc. 2019 Jun 27;33(9):2726–41. doi: 10.1007/s00464-019-06882-z (PMC6684540; doi:10.1007/s00464-019-06882-z)
Supplement: Supplementary file 4 — Supplementary material 4 (DOCX 229 kb) [file 464_2019_6882_MOESM4_ESM.docx]

***Topic II: Diagnosis and classification of acute diverticulitis***

***Diverticulitis Classification System:***

**Statement: There are multiple classification systems for acute diverticulitis. None has been conclusively demonstrated to be superior to another in predicting patient outcomes and therefore, a specific recommendation cannot be provided.**

**GRADE: Low-quality evidence, no applicable recommendation**

Comment

There are over ten classification schemata that have been created to classify the severity of diverticulitis or peritonitis associated with diverticulitis ^1,2^. Examples of these classification systems include the modified Hinchey Classification, the Hansen-Stock Classification, the Ambrosetti Classification, the Mannheim Peritonitis Index (MPI), the American Association for the Surgery of Trauma (AAST) grading system for acute colonic diverticulitis, the Cleveland Clinic Diverticular Disease Propensity Score (CCDDP), the Boostrom Classification, and the Kruis classification ^1-10^. Available classification systems, in general, classify acute diverticulitis as complicated or non-complicated based on the presence or absence, respectively, of an abscess, fistula, or free intra-peritoneal contamination ^1-9^. Complicated diverticulitis is further subdivided into contained perforation (perforation with an abscess either pericolic or distant) and uncontained perforation (free intra-peritoneal contamination with either purulent or feculent peritonitis) ^1-9^.

There is only one retrospective cohort study that directly compares scoring systems ^1,2^. Sallinen et al included 631 patients and calculated the area under the curve (AUC) for several classification schemata and created their own classification similar to the Hinchey classification system with an additional stage for patients with generalized peritonitis and organ dysfunction ^1^. The AUC for predicting 30-day mortality, need for operation, and ICU admission was 0.89-0.96 for the Hinchey Classification, Hansen-Stock Classification, and Sallinen Classification but was only 0.74-0.80 for the Ambrosetti Classification ^1^.

In 8 retrospective and 1 prospective studies, higher Hinchey Classification has been associated with higher mortality, operative intervention, and complications ^8,11-18^. In addition, in 6 retrospective and 1 prospective studies, higher MPI has also been associated with higher mortality ^2,12,18-23^. In one large, retrospective, multicenter cohort study, the AAST diverticulitis grading system was found to be associated with complications, need for surgery, intensive care unit utilization, and 30-day readmission ^6^.

While several classification systems of acute diverticulitis have been shown to predict patient outcomes, comparative data among existing systems are severely limited and do not allow firm recommendations. Given that the modified Hinchey classification has received the most attention in the literature and has been used in several clinical trials on diverticulitis ^24-27^ it will be used for the purposes of these guidelines.

***Diverticulitis Diagnosis: History and Exam:***

**Statement: Left-lower quadrant abdominal pain and tenderness in the absence of vomiting are the clinical features most consistent with diverticulitis.**

**Recommendation: A focused history and physical exam are recommended for all patients with suspected diverticulitis.**

**GRADE: Moderate-quality evidence, strong recommendation**

Comment:

A history and physical exam is essential for the diagnosis of diverticulitis. The clinical diagnosis of diverticulitis has been found to have a sensitivity of 64-71% and a specificity of 97-98% based on two systematic reviews that included both prospective and retrospective observational studies ^5,28-32^. In one large prospective study of 1021 patients of whom 101 had diverticulitis, left-lower quadrant abdominal pain and tenderness in the absence of vomiting were the most consistent clinical findings for the diagnosis of diverticulitis (AUC of 0.72 and 0.64 respectively) ^31^. The combination of left-lower quadrant pain, absence of vomiting, and a C-reactive protein (CRP)>50 mg/L has been found to be very specific (93-100%) but not very sensitive (24-37%) for the diagnosis of diverticulitis in 2 systematic reviews of retrospective and prospective studies ^5,30,31,33^.

Elevated body temperature has been found to not be useful to diagnose or stage diverticulitis with an AUC of only 0.54-0.57 and a sensitivity of 28% and specificity of 87% for colonic perforation ^29,31,34,35^. One retrospective study found that normalization of fevers in the first 48 hours after medical management of diverticulitis predicted early discharge with oral antibiotics ^36^. Duration of complaint, rapidity of onset, progressive nature of pain, or diarrhea have also not been found to be significantly associated with the diagnosis of diverticulitis s^31,37^.

***Diverticulitis Diagnosis: Laboratory Work-Up:***

**Statement: Numerous studies have demonstrated the diagnostic and prognostic value of C-reactive protein for patients with acute diverticulitis.**

**Recommendation: We recommend that CRP be included in the laboratory evaluation of a patient with acute diverticulitis.**

**GRADE: Moderate-quality evidence, Strong recommendation**

Comment:

Studies on the laboratory evaluation of patients with diverticulitis have assessed the utility of CRP, white blood cell (WBC) count, hyponatremia, and the fecal microbiome. CRP is the best established laboratory marker and has been found to be important for diagnosis and prognostication based on 3 systematic reviews ^3,5,14,29-31,34,35,38-42^. An elevated CRP (defined as a CRP>50 mg/L) has been found to have an AUC of 0.63-0.81 for the diagnosis of diverticulitis ^29,31,38^. Multiple studies have also found strong associations between increased levels of CRP and complicated diverticulitis ^14,34,35,39-41,43^. The optimal cutoff of CRP to define complicated disease is not well-defined and ranges between 50 mg/L and 175 mg/L in different studies. A cutoff of 150 mg/L for example is associated with a specificity of 75-91% and a sensitivity of 44-88% for complicated disease ^34,39,40^. In general, higher cutoffs are associated with improved specificity at the cost of worse sensitivity.

Elevated WBC count has not been found to be sensitive nor specific for the diagnosis of diverticulitis (AUC of 0.59-0.61, sensitivity=47-66%, specificity=22-64% (2 prospective; 1 retrospective)) or the severity of the disease (AUC of 0.544, sensitivity=74-88%, specificity=39-44% (4 retrospective studies)) ^29,34,35,38,39,42,44,45^. On the other hand, improvements of the WBC 48 hours after initial diagnosis has been associated with improved outcomes ^36^.

One small retrospective study found that hyponatremia was 79% specific (but only 31% sensitive) for the diagnosis of diverticulitis ^35^. The fecal microbiome and other immunologic markers, such as interleukin-6 and lipopolysaccharide binding protein, have also been implicated in the diagnosis and prognostication of diverticulitis, but are not widely studied or utilized at this time ^46,47^.

***Diverticulitis Diagnosis: Imaging***

**Statement: Patients with pain localized to the left lower quadrant, absence of vomiting, and a CRP>50 mg/l are highly likely to have acute diverticulitis.**

**Recommendation: We recommend selective imaging in patients with pain localized to the left lower quadrant, absence of vomiting, a CRP > 50mg/L, and/or a prior history of acute diverticulitis.**

**GRADE: Moderate-quality of evidence, Weak recommendation**

Comment

Acute colonic diverticulitis can be diagnosed by clinical examination in two-thirds of patients ^5,30^. Patients with pain solely in the left lower quadrant, absence of vomiting and CRP>50 mg/L are highly likely to have diverticulitis (specificity of 93-100%) ^5,30,31,33^. However, one small prospective study with 102 patients found that computed tomography (CT) scanning changed the suspected clinical stage of diverticulitis in 38% of the patients; 13% were clinically understaged and 25% overstaged ^48^. As a consequence, less patients were operated on ^48^. Toorenvliet et al. describe that in acute colonic diverticulitis cross-sectional imaging leads to change in diagnoses in 37% of the patients; on the other hand it leads to alteration of the initial management in only 7% ^32^. Neither of these studies, however, used the aforementioned decision rule. If patients meet all three criteria of the clinical decision rule, selective imaging may be considered. Patients who do not have all three features of the clinical decision rule and have persistent concern for diverticulitis should undergo imaging. Patients with severe symptoms or some concern for complicated acute diverticulitis (such as a CRP>100 mg/L) should also undergo imaging.

**When imaging is deemed necessary, the recommended modality of choice is CT scan. Alternatively, ultrasound at centers with expertise in that modality could be used.**

**GRADE: High-quality of evidence, strong recommendation**

Comment

In diagnosing, but not classifying, diverticulitis, both US and CT scan have been to found to be similarly efficacious. The pooled sensitivities of both modalities do not significantly differ in two meta-analyses (90% for US vs 95% for CT p=0.86 in one meta-analysis and 92% for US vs 94% for CT, p=0.65 in the other) ^30,49^. However, the specificity of CT was slightly higher (90% for US vs 96% for CT p=0.04 in one meta-analysis and 90% for US vs 99% for CT p=0.07 in the other) ^30,49^. Despite the modestly higher specificity with CT, proponents of ultrasound point to its lower costs, lack of exposure to radiation or contrast, and wide availability as a rationale for its initial use ^5,30,49,50^. Thus, authors of several systematic reviews and meta-analyses have suggested using an ultrasound first approach to diagnose acute colonic diverticulitis ^5,30,49,50^.

Others advocate CT should be the primary method of imaging for several reasons ^4,51^. Abdominal ultrasound is highly operator dependent, requiring a high level of expertise and is more difficult in obese patients ^4,50-52^. Ultrasound may be less sensitive in complicated diverticulitis. A prospective study of 175 patients found that the sensitivity of ultrasound in uncomplicated disease was 96%; however, 17 patients with complicated diverticulitis had no inflamed diverticulum on ultrasound and 6 ultrasound exams in these patients were falsely negative ^53^. Nielsen et al showed that ultrasound misdiagnosed only 17% of patients with uncomplicated diverticulitis but misdiagnosed 79% of patients with complicated disease ^54^. Ripolles et al showed in a study of 34 patients with diverticulitis that required emergent surgery, that 10 patients (29%) had a falsely negative ultrasound ^55^. Another study found that transrectcal ultrasound augmented the findings of transabdominal ultrasound in 43% of patients ^56^. Further, ultrasound has also not been extensively studied to classify the stage of diverticulitis.

CT in contrast is highly accurate in diagnosing complications of acute colonic diverticulitis and has substantial inter-observer agreement (median kappa value of 0.72) for classifying diverticulitis ^57-70^. CT also has an enhanced ability to diagnose alternative diagnoses ^3,4,30,49-51,71,72^. Additionally, the images from CT can be re-read at any time, and CT may be easier for pre-operative planning by surgeons ^3,49,61,64^. CT has been found to add value to the clinical diagnosis ^73^.

Contrast enema, or barium enema, should not be used in diagnosing acute diverticulitis because of lower sensitivity of 80%-83%, and specificity of 81%-100% compared to CT ^3-5,30,51,57,74,75^.

The role of MRI in diagnosing acute colonic diverticulitis is not clear. Some results are promising with sensitivities of 86-100% and specificities of 88-100% ^5,51,76-79^. Prolonged examination time in acutely ill patients and a lower sensitivity to identify free air are arguments against its routine use ^4^. Overall there is not enough available evidence to recommend MRI.

Both US and CT can be used in diagnosing acute colonic diverticulitis. However, outside of centers with expertise in ultrasound, we recommend CT scanning. If an ultrasound first strategy is pursued, patients with suspected complicated disease and/or in persistent clinical suspicion for diverticulitis after a negative ultrasound should also undergo a CT scan.

***Citations:***

1. Sallinen VJ, Leppaniemi AK, Mentula PJ. Staging of acute diverticulitis based on clinical, radiologic, and physiologic parameters. *The journal of trauma and acute care surgery.* 2015;78(3):543-551.

2. Pasternak I, Dietrich M, Woodman R, Metzger U, Wattchow DA, Zingg U. Use of severity classification systems in the surgical decision-making process in emergency laparotomy for perforated diverticulitis. *International journal of colorectal disease.* 2010;25(4):463-470.

3. Kruis W, Germer CT, Leifeld L. Diverticular disease: guidelines of the german society for gastroenterology, digestive and metabolic diseases and the german society for general and visceral surgery. *Digestion.* 2014;90(3):190-207.

4. Biondo S, Lopez Borao J, Millan M, Kreisler E, Jaurrieta E. Current status of the treatment of acute colonic diverticulitis: a systematic review. *Colorectal disease : the official journal of the Association of Coloproctology of Great Britain and Ireland.* 2012;14(1):e1-e11.

5. Andeweg CS, Mulder IM, Felt-Bersma RJ, et al. Guidelines of diagnostics and treatment of acute left-sided colonic diverticulitis. *Digestive surgery.* 2013;30(4):278-292.

6. Shafi S, Priest EL, Crandall ML, et al. Multicenter validation of American Association for the Surgery of Trauma grading system for acute colonic diverticulitis and its use for emergency general surgery quality improvement program. *The journal of trauma and acute care surgery.* 2016;80(3):405-410; discussion 410-401.

7. Ambrosetti P, Becker C, Terrier F. Colonic diverticulitis: impact of imaging on surgical management -- a prospective study of 542 patients. *European radiology.* 2002;12(5):1145-1149.

8. Constantinides VA, Tekkis PP, Senapati A. Comparison of POSSUM scoring systems and the surgical risk scale in patients undergoing surgery for complicated diverticular disease. *Diseases of the colon and rectum.* 2006;49(9):1322-1331.

9. Aydin HN, Tekkis PP, Remzi FH, Constantinides V, Fazio VW. Evaluation of the risk of a nonrestorative resection for the treatment of diverticular disease: the Cleveland Clinic diverticular disease propensity score. *Diseases of the colon and rectum.* 2006;49(5):629-639.

10. Boostrom SY, Wolff BG, Cima RR, Merchea A, Dozois EJ, Larson DW. Uncomplicated diverticulitis, more complicated than we thought. *Journal of gastrointestinal surgery : official journal of the Society for Surgery of the Alimentary Tract.* 2012;16(9):1744-1749.

11. Antolovic D, Reissfelder C, Koch M, et al. Surgical treatment of sigmoid diverticulitis--analysis of predictive risk factors for postoperative infections, surgical complications, and mortality. *International journal of colorectal disease.* 2009;24(5):577-584.

12. Bielecki K, Kaminski P, Klukowski M. Large bowel perforation: morbidity and mortality. *Techniques in coloproctology.* 2002;6(3):177-182.

13. Kaiser AM, Jiang JK, Lake JP, et al. The management of complicated diverticulitis and the role of computed tomography. *The American journal of gastroenterology.* 2005;100(4):910-917.

14. Murphy SF, Waters PS, Waldron RM, et al. Predictive factors for colonic resection in patients less than 49 years with symptomatic diverticular disease. *American journal of surgery.* 2016;212(1):47-52.

15. Occhionorelli S, Zese M, Tartarini D, et al. An approach to complicated diverticular disease. A retrospective study in an Acute Care Surgery service recently established. *Ann Ital Chir.* 2016;87:553-563.

16. Setti Carraro PG, Magenta A, Segala M, Ravizzini C, Nespoli A, Tiberio G. Predictive value of a pathophysiological score in the surgical treatment of perforated diverticular disease. *Chirurgia italiana.* 1999;51(1):31-36.

17. van de Wall BJ, Draaisma WA, Consten EC, van der Kaaij RT, Wiezer MJ, Broeders IA. Does the presence of abscesses in diverticular disease prelude surgery? *Journal of gastrointestinal surgery : official journal of the Society for Surgery of the Alimentary Tract.* 2013;17(3):540-547.

18. Vermeulen J, Gosselink MP, Hop WC, et al. Long-term survival after perforated diverticulitis. *Colorectal disease : the official journal of the Association of Coloproctology of Great Britain and Ireland.* 2011;13(2):203-209.

19. Gooszen AW, Tollenaar RA, Geelkerken RH, et al. Prospective study of primary anastomosis following sigmoid resection for suspected acute complicated diverticular disease. *The British journal of surgery.* 2001;88(5):693-697.

20. Pisanu A, Cois A, Uccheddu A. Surgical treatment of perforated diverticular disease: evaluation of factors predicting prognosis in the elderly. *International surgery.* 2004;89(1):35-38.

21. Pisanu A, Reccia I, Deplano D, Porru F, Uccheddu A. Factors predicting in-hospital mortality of patients with diffuse peritonitis from perforated colonic diverticulitis. *Ann Ital Chir.* 2012;83(4):319-324.

22. Thaler K, Neumann F, Gero A, Kreuzer W. Utility of appropriate peritonitis grading in the surgical management of perforated sigmoid diverticulitis. *Colorectal disease : the official journal of the Association of Coloproctology of Great Britain and Ireland.* 2000;2(6):359-363.

23. Wysocki A, Poźniczek M, Zub A. Selected prognostic factors in perforated left colon diverticula. *Pol Przegl Chir.* 2005;77(2):121-131.

24. Angenete E, Thornell A, Burcharth J, et al. Laparoscopic Lavage Is Feasible and Safe for the Treatment of Perforated Diverticulitis With Purulent Peritonitis: The First Results From the Randomized Controlled Trial DILALA. *Annals of Surgery.* 2016;263(1):117-122.

25. Galbraith N, Carter JV, Netz U, et al. Laparoscopic Lavage in the Management of Perforated Diverticulitis: a Contemporary Meta-analysis. *Journal of gastrointestinal surgery : official journal of the Society for Surgery of the Alimentary Tract.* 2017.

26. Lamb MN, Kaiser AM. Elective resection versus observation after nonoperative management of complicated diverticulitis with abscess: a systematic review and meta-analysis. *Diseases of the colon and rectum.* 2014;57(12):1430-1440.

27. Schultz JK, Yaqub S, Wallon C, et al. Laparoscopic Lavage vs Primary Resection for Acute Perforated Diverticulitis: The SCANDIV Randomized Clinical Trial. *Jama.* 2015;314(13):1364-1375.

28. Laurell H, Hansson LE, Gunnarsson U. Acute diverticulitis--clinical presentation and differential diagnostics. *Colorectal disease : the official journal of the Association of Coloproctology of Great Britain and Ireland.* 2007;9(6):496-501; discussion 501-492.

29. Jamal Talabani A, Endreseth BH, Lydersen S, Edna TH. Clinical diagnostic accuracy of acute colonic diverticulitis in patients admitted with acute abdominal pain, a receiver operating characteristic curve analysis. *International journal of colorectal disease.* 2017;32(1):41-47.

30. Andeweg CS, Wegdam JA, Groenewoud J, van der Wilt GJ, van Goor H, Bleichrodt RP. Toward an evidence-based step-up approach in diagnosing diverticulitis. *Scandinavian journal of gastroenterology.* 2014;49(7):775-784.

31. Lameris W, van Randen A, van Gulik TM, et al. A clinical decision rule to establish the diagnosis of acute diverticulitis at the emergency department. *Diseases of the colon and rectum.* 2010;53(6):896-904.

32. Toorenvliet BR, Bakker RF, Breslau PJ, Merkus JW, Hamming JF. Colonic diverticulitis: a prospective analysis of diagnostic accuracy and clinical decision-making. *Colorectal disease : the official journal of the Association of Coloproctology of Great Britain and Ireland.* 2010;12(3):179-186.

33. Kiewiet JJ, Andeweg CS, Laurell H, et al. External validation of two tools for the clinical diagnosis of acute diverticulitis without imaging. *Digestive and liver disease : official journal of the Italian Society of Gastroenterology and the Italian Association for the Study of the Liver.* 2014;46(2):119-124.

34. van de Wall BJ, Draaisma WA, van der Kaaij RT, Consten EC, Wiezer MJ, Broeders IA. The value of inflammation markers and body temperature in acute diverticulitis. *Colorectal disease : the official journal of the Association of Coloproctology of Great Britain and Ireland.* 2013;15(5):621-626.

35. Kaser SA, Furler R, Evequoz DC, Maurer CA. Hyponatremia is a specific marker of perforation in sigmoid diverticulitis or appendicitis in patients older than 50 years. *Gastroenterology research and practice.* 2013;2013:462891.

36. Evans J, Kozol R, Frederick W, et al. Does a 48-hour rule predict outcomes in patients with acute sigmoid diverticulitis? *Journal of gastrointestinal surgery : official journal of the Society for Surgery of the Alimentary Tract.* 2008;12(3):577-582.

37. Iyer R, Longstreth GF, Chu LH, et al. Acute colonic diverticulitis: diagnostic evidence, demographic and clinical features in three practice settings. *Journal of gastrointestinal and liver diseases : JGLD.* 2014;23(4):379-386.

38. Andeweg CS, Knobben L, Hendriks JC, Bleichrodt RP, van Goor H. How to diagnose acute left-sided colonic diverticulitis: proposal for a clinical scoring system. *Annals of Surgery.* 2011;253(5):940-946.

39. Kaser SA, Fankhauser G, Glauser PM, Toia D, Maurer CA. Diagnostic value of inflammation markers in predicting perforation in acute sigmoid diverticulitis. *World journal of surgery.* 2010;34(11):2717-2722.

40. Kechagias A, Rautio T, Kechagias G, Makela J. The role of C-reactive protein in the prediction of the clinical severity of acute diverticulitis. *The American surgeon.* 2014;80(4):391-395.

41. Nizri E, Spring S, Ben-Yehuda A, Khatib M, Klausner J, Greenberg R. C-reactive protein as a marker of complicated diverticulitis in patients on anti-inflammatory medications. *Techniques in coloproctology.* 2014;18(2):145-149.

42. Tursi A, Elisei W, Brandimarte G, Giorgetti GM, Aiello F. Predictive value of serologic markers of degree of histologic damage in acute uncomplicated colonic diverticulitis. *Journal of clinical gastroenterology.* 2010;44(10):702-706.

43. Jurowich CF, Jellouschek S, Adamus R, et al. How complicated is complicated diverticulitis?--phlegmonous diverticulitis revisited. *International journal of colorectal disease.* 2011;26(12):1609-1617.

44. Park NS, Jeen YT, Choi HS, et al. Risk factors for severe diverticulitis in computed tomography-confirmed acute diverticulitis in Korea. *Gut Liver.* 2013;7(4):443-449.

45. Tursi A, Brandimarte G, Giorgetti G, Elisei W, Maiorano M, Aiello F. The clinical picture of uncomplicated versus complicated diverticulitis of the colon. *Digestive diseases and sciences.* 2008;53(9):2474-2479.

46. Daniels L, Budding AE, de Korte N, et al. Fecal microbiome analysis as a diagnostic test for diverticulitis. *Eur J Clin Microbiol Infect Dis.* 2014;33(11):1927-1936.

47. Elsing C, Ernst S, Stremmel W. Value of lipopolysaccharide binding protein, interleukin-6 and C-reactive protein as biomarkers of severity in acute diverticulitis: a prospective study. *Clin Lab.* 2012;58(1):145-151.

48. Martin Arevalo J, Garcia-Granero E, Garcia Botello S, et al. [Early use of CT in the management of acute diverticulitis of the colon]. *Revista espanola de enfermedades digestivas : organo oficial de la Sociedad Espanola de Patologia Digestiva.* 2007;99(6):320-324.

49. Lameris W, Randen A, Bipat S, Bossuyt P, Boermeester M, Stoker J. Graded compression ultrasonography and computed tomography in acute colonic diverticulitis: meta-analysis of test accuracy (Structured abstract). *European radiology.* 2008;18(11):2498-2511.

50. Liljegren G, Chabok A, Wickbom M, Smedh K, Nilsson K. Acute colonic diverticulitis: a systematic review of diagnostic accuracy. *Colorectal disease : the official journal of the Association of Coloproctology of Great Britain and Ireland.* 2007;9(6):480-488.

51. Mcnamara MM, Lalani T, Camacho MA, et al. Left Lower Quadrant Pain — Suspected Diverticulitis. *American College of Radiology.* 2014;ACR Appropriateness Criteria:1-7.

52. van Randen A, Lameris W, van Es HW, et al. A comparison of the accuracy of ultrasound and computed tomography in common diagnoses causing acute abdominal pain. *European radiology.* 2011;21(7):1535-1545.

53. Hollerweger A, Macheiner P, Rettenbacher T, Brunner W, Gritzmann N. Colonic diverticulitis: diagnostic value and appearance of inflamed diverticula-sonographic evaluation. *European radiology.* 2001;11(10):1956-1963.

54. Nielsen K, Richir MC, Stolk TT, et al. The limited role of ultrasound in the diagnostic process of colonic diverticulitis. *World journal of surgery.* 2014;38(7):1814-1818.

55. Ripolles T, Agramunt M, Martinez MJ, Costa S, Gomez-Abril SA, Richart J. The role of ultrasound in the diagnosis, management and evolutive prognosis of acute left-sided colonic diverticulitis: a review of 208 patients. *European radiology.* 2003;13(12):2587-2595.

56. Hollerweger A, Rettenbacher T, Macheiner P, Brunner W, Gritzmann N. Sigmoid diverticulitis: value of transrectal sonography in addition to transabdominal sonography. *AJR American journal of roentgenology.* 2000;175(4):1155-1160.

57. Eggesbo HB, Jacobsen T, Kolmannskog F, Bay D, Nygaard K. Diagnosis of acute left-sided colonic diverticulitis by three radiological modalities. *Acta Radiol.* 1998;39(3):315-321.

58. Lohrmann C, Ghanem N, Pache G, Makowiec F, Kotter E, Langer M. CT in acute perforated sigmoid diverticulitis. *Eur J Radiol.* 2005;56(1):78-83.

59. Ritz JP, Lehmann KS, Loddenkemper C, Frericks B, Buhr HJ, Holmer C. Preoperative CT staging in sigmoid diverticulitis--does it correlate with intraoperative and histological findings? *Langenbeck's archives of surgery / Deutsche Gesellschaft fur Chirurgie.* 2010;395(8):1009-1015.

60. Unlu C, Beenen LF, Fauquenot JM, et al. Inter-observer reliability of computed tomographic classifications of diverticulitis. *Colorectal disease : the official journal of the Association of Coloproctology of Great Britain and Ireland.* 2014;16(6):O212-219.

61. Gielens MP, Mulder IM, van der Harst E, et al. Preoperative staging of perforated diverticulitis by computed tomography scanning. *Techniques in coloproctology.* 2012;16(5):363-368.

62. Rao PM, Rhea JT, Novelline RA, et al. Helical CT with only colonic contrast material for diagnosing diverticulitis: prospective evaluation of 150 patients. *AJR American journal of roentgenology.* 1998;170(6):1445-1449.

63. Lefevre F, Beot S, Chapuis F, et al. [Computed tomography study of the sigmoid colon: discriminating diagnostic criteria and interobserver correlations]. *J Radiol.* 1999;80(5):447-456.

64. Poletti PA, Platon A, Rutschmann O, et al. Acute left colonic diverticulitis: can CT findings be used to predict recurrence? *AJR American journal of roentgenology.* 2004;182(5):1159-1165.

65. Farag Soliman M, Wustner M, Sturm J, et al. [Primary diagnostics of acute diverticulitis of the sigmoid]. *Ultraschall in der Medizin (Stuttgart, Germany : 1980).* 2004;25(5):342-347.

66. García-Aguayo FJ, Gil PM. Acute diverticulitis of the sigmoid colon: Value of ultrasound as an initial diagnostic test. *Radiologia.* 2002;44(2):47-53.

67. Kircher MF, Rhea JT, Kihiczak D, Novelline RA. Frequency, sensitivity, and specificity of individual signs of diverticulitis on thin-section helical CT with colonic contrast material: experience with 312 cases. *AJR American journal of roentgenology.* 2002;178(6):1313-1318.

68. Werner A, Diehl SJ, Farag-Soliman M, Duber C. Multi-slice spiral CT in routine diagnosis of suspected acute left-sided colonic diverticulitis: a prospective study of 120 patients. *European radiology.* 2003;13(12):2596-2603.

69. Tack D, Bohy P, Perlot I, et al. Suspected acute colon diverticulitis: imaging with low-dose unenhanced multi-detector row CT. *Radiology.* 2005;237(1):189-196.

70. Rao PM, Rhea JT. Colonic diverticulitis: evaluation of the arrowhead sign and the inflamed diverticulum for CT diagnosis. *Radiology.* 1998;209(3):775-779.

71. Ince AT, Baysal B, Kayar Y, et al. Comparison of tomographic and colonoscopic diagnoses in the presence of colonic wall thickening. *Int J Clin Exp Med.* 2014;7(11):4413-4419.

72. Shen SH, Chen JD, Tiu CM, et al. Differentiating colonic diverticulitis from colon cancer: the value of computed tomography in the emergency setting. *J Chin Med Assoc.* 2005;68(9):411-418.

73. Brengman ML, Otchy DP. Timing of computed tomography in acute diverticulitis. *Diseases of the colon and rectum.* 1998;41(8):1023-1028.

74. Ambrosetti P, Jenny A, Becker C, Terrier TF, Morel P. Acute left colonic diverticulitis--compared performance of computed tomography and water-soluble contrast enema: prospective evaluation of 420 patients. *Diseases of the colon and rectum.* 2000;43(10):1363-1367.

75. Hansen O, Graupe F, Stock W. [Diagnosis of diverticulitis in routine practice: progress due to pelvic CT?]. *Langenbecks Arch Chir Suppl Kongressbd.* 1998;115:170-173.

76. Ajaj W, Ruehm SG, Lauenstein T, et al. Dark-lumen magnetic resonance colonography in patients with suspected sigmoid diverticulitis: a feasibility study. *European radiology.* 2005;15(11):2316-2322.

77. Heverhagen JT, Sitter H, Zielke A, Klose KJ. Prospective evaluation of the value of magnetic resonance imaging in suspected acute sigmoid diverticulitis. *Diseases of the colon and rectum.* 2008;51(12):1810-1815.

78. Oistamo E, Hjern F, Blomqvist L, Von Heijne A, Abraham-Nordling M. Cancer and diverticulitis of the sigmoid colon. Differentiation with computed tomography versus magnetic resonance imaging: preliminary experiences. *Acta Radiol.* 2013;54(3):237-241.

79. Heverhagen JT, Ishaque N, Zielke A, et al. Feasibility of MRI in the diagnosis of acute diverticulitis: initial results. *Magma.* 2001;12(1):4-9.
