# Supplementary material for: EAES and SAGES 2018 consensus conference on acute diverticulitis management: evidence-based recommendations for clinical practice
Source: Surg Endosc. 2019 Jun 27;33(9):2726–41. doi: 10.1007/s00464-019-06882-z (PMC6684540; doi:10.1007/s00464-019-06882-z)
Supplement: Supplementary file 5 — Supplementary material 5 (DOC 71 kb) [file 464_2019_6882_MOESM5_ESM.doc]

**SAGES / EAES Diverticulitis Consensus Conference**

**Team 3**

**Non-Resection Management of Uncomplicated Diverticulitis**

Residents: Richard Garfinkle (SAGES), Marguerite Gorter (EAES)

Experts: Marylise Boutros (SAGES), Tan Arulampalam (EAES)

**3.1 What are the risk factors for developing recurrent or ongoing diverticulitis among patients with uncomplicated acute diverticulitis?**

Statement

In patients successfully treated for uncomplicated acute diverticulitis, the most commonly reported risk factors for the development of recurrent diverticulitis are young age (<50) and previous history.

LOE: Low

Recommendation

We recommend that patients with risk factors for recurrent diverticulitis be counseled regarding an elevated risk for future episodes and the possible long-term treatment implications.

LOE: Low

SOR: Strong for using

Many studies have attempted to identify risk factors for the development of recurrent diverticulitis, with younger age and previous history of diverticulitis being the most commonly reported. Broderick-villa and colleagues retrospectively reviewed the Southern California Kaiser Permanente Discharge Abstract Database and identified 3,165 patients admitted with acute diverticulitis.^1^ Over a mean follow-up of 8.9 years, 222 (9.4%) patients developed a recurrence. On multivariate analysis, only age (≥50 years-old, HR 0.75, 0.59-0.95) and Charlson Comorbidity Index score of 1 (vs. 0, HR 1.45, 1.12-1.89) remained significantly associated with recurrence. Furthermore, a first recurrent episode was the only factor predictive of future recurrences. Binda and colleagues reported on a large, multicenter cohort of patients from 17 Italian centres with a mean follow-up of 10.7 years.^2^ Among 320 eligible patients, age <40 years-old (HR 5.01, 1.25-20.08) and ≥3 prior episodes of diverticulitis (HR 3.90, 1.69-9.03) were predictive of recurrence. A recent comprehensive systematic review evaluating risk factors for recurrent diverticulitis also identified age and previous diverticulitis recurrence as the only variables of high likelihood to predict future recurrence, based both on the strength of association and the number of studies supporting the association.^3^ Several other risk factors have been suggested, such as female gender^4-7^, family history^8^, immunosuppression^6,9^, and inflammatory extension and location within the colon^8,10^, but either inconsistently or in very few studies. It is important to note that most studies with recurrent diverticulitis as a primary outcome, particularly those with the longest follow-up times, are retrospective in nature.

References

1. Broderick-Villa G, Burchette RJ, Collins JC, Abbas MA, Haigh PI. Hospitalization for acute diverticulitis does not mandate routine elective colectomy. Arch Surg. 2005;140:576-81.
2. Binda GA, Arezzo A, Serventi A; Italian Study Group on Complicated Diverticulosis. Multicenre observational study on the natural history of left-sided acute diverticulitis. Br J Surg. 2012;99:276-85.
3. Hupfeld L, Burcharth J, Pammergaard HC, Rosenberg J. Risk factors for recurrence after acute colonic diverticulitis: a systematic review. Int J Colorectal Dis. 2017;32:611-22.
4. Li D, de Mestral C, Baxter NN, et al. Risk of readmission and emergency surgery following nonoperative management of colonic diverticulitis: a population-based analysis. Ann Surg. 2014;260:423-30.
5. Ho VP, Nash GM, Milsom JW, Lee SW. Identification of diverticulitis patients at high risk for recurrence and poor outcomes. J Trauma Acute Care Surg. 2015;78:112-9.
6. Rose J, Parina RP, Faiz O, Chang DC, Talamini MA. Long-term outcomes after initial presentation of diverticulitis. Ann Surg. 2015;262:1046-53.
7. Garfinkle R, Kugler A, Pelsser V, et al. Diverticular abscess managed with long-term definitive nonoperative intent is safe. Dis Colon Rectum. 2016;59:648-55.
8. Hall JF, Roberts PL, Ricciardi R, et al. Long-term follow-up after an initial episode of diverticulitis: what are the predictors of recurrence? Dis Colon Rectum. 2011;54:283-8.
9. Sallinen V, Mali J, Leppaniemi A, Mentula P. Assessment of risk for recurrent diverticulitis: a proposal of risk score for complicated recurrence. Medicine (Baltimore). 2015;94:e557.
10. Park SM, Kwon TS, Kim DJ, et al. Prediction and management of recurrent right colon diverticulitis. Int J Colorectal Dis. 2014;29:1255-60.

**3.1 What are the risk factors for developing recurrent or ongoing diverticulitis among patients with uncomplicated acute diverticulitis?**

Statement

There is no consensus on the definition of, or risk factors for, ongoing diverticulitis. As such, no recommendation can be made with regards to risk factors for ongoing diverticulitis

LOE: Very low

The definition of ongoing diverticulitis in the surgical literature remains ill-defined, and presents a potential source of confusion and disagreement among researchers. By definition, recurrent diverticulitis can be distinguished from ongoing disease by the presence of a symptom and inflammation-free period between disease presentations. However, in the research setting, this definition is often difficult to capture, and authors instead rely on an arbitrary amount of time between disease presentations to differentiate the two terms. Two letters to the editor have been published on this subject, with one proposing a 90-day disease-free interval^1^ and the other a 60-day disease-free interval^2^ to make a diagnosis of recurrent diverticulitis. We observed great variation in the definitions used to distinguish ongoing from recurrent diverticulitis in the studies identified in our search, with 12 using a 30-day cut-off,^3-14^ 5 using a 60-day cut-off,^15-19^ and 2 using a 90-day cut-off.^20,21^ Furthermore, many other studies failed to include time in their definition of diverticulitis recurrence altogether.^22-27^ For future studies evaluating risk factors for either ongoing or recurrent diverticulitis, a consensus definition is likely required to allow for their proper comparison.

References

1. Gervaz P, Ambrosetti P. Time for a (re) definition of (recurrent) sigmoid diverticulitis? Ann Surg. 2011;254:1076-7.
2. Garfinkle R, Boutros M. Recurrent versus persistent diverticulitis: an important distinction. Dis Colon Rectum. 2016;59:648-55.
3. Unlu C, Gunadi PM, Gerhards MF, Boermeester MA, Vrouenraets BC. Outpatient treatment for acute uncomplicated diverticulitis. Eur J Gastroenterol Hepatol. 2013;25:1038-43.
4. Isacson D, Andreasson K, Nikberg M, Smedh K, Chabok A. No antibiotics in acute uncomplicated diverticulitis: does it work? Scand J Gastroenterol. 2014;49:1441-6.
5. Isacson D, Thorisson A, Andreasson K, Nikberg M, Smedh K, Chabok A. Outpatient, non-antibiotic management in acute uncomplicated diverticulitis: a prospective study. Int J Colorectal Dis. 2015;30:1229-34.
6. Hall JF, Roberts PL, Ricciardi R, et al. Long-term follow-up after an initial episode of diverticulitis: what are the predictors of recurrence? Dis Colon Rectum. 2011;54:283-8.
7. Li D, de Mestral C, Baxter NN, et al. Risk of readmission and emergency surgery following nonoperative management of colonic diverticulitis: a population-based analysis. Ann Surg. 2014;260:423-30.
8. Mali JP, Mentula PJ, Leppaniemi AK, Sallinen VJ. Symptomatic treatment for uncomplicated acute diverticulitis: a prospective cohort study. Dis Colon Rectum. 2016;59:529-34.
9. Brochmann ND, Schultz JK, Jacobsen GS, Oresland T. Management of acute uncomplicated diverticulitis without antibiotics: a single-center cohort study. Colorectal Dis. 2016;18:1101-07.
10. Chabok A, Andreasson K, Nikberg M. Low risk of complications in patients with first-time acute uncomplicated diverticulitis. Int J Colorectal Dis. 2017;32:1699-1702.
11. Sirany AE, Gaertner WB, Madoff RD, Kwaan MR. Diverticulitis diagnosed in the emergency room: is it safe to discharge home? J Am Coll Surg. 2017;225:21-25.
12. Joliat GR, Emery J, Demartines N, Hubner M, Yersin B, Hahnloser D. Antibiotic treatment for uncomplicated and mild complicated diverticulitis: outpatient treatment for everyone. Int J Colorectal Dis. 2017;32:1313-1319.
13. Poletti PA, Platon A, Rutschmann O, et al. Acute left colonic diverticulitis: can CT findings be used to predict recurrence? Am J Roentgenol. 2004;182:1159-65.
14. Schug-Pass C, Geers P, Hugel O, Lippert H, Kockerling F. Prospective randomized trial comparing short-term antibiotic therapy versus standard therapy for acute uncomplicated sigmoid diverticulitis. Int J Colorectal Dis. 2010;25:751-9.
15. Binda GA, Arezzo A, Serventi A; Italian Study Group on Complicated Diverticulosis. Multicenre observational study on the natural history of left-sided acute diverticulitis. Br J Surg. 2012;99:276-85.
16. de Korte N, Kuyvenhoven JP, van der Peet DL, Felt-Bersma RJ, Cuesta MA, Stockmann HB. Mild colonic diverticulitis can be treated without antibiotics. A case-control study. Colorectal Dis. 2012;14:325-30.
17. Biondo S, Golda T, Kreisler E, et al. Outpatient versus hospitalization management of uncomplicated diverticulitis: a prospective, multicentre randomized clinical trial (DIVER trial). Ann Surg. 2014;259:38-44.
18. Trenti L, Kreisler E, Galvez A, Golda T, Frago R, Biondo S. Long-term evolution of acute colonic diverticulitis after successful medical treatment. World J Surg. 2015;39:266-74.
19. Unlu C, van de Wall BJ, Gerhards MF, et al. Influence of age on clinical outcomes of acute diverticulitis. J Gastrointest Surg. 2013;17:1651-6.
20. Scarpa CR, Buchs NC, Poncet A, et al. Short-term intravenous antibiotic treatment in uncomplicated diverticulitis does not increase the risk of recurrence compared to long-term treatment. Ann Coloproctol. 2015;31:52-6.
21. Stam MA, Draaisma WA, van de Wall BJ, Bolkenstein HE, Consten EC, Broeders IA. An unrestricted diet for uncomplicated diverticulitis is safe: results of a prospective diverticulitis diet study. Colorectal Dis. 2017;19:372-77.
22. Ho VP, Nash GM, Milsom JW, Lee SW. Identification of diverticulitis patients at high risk for recurrence and poor outcomes. J Trauma Acute Care Surg. 2015;78:112-9.
23. Broderick-Villa G, Burchette RJ, Collins JC, Abbas MA, Haigh PI. Hospitalization for acute diverticulitis does not mandate routine elective colectomy. Arch Surg. 2005;140:576-81.
24. Chabok A, Pahlman L, Hjern F, et al.; AVOD Study Group. Randomized clinical trial of antibiotics in acute uncomplicated diverticulitis. Br J Surg. 2012;99:532-9.
25. Daniels L, Unlu C, de Korte A, et al.; Dutch Diverticular Disease 3D Collaborative Study Group. Randomized clinical trial of observational versus antibiotic treatment for a first episodes of CT-proven uncomplicated acute diverticulitis. Br J Surg. 2017;104:52-61.
26. Biondo S, Borao JL, Kresiler E, et al. Recurrence and virulence of colonic diverticulitis in immunocompromised patients. Am J Surg. 2012;204:172-9.
27. Frileux P, Dubrez J, Burdy G, et al. Sigmoid diverticulitis. Longitudinal analysis of 222 patients with a minimal follow up of 5 years. Colorectal Dis. 2010;12:674-80.

**3.2 What is the optimum non-operative measures of uncomplicated acute diverticulitis?**

Statement

In immunocompetent individuals presenting with uncomplicated acute diverticulitis, symptomatic treatment without antibiotics provides similar outcomes to treatment with antibiotics.

LOE: High

Recommendation

In selected immunocompetent individuals presenting with uncomplicated acute diverticulitis, we recommend that a trial of non-antibiotic therapy be considered with appropriate follow-up.

LOE: High

SOR: Weak for using

Two randomized controlled trials have evaluated the use of antibiotic therapy in the treatment of uncomplicated acute diverticulitis. The AVOD trial randomized over 600 patients with CT-confirmed uncomplicated acute diverticulitis to either antibiotic or non-antibiotic therapy, and demonstrated equivalent rates of complications (abscess and/or perforation) in both groups (<2%).^1^ Only 3.2% of patients initially allocated to non-antibiotic therapy were ultimately started on antibiotics, and none of these patients suffered from complications. The DIABOLO trial, published five years later in 2017, similarly randomized over 500 patients, and reported an equivalent time to full recovery with and without antibiotics (12 vs. 14 days).^2^ There were no observed differences in secondary outcomes either, including complicated diverticulitis (2.6% vs. 3.8%), ongoing diverticulitis (4.1% vs. 7.3%), or recurrence and surgery at 1 year. Cases of uncomplicated diverticulitis with microperforation were not included in either trial, and while DIABOLO included few patients (<10%) with modified Hinchey 1B disease, the authors caution against using non-antibiotic therapy in such patients until larger cohorts are studied. In a follow-up study of DIABOLO, fluid collections on CT-scan were predictive of complications in the early period.^3^ Also noteworthy is the fact that AVOD did not limit enrollment to patients presenting with a first episode of diverticulitis, with roughly 40% of patients having a previous history. This is supported by recent data reporting no differences in the development of complications among patients with either a first episode or recurrent episode of uncomplicated acute diverticulitis.^4^ Outside of the two trials, several prospective^5-7^ and retrospective^8-10^ observational studies have demonstrated similar safety and rates of treatment success without antibiotics; however, all of the identified studies originate from European countries, which likely contributes to the slower adoption of such an approach in North American patients.

References

1. Chabok A, Pahlman L, Hjern F, et al.; AVOD Study Group. Randomized clinical trial of antibiotics in acute uncomplicated diverticulitis. Br J Surg. 2012;99:532-9.
2. Daniels L, Unlu C, de Korte A, et al.; Dutch Diverticular Disease 3D Collaborative Study Group. Randomized clinical trial of observational versus antibiotic treatment for a first episodes of CT-proven uncomplicated acute diverticulitis. Br J Surg. 2017;104:52-61.
3. van Dijk ST, Daniels L, Nio CY, Somers I, van Geloven AAW, Boermeester MA. Predictive factors on CT imaging for progression of uncomplicated into complicated acute diverticulitis. Int J Colorectal Dis. 2017;32:1693-98.
4. Chabok A, Andreasson K, Nikberg M. Low risk of complications in patients with first-time acute uncomplicated diverticulitis. Int J Colorectal Dis. 2017;32:1699-1702.
5. Isacson D, Andreasson K, Nikberg M, Smedh K, Chabok A. No antibiotics in acute uncomplicated diverticulitis: does it work? Scand J Gastroenterol. 2014;49:1441-6.
6. Mali JP, Mentula PJ, Leppaniemi AK, Sallinen VJ. Symptomatic treatment for uncomplicated acute diverticulitis: a prospective cohort study. Dis Colon Rectum. 2016;59:529-34.
7. Brochmann ND, Schultz JK, Jacobsen GS, Oresland T. Management of acute uncomplicated diverticulitis without antibiotics: a single-center cohort study. Colorectal Dis. 2016;18:1101-07.
8. de Korte N, Kuyvenhoven JP, van der Peet DL, Felt-Bersma RJ, Cuesta MA, Stockmann HB. Mild colonic diverticulitis can be treated without antibiotics. A case-control study. Colorectal Dis. 2012;14:325-30.
9. Unlu C, Gunadi PM, Gerhards MF, Boermeester MA, Vrouenraets BC. Outpatient treatment for acute uncomplicated diverticulitis. Eur J Gastroenterol Hepatol. 2013;25:1038-43.
10. Hjern F, Josephson T, Altman D, et al. Conservative treatment of acute colonic diverticulitis: are antibiotics always mandatory? Scand J Gastroenterol. 2007;42:41-7.

**3.2 What is the optimum non-operative measures of uncomplicated acute diverticulitis?**

Recommendation

We recommend that immunocompetent individuals presenting with uncomplicated acute diverticulitis and mild symptoms may be managed in the outpatient setting.

LOE: Moderate

SOR: Weak for using

The DIVER trial is the only randomized controlled trial evaluating the safety of outpatient management for patients with uncomplicated acute diverticulitis.^1^ One-hundred and thirty-two patients were randomized to admission or outpatient therapy, and all received antibiotic therapy. There was no difference in readmission due to failure of medical treatment between the two groups (4.5% vs. 6.1%). Patients assigned to outpatient care were contacted daily for 5 days after presentation and only patients who demonstrated improvement of pain and fever while in the emergency room were eligible for randomization, both of which raise some concerns with regards to the widespread applicability of outpatient management. It is imperative that a system be in place to follow such patients for the possible development of treatment failure and diverticulitis complications. Two prospective studies have also evaluated outpatient therapy with similar results. Mali et al. followed 161 patients, 140 of whom were treated as outpatients without antibiotics.^2^ Only four patients ultimately required admission, none of whom suffered a complication. Isacson et al. treated 155 patients as outpatients, also without antibiotics, and reported a 2.6% treatment failure.^3^ Sirany et al. retrospectively reviewed their experience with emergency room discharge or admission, and found equivalent 30-day readmissions and emergency room visits in both groups of patients (12.5% vs. 15.0%).^4^ Interestingly, 50% of patients discharged from the emergency room with microperforation on CT-scan were readmitted, perhaps indicating a subgroup unfit for outpatient management. Altogether, the evidence points to the safety of outpatient management in reliable patients with mild symptoms. It is important to recognize that there is less evidence supporting the application of both outpatient management and non-antibiotic therapy together, and future studies should aim to address these two measures.

References

1. Biondo S, Golda T, Kreisler E, et al. Outpatient versus hospitalization management of uncomplicated diverticulitis: a prospective, multicentre randomized clinical trial (DIVER trial). Ann Surg. 2014;259:38-44.
2. Mali JP, Mentula PJ, Leppaniemi AK, Sallinen VJ. Symptomatic treatment for uncomplicated acute diverticulitis: a prospective cohort study. Dis Colon Rectum. 2016;59:529-34.
3. Isacson D, Thorisson A, Andreasson K, Nikberg M, Smedh K, Chabok A. Outpatient, non-antibiotic management in acute uncomplicated diverticulitis: a prospective study. Int J Colorectal Dis. 2015;30:1229-34.
4. Sirany AE, Gaertner WB, Madoff RD, Kwaan MR. Diverticulitis diagnosed in the emergency room: is it safe to discharge home? J Am Coll Surg. 2017;225:21-25.

**3.3 What is the optimum follow-up following uncomplicated acute diverticulitis?**

Statement

The use of 5-ASA agents does not prevent recurrent diverticulitis or improve chronic gastrointestinal symptoms after successful treatment for an episode of uncomplicated acute diverticulitis. The role of rifaximin, probiotics, and fiber is less well defined.

LOE: High

Recommendation

We do not recommend the use of 5-ASA to prevent recurrent diverticulitis or improve chronic gastrointestinal symptoms among patients successfully treated for an episode of uncomplicated acute diverticulitis.

LOE: High

SOR: Strong against using

The use of 5-ASA agents to prevent recurrent diverticulitis has been well studied in multiple placebo-controlled, double-blind, randomized controlled trials (RCTs). The DIVA trial, PREVENT-1 and PREVENT-2 all failed to show a reduction in recurrence at 52 weeks and 104 weeks, respectively, with mesalamine.^1-2^ There were also no differences in health-related quality of life outcomes over the follow-up period, including global symptom scores. Several other RCTs have evaluated the role of mesalamine after an episode of acute diverticulitis but with high risk of bias.^3-6^ A 2017 Cochrane Review summarized the available data, and concluded that 5-ASA agents were not superior to control interventions for the prevention of recurrent diverticulitis.^7^ The most recent RCT not included in the Cochrane Review supports such a conclusion, also demonstrating no difference in recurrence-free survival at 48 and 96 weeks.^8^ With regards to other medical therapies to prevent diverticulitis recurrence, the evidence is far inferior for or against their use. In one underpowered, proof-of-concept RCT, the use of rifaximin and probiotics was superior to probiotics alone.^9^ In other observational series, rifaximin has been compared to mesalamine with conflicting results.^10,11^ A small RCT published in 2004 randomized 83 patients to probiotics or nothing, and reported less recurrence in the probiotics group (4.6% vs. 12.5%); however, CT-scans were not used for incident or recurrent diagnosis, and the trial was likely underpowered.^12^ The recommendation for fiber in patients with diverticulitis has long been part of surgical dogma, but is lacking in evidence. Two systematic reviews have attempted to assess the evidence for fibre in patients treated for diverticulitis but were unable to provide any conclusions.^13,14^ Identified studies in both reviews were evaluating fiber in patients with symptomatic uncomplicated diverticular disease, and thus were not measuring diverticulitis recurrence.

References

1. Stollman N, Magowan S, Shanahan F, et al.; DIVA Investigator Group. A randomized controlled study of mesalamine after acute diverticulitis: results of the DIVA trial. J Clin Gastroenterol. 2013;47:621-9.
2. Raskin JB, Kamm MA, Jamal MM, et al. Mesalamine did not prevent recurrent diverticulitis in phase 3 controlled trials. Gastroenterology. 2014;127:793-802.
3. Parente F, Bargiggia S, Prada A, et al.; Gismi Study Group. Intermittent treatment with mesalazine in the prevention of diverticulitis recurrence: a randomized multicentre pilot double-blind placebo-controlled study of 24-month duration. Int J Colorectal Dis. 2013;28:1423-31.
4. Tursi A, Brandimarte G, Daffina R. Long-term treatment with mesalazine and rifaximin versus rifaximin alone for patients with recurrent attacks of acute diverticulitis of colon. Dig Liver Dis. 2002;34:510-5.
5. Tursi A, Brandimarte G, Giorgetti GM, Elisei W, Aiello F. Balsalazide and/or high-potency probiotic mixture (VSL#3) in maintaining remission after attack of acute, uncomplicated diverticulitis of the colon. Int J Colorectal Dis. 2007;22:1103-8.
6. Trepsi E, Colla C, Panizza P, et al. [Therapeutic and prophylactic role of mesalazine (5-ASA) in symptomatic diverticular disease of the large intestine. 4 year follow-up results]. Minerva Gastroenterol Dietol. 1999;45:245-52.
7. Carter F, Alsayb M, Marshall JK, Yuan Y. Mesalamine (5-ASA) for the prevention of recurrent diverticulitis. Cochrane Database Syst Rev. 2017 Oct 3;10:CD009839.
8. Kruis W, Kardalinos V, Eisenbach T, et al. Randomized clinical trial: mesalazine versus placebo in the prevention of diverticulitis recurrence. Aliment Pharmacol Ther. 2017;46:282-91.
9. Lanas A, Ponce J, Bignamini A, Mearin F. One year intermittent rifaximin plus fibre supplementation vs. fibre supplementation alone to prevent diverticulitis recurrence: proof-of-concept study. Dig Liver Dis. 2013;45:104-9.
10. Tursi A, Elisei W, Giorgetti GM, et al. Effectiveness of different therapeutic strategies in preventing diverticulitis recurrence. Eur Rev Med Pharmacol Sci. 2013;17:342-8.
11. Festa V, Spila Alegiani S, Chiesara F, et al. Retrospective comparison of long-term ten-day/month rifaximin or mesalazine in prevention of relapse in acute diverticulitis. Eur Rev Med Pharmacol Sci. 2017;21:1397-1404.
12. Dughera L, Serra AM, Battaglia E, Tibaudi D, Navino M, Emanuelli G. Acute recurrent diverticulitis is prevented by oral administration of a polybacterial lysate suspension. Minerva Gastroenterol Dietol. 2004;50:149-53.
13. Maconi G, Barbara G, Bosetti C, Cuomo R, Annibale B. Treatment of diverticular disease of the colon and prevention of acute diverticulitis: a systematic review. Dis Colon Rectum. 2011;54:1326-38.
14. Unlu C, Daniels L, Vrouenraets BC, Boermeester MA. A systematic review of high-fibre dietary therapy in diverticular disease. Int J Colorectal Dis. 2012;27:419-27.

**3.4 What is the role of ~~of~~ interval endoscopy following an acute episode of diverticulitis?**

Statement

In the absence of high-risk features, the detection rate for advanced adenomas or malignant lesions with colonic evaluation after an episode of uncomplicated acute diverticulitis is very low.

LOE: Moderate

Recommendation

Among patients treated successfully for uncomplicated acute diverticulitis, we do not recommend routine colonic evaluation unless high-risk features are present.

LOE: Moderate

SOR: Weak for using

Routine colonic evaluation after an episode of acute diverticulitis is recommended by most surgical authorities. However, among patients successfully treated for uncomplicated acute diverticulitis, the detection rate for advanced lesions is very low. The AVOD trial, which evaluated the use of antibiotics in the treatment of uncomplicated diverticulitis, reported a 0% detection rate for cancer among 545 patients who received a follow-up colonoscopy 6-8 weeks after their disease presentation.^1^ Brar et al. similarly demonstrated no cancers among 185 patients who underwent colonoscopy, all of whom had not received any form of lower endoscopy within the 2 years prior to disease presentation.^2^ A systematic review and meta-analysis attempted to aggregate all of the available data, and reported a pooled estimate of 0.7% (95% CI 0.3%-1.4%) for cancer detection among 1,497 patients with uncomplicated diverticulitis, comparable to the reported cancer rate of 0.78% among 68,324 asymptomatic patients undergoing screening colonoscopy.^3^ Based on this data, it would appear that routine colonoscopy after an episode of uncomplicated diverticulitis is not warranted. However, in patients with certain high-risk features, colonoscopy should still be performed. Among patients with perforation and an abscess, the cancer detection rate was estimated at 10.8% (95% CI 5.2%-21.0%) in the aforementioned systematic review.^3^ Brar et al. also reported 4 cancers among 74 patients with an abscess, and another 13.5% in whom an advanced lesion was found.^2^ In another prospective cohort of 155 diverticulitis patients, only 2 patients were found to have a cancer, but both had CT-scans that were reported as highly suspicious for malignancy.^4^ Furthermore, in a randomized controlled trial out of Israel evaluating early vs. late colonoscopy after diverticulitis, 4/23 patients with protracted symptoms (defined as persistent after 1 week of treatment) had positive findings.^5^ Therefore, while routine colonic evaluation after an episode of uncomplicated diverticulitis may be unwarranted, we would still recommend colonoscopy in patients with an associated perforation and/or abscess, suspicious findings on CT-scan, or ongoing symptoms.

References

1. Chabok A, Pahlman L, Hjern F, et al.; AVOD Study Group. Randomized clinical trial of antibiotics in acute uncomplicated diverticulitis. Br J Surg. 2012;99:532-9.
2. Brar MS, Roxin G, Yaffe PB, Stranger J, MacLean AB, Buie WD. Colonoscopy following nonoperative management of uncomplicated diverticulitis may not be warranted. Dis Colon Rectum. 2013;56:1259-64.
3. Sharma PV, Eglinton T, Hider P, Frizelle F. Systematic review and meta-analysis of the role of routine colonic evaluation after radiologically confirmed acute diverticulitis. Ann Surg. 2014;259:263-72.
4. Isacson D, Thorisson A, Andreasson K, Nikberg M, Smedh K, Chabok A. Outpatient, non-antibiotic management in acute uncomplicated diverticulitis: a prospective study. Int J Colorectal Dis. 2015;30:1229-34.
5. Lahat A, Yanai H, Sakhnini E, Menachem Y, Bar-Meir S. Role of colonoscopy in patients with persistent acute diverticulitis. World J Gastroenterol. 2008;14:2763-6.
