# Supplementary material for: EAES and SAGES 2018 consensus conference on acute diverticulitis management: evidence-based recommendations for clinical practice
Source: Surg Endosc. 2019 Jun 27;33(9):2726–41. doi: 10.1007/s00464-019-06882-z (PMC6684540; doi:10.1007/s00464-019-06882-z)
Supplement: Supplementary file 6 — Supplementary material 6 (DOCX 164 kb) [file 464_2019_6882_MOESM6_ESM.docx]

SAGES/EAES acute diverticulitis consensus – Topic 4

Non-resectional management of complicated acute diverticulitis

Nathan J Curtis (Department of General Surgery, Yeovil District Hospital NHS Foundation Trust, UK & Department of Surgery and Cancer, Imperial College London, UK)

Daniel A Hashimoto (Department of Surgery, Massachusetts General Hospital, Boston, MA, USA)

Alberto Arezzo (Department of Surgical Sciences, University of Torino, Torino, Italy)

Todd Francone (Division of Colon & Rectal Surgery, Newton-Wellesley Hospital, Newton, MA, USA)

Search strategy

632 abstracts were identified from the search string with 64 meeting final inclusion criteria and were reviewed to draft the following statements and recommendations.

Q4.1 What is the non-operative management of complicated AD?

Twenty-five papers covering 21,656 patients reported on the non-resectional management of diverticular abscesses (Hinchey Ib-II) and/or perforated diverticulitis (1-25)(GRADE - 8% High, 4% moderate, 40% low, 48% very low).

A systematic review of 8766 patients from 42 observational studies reported antibiotic therapy alone is successful in 81% of abscesses (12). An inverse size relationship was observed (antibiotic success 100% ≤3cm, 82% 3-10cm, 66% 3-18.5cm), although an association between abscess size and success rate of antibiotic only treatment has not been definitely established (12). We consider antibiotics to be first line therapy for acute diverticular abscesses. No studies investigated antibiotic duration specifically in diverticulitis. However, for patients who undergo an infectious source control procedure (e.g. percutaneous drainage), the STOP-IT trial demonstrated that a short course of antibiotics (4±1 days after source control or two days after resolution of physiological abnormalities) was equivalent to longer courses of antibiotics (26-28). No evidence on optimal antibiotic regime or route identified. Therefore, treatment should adhere to local guidelines and antibiotic stewardship principles.

After one-day of in-hospital observation and intravenous antibiotics, very high success rates have been reported in a series of patients with small abscesses managed in ambulatory care settings with oral antibiotics (18). Although there are limited reports of resolution without therapy (25), there is insufficient evidence to withhold antibiotics where an abscess or perforated disease has been diagnosed. Overall 1% mortality was observed in non-operatively treated patients (12).

*Statement: Antibiotic therapy alone is associated with a very high treatment success rate for abscesses (<4 cm). An association between size and success rate has been observed.*

*Recommendation: For all abscesses, we recommend antibiotics should be considered first line treatment.*

*(LoE 2a, Quality of Evidence: Moderate, Strength: Strong recommendation for antibiotics)*

*Statement: There is no evidence to support a particular antibiotic regime, route, or duration for complicated acute diverticulitis. Antibiotics are indicated in all complicated acute diverticulitis cases. If a drainage procedure is indicated, there is no evidence to support a prolonged course of antibiotics after source control is achieved.*

*Recommendation: We recommend antibiotic use covers gram-negative and anaerobic bacteria based on institutional protocols and antibiotic stewardship principles.*

*(LoE 4, Quality of Evidence: Very low, Strength: Strong recommendation for antibiotics covering gram-negative and anaerobic bacteria)*

For smaller abscesses, antibiotic therapy alone and percutaneous drainage have similar success rates, morbidity, and mortality (12). Although the use of percutaneous drainage is increasing (17, 29) and may allow a subset of patients to avoid emergent surgery (1, 5, 14, 17), no evidence exists to define when to recommend percutaneous drainage over antibiotic therapy. However, percutaneously drained abscesses >4 cm successfully resolved in 80% of patients (3).

Drain placement has a reported 2.5% complication rate (range 0-12.5%) consisting primarily of small bowel injury and/or fistulation, and 15% of drains required replacement or adjustment (range 0-42%) (12). A 15% recurrence rate has been reported following drainage, with abscesses >5cm presenting an increased risk (10, 14). We suggest a stepwise management strategy where drainage is added for accessible abscesses that fail to, or are not expected to, resolve with antibiotic therapy or in the unwell patient. No study investigating optimal drainage duration was found.

*Statement: Percutaneously drained abscesses >4 cm successfully resolved in 80% of patients with a low complication and re-intervention rate.*

*Recommendation: We recommend percutaneous drainage be considered for larger abscesses, those that do not resolve on antibiotics, and/or in the presence of patient deterioration.*

*(LoE 2b, Quality of evidence: low, Strength: Weak recommendation for percutaneous drainage)*

Where an acute diverticular episode is associated with extraluminal air, in observational studies, the majority of patients were successfully managed with non-operative measures (6, 21, 25). Where peri-colonic air alone is seen, 99% avoided further intervention, decreasing to 66-93% for distal air (6, 21). An associated abscess predicted the need for further treatment. There are limited reports of selected patients with small abscesses (18) or extra-luminal air (25) being successfully managed as outpatients but the role for this strategy is not defined and not recommended outside of research studies.

*Statement: The majority of stable patients with radiological evidence of extraluminal air and no extravasation of contrast can be successfully managed non-operatively. The presence of an associated abscess or distant air are predictors of failure of non-operative management.*

*Recommendation: In stable patients diagnosed with free air, we recommend initial non-operative management.*

*(LoE 3b, Quality of evidence: low, Strength: Weak recommendation for non-operative management)*

No included study investigated acute management of diverticular fistulae, the need for or timing of reimaging following diagnosis of a complicated episode, or specific criteria for either hospital discharge or selection for outpatient management.

Q4.2 What is the role of laparoscopic lavage in the management of diverticulitis?

Laparoscopic lavage has gained a high profile as a possible alternative to acute resection. 38 studies were identified (16, 22, 30-65), including seven reports from three randomized control trials (DILALA (32, 62), SCANDIV (56, 57) and the LOLA arm of the LADIES trial (63, 64, 66)) and two earlier reviews of observational studies (30, 39). Nearly all reports are based on Hinchey III cases. The RCTs have been meta-analysed six times (31, 34, 35, 40, 48, 52) (GRADE - 36% High, 6% moderate, 24% low, 34% very low). According to the Cochrane tool, the RCTs show low risk of bias with the exception that surgeon, patient, or assessor blinding was not possible.

The RCTs randomized a total of 372 with predominantly Hinchey III purulent peritonitis without a visible perforation to lavage (n=163) or resection (n=152). The LOLA study was halted by their data safety committee due to higher in-hospital mortality or major morbidity primarily consisting of surgical re-intervention (35 vs 18%, OR 2.74, p=0.12) in the lavage group, despite equal in-hospital mortality or major morbidity at 12 months (67 vs. 60%, p=0.58) (63). Nevertheless, meta-analyses of all three RCTs showed no differences in 12-month mortality, quality of life, or readmission. Lavage was associated with a higher rate of post-operative abscess formation and re-operation. The risk of reoperation was quantified as 9-10% higher, particularly after 30 days (40). Lavage was also associated with decreased stoma formation (14% vs. 90%) (52) or a 67% reduction (40), fewer wound infections, cardiac complications, and shorter operations and hospital length of stay.

When lavage is being considered, a personalized approach with careful patient communication is advocated. Presenting the risks and benefits versus definitive management may be challenging, especially explaining that re-operation after lavage is likely to leave the patient in a similar position to initial resectional surgery. Neither a standardized nor an optimal lavage technique has been researched, potentially influencing the reported results through intervention heterogeneity. Given the identified concerns, we suggest that lavage, when selected, be performed by experienced laparoscopic surgeons able to perform full lavage (including all intra-loop spaces) and have the ability to manage early post-operative complications. Surgeons should ensure careful patient monitoring is in place, including the ability to engage in prompt re-intervention when indicated.

Given the lack of reports and concerns above, visualized perforations or Hinchey IV disease should not be managed with laparoscopic lavage outside of research settings, and conversion to resection appears indicated when faecal peritonitis is unexpectedly identified on table. Additionally, as Hinchey I-II cases are likely to settle without operative intervention (12), laparoscopic lavage should be restricted to Hinchey III cases. There is no evidence to allow specific discharge or follow up criteria, including endoscopic assessment, to be recommended following lavage.

*Statement: Laparoscopic lavage has been shown to decrease stoma formation rate without impacting one-year mortality, although short term morbidity may be increased. There is no consensus on an effective laparoscopic lavage technique.*

*Recommendation: We recommend that lavage may be considered in selected Hinchey III patients by surgeons with appropriate expertise and the ability to closely watch for and manage complications. The lower stoma rate should be weighed against the higher risk of complications and re-intervention.*

*(LoE: 1a, Quality of evidence: High, Strength: Weak recommendation for lavage in select patients)*

Q4.3 When is surgical treatment indicated in acute complicated diverticulitis?

As discussed in Q4.1, Hinchey I-II abscesses or presence of peri-colonic air are likely to settle with non-operative management (12, 21). Therefore, acute surgery should be reserved for those patients who have exhausted these options without improvement of symptoms or remain systemically unwell. In purulent or faecal peritonitis secondary to diverticulitis, non-operative therapies are not advocated as they expose the patient to additional risk through delay in definitive source control.

*Statement: The majority of Hinchey Ib-II abscesses and presence of peri-colonic air can successfully be managed non-operatively.*

*Recommendation: In Hinchey Ib-II abscesses or presence of peri-colonic air cases, acute surgery should be reserved to patients who have exhausted non-operative options without improvement of symptoms or remain systemically unwell.*

*(LoE: 2a, Quality of evidence: Moderate, Strength: Strong against surgery)*

*Statement: Non-operative management of Hinchey III or IV disease has a low success rate.*

*Recommendation: When there is clinical and/or radiological suspicion of Hinchey III or IV diverticulitis, acute surgery should be considered.*

*(LoE: 3b, Quality of evidence: Low, Strength: Strong for surgery)*

After successful resolution of a complicated acute diverticulitis episode, in a series of 14,124 patients, 11% were admitted with recurrent diverticulitis in the subsequent four years (17). The highest risk of recurrence occurred early before plateauing (17). After percutaneous drainage, 58% had no further diverticular episode in the following seven years (10). When initial management was compared, 16% of percutaneously drained abscess recurred in the short term compared with 25% of those managed with antibiotics alone (12).

When a further episode does occur, the majority tend to be uncomplicated with low emergency surgery rates reported (3, 5, 17). In those that do recur, only 29% experienced a further subsequent recurrent episode (2). No studies investigating quality of life were identified, but surgery solely to avoid future diverticulitis episodes does not appear justified.

*Statement: The majority of patients with Hinchey Ib-II abscesses that are successfully managed non-operatively for a single episode of diverticulitis are unlikely to experience any further acute diverticulitis episode during long term follow up.*

*Recommendation: We recommend that following a single episode of successfully treated Hinchey I/II acute diverticulitis, surgery should not be routinely offered solely to avoid future episodes.*

*(LoE: 2b, Quality of evidence: Low, Strength: Weak against surgery)*

Q4.4 How should complicated diverticulitis be managed in specific patient groups?

Ten observational papers were reviewed to explore management of specific patient groups (7, 9, 17, 20, 22, 43, 54, 67-69) (GRADE – 60% low, 40% very low). No patient demographic was shown to influence presentation, severity or management of complicated acute diverticulitis. Immunosuppression (defined as malignancy, chronic kidney disease, transplantation or any immunosuppressive medication) was reported as a significant adverse risk factor (67, 68). Non-operative management of complicated diverticulitis was seen to be successful in 60% of patients in this challenging group; and when emergency resectional surgery was required, the observed mortality exceeded 30%. In a small subgroup where lavage was performed, it was unsuccessful for the majority of these patients (43). When non-operative care was successful, the majority developed a further complicated episode within six months with an associated high mortality rate.

*Statement: Immunosuppressed patients are a high-risk group for early, frequent and severe relapses after complicated acute diverticulitis managed non-operatively*

*Recommendation:* *In immunosuppressed patients with complicated diverticulitis, we recommend early elective resectional surgery.*

*(LoE: 3b, Quality of evidence: Very Low, Strength: Weak against non-operative management)*

In those presenting with acute diverticulitis, diabetic patients were seen to have a small but significantly higher rate of complicated diverticulitis than non-diabetics (Hinchey III+IV: 12% vs. 9%; although the success of non-operative management was not different (69). Longer antibiotic courses do not appear indicated in high risk patient groups (70). When acute surgery was performed, diabetic patients were more likely to experience surgical site infections (69).

*Statement: Diabetic patients presenting with acute diverticulitis have a higher incidence of complicated episodes but similar success with non-operative management compared to non-diabetic patients.*

*Recommendation: We recommend clinicians consider diabetes as a risk factor for complicated acute diverticulitis, but non-operative management remains appropriate.*

*(LoE: 2c, Quality of evidence: Low, Strength: Weak for non-operative management)*

References

1. Brandt D, Gervaz P, Durmishi Y, Platon A, Morel P, Poletti PA. Percutaneous CT scan-guided drainage vs. antibiotherapy alone for Hinchey II diverticulitis: a case-control study. Dis Colon Rectum. 2006;49(10):1533-8.

2. Broderick-Villa G, Burchette RJ, Collins JC, Abbas MA, Haigh PI. Hospitalization for acute diverticulitis does not mandate routine elective colectomy. Arch Surg. 2005;140(6):576-81; discussion 81-3.

3. Buchwald P, Dixon L, Wakeman CJ, Eglinton TW, Frizelle FA. Hinchey I and II diverticular abscesses: long-term outcome of conservative treatment. ANZ J Surg. 2016.

4. Costi R, Cauchy F, Le Bian A, Honart JF, Creuze N, Smadja C. Challenging a classic myth: pneumoperitoneum associated with acute diverticulitis is not an indication for open or laparoscopic emergency surgery in hemodynamically stable patients. A 10-year experience with a nonoperative treatment. Surg Endosc. 2012;26(7):2061-71.

5. Devaraj B, Liu W, Tatum J, Cologne K, Kaiser AM. Medically Treated Diverticular Abscess Associated With High Risk of Recurrence and Disease Complications. Dis Colon Rectum. 2016;59(3):208-15.

6. Dharmarajan S, Hunt SR, Birnbaum EH, Fleshman JW, Mutch MG. The efficacy of nonoperative management of acute complicated diverticulitis. Dis Colon Rectum. 2011;54(6):663-71.

7. Elagili F, Stocchi L, Ozuner G, Dietz DW, Kiran RP. Outcomes of percutaneous drainage without surgery for patients with diverticular abscess. Dis Colon Rectum. 2014;57(3):331-6.

8. Elagili F, Stocchi L, Ozuner G, Kiran RP. Antibiotics alone instead of percutaneous drainage as initial treatment of large diverticular abscess. Tech Coloproctol. 2015;19(2):97-103.

9. Felder SI, Barmparas G, Lynn J, Murrell Z, Margulies DR, Fleshner P. Can the need for colectomy after computed tomography-guided percutaneous drainage for diverticular abscess be predicted? Am Surg. 2013;79(10):1013-6.

10. Gaertner WB, Willis DJ, Madoff RD, Rothenberger DA, Kwaan MR, Belzer GE, et al. Percutaneous drainage of colonic diverticular abscess: is colon resection necessary? Dis Colon Rectum. 2013;56(5):622-6.

11. Garfinkle R, Kugler A, Pelsser V, Vasilevsky CA, Morin N, Gordon P, et al. Diverticular Abscess Managed With Long-term Definitive Nonoperative Intent Is Safe. Dis Colon Rectum. 2016;59(7):648-55.

12. Gregersen R, Mortensen LQ, Burcharth J, Pommergaard HC, Rosenberg J. Treatment of patients with acute colonic diverticulitis complicated by abscess formation: A systematic review. Int J Surg. 2016;35:201-8.

13. Hamy A, Paineau J. [Percutaneous drainage of perisigmoid abscesses of diverticular origin]. Ann Chir. 2001;126(2):133-7.

14. Kaiser AM, Jiang JK, Lake JP, Ault G, Artinyan A, Gonzalez-Ruiz C, et al. The management of complicated diverticulitis and the role of computed tomography. Am J Gastroenterol. 2005;100(4):910-7.

15. Lamb MN, Kaiser AM. Elective resection versus observation after nonoperative management of complicated diverticulitis with abscess: a systematic review and meta-analysis. Dis Colon Rectum. 2014;57(12):1430-40.

16. Li D, Baxter NN, McLeod RS, Moineddin R, Wilton AS, Nathens AB. Evolving practice patterns in the management of acute colonic diverticulitis: a population-based analysis. Dis Colon Rectum. 2014;57(12):1397-405.

17. Li D, de Mestral C, Baxter NN, McLeod RS, Moineddin R, Wilton AS, et al. Risk of readmission and emergency surgery following nonoperative management of colonic diverticulitis: a population-based analysis. Ann Surg. 2014;260(3):423-30; discussion 30-1.

18. Martin Gil J, Serralta De Colsa D, Garcia Marin A, Vaquero Rodriguez A, Rey Valcarcel C, Perez Diaz MD, et al. [Safety and efficiency of ambulatory treatment of acute diverticulitis]. Gastroenterol Hepatol. 2009;32(2):83-7.

19. Nelson RS, Ewing BM, Wengert TJ, Thorson AG. Clinical outcomes of complicated diverticulitis managed nonoperatively. Am J Surg. 2008;196(6):969-72; discussion 73-4.

20. Poletti PA, Platon A, Rutschmann O, Kinkel K, Nyikus V, Ghiorghiu S, et al. Acute left colonic diverticulitis: can CT findings be used to predict recurrence? AJR Am J Roentgenol. 2004;182(5):1159-65.

21. Sallinen VJ, Mentula PJ, Leppaniemi AK. Nonoperative management of perforated diverticulitis with extraluminal air is safe and effective in selected patients. Dis Colon Rectum. 2014;57(7):875-81.

22. Sartelli M, Binda GA, Brandara F, Borasi A, Feroci F, Vadala S, et al. IPOD Study: Management of Acute Left Colonic Diverticulitis in Italian Surgical Departments. World J Surg. 2017;41(3):851-9.

23. Singh B, May K, Coltart I, Moore NR, Cunningham C. The long-term results of percutaneous drainage of diverticular abscess. Ann R Coll Surg Engl. 2008;90(4):297-301.

24. Suarez Alecha J, Amoza Pais S, Batlle Marin X, Oronoz Martinez B, Balen Ribera E, Yarnoz Irazabal C. Safety of nonoperative management after acute diverticulitis. Ann Coloproctol. 2014;30(5):216-21.

25. Thorisson A, Smedh K, Torkzad MR, Pahlman L, Chabok A. CT imaging for prediction of complications and recurrence in acute uncomplicated diverticulitis. Int J Colorectal Dis. 2016;31(2):451-7.

26. Sawyer RG, Claridge JA, Nathens AB, Rotstein OD, Duane TM, Evans HL, et al. Trial of Short-Course Antimicrobial Therapy for Intraabdominal Infection. New England Journal of Medicine. 2015;372(21):1996-2005.

27. Rattan R, Allen CJ, Sawyer RG, Askari R, Banton KL, Coimbra R, et al. Percutaneously drained intra-abdominal infections do not require longer duration of antimicrobial therapy. J Trauma Acute Care Surg. 2016;81(1):108-13.

28. Rattan R, Namias N, Sawyer RG. Patients with Complicated Intra-Abdominal Infection Presenting with Sepsis Do Not Require Longer Duration of Antimicrobial Therapy: In reply to Spartalis and colleagues. J Am Coll Surg. 2016;223(1):206-7.

29. Salem L, Anaya DA, Flum DR. Temporal changes in the management of diverticulitis. J Surg Res. 2005;124(2):318-23.

30. Alamili M, Gogenur I, Rosenberg J. Acute complicated diverticulitis managed by laparoscopic lavage. Dis Colon Rectum. 2009;52(7):1345-9.

31. Angenete E, Bock D, Rosenberg J, Haglind E. Laparoscopic lavage is superior to colon resection for perforated purulent diverticulitis-a meta-analysis. Int J Colorectal Dis. 2017;32(2):163-9.

32. Angenete E, Thornell A, Burcharth J, Pommergaard HC, Skullman S, Bisgaard T, et al. Laparoscopic Lavage Is Feasible and Safe for the Treatment of Perforated Diverticulitis With Purulent Peritonitis: The First Results From the Randomized Controlled Trial DILALA. Ann Surg. 2016;263(1):117-22.

33. Catry J, Brouquet A, Peschaud F, Vychnevskaia K, Abdalla S, Malafosse R, et al. Sigmoid resection with primary anastomosis and ileostomy versus laparoscopic lavage in purulent peritonitis from perforated diverticulitis: outcome analysis in a prospective cohort of 40 consecutive patients. Int J Colorectal Dis. 2016;31(10):1693-9.

34. Ceresoli M, Coccolini F, Montori G, Catena F, Sartelli M, Ansaloni L. Laparoscopic lavage versus resection in perforated diverticulitis with purulent peritonitis: a meta-analysis of randomized controlled trials. World J Emerg Surg. 2016;11(1):42.

35. Cirocchi R, Di Saverio S, Weber DG, Tabola R, Abraha I, Randolph J, et al. Laparoscopic lavage versus surgical resection for acute diverticulitis with generalised peritonitis: a systematic review and meta-analysis. Tech Coloproctol. 2017;21(2):93-110.

36. Cirocchi R, Trastulli S, Vettoretto N, Milani D, Cavaliere D, Renzi C, et al. Laparoscopic peritoneal lavage: a definitive treatment for diverticular peritonitis or a "bridge" to elective laparoscopic sigmoidectomy?: a systematic review. Medicine (Baltimore). 2015;94(1):e334.

37. Faranda C, Barrat C, Catheline JM, Champault GG. Two-stage laparoscopic management of generalized peritonitis due to perforated sigmoid diverticula: eighteen cases. Surg Laparosc Endosc Percutan Tech. 2000;10(3):135-8; discussion 9-41.

38. Franklin ME, Jr., Portillo G, Trevino JM, Gonzalez JJ, Glass JL. Long-term experience with the laparoscopic approach to perforated diverticulitis plus generalized peritonitis. World J Surg. 2008;32(7):1507-11.

39. Gaertner WB, Kwaan MR, Madoff RD, Willis D, Belzer GE, Rothenberger DA, et al. The evolving role of laparoscopy in colonic diverticular disease: a systematic review. World J Surg. 2013;37(3):629-38.

40. Galbraith N, Carter JV, Netz U, Yang D, Fry DE, McCafferty M, et al. Laparoscopic Lavage in the Management of Perforated Diverticulitis: a Contemporary Meta-analysis. J Gastrointest Surg. 2017.

41. Gehrman J, Angenete E, Bjorholt I, Bock D, Rosenberg J, Haglind E. Health economic analysis of laparoscopic lavage versus Hartmann's procedure for diverticulitis in the randomized DILALA trial. Br J Surg. 2016;103(11):1539-47.

42. Gentile V, Ferrarese A, Marola S, Surace A, Borello A, Ferrara Y, et al. Perioperative and postoperative outcomes of perforated diverticulitis Hinchey II and III: open Hartmann's procedure vs. laparoscopic lavage and drainage in the elderly. Int J Surg. 2014;12:S86-s9.

43. Greilsamer T, Abet E, Meurette G, Comy M, Hamy A, Lehur PA, et al. Is the Failure of Laparoscopic Peritoneal Lavage Predictable in Hinchey III Diverticulitis Management? Dis Colon Rectum. 2017;60(9):965-70.

44. Horesh N, Zbar AP, Nevler A, Haim N, Gutman M, Zmora O. Early experience with laparoscopic lavage in acute complicated diverticulitis. Dig Surg. 2015;32(2):108-11.

45. Karoui M, Champault A, Pautrat K, Valleur P, Cherqui D, Champault G. Laparoscopic peritoneal lavage or primary anastomosis with defunctioning stoma for Hinchey 3 complicated diverticulitis: results of a comparative study. Dis Colon Rectum. 2009;52(4):609-15.

46. Liang S, Russek K, Franklin ME, Jr. Damage control strategy for the management of perforated diverticulitis with generalized peritonitis: laparoscopic lavage and drainage vs. laparoscopic Hartmann's procedure. Surg Endosc. 2012;26(10):2835-42.

47. Lippi CE, Beatini L, Cervia S, Fabbricotti A, Miaruelli PA, Spessa E, et al. [Laparoscopic lavage and drainage in the surgical treatment of diverticular disease complicated by peritonitis]. Chir Ital. 2009;61(4):467-74.

48. Marshall JR, Buchwald PL, Gandhi J, Schultz JK, Hider PN, Frizelle FA, et al. Laparoscopic Lavage in the Management of Hinchey Grade III Diverticulitis: A Systematic Review. Ann Surg. 2017;265(4):670-6.

49. Mutter D, Bouras G, Forgione A, Vix M, Leroy J, Marescaux J. Two-stage totally minimally invasive approach for acute complicated diverticulitis. Colorectal Dis. 2006;8(6):501-5.

50. Myers E, Hurley M, O'Sullivan GC, Kavanagh D, Wilson I, Winter DC. Laparoscopic peritoneal lavage for generalized peritonitis due to perforated diverticulitis. Br J Surg. 2008;95(1):97-101.

51. Parisi A, Gemini A, Desiderio J, Petrina A, Trastulli S, Grassi V, et al. Laparoscopic peritoneal lavage: our experience and review of the literature. Wideochir Inne Tech Maloinwazyjne. 2016;11(2):83-7.

52. Penna M, Markar SR, Mackenzie H, Hompes R, Cunningham C. Laparoscopic Lavage Versus Primary Resection for Acute Perforated Diverticulitis: Review and Meta-analysis. Ann Surg. 2017.

53. Rade F, Bretagnol F, Auguste M, Di Guisto C, Huten N, de Calan L. Determinants of outcome following laparoscopic peritoneal lavage for perforated diverticulitis. Br J Surg. 2014;101(12):1602-6; discussion 6.

54. Rogers AC, Collins D, O'Sullivan GC, Winter DC. Laparoscopic lavage for perforated diverticulitis: a population analysis. Dis Colon Rectum. 2012;55(9):932-8.

55. Rossi GL, Mentz R, Bertone S, Ojea Quintana G, Bilbao S, Im VM, et al. Laparoscopic peritoneal lavage for Hinchey III diverticulitis: is it as effective as it is applicable? Dis Colon Rectum. 2014;57(12):1384-90.

56. Schultz JK, Wallon C, Blecic L, Forsmo HM, Folkesson J, Buchwald P, et al. One-year results of the SCANDIV randomized clinical trial of laparoscopic lavage versus primary resection for acute perforated diverticulitis. Br J Surg. 2017;104(10):1382-92.

57. Schultz JK, Yaqub S, Wallon C, Blecic L, Forsmo HM, Folkesson J, et al. Laparoscopic Lavage vs Primary Resection for Acute Perforated Diverticulitis: The SCANDIV Randomized Clinical Trial. Jama. 2015;314(13):1364-75.

58. Shaikh FM, Stewart PM, Walsh SR, Davies RJ. Laparoscopic peritoneal lavage or surgical resection for acute perforated sigmoid diverticulitis: A systematic review and meta-analysis. Int J Surg. 2017;38:130-7.

59. Sorrentino M, Brizzolari M, Scarpa E, Malisan D, Bruschi F, Bertozzi S, et al. Laparoscopic peritoneal lavage for perforated colonic diverticulitis: a definitive treatment? Retrospective analysis of 63 cases. Tech Coloproctol. 2015;19(2):105-10.

60. Swank HA, Mulder IM, Hoofwijk AG, Nienhuijs SW, Lange JF, Bemelman WA. Early experience with laparoscopic lavage for perforated diverticulitis. Br J Surg. 2013;100(5):704-10.

61. Taylor CJ, Layani L, Ghusn MA, White SI. Perforated diverticulitis managed by laparoscopic lavage. ANZ J Surg. 2006;76(11):962-5.

62. Thornell A, Angenete E, Bisgaard T, Bock D, Burcharth J, Heath J, et al. Laparoscopic Lavage for Perforated Diverticulitis With Purulent Peritonitis: A Randomized Trial. Ann Intern Med. 2016;164(3):137-45.

63. Vennix S, Musters GD, Mulder IM, Swank HA, Consten EC, Belgers EH, et al. Laparoscopic peritoneal lavage or sigmoidectomy for perforated diverticulitis with purulent peritonitis: a multicentre, parallel-group, randomised, open-label trial. Lancet. 2015;386(10000):1269-77.

64. Vennix S, van Dieren S, Opmeer BC, Lange JF, Bemelman WA. Cost analysis of laparoscopic lavage compared with sigmoid resection for perforated diverticulitis in the Ladies trial. Br J Surg. 2017;104(1):62-8.

65. White SI, Frenkiel B, Martin PJ. A ten-year audit of perforated sigmoid diverticulitis: highlighting the outcomes of laparoscopic lavage. Dis Colon Rectum. 2010;53(11):1537-41.

66. Vennix S, Musters G, Swank H, Mulder I, Consten E, Boermeester M, et al. Laparoscopic peritoneal lavage or sigmoidectomy for generalized peritonitis due to perforated diverticulitis; results: of a multicenter randomised trial (the ladies trial). Surgical endoscopy and other interventional techniques. 2015;29:S6.

67. Biondo S, Borao JL, Kreisler E, Golda T, Millan M, Frago R, et al. Recurrence and virulence of colonic diverticulitis in immunocompromised patients. Am J Surg. 2012;204(2):172-9.

68. Biondo S, Trenti L, Elvira J, Golda T, Kreisler E. Outcomes of colonic diverticulitis according to the reason of immunosuppression. Am J Surg. 2016;212(3):384-90.

69. Cologne KG, Skiada D, Beale E, Inaba K, Senagore AJ, Demetriades D. Effects of diabetes mellitus in patients presenting with diverticulitis: clinical correlations and disease characteristics in more than 1,000 patients. J Trauma Acute Care Surg. 2014;76(3):704-9.

70. Rattan R, Allen CJ, Sawyer RG, Mazuski J, Duane TM, Askari R, et al. Patients with Risk Factors for Complications Do Not Require Longer Antimicrobial Therapy for Complicated Intra-Abdominal Infection. Am Surg. 2016;82(9):860-6.
