# Supplementary material for: EAES and SAGES 2018 consensus conference on acute diverticulitis management: evidence-based recommendations for clinical practice
Source: Surg Endosc. 2019 Jun 27;33(9):2726–41. doi: 10.1007/s00464-019-06882-z (PMC6684540; doi:10.1007/s00464-019-06882-z)
Supplement: Supplementary file 7 — Supplementary material 7 (DOCX 57 kb) [file 464_2019_6882_MOESM7_ESM.docx]

**Topic 5: Emergency operative management of acute diverticulitis**

Literature searches identified 2295 articles. Following removal of duplicates and papers not meeting inclusion criteria, remaining abstracts were jointly reviewed by our group. After further review, 79 full articles were reviewed.

**Q5.1:** what are the indication and timing of emergency surgery in acute complicated diverticulitis?

Statement

Patients with perforated diverticulitis and peritonitis should be evaluated early for operative intervention to control infection. There is little data to inform the timing of operative intervention, but the clinical status of the patient should guide urgency of surgical intervention.

LOE: Low

Recommendations:

Patients with perforated diverticulitis with diffuse peritonitis (Hinchey 3 and 4) should undergo emergent surgical intervention.

SOR: Strong for using

Although many authors have attempted to accurately predict the indications and timing of surgery for acute complicated diverticulitis, they have not agreed on specific guidelines. Indeed, the literature is not consistent in identifying an exact time for surgical intervention beyond patient’s hemodynamic status, the most important indicator for the urgency of surgery. Extracolonic air on abdominal CT scan which has a sensitivity of 76-100% and specificity of 83-91% in detecting colonic perforation, especially when found at a distance from the site of perforation, is a radiological sign predictive of diffuse peritonitis and commonly considered an indication for urgent operation (Gielens 2012). However, Costi et al published a series of patients with the presence of extradigestive air on CT who were managed safely by antibiotic therapy alone in 36 out of 39 patients. Although the literature is not consistent in identifying timing of surgical intervention beyond patient’s hemodynamic status, the patient’s clinical factors should establish the indication and urgency of surgery.

**References**

1. [Shaikh FM](https://www.ncbi.nlm.nih.gov/pubmed/?term=Shaikh%20FM%5BAuthor%5D&cauthor=true&cauthor_uid=28089941), [Stewart PM](https://www.ncbi.nlm.nih.gov/pubmed/?term=Stewart%20PM%5BAuthor%5D&cauthor=true&cauthor_uid=28089941), [Walsh SR](https://www.ncbi.nlm.nih.gov/pubmed/?term=Walsh%20SR%5BAuthor%5D&cauthor=true&cauthor_uid=28089941), [Davies RJ](https://www.ncbi.nlm.nih.gov/pubmed/?term=Davies%20RJ%5BAuthor%5D&cauthor=true&cauthor_uid=28089941). Laparoscopic peritoneal lavage or surgical resection for acute perforated sigmoid diverticulitis: A systematic review and meta-analysis. [Int J Surg.](https://www.ncbi.nlm.nih.gov/pubmed/?term=Int+J+Surg.+2017+Feb%3B38%3A130-137) 2017; 38:130-137
2. [Angenete E](https://www.ncbi.nlm.nih.gov/pubmed/?term=Angenete%20E%5BAuthor%5D&cauthor=true&cauthor_uid=27567926), [Bock D](https://www.ncbi.nlm.nih.gov/pubmed/?term=Bock%20D%5BAuthor%5D&cauthor=true&cauthor_uid=27567926), [Rosenberg J](https://www.ncbi.nlm.nih.gov/pubmed/?term=Rosenberg%20J%5BAuthor%5D&cauthor=true&cauthor_uid=27567926), [Haglind E](https://www.ncbi.nlm.nih.gov/pubmed/?term=Haglind%20E%5BAuthor%5D&cauthor=true&cauthor_uid=27567926). Laparoscopic lavage is superior to colon resection for perforated purulent diverticulitis-a meta-analysis. [Int J Colorectal Dis.](https://www.ncbi.nlm.nih.gov/pubmed/?term=Int+J+Colorectal+Dis.+2017+Feb%3B32(2)%3A163-169) 2017;32(2):163-169
3. [Cirocchi R](https://www.ncbi.nlm.nih.gov/pubmed/?term=Cirocchi%20R%5BAuthor%5D&cauthor=true&cauthor_uid=28197792), [Di Saverio S](https://www.ncbi.nlm.nih.gov/pubmed/?term=Di%20Saverio%20S%5BAuthor%5D&cauthor=true&cauthor_uid=28197792), [Weber DG](https://www.ncbi.nlm.nih.gov/pubmed/?term=Weber%20DG%5BAuthor%5D&cauthor=true&cauthor_uid=28197792), [Taboła R](https://www.ncbi.nlm.nih.gov/pubmed/?term=Tabo%C5%82a%20R%5BAuthor%5D&cauthor=true&cauthor_uid=28197792), [Abraha I](https://www.ncbi.nlm.nih.gov/pubmed/?term=Abraha%20I%5BAuthor%5D&cauthor=true&cauthor_uid=28197792), [Randolph J](https://www.ncbi.nlm.nih.gov/pubmed/?term=Randolph%20J%5BAuthor%5D&cauthor=true&cauthor_uid=28197792), [Arezzo A](https://www.ncbi.nlm.nih.gov/pubmed/?term=Arezzo%20A%5BAuthor%5D&cauthor=true&cauthor_uid=28197792), [Binda GA](https://www.ncbi.nlm.nih.gov/pubmed/?term=Binda%20GA%5BAuthor%5D&cauthor=true&cauthor_uid=28197792). Laparoscopic lavage versus surgical resection for acute diverticulitis with generalised peritonitis: a systematic review and meta-analysis. [Tech Coloproctol.](https://www.ncbi.nlm.nih.gov/pubmed/?term=Tech+Coloproctol.+2017+Feb%3B21(2)%3A93-110) 2017;21(2):93-110
4. [Galbraith N](https://www.ncbi.nlm.nih.gov/pubmed/?term=Galbraith%20N%5BAuthor%5D&cauthor=true&cauthor_uid=28608041), [Carter JV](https://www.ncbi.nlm.nih.gov/pubmed/?term=Carter%20JV%5BAuthor%5D&cauthor=true&cauthor_uid=28608041), [Netz U](https://www.ncbi.nlm.nih.gov/pubmed/?term=Netz%20U%5BAuthor%5D&cauthor=true&cauthor_uid=28608041), [Yang D](https://www.ncbi.nlm.nih.gov/pubmed/?term=Yang%20D%5BAuthor%5D&cauthor=true&cauthor_uid=28608041), [Fry DE](https://www.ncbi.nlm.nih.gov/pubmed/?term=Fry%20DE%5BAuthor%5D&cauthor=true&cauthor_uid=28608041), [McCafferty M](https://www.ncbi.nlm.nih.gov/pubmed/?term=McCafferty%20M%5BAuthor%5D&cauthor=true&cauthor_uid=28608041), [Galandiuk S](https://www.ncbi.nlm.nih.gov/pubmed/?term=Galandiuk%20S%5BAuthor%5D&cauthor=true&cauthor_uid=28608041). Laparoscopic Lavage in the Management of Perforated Diverticulitis: a Contemporary Meta-analysis. [J Gastrointest Surg.](https://www.ncbi.nlm.nih.gov/pubmed/?term=J+Gastrointest+Surg.+2017+Sep%3B21(9)%3A1491-1499) 2017;21(9):1491-1499
5. [Ceresoli M](https://www.ncbi.nlm.nih.gov/pubmed/?term=Ceresoli%20M%5BAuthor%5D&cauthor=true&cauthor_uid=27582782), [Coccolini F](https://www.ncbi.nlm.nih.gov/pubmed/?term=Coccolini%20F%5BAuthor%5D&cauthor=true&cauthor_uid=27582782), [Montori G](https://www.ncbi.nlm.nih.gov/pubmed/?term=Montori%20G%5BAuthor%5D&cauthor=true&cauthor_uid=27582782), [Catena F](https://www.ncbi.nlm.nih.gov/pubmed/?term=Catena%20F%5BAuthor%5D&cauthor=true&cauthor_uid=27582782), [Sartelli M](https://www.ncbi.nlm.nih.gov/pubmed/?term=Sartelli%20M%5BAuthor%5D&cauthor=true&cauthor_uid=27582782), [Ansaloni L](https://www.ncbi.nlm.nih.gov/pubmed/?term=Ansaloni%20L%5BAuthor%5D&cauthor=true&cauthor_uid=27582782). Laparoscopic lavage versus resection in perforated diverticulitis with purulent peritonitis: a meta-analysis of randomized controlled trials. [World J Emerg Surg.](https://www.ncbi.nlm.nih.gov/pubmed/?term=World+J+Emerg+Surg.+2016+Aug+30%3B11(1)%3A42) 2016;11(1):42
6. Cirocchi R, Trastulli S, Vettoretto N, Milani D, Cavaliere D, Renzi C, Adamenko O, Desiderio J, Burattini MF, Parisi A, Arezzo A, Fingerhut A. [Laparoscopic peritoneal lavage: a definitive treatment for diverticular peritonitis or a "bridge" to elective laparoscopic sigmoidectomy?: a systematic review.](https://www.ncbi.nlm.nih.gov/pubmed/25569649) Medicine (Baltimore). 2015;94(1):e334
7. [Afshar S](https://www.ncbi.nlm.nih.gov/pubmed/?term=Afshar%20S%5BAuthor%5D&cauthor=true&cauthor_uid=21689299), [Kurer MA](https://www.ncbi.nlm.nih.gov/pubmed/?term=Kurer%20MA%5BAuthor%5D&cauthor=true&cauthor_uid=21689299). Laparoscopic peritoneal lavage for perforated sigmoid diverticulitis. [Colorectal Dis.](https://www.ncbi.nlm.nih.gov/pubmed/?term=Colorectal+Dis.+2012+Feb%3B14(2)%3A135-42) 2012;14(2):135-42
8. [Toorenvliet BR](https://www.ncbi.nlm.nih.gov/pubmed/?term=Toorenvliet%20BR%5BAuthor%5D&cauthor=true&cauthor_uid=19788490), [Swank H](https://www.ncbi.nlm.nih.gov/pubmed/?term=Swank%20H%5BAuthor%5D&cauthor=true&cauthor_uid=19788490), [Schoones JW](https://www.ncbi.nlm.nih.gov/pubmed/?term=Schoones%20JW%5BAuthor%5D&cauthor=true&cauthor_uid=19788490), [Hamming JF](https://www.ncbi.nlm.nih.gov/pubmed/?term=Hamming%20JF%5BAuthor%5D&cauthor=true&cauthor_uid=19788490), [Bemelman WA](https://www.ncbi.nlm.nih.gov/pubmed/?term=Bemelman%20WA%5BAuthor%5D&cauthor=true&cauthor_uid=19788490). Laparoscopic peritoneal lavage for perforated colonic diverticulitis: a systematic review. [Colorectal Dis.](https://www.ncbi.nlm.nih.gov/pubmed/?term=Colorectal+Dis.+2010+Sep%3B12(9)%3A862-7) 2010;12(9):862-7
9. Penna M, Markar SR, Mackenzie H, Hompes R, Cunningham C. [Laparoscopic Lavage Versus Primary Resection for Acute Perforated Diverticulitis: Review and Meta-analysis.](https://www.ncbi.nlm.nih.gov/pubmed/28338510) Ann Surg. 2018;267(2):252-258
10. [Marshall JR](https://www.ncbi.nlm.nih.gov/pubmed/?term=Marshall%20JR%5BAuthor%5D&cauthor=true&cauthor_uid=27631772), [Buchwald PL](https://www.ncbi.nlm.nih.gov/pubmed/?term=Buchwald%20PL%5BAuthor%5D&cauthor=true&cauthor_uid=27631772), [Gandhi J](https://www.ncbi.nlm.nih.gov/pubmed/?term=Gandhi%20J%5BAuthor%5D&cauthor=true&cauthor_uid=27631772), [Schultz JK](https://www.ncbi.nlm.nih.gov/pubmed/?term=Schultz%20JK%5BAuthor%5D&cauthor=true&cauthor_uid=27631772), [Hider PN](https://www.ncbi.nlm.nih.gov/pubmed/?term=Hider%20PN%5BAuthor%5D&cauthor=true&cauthor_uid=27631772), [Frizelle FA](https://www.ncbi.nlm.nih.gov/pubmed/?term=Frizelle%20FA%5BAuthor%5D&cauthor=true&cauthor_uid=27631772), [Eglinton TW](https://www.ncbi.nlm.nih.gov/pubmed/?term=Eglinton%20TW%5BAuthor%5D&cauthor=true&cauthor_uid=27631772). Laparoscopic Lavage in the Management of Hinchey Grade III Diverticulitis: A Systematic Review. [Ann Surg.](https://www.ncbi.nlm.nih.gov/pubmed/?term=Ann+Surg2017%3B265%3A670%E2%80%93676) 2017;265(4):670-676
11. [Alamili M](https://www.ncbi.nlm.nih.gov/pubmed/?term=Alamili%20M%5BAuthor%5D&cauthor=true&cauthor_uid=19571714), [Gögenur I](https://www.ncbi.nlm.nih.gov/pubmed/?term=G%C3%B6genur%20I%5BAuthor%5D&cauthor=true&cauthor_uid=19571714), [Rosenberg J](https://www.ncbi.nlm.nih.gov/pubmed/?term=Rosenberg%20J%5BAuthor%5D&cauthor=true&cauthor_uid=19571714). Acute complicated diverticulitis managed by laparoscopic lavage. [Dis Colon Rectum.](https://www.ncbi.nlm.nih.gov/pubmed/?term=Dis+Colon+Rectum.+2009+Jul%3B52(7)%3A1345-9) 2009;52(7):1345-9
12. [Schultz JK](https://www.ncbi.nlm.nih.gov/pubmed/?term=Schultz%20JK%5BAuthor%5D&cauthor=true&cauthor_uid=28631827), [Wallon C](https://www.ncbi.nlm.nih.gov/pubmed/?term=Wallon%20C%5BAuthor%5D&cauthor=true&cauthor_uid=28631827), [Blecic L](https://www.ncbi.nlm.nih.gov/pubmed/?term=Blecic%20L%5BAuthor%5D&cauthor=true&cauthor_uid=28631827), [Forsmo HM](https://www.ncbi.nlm.nih.gov/pubmed/?term=Forsmo%20HM%5BAuthor%5D&cauthor=true&cauthor_uid=28631827), [Folkesson J](https://www.ncbi.nlm.nih.gov/pubmed/?term=Folkesson%20J%5BAuthor%5D&cauthor=true&cauthor_uid=28631827), [Buchwald P](https://www.ncbi.nlm.nih.gov/pubmed/?term=Buchwald%20P%5BAuthor%5D&cauthor=true&cauthor_uid=28631827), [Kørner H](https://www.ncbi.nlm.nih.gov/pubmed/?term=K%C3%B8rner%20H%5BAuthor%5D&cauthor=true&cauthor_uid=28631827), [Dahl FA](https://www.ncbi.nlm.nih.gov/pubmed/?term=Dahl%20FA%5BAuthor%5D&cauthor=true&cauthor_uid=28631827), [Øresland T](https://www.ncbi.nlm.nih.gov/pubmed/?term=%C3%98resland%20T%5BAuthor%5D&cauthor=true&cauthor_uid=28631827), [Yaqub S](https://www.ncbi.nlm.nih.gov/pubmed/?term=Yaqub%20S%5BAuthor%5D&cauthor=true&cauthor_uid=28631827); [SCANDIV Study Group](https://www.ncbi.nlm.nih.gov/pubmed/?term=SCANDIV%20Study%20Group%5BCorporate%20Author%5D) One-year results of the SCANDIV randomized clinical trial of laparoscopic lavage versus primary resection for acute perforated diverticulitis. [Br J Surg.](https://www.ncbi.nlm.nih.gov/pubmed/?term=BJS+2017%3B+104%3A+1382%E2%80%931392) 2017;104(10):1382-1392
13. [Thornell A](https://www.ncbi.nlm.nih.gov/pubmed/?term=Thornell%20A%5BAuthor%5D&cauthor=true&cauthor_uid=26784672), [Angenete E](https://www.ncbi.nlm.nih.gov/pubmed/?term=Angenete%20E%5BAuthor%5D&cauthor=true&cauthor_uid=26784672), [Bisgaard T](https://www.ncbi.nlm.nih.gov/pubmed/?term=Bisgaard%20T%5BAuthor%5D&cauthor=true&cauthor_uid=26784672), [Bock D](https://www.ncbi.nlm.nih.gov/pubmed/?term=Bock%20D%5BAuthor%5D&cauthor=true&cauthor_uid=26784672), [Burcharth J](https://www.ncbi.nlm.nih.gov/pubmed/?term=Burcharth%20J%5BAuthor%5D&cauthor=true&cauthor_uid=26784672), [Heath J](https://www.ncbi.nlm.nih.gov/pubmed/?term=Heath%20J%5BAuthor%5D&cauthor=true&cauthor_uid=26784672), [Pommergaard HC](https://www.ncbi.nlm.nih.gov/pubmed/?term=Pommergaard%20HC%5BAuthor%5D&cauthor=true&cauthor_uid=26784672), [Rosenberg J](https://www.ncbi.nlm.nih.gov/pubmed/?term=Rosenberg%20J%5BAuthor%5D&cauthor=true&cauthor_uid=26784672), [Stilling N](https://www.ncbi.nlm.nih.gov/pubmed/?term=Stilling%20N%5BAuthor%5D&cauthor=true&cauthor_uid=26784672), [Skullman S](https://www.ncbi.nlm.nih.gov/pubmed/?term=Skullman%20S%5BAuthor%5D&cauthor=true&cauthor_uid=26784672), [Haglind E](https://www.ncbi.nlm.nih.gov/pubmed/?term=Haglind%20E%5BAuthor%5D&cauthor=true&cauthor_uid=26784672) Laparoscopic Lavage for Perforated Diverticulitis With Purulent Peritonitis: A Randomized Trial. [Ann Intern Med.](https://www.ncbi.nlm.nih.gov/pubmed/?term=Ann+Intern+Med.+2016+Feb+2%3B164(3)%3A137-45) 2016;164(3):137-45
14. [Vennix S](https://www.ncbi.nlm.nih.gov/pubmed/?term=Vennix%20S%5BAuthor%5D&cauthor=true&cauthor_uid=26209030), [Musters GD](https://www.ncbi.nlm.nih.gov/pubmed/?term=Musters%20GD%5BAuthor%5D&cauthor=true&cauthor_uid=26209030), [Mulder IM](https://www.ncbi.nlm.nih.gov/pubmed/?term=Mulder%20IM%5BAuthor%5D&cauthor=true&cauthor_uid=26209030), [Swank HA](https://www.ncbi.nlm.nih.gov/pubmed/?term=Swank%20HA%5BAuthor%5D&cauthor=true&cauthor_uid=26209030), [Consten EC](https://www.ncbi.nlm.nih.gov/pubmed/?term=Consten%20EC%5BAuthor%5D&cauthor=true&cauthor_uid=26209030), [Belgers EH](https://www.ncbi.nlm.nih.gov/pubmed/?term=Belgers%20EH%5BAuthor%5D&cauthor=true&cauthor_uid=26209030), [van Geloven AA](https://www.ncbi.nlm.nih.gov/pubmed/?term=van%20Geloven%20AA%5BAuthor%5D&cauthor=true&cauthor_uid=26209030), [Gerhards MF](https://www.ncbi.nlm.nih.gov/pubmed/?term=Gerhards%20MF%5BAuthor%5D&cauthor=true&cauthor_uid=26209030), [Govaert MJ](https://www.ncbi.nlm.nih.gov/pubmed/?term=Govaert%20MJ%5BAuthor%5D&cauthor=true&cauthor_uid=26209030), [van Grevenstein WM](https://www.ncbi.nlm.nih.gov/pubmed/?term=van%20Grevenstein%20WM%5BAuthor%5D&cauthor=true&cauthor_uid=26209030), [Hoofwijk AG](https://www.ncbi.nlm.nih.gov/pubmed/?term=Hoofwijk%20AG%5BAuthor%5D&cauthor=true&cauthor_uid=26209030), [Kruyt PM](https://www.ncbi.nlm.nih.gov/pubmed/?term=Kruyt%20PM%5BAuthor%5D&cauthor=true&cauthor_uid=26209030), [Nienhuijs SW](https://www.ncbi.nlm.nih.gov/pubmed/?term=Nienhuijs%20SW%5BAuthor%5D&cauthor=true&cauthor_uid=26209030), [Boermeester MA](https://www.ncbi.nlm.nih.gov/pubmed/?term=Boermeester%20MA%5BAuthor%5D&cauthor=true&cauthor_uid=26209030), [Vermeulen J](https://www.ncbi.nlm.nih.gov/pubmed/?term=Vermeulen%20J%5BAuthor%5D&cauthor=true&cauthor_uid=26209030), [van Dieren S](https://www.ncbi.nlm.nih.gov/pubmed/?term=van%20Dieren%20S%5BAuthor%5D&cauthor=true&cauthor_uid=26209030), [Lange JF](https://www.ncbi.nlm.nih.gov/pubmed/?term=Lange%20JF%5BAuthor%5D&cauthor=true&cauthor_uid=26209030), [Bemelman WA](https://www.ncbi.nlm.nih.gov/pubmed/?term=Bemelman%20WA%5BAuthor%5D&cauthor=true&cauthor_uid=26209030); [Ladies trial colloborators](https://www.ncbi.nlm.nih.gov/pubmed/?term=Ladies%20trial%20colloborators%5BCorporate%20Author%5D). Laparoscopic peritoneal lavage or sigmoidectomy for perforated diverticulitis with purulent peritonitis: a multicentre, parallel-group, randomised, open-label trial. [Lancet.](https://www.ncbi.nlm.nih.gov/pubmed/?term=Lancet.+2015+Sep+26%3B386(10000)%3A1269-1277) 2015;386(10000):1269-1277
15. [Schultz JK](https://www.ncbi.nlm.nih.gov/pubmed/?term=Schultz%20JK%5BAuthor%5D&cauthor=true&cauthor_uid=26441181), [Yaqub S](https://www.ncbi.nlm.nih.gov/pubmed/?term=Yaqub%20S%5BAuthor%5D&cauthor=true&cauthor_uid=26441181), [Wallon C](https://www.ncbi.nlm.nih.gov/pubmed/?term=Wallon%20C%5BAuthor%5D&cauthor=true&cauthor_uid=26441181), [Blecic L](https://www.ncbi.nlm.nih.gov/pubmed/?term=Blecic%20L%5BAuthor%5D&cauthor=true&cauthor_uid=26441181), [Forsmo HM](https://www.ncbi.nlm.nih.gov/pubmed/?term=Forsmo%20HM%5BAuthor%5D&cauthor=true&cauthor_uid=26441181), [Folkesson J](https://www.ncbi.nlm.nih.gov/pubmed/?term=Folkesson%20J%5BAuthor%5D&cauthor=true&cauthor_uid=26441181), [Buchwald P](https://www.ncbi.nlm.nih.gov/pubmed/?term=Buchwald%20P%5BAuthor%5D&cauthor=true&cauthor_uid=26441181), [Körner H](https://www.ncbi.nlm.nih.gov/pubmed/?term=K%C3%B6rner%20H%5BAuthor%5D&cauthor=true&cauthor_uid=26441181), [Dahl FA](https://www.ncbi.nlm.nih.gov/pubmed/?term=Dahl%20FA%5BAuthor%5D&cauthor=true&cauthor_uid=26441181), [Øresland T](https://www.ncbi.nlm.nih.gov/pubmed/?term=%C3%98resland%20T%5BAuthor%5D&cauthor=true&cauthor_uid=26441181); [SCANDIV Study Group](https://www.ncbi.nlm.nih.gov/pubmed/?term=SCANDIV%20Study%20Group%5BCorporate%20Author%5D). Laparoscopic Lavage vs Primary Resection for Acute Perforated Diverticulitis: The SCANDIV Randomized Clinical Trial. [JAMA.](https://www.ncbi.nlm.nih.gov/pubmed/?term=JAMA.+2015+Oct+6%3B314(13)%3A1364-75) 2015;314(13):1364-75
16. [Angenete E](https://www.ncbi.nlm.nih.gov/pubmed/?term=Angenete%20E%5BAuthor%5D&cauthor=true&cauthor_uid=25489672), [Thornell A](https://www.ncbi.nlm.nih.gov/pubmed/?term=Thornell%20A%5BAuthor%5D&cauthor=true&cauthor_uid=25489672), [Burcharth J](https://www.ncbi.nlm.nih.gov/pubmed/?term=Burcharth%20J%5BAuthor%5D&cauthor=true&cauthor_uid=25489672), [Pommergaard HC](https://www.ncbi.nlm.nih.gov/pubmed/?term=Pommergaard%20HC%5BAuthor%5D&cauthor=true&cauthor_uid=25489672), [Skullman S](https://www.ncbi.nlm.nih.gov/pubmed/?term=Skullman%20S%5BAuthor%5D&cauthor=true&cauthor_uid=25489672), [Bisgaard T](https://www.ncbi.nlm.nih.gov/pubmed/?term=Bisgaard%20T%5BAuthor%5D&cauthor=true&cauthor_uid=25489672), [Jess P](https://www.ncbi.nlm.nih.gov/pubmed/?term=Jess%20P%5BAuthor%5D&cauthor=true&cauthor_uid=25489672), [Läckberg Z](https://www.ncbi.nlm.nih.gov/pubmed/?term=L%C3%A4ckberg%20Z%5BAuthor%5D&cauthor=true&cauthor_uid=25489672), [Matthiessen P](https://www.ncbi.nlm.nih.gov/pubmed/?term=Matthiessen%20P%5BAuthor%5D&cauthor=true&cauthor_uid=25489672), [Heath J](https://www.ncbi.nlm.nih.gov/pubmed/?term=Heath%20J%5BAuthor%5D&cauthor=true&cauthor_uid=25489672), [Rosenberg J](https://www.ncbi.nlm.nih.gov/pubmed/?term=Rosenberg%20J%5BAuthor%5D&cauthor=true&cauthor_uid=25489672), [Haglind E](https://www.ncbi.nlm.nih.gov/pubmed/?term=Haglind%20E%5BAuthor%5D&cauthor=true&cauthor_uid=25489672). Laparoscopic Lavage Is Feasible and Safe for the Treatment of Perforated Diverticulitis With Purulent Peritonitis: The First Results From the Randomized Controlled Trial DILALA. [Ann Surg.](https://www.ncbi.nlm.nih.gov/pubmed/?term=Ann+Surg+2016%3B263%3A117%E2%80%93122) 2016;263(1):117-22.
17. [Costi R](https://www.ncbi.nlm.nih.gov/pubmed/?term=Costi%20R%5BAuthor%5D&cauthor=true&cauthor_uid=22274929), [Cauchy F](https://www.ncbi.nlm.nih.gov/pubmed/?term=Cauchy%20F%5BAuthor%5D&cauthor=true&cauthor_uid=22274929), [Le Bian A](https://www.ncbi.nlm.nih.gov/pubmed/?term=Le%20Bian%20A%5BAuthor%5D&cauthor=true&cauthor_uid=22274929), [Honart JF](https://www.ncbi.nlm.nih.gov/pubmed/?term=Honart%20JF%5BAuthor%5D&cauthor=true&cauthor_uid=22274929), [Creuze N](https://www.ncbi.nlm.nih.gov/pubmed/?term=Creuze%20N%5BAuthor%5D&cauthor=true&cauthor_uid=22274929), [Smadja C](https://www.ncbi.nlm.nih.gov/pubmed/?term=Smadja%20C%5BAuthor%5D&cauthor=true&cauthor_uid=22274929). Challenging a classic myth: pneumoperitoneum associated with acute diverticulitis is not an indication for open or laparoscopic emergency surgery in hemodynamically stable patients. A 10-year experience with a nonoperative treatment. [Surg Endosc.](https://www.ncbi.nlm.nih.gov/pubmed/?term=19.%09Surg+Endosc.+2012+Jul%3B26(7)%3A2061-71.) 2012;26(7):2061-71
18. [[Gielens MP](https://www.ncbi.nlm.nih.gov/pubmed/?term=Gielens%20MP%5BAuthor%5D&cauthor=true&cauthor_uid=22752330), [Mulder IM](https://www.ncbi.nlm.nih.gov/pubmed/?term=Mulder%20IM%5BAuthor%5D&cauthor=true&cauthor_uid=22752330), [van der Harst E](https://www.ncbi.nlm.nih.gov/pubmed/?term=van%20der%20Harst%20E%5BAuthor%5D&cauthor=true&cauthor_uid=22752330), [Gosselink MP](https://www.ncbi.nlm.nih.gov/pubmed/?term=Gosselink%20MP%5BAuthor%5D&cauthor=true&cauthor_uid=22752330), [Kraal KJ](https://www.ncbi.nlm.nih.gov/pubmed/?term=Kraal%20KJ%5BAuthor%5D&cauthor=true&cauthor_uid=22752330), [Teng HT](https://www.ncbi.nlm.nih.gov/pubmed/?term=Teng%20HT%5BAuthor%5D&cauthor=true&cauthor_uid=22752330), [Lange JF](https://www.ncbi.nlm.nih.gov/pubmed/?term=Lange%20JF%5BAuthor%5D&cauthor=true&cauthor_uid=22752330), [Vermeulen J](https://www.ncbi.nlm.nih.gov/pubmed/?term=Vermeulen%20J%5BAuthor%5D&cauthor=true&cauthor_uid=22752330). Preoperative staging of perforated diverticulitis by computed tomography scanning. [Tech Coloproctol](https://www.ncbi.nlm.nih.gov/pubmed/?term=Tech+Coloproctol.+2012+Oct%3B16(5)%3A363-8);16(5):363-8](https://www.ncbi.nlm.nih.gov/pubmed/?term=Preoperative+staging+of+perforated+diverticulitis+by+computed+tomography+scanning)
19. [Käser SA](https://www.ncbi.nlm.nih.gov/pubmed/?term=K%C3%A4ser%20SA%5BAuthor%5D&cauthor=true&cauthor_uid=23476637), [Furler R](https://www.ncbi.nlm.nih.gov/pubmed/?term=Furler%20R%5BAuthor%5D&cauthor=true&cauthor_uid=23476637), [Evequoz DC](https://www.ncbi.nlm.nih.gov/pubmed/?term=Evequoz%20DC%5BAuthor%5D&cauthor=true&cauthor_uid=23476637), [Maurer CA](https://www.ncbi.nlm.nih.gov/pubmed/?term=Maurer%20CA%5BAuthor%5D&cauthor=true&cauthor_uid=23476637). Hyponatremia is a specific marker of perforation in sigmoid diverticulitis or appendicitis in patients older than 50 years. [Gastroenterol Res Pract.](https://www.ncbi.nlm.nih.gov/pubmed/?term=23.%09Gastroenterol+Res+Pract.+2013%3B2013%3A462891) 2013;2013:462891

Note: As discussed during the meeting; consideration of removal of this question should be made as it encompasses the bigger question of indications and timing of surgical interventions in terms of failed medical management for ex.

**Q5.2:** What is the role of laparoscopic resection in emergency surgery for diverticulitis?

Statement

Laparoscopic sigmoid resection with or without stoma in the emergency setting has been shown to decrease overall complications compared to open resections.

LOE: Low

Recommendations:

When resection is indicated, we recommend consideration of laparoscopic approach for perforated diverticulitis in the appropriate clinical setting.

SOR: Weak

The benefits of laparoscopic surgery in elective colorectal surgery have also been apparent in emergency setting of perforated diverticulitis. Vennix et al published in 2016 a SR of retrospective studies collecting 104 patients who underwent laparoscopic emergency resections (84 Hartmann's procedures and 20 primary anastomoses) with Hinchey 3 or 4 diverticulitis. They reported an overall morbidity of 21% with no reports of anastomotic leaks, an overall mortality of 2.9%, and a conversion rate up to 20%. Many retrospective studies compared laparoscopic versus open resections in the emergency settings, reporting comparable morbidity and mortality (Karoui 2009, Rea 2012, Zdichawsky 2013) or lower morbidity and mortality in laparoscopic group (Vennix 2016, Letarte 2015, Turley 2013). A consistent benefit of laparoscopy in the retrospective series was the shorter length of hospital stay compared to open surgery (6-7 days versus 8.5-9 days). Care must be taken when interpreting this data as some of these series may be affected by selection bias, including patients with a lower grade of peritonitis in the laparoscopic group compared to the open group(Letarte, Titu, White). Nevertheless laparoscopic technique seems to reduce post-operative morbidity and to shorten post-operative hospital stay. Peritonitis brings on specific challenges for the laparoscopic surgeon thus an expertise in laparoscopy and diverticular colorectal resections are required to safely perform these procedures.

**References**

1. [Vennix S](https://www.ncbi.nlm.nih.gov/pubmed/?term=Vennix%20S%5BAuthor%5D&cauthor=true&cauthor_uid=26551040), [Boersema GS](https://www.ncbi.nlm.nih.gov/pubmed/?term=Boersema%20GS%5BAuthor%5D&cauthor=true&cauthor_uid=26551040), [Buskens CJ](https://www.ncbi.nlm.nih.gov/pubmed/?term=Buskens%20CJ%5BAuthor%5D&cauthor=true&cauthor_uid=26551040), [Menon AG](https://www.ncbi.nlm.nih.gov/pubmed/?term=Menon%20AG%5BAuthor%5D&cauthor=true&cauthor_uid=26551040), [Tanis PJ](https://www.ncbi.nlm.nih.gov/pubmed/?term=Tanis%20PJ%5BAuthor%5D&cauthor=true&cauthor_uid=26551040), [Lange JF](https://www.ncbi.nlm.nih.gov/pubmed/?term=Lange%20JF%5BAuthor%5D&cauthor=true&cauthor_uid=26551040), [Bemelman WA](https://www.ncbi.nlm.nih.gov/pubmed/?term=Bemelman%20WA%5BAuthor%5D&cauthor=true&cauthor_uid=26551040). Emergency Laparoscopic Sigmoidectomy for Perforated Diverticulitis with Generalised Peritonitis: A Systematic Review. [Dig Surg.](https://www.ncbi.nlm.nih.gov/pubmed/?term=Dig+Surg.+2016%3B33(1)%3A1-7) 2016;33(1):1-7
2. [Karoui M](https://www.ncbi.nlm.nih.gov/pubmed/?term=Karoui%20M%5BAuthor%5D&cauthor=true&cauthor_uid=19404062), [Champault A](https://www.ncbi.nlm.nih.gov/pubmed/?term=Champault%20A%5BAuthor%5D&cauthor=true&cauthor_uid=19404062), [Pautrat K](https://www.ncbi.nlm.nih.gov/pubmed/?term=Pautrat%20K%5BAuthor%5D&cauthor=true&cauthor_uid=19404062), [Valleur P](https://www.ncbi.nlm.nih.gov/pubmed/?term=Valleur%20P%5BAuthor%5D&cauthor=true&cauthor_uid=19404062), [Cherqui D](https://www.ncbi.nlm.nih.gov/pubmed/?term=Cherqui%20D%5BAuthor%5D&cauthor=true&cauthor_uid=19404062), [Champault G](https://www.ncbi.nlm.nih.gov/pubmed/?term=Champault%20G%5BAuthor%5D&cauthor=true&cauthor_uid=19404062). Laparoscopic peritoneal lavage or primary anastomosis with defunctioning stoma for Hinchey 3 complicated diverticulitis: results of a comparative study. [Dis Colon Rectum.](https://www.ncbi.nlm.nih.gov/pubmed/?term=Dis+Colon+Rectum+2009%3B+52%3A+609Y615) 2009;52(4):609-15.
3. [Rea JD](https://www.ncbi.nlm.nih.gov/pubmed/?term=Rea%20JD%5BAuthor%5D&cauthor=true&cauthor_uid=22444830), [Herzig DO](https://www.ncbi.nlm.nih.gov/pubmed/?term=Herzig%20DO%5BAuthor%5D&cauthor=true&cauthor_uid=22444830), [Diggs BS](https://www.ncbi.nlm.nih.gov/pubmed/?term=Diggs%20BS%5BAuthor%5D&cauthor=true&cauthor_uid=22444830), [Cone MM](https://www.ncbi.nlm.nih.gov/pubmed/?term=Cone%20MM%5BAuthor%5D&cauthor=true&cauthor_uid=22444830), [Lu KC](https://www.ncbi.nlm.nih.gov/pubmed/?term=Lu%20KC%5BAuthor%5D&cauthor=true&cauthor_uid=22444830). Use and outcomes of emergent laparoscopic resection for acute diverticulitis. [Am J Surg.](https://www.ncbi.nlm.nih.gov/pubmed/?term=Am+J+Surg.+2012+May%3B203(5)%3A639-43) 2012;203(5):639-43
4. [Zdichavsky M](https://www.ncbi.nlm.nih.gov/pubmed/?term=Zdichavsky%20M%5BAuthor%5D&cauthor=true&cauthor_uid=23918084), [Kratt T](https://www.ncbi.nlm.nih.gov/pubmed/?term=Kratt%20T%5BAuthor%5D&cauthor=true&cauthor_uid=23918084), [Stüker D](https://www.ncbi.nlm.nih.gov/pubmed/?term=St%C3%BCker%20D%5BAuthor%5D&cauthor=true&cauthor_uid=23918084), [Meile T](https://www.ncbi.nlm.nih.gov/pubmed/?term=Meile%20T%5BAuthor%5D&cauthor=true&cauthor_uid=23918084), [Feilitzsch MV](https://www.ncbi.nlm.nih.gov/pubmed/?term=Feilitzsch%20MV%5BAuthor%5D&cauthor=true&cauthor_uid=23918084), [Wichmann D](https://www.ncbi.nlm.nih.gov/pubmed/?term=Wichmann%20D%5BAuthor%5D&cauthor=true&cauthor_uid=23918084), [Königsrainer A](https://www.ncbi.nlm.nih.gov/pubmed/?term=K%C3%B6nigsrainer%20A%5BAuthor%5D&cauthor=true&cauthor_uid=23918084). Acute and elective laparoscopic resection for complicated sigmoid diverticulitis: clinical and histological outcome. [J Gastrointest Surg.](https://www.ncbi.nlm.nih.gov/pubmed/?term=Acute+and+elective+laparoscopic+resection+for+complicated+sigmoid+diverticulitis%3A+clinical+and+histological+outcome) 2013;17(11):1966-71
5. [Vennix S](https://www.ncbi.nlm.nih.gov/pubmed/?term=Vennix%20S%5BAuthor%5D&cauthor=true&cauthor_uid=26679173), [Lips DJ](https://www.ncbi.nlm.nih.gov/pubmed/?term=Lips%20DJ%5BAuthor%5D&cauthor=true&cauthor_uid=26679173), [Di Saverio S](https://www.ncbi.nlm.nih.gov/pubmed/?term=Di%20Saverio%20S%5BAuthor%5D&cauthor=true&cauthor_uid=26679173), [van Wagensveld BA](https://www.ncbi.nlm.nih.gov/pubmed/?term=van%20Wagensveld%20BA%5BAuthor%5D&cauthor=true&cauthor_uid=26679173), [Brokelman WJ](https://www.ncbi.nlm.nih.gov/pubmed/?term=Brokelman%20WJ%5BAuthor%5D&cauthor=true&cauthor_uid=26679173), [Gerhards MF](https://www.ncbi.nlm.nih.gov/pubmed/?term=Gerhards%20MF%5BAuthor%5D&cauthor=true&cauthor_uid=26679173), [van Geloven AA](https://www.ncbi.nlm.nih.gov/pubmed/?term=van%20Geloven%20AA%5BAuthor%5D&cauthor=true&cauthor_uid=26679173), [van Dieren S](https://www.ncbi.nlm.nih.gov/pubmed/?term=van%20Dieren%20S%5BAuthor%5D&cauthor=true&cauthor_uid=26679173), [Lange JF](https://www.ncbi.nlm.nih.gov/pubmed/?term=Lange%20JF%5BAuthor%5D&cauthor=true&cauthor_uid=26679173), [Bemelman WA](https://www.ncbi.nlm.nih.gov/pubmed/?term=Bemelman%20WA%5BAuthor%5D&cauthor=true&cauthor_uid=26679173). Acute laparoscopic and open sigmoidectomy for perforated diverticulitis: a propensity score-matched cohort. [Surg Endosc.](https://www.ncbi.nlm.nih.gov/pubmed/?term=Surg+Endosc.+2016+Sep%3B30(9)%3A3889-96) 2016;30(9):3889-96
6. [Letarte F](https://www.ncbi.nlm.nih.gov/pubmed/?term=Letarte%20F%5BAuthor%5D&cauthor=true&cauthor_uid=25457252), [Hallet J](https://www.ncbi.nlm.nih.gov/pubmed/?term=Hallet%20J%5BAuthor%5D&cauthor=true&cauthor_uid=25457252), [Drolet S](https://www.ncbi.nlm.nih.gov/pubmed/?term=Drolet%20S%5BAuthor%5D&cauthor=true&cauthor_uid=25457252), [Boulanger-Gobeil C](https://www.ncbi.nlm.nih.gov/pubmed/?term=Boulanger-Gobeil%20C%5BAuthor%5D&cauthor=true&cauthor_uid=25457252), [Bouchard A](https://www.ncbi.nlm.nih.gov/pubmed/?term=Bouchard%20A%5BAuthor%5D&cauthor=true&cauthor_uid=25457252), [Grégoire RC](https://www.ncbi.nlm.nih.gov/pubmed/?term=Gr%C3%A9goire%20RC%5BAuthor%5D&cauthor=true&cauthor_uid=25457252), [Gagné JP](https://www.ncbi.nlm.nih.gov/pubmed/?term=Gagn%C3%A9%20JP%5BAuthor%5D&cauthor=true&cauthor_uid=25457252), [Thibault C](https://www.ncbi.nlm.nih.gov/pubmed/?term=Thibault%20C%5BAuthor%5D&cauthor=true&cauthor_uid=25457252), [Bouchard P](https://www.ncbi.nlm.nih.gov/pubmed/?term=Bouchard%20P%5BAuthor%5D&cauthor=true&cauthor_uid=25457252). Laparoscopic versus open colonic resection for complicated diverticular disease in the emergency setting: a safe choice? A retrospective comparative cohort study. [Am J Surg.](https://www.ncbi.nlm.nih.gov/pubmed/?term=Am+J+Surg.+2015+Jun%3B209(6)%3A992-8) 2015;209(6):992-8
7. [Turley RS](https://www.ncbi.nlm.nih.gov/pubmed/?term=Turley%20RS%5BAuthor%5D&cauthor=true&cauthor_uid=23222283), [Barbas AS](https://www.ncbi.nlm.nih.gov/pubmed/?term=Barbas%20AS%5BAuthor%5D&cauthor=true&cauthor_uid=23222283), [Lidsky ME](https://www.ncbi.nlm.nih.gov/pubmed/?term=Lidsky%20ME%5BAuthor%5D&cauthor=true&cauthor_uid=23222283), [Mantyh CR](https://www.ncbi.nlm.nih.gov/pubmed/?term=Mantyh%20CR%5BAuthor%5D&cauthor=true&cauthor_uid=23222283), [Migaly J](https://www.ncbi.nlm.nih.gov/pubmed/?term=Migaly%20J%5BAuthor%5D&cauthor=true&cauthor_uid=23222283), [Scarborough JE](https://www.ncbi.nlm.nih.gov/pubmed/?term=Scarborough%20JE%5BAuthor%5D&cauthor=true&cauthor_uid=23222283). Laparoscopic versus open Hartmann procedure for the emergency treatment of diverticulitis: a propensity-matched analysis. [Dis Colon Rectum.](https://www.ncbi.nlm.nih.gov/pubmed/?term=Dis+Colon+Rectum.+2013+Jan%3B56(1)%3A72-82.) 2013;56(1):72-82
8. [Titu LV](https://www.ncbi.nlm.nih.gov/pubmed/?term=Titu%20LV%5BAuthor%5D&cauthor=true&cauthor_uid=18616737), [Zafar N](https://www.ncbi.nlm.nih.gov/pubmed/?term=Zafar%20N%5BAuthor%5D&cauthor=true&cauthor_uid=18616737), [Phillips SM](https://www.ncbi.nlm.nih.gov/pubmed/?term=Phillips%20SM%5BAuthor%5D&cauthor=true&cauthor_uid=18616737), [Greenslade GL](https://www.ncbi.nlm.nih.gov/pubmed/?term=Greenslade%20GL%5BAuthor%5D&cauthor=true&cauthor_uid=18616737), [Dixon AR](https://www.ncbi.nlm.nih.gov/pubmed/?term=Dixon%20AR%5BAuthor%5D&cauthor=true&cauthor_uid=18616737)Emergency laparoscopic surgery for complicated diverticular disease. [Colorectal Dis.](https://www.ncbi.nlm.nih.gov/pubmed/?term=Colorectal+Dis.+2009+May%3B11(4)%3A401-4) 2009;11(4):401-4
9. [White SI](https://www.ncbi.nlm.nih.gov/pubmed/?term=White%20SI%5BAuthor%5D&cauthor=true&cauthor_uid=20940603), [Frenkiel B](https://www.ncbi.nlm.nih.gov/pubmed/?term=Frenkiel%20B%5BAuthor%5D&cauthor=true&cauthor_uid=20940603), [Martin PJ](https://www.ncbi.nlm.nih.gov/pubmed/?term=Martin%20PJ%5BAuthor%5D&cauthor=true&cauthor_uid=20940603). A ten-year audit of perforated sigmoid diverticulitis: highlighting the outcomes of laparoscopic lavage. [Dis Colon Rectum.](https://www.ncbi.nlm.nih.gov/pubmed/?term=Dis+Colon+Rectum+2010%3B+53%3A+1537%E2%80%931541) 2010;53(11):1537-41

**Q5.3:** What is the optimal surgical strategy in the acute setting?

Statement:

In Hinchey 3 diverticulitis sigmoid resection with primary anastomosis with proximal diversion has similar mortality, lower morbidity and lower stoma rate at 12 months compared to Hartmann procedure with reversal.

LOE: Moderate

Recommendation:

In the appropriate clinical setting, we recommend consideration of sigmoid resection with primary anastomosis and proximal diversion over HP in patients with Hinchey 3/4 diverticulitis.

SOR: weak

Recommendation:
Hartmann’s procedure is the preferred operation for hemodynamically unstable patients with perforated diverticulitis.

LOE Low

SOR: strong for using

Statement: In unstable perforated diverticulitis damage control strategies (resection without anastomosis, temporary abdominal closure and second look) showed acceptable mortality and morbidity and lower stoma rates

LOE: Low

Recommendation:

We recommend in unstable patients with perforated diverticulitis damage control strategies (resection without anastomosis, temporary abdominal closure and second look) be considered.

SOR: strong

There is debate on the optimal surgery for perforated diverticulitis in the emergent setting. The patient’s clinical status is the most important factor to consider with regards to the type of operation performed. While ligation of the colonic perforation and diversion alone has fallen out of favor compared to primary anastomosis with or without a diverting ileostomy, recent data supports that in select patients, sigmoid resection with primary anastomosis has at least a similar mortality but a decreased morbidity compared to Hartmann’s procedure including lower permanent stomas, lower re-intervention rates and shorter hospital stays (Cirocchi 2013, Brideoux 2017, Oberkofler). HP is still a very commonly performed operation in this setting and has been advocated in the case of faecal peritonitis, septic shock, haemodynamic instability, chronic steroid therapy and poor baseline of the patient (Biondo 2012). Despite evidence to suggest similarities between patients undergoing HP and PA, there may be some unmeasured confounders biasing the conclusions to be drawn from comparisons between the two types of operations. Primary anastomosis may still be appropriate in some cases of limited faecal contamination whereas more widespread fecal contamination with primary anastomosis would not be recommended. It remains important to point out that Hartmann’s reversal is a challenging operation, with a higher morbidity and mortality compared to the reversal of a diverting ileostomy, with overall reversal morbidity of 44% (Vermeulen et al). In patients who are hemodynamically unstable, damage control surgeries are a consideration. Cirocchi et al defined as damage control both peritoneal lavage and resection without anastomosis, temporary closure and second look. Focusing only on the second group, the majority of surgeons insisted on limited resection of diseased colon leaving the colon stapled-off in situ without reconstruction. Other authors performed colonic resection with colostomy in all cases (HP) with temporary closure and second look. The most recent series (Sohn 2016), including only patients with severe peritonitis, reported a mortality rate of 11% and a stoma rate of 45%. Temporary abdominal closures may be underutilized in perforated diverticulitis and although limited, some evidence suggests that their use decreases the need for HP and increases PA and stoma.

**References**

1. [Cirocchi R](https://www.ncbi.nlm.nih.gov/pubmed/?term=Cirocchi%20R%5BAuthor%5D&cauthor=true&cauthor_uid=23242271), [Trastulli S](https://www.ncbi.nlm.nih.gov/pubmed/?term=Trastulli%20S%5BAuthor%5D&cauthor=true&cauthor_uid=23242271), [Desiderio J](https://www.ncbi.nlm.nih.gov/pubmed/?term=Desiderio%20J%5BAuthor%5D&cauthor=true&cauthor_uid=23242271), [Listorti C](https://www.ncbi.nlm.nih.gov/pubmed/?term=Listorti%20C%5BAuthor%5D&cauthor=true&cauthor_uid=23242271), [Boselli C](https://www.ncbi.nlm.nih.gov/pubmed/?term=Boselli%20C%5BAuthor%5D&cauthor=true&cauthor_uid=23242271), [Parisi A](https://www.ncbi.nlm.nih.gov/pubmed/?term=Parisi%20A%5BAuthor%5D&cauthor=true&cauthor_uid=23242271), [Noya G](https://www.ncbi.nlm.nih.gov/pubmed/?term=Noya%20G%5BAuthor%5D&cauthor=true&cauthor_uid=23242271), [Liu L](https://www.ncbi.nlm.nih.gov/pubmed/?term=Liu%20L%5BAuthor%5D&cauthor=true&cauthor_uid=23242271). Treatment of Hinchey stage III-IV diverticulitis: a systematic review and meta-analysis. [Int J Colorectal Dis.](https://www.ncbi.nlm.nih.gov/pubmed/?term=Int+J+Colorectal+Dis.+2013+Apr%3B28(4)%3A447-57) 2013;28(4):447-57
2. [Toro A](https://www.ncbi.nlm.nih.gov/pubmed/?term=Toro%20A%5BAuthor%5D&cauthor=true&cauthor_uid=23116833), [Mannino M](https://www.ncbi.nlm.nih.gov/pubmed/?term=Mannino%20M%5BAuthor%5D&cauthor=true&cauthor_uid=23116833), [Reale G](https://www.ncbi.nlm.nih.gov/pubmed/?term=Reale%20G%5BAuthor%5D&cauthor=true&cauthor_uid=23116833), [Cappello G](https://www.ncbi.nlm.nih.gov/pubmed/?term=Cappello%20G%5BAuthor%5D&cauthor=true&cauthor_uid=23116833), [Di Carlo I](https://www.ncbi.nlm.nih.gov/pubmed/?term=Di%20Carlo%20I%5BAuthor%5D&cauthor=true&cauthor_uid=23116833). Primary anastomosis vs Hartmann procedure in acute complicated diverticulitis. Evolution over the last twenty years. [Chirurgia (Bucur).](https://www.ncbi.nlm.nih.gov/pubmed/?term=Chirurgia+(Bucur).+2012+Sep-Oct%3B107(5)%3A598-604.) 2012;107(5):598-604
3. [Biondo S](https://www.ncbi.nlm.nih.gov/pubmed/?term=Biondo%20S%5BAuthor%5D&cauthor=true&cauthor_uid=21848896), [Lopez Borao J](https://www.ncbi.nlm.nih.gov/pubmed/?term=Lopez%20Borao%20J%5BAuthor%5D&cauthor=true&cauthor_uid=21848896), [Millan M](https://www.ncbi.nlm.nih.gov/pubmed/?term=Millan%20M%5BAuthor%5D&cauthor=true&cauthor_uid=21848896), [Kreisler E](https://www.ncbi.nlm.nih.gov/pubmed/?term=Kreisler%20E%5BAuthor%5D&cauthor=true&cauthor_uid=21848896), [Jaurrieta E](https://www.ncbi.nlm.nih.gov/pubmed/?term=Jaurrieta%20E%5BAuthor%5D&cauthor=true&cauthor_uid=21848896). Current status of the treatment of acute colonic diverticulitis: a systematic review. [Colorectal Dis.](https://www.ncbi.nlm.nih.gov/pubmed/?term=Colorectal+Dis.+2012+Jan%3B14(1)%3Ae1-e11) 2012;14(1):e1-e11
4. [Binda GA](https://www.ncbi.nlm.nih.gov/pubmed/?term=Binda%20GA%5BAuthor%5D&cauthor=true&cauthor_uid=22672447), [Karas JR](https://www.ncbi.nlm.nih.gov/pubmed/?term=Karas%20JR%5BAuthor%5D&cauthor=true&cauthor_uid=22672447), [Serventi A](https://www.ncbi.nlm.nih.gov/pubmed/?term=Serventi%20A%5BAuthor%5D&cauthor=true&cauthor_uid=22672447), [Sokmen S](https://www.ncbi.nlm.nih.gov/pubmed/?term=Sokmen%20S%5BAuthor%5D&cauthor=true&cauthor_uid=22672447), [Amato A](https://www.ncbi.nlm.nih.gov/pubmed/?term=Amato%20A%5BAuthor%5D&cauthor=true&cauthor_uid=22672447), [Hydo L](https://www.ncbi.nlm.nih.gov/pubmed/?term=Hydo%20L%5BAuthor%5D&cauthor=true&cauthor_uid=22672447), [Bergamaschi R](https://www.ncbi.nlm.nih.gov/pubmed/?term=Bergamaschi%20R%5BAuthor%5D&cauthor=true&cauthor_uid=22672447); [Study Group on Diverticulitis](https://www.ncbi.nlm.nih.gov/pubmed/?term=Study%20Group%20on%20Diverticulitis%5BCorporate%20Author%5D). Primary anastomosis vs nonrestorative resection for perforated diverticulitis with peritonitis: a prematurely terminated randomized controlled trial. [Colorectal Dis](https://www.ncbi.nlm.nih.gov/pubmed/?term=Colorectal+Dis.+2012+Nov%3B14(11)%3A1403-10) 2012;14(11):1403-10
5. [Oberkofler CE](https://www.ncbi.nlm.nih.gov/pubmed/?term=Oberkofler%20CE%5BAuthor%5D&cauthor=true&cauthor_uid=23095627), [Rickenbacher A](https://www.ncbi.nlm.nih.gov/pubmed/?term=Rickenbacher%20A%5BAuthor%5D&cauthor=true&cauthor_uid=23095627), [Raptis DA](https://www.ncbi.nlm.nih.gov/pubmed/?term=Raptis%20DA%5BAuthor%5D&cauthor=true&cauthor_uid=23095627), [Lehmann K](https://www.ncbi.nlm.nih.gov/pubmed/?term=Lehmann%20K%5BAuthor%5D&cauthor=true&cauthor_uid=23095627), [Villiger P](https://www.ncbi.nlm.nih.gov/pubmed/?term=Villiger%20P%5BAuthor%5D&cauthor=true&cauthor_uid=23095627), [Buchli C](https://www.ncbi.nlm.nih.gov/pubmed/?term=Buchli%20C%5BAuthor%5D&cauthor=true&cauthor_uid=23095627), [Grieder F](https://www.ncbi.nlm.nih.gov/pubmed/?term=Grieder%20F%5BAuthor%5D&cauthor=true&cauthor_uid=23095627), [Gelpke H](https://www.ncbi.nlm.nih.gov/pubmed/?term=Gelpke%20H%5BAuthor%5D&cauthor=true&cauthor_uid=23095627), [Decurtins M](https://www.ncbi.nlm.nih.gov/pubmed/?term=Decurtins%20M%5BAuthor%5D&cauthor=true&cauthor_uid=23095627), [Tempia-Caliera AA](https://www.ncbi.nlm.nih.gov/pubmed/?term=Tempia-Caliera%20AA%5BAuthor%5D&cauthor=true&cauthor_uid=23095627), [Demartines N](https://www.ncbi.nlm.nih.gov/pubmed/?term=Demartines%20N%5BAuthor%5D&cauthor=true&cauthor_uid=23095627), [Hahnloser D](https://www.ncbi.nlm.nih.gov/pubmed/?term=Hahnloser%20D%5BAuthor%5D&cauthor=true&cauthor_uid=23095627), [Clavien PA](https://www.ncbi.nlm.nih.gov/pubmed/?term=Clavien%20PA%5BAuthor%5D&cauthor=true&cauthor_uid=23095627), [Breitenstein S](https://www.ncbi.nlm.nih.gov/pubmed/?term=Breitenstein%20S%5BAuthor%5D&cauthor=true&cauthor_uid=23095627). A multicenter randomized clinical trial of primary anastomosis or Hartmann's procedure for perforated left colonic diverticulitis with purulent or fecal peritonitis. [Ann Surg.](https://www.ncbi.nlm.nih.gov/pubmed/?term=Ann+Surg+2012%3B256%3A+819%E2%80%93827) 2012;256(5):819-26
6. [Jafferji MS](https://www.ncbi.nlm.nih.gov/pubmed/?term=Jafferji%20MS%5BAuthor%5D&cauthor=true&cauthor_uid=24755189), [Hyman N](https://www.ncbi.nlm.nih.gov/pubmed/?term=Hyman%20N%5BAuthor%5D&cauthor=true&cauthor_uid=24755189). Surgeon, not disease severity, often determines the operation for acute complicated diverticulitis. [J Am Coll Surg](https://www.ncbi.nlm.nih.gov/pubmed/?term=J+Am+Coll+Surg.+2014+Jun%3B218(6)%3A1156-61) 2014;218(6):1156-61
7. [Boyce SA](https://www.ncbi.nlm.nih.gov/pubmed/?term=Boyce%20SA%5BAuthor%5D&cauthor=true&cauthor_uid=22966859), [Bartolo DC](https://www.ncbi.nlm.nih.gov/pubmed/?term=Bartolo%20DC%5BAuthor%5D&cauthor=true&cauthor_uid=22966859), [Paterson HM](https://www.ncbi.nlm.nih.gov/pubmed/?term=Paterson%20HM%5BAuthor%5D&cauthor=true&cauthor_uid=22966859); [Edinburgh Coloproctology Unit](https://www.ncbi.nlm.nih.gov/pubmed/?term=Edinburgh%20Coloproctology%20Unit%5BCorporate%20Author%5D). Subspecialist emergency management of diverticulitis is associated with reduced mortality and fewer stomas. [Colorectal Dis.](https://www.ncbi.nlm.nih.gov/pubmed/?term=Colorectal+Dis.+2013+Apr%3B15(4)%3A442-7) 2013;15(4):442-7
8. [Liang S](https://www.ncbi.nlm.nih.gov/pubmed/?term=Liang%20S%5BAuthor%5D&cauthor=true&cauthor_uid=22543992), [Russek K](https://www.ncbi.nlm.nih.gov/pubmed/?term=Russek%20K%5BAuthor%5D&cauthor=true&cauthor_uid=22543992), [Franklin ME Jr](https://www.ncbi.nlm.nih.gov/pubmed/?term=Franklin%20ME%20Jr%5BAuthor%5D&cauthor=true&cauthor_uid=22543992). Damage control strategy for the management of perforated diverticulitis with generalized peritonitis: laparoscopic lavage and drainage vs. laparoscopic Hartmann's procedure. [Surg Endosc.](https://www.ncbi.nlm.nih.gov/pubmed/?term=Surg+Endosc.+2012+Oct%3B26(10)%3A2835-42) 2012;26(10):2835-42
9. [Masoomi H](https://www.ncbi.nlm.nih.gov/pubmed/?term=Masoomi%20H%5BAuthor%5D&cauthor=true&cauthor_uid=23075540), [Stamos MJ](https://www.ncbi.nlm.nih.gov/pubmed/?term=Stamos%20MJ%5BAuthor%5D&cauthor=true&cauthor_uid=23075540), [Carmichael JC](https://www.ncbi.nlm.nih.gov/pubmed/?term=Carmichael%20JC%5BAuthor%5D&cauthor=true&cauthor_uid=23075540), [Nguyen B](https://www.ncbi.nlm.nih.gov/pubmed/?term=Nguyen%20B%5BAuthor%5D&cauthor=true&cauthor_uid=23075540), [Buchberg B](https://www.ncbi.nlm.nih.gov/pubmed/?term=Buchberg%20B%5BAuthor%5D&cauthor=true&cauthor_uid=23075540), [Mills S](https://www.ncbi.nlm.nih.gov/pubmed/?term=Mills%20S%5BAuthor%5D&cauthor=true&cauthor_uid=23075540). Does primary anastomosis with diversion have any advantages over Hartmann's procedure in acute diverticulitis? [Dig Surg.](https://www.ncbi.nlm.nih.gov/pubmed/?term=Dig+Surg.+2012%3B29(4)%3A315-20) 2012;29(4):315-20
10. Tabbara M, Velmahos GC, Butt MU, Chang Y, Spaniolas K, Demoya M, King DR, Alam HB. [Missed opportunities for primary repair in complicated acute diverticulitis.](https://www.ncbi.nlm.nih.gov/pubmed/20378139) Surgery 2010;148(5):919-24
11. [Pasternak I](https://www.ncbi.nlm.nih.gov/pubmed/?term=Pasternak%20I%5BAuthor%5D&cauthor=true&cauthor_uid=20091171), [Dietrich M](https://www.ncbi.nlm.nih.gov/pubmed/?term=Dietrich%20M%5BAuthor%5D&cauthor=true&cauthor_uid=20091171), [Woodman R](https://www.ncbi.nlm.nih.gov/pubmed/?term=Woodman%20R%5BAuthor%5D&cauthor=true&cauthor_uid=20091171), [Metzger U](https://www.ncbi.nlm.nih.gov/pubmed/?term=Metzger%20U%5BAuthor%5D&cauthor=true&cauthor_uid=20091171), [Wattchow DA](https://www.ncbi.nlm.nih.gov/pubmed/?term=Wattchow%20DA%5BAuthor%5D&cauthor=true&cauthor_uid=20091171), [Zingg U](https://www.ncbi.nlm.nih.gov/pubmed/?term=Zingg%20U%5BAuthor%5D&cauthor=true&cauthor_uid=20091171). Use of severity classification systems in the surgical decision-making process in emergency laparotomy for perforated diverticulitis. [Int J Colorectal Dis.](https://www.ncbi.nlm.nih.gov/pubmed/?term=Int+J+Colorectal+Dis.+2010+Apr%3B25(4)%3A463-70) 2010;25(4):463-70
12. [Zingg U](https://www.ncbi.nlm.nih.gov/pubmed/?term=Zingg%20U%5BAuthor%5D&cauthor=true&cauthor_uid=19175638), [Pasternak I](https://www.ncbi.nlm.nih.gov/pubmed/?term=Pasternak%20I%5BAuthor%5D&cauthor=true&cauthor_uid=19175638), [Dietrich M](https://www.ncbi.nlm.nih.gov/pubmed/?term=Dietrich%20M%5BAuthor%5D&cauthor=true&cauthor_uid=19175638), [Seifert B](https://www.ncbi.nlm.nih.gov/pubmed/?term=Seifert%20B%5BAuthor%5D&cauthor=true&cauthor_uid=19175638), [Oertli D](https://www.ncbi.nlm.nih.gov/pubmed/?term=Oertli%20D%5BAuthor%5D&cauthor=true&cauthor_uid=19175638), [Metzger U](https://www.ncbi.nlm.nih.gov/pubmed/?term=Metzger%20U%5BAuthor%5D&cauthor=true&cauthor_uid=19175638). Primary anastomosis vs Hartmann's procedure in patients undergoing emergency left colectomy for perforated diverticulitis. [Colorectal Dis.](https://www.ncbi.nlm.nih.gov/pubmed/?term=Colorectal+Dis.+2010+Jan%3B12(1)%3A54-60) 2010;12(1):54-60
13. [Di Stefano G](https://www.ncbi.nlm.nih.gov/pubmed/?term=Di%20Stefano%20G%5BAuthor%5D&cauthor=true&cauthor_uid=19735614), [Zanghì GN](https://www.ncbi.nlm.nih.gov/pubmed/?term=Zangh%C3%AC%20GN%5BAuthor%5D&cauthor=true&cauthor_uid=19735614), [Biondi A](https://www.ncbi.nlm.nih.gov/pubmed/?term=Biondi%20A%5BAuthor%5D&cauthor=true&cauthor_uid=19735614), [Benfatto G](https://www.ncbi.nlm.nih.gov/pubmed/?term=Benfatto%20G%5BAuthor%5D&cauthor=true&cauthor_uid=19735614), [Basile F](https://www.ncbi.nlm.nih.gov/pubmed/?term=Basile%20F%5BAuthor%5D&cauthor=true&cauthor_uid=19735614). Surgical treatment of acute complicated diverticulitis. [G Chir.](https://www.ncbi.nlm.nih.gov/pubmed/?term=G+Chir.+2009+Aug-Sep%3B30(8-9)%3A355-8) 2009;30(8-9):355-8.
14. [Tadlock MD](https://www.ncbi.nlm.nih.gov/pubmed/?term=Tadlock%20MD%5BAuthor%5D&cauthor=true&cauthor_uid=23694862), [Karamanos E](https://www.ncbi.nlm.nih.gov/pubmed/?term=Karamanos%20E%5BAuthor%5D&cauthor=true&cauthor_uid=23694862), [Skiada D](https://www.ncbi.nlm.nih.gov/pubmed/?term=Skiada%20D%5BAuthor%5D&cauthor=true&cauthor_uid=23694862), [Inaba K](https://www.ncbi.nlm.nih.gov/pubmed/?term=Inaba%20K%5BAuthor%5D&cauthor=true&cauthor_uid=23694862), [Talving P](https://www.ncbi.nlm.nih.gov/pubmed/?term=Talving%20P%5BAuthor%5D&cauthor=true&cauthor_uid=23694862), [Senagore A](https://www.ncbi.nlm.nih.gov/pubmed/?term=Senagore%20A%5BAuthor%5D&cauthor=true&cauthor_uid=23694862), [Demetriades D](https://www.ncbi.nlm.nih.gov/pubmed/?term=Demetriades%20D%5BAuthor%5D&cauthor=true&cauthor_uid=23694862). Emergency surgery for acute diverticulitis: which operation? A National Surgical Quality Improvement Program study. [J Trauma Acute Care Surg.](https://www.ncbi.nlm.nih.gov/pubmed/?term=J+Trauma+Acute+Care+Surg+Volume+74%2C+Number+6+1385) 2013;74(6):1385-91
15. [Vermeulen J](https://www.ncbi.nlm.nih.gov/pubmed/?term=Vermeulen%20J%5BAuthor%5D&cauthor=true&cauthor_uid=18727727), [Coene PP](https://www.ncbi.nlm.nih.gov/pubmed/?term=Coene%20PP%5BAuthor%5D&cauthor=true&cauthor_uid=18727727), [Van Hout NM](https://www.ncbi.nlm.nih.gov/pubmed/?term=Van%20Hout%20NM%5BAuthor%5D&cauthor=true&cauthor_uid=18727727), [van der Harst E](https://www.ncbi.nlm.nih.gov/pubmed/?term=van%20der%20Harst%20E%5BAuthor%5D&cauthor=true&cauthor_uid=18727727), [Gosselink MP](https://www.ncbi.nlm.nih.gov/pubmed/?term=Gosselink%20MP%5BAuthor%5D&cauthor=true&cauthor_uid=18727727), [Mannaerts GH](https://www.ncbi.nlm.nih.gov/pubmed/?term=Mannaerts%20GH%5BAuthor%5D&cauthor=true&cauthor_uid=18727727), [Weidema WF](https://www.ncbi.nlm.nih.gov/pubmed/?term=Weidema%20WF%5BAuthor%5D&cauthor=true&cauthor_uid=18727727), [Lange JF](https://www.ncbi.nlm.nih.gov/pubmed/?term=Lange%20JF%5BAuthor%5D&cauthor=true&cauthor_uid=18727727). Restoration of bowel continuity after surgery for acute perforated diverticulitis: should Hartmann's procedure be considered a one-stage procedure? [Colorectal Dis.](https://www.ncbi.nlm.nih.gov/pubmed/?term=Colorectal+Dis.+2009+Jul%3B11(6)%3A619-24.) 2009;11(6):619-24
16. [Isbister WH](https://www.ncbi.nlm.nih.gov/pubmed/?term=Isbister%20WH%5BAuthor%5D&cauthor=true&cauthor_uid=19861778). Diverticular disease: an experience at King Faisal specialist hospital. [Saudi J Gastroenterol.](https://www.ncbi.nlm.nih.gov/pubmed/?term=Journal+Article+India+Saudi+J+Gastroenterol.+2001+Sep%3B7(3)%3A109-12.) 2001;7(3):109-12.
17. [Regenet N](https://www.ncbi.nlm.nih.gov/pubmed/?term=Regenet%20N%5BAuthor%5D&cauthor=true&cauthor_uid=12910361), [Pessaux P](https://www.ncbi.nlm.nih.gov/pubmed/?term=Pessaux%20P%5BAuthor%5D&cauthor=true&cauthor_uid=12910361), [Hennekinne S](https://www.ncbi.nlm.nih.gov/pubmed/?term=Hennekinne%20S%5BAuthor%5D&cauthor=true&cauthor_uid=12910361), [Lermite E](https://www.ncbi.nlm.nih.gov/pubmed/?term=Lermite%20E%5BAuthor%5D&cauthor=true&cauthor_uid=12910361), [Tuech JJ](https://www.ncbi.nlm.nih.gov/pubmed/?term=Tuech%20JJ%5BAuthor%5D&cauthor=true&cauthor_uid=12910361), [Brehant O](https://www.ncbi.nlm.nih.gov/pubmed/?term=Brehant%20O%5BAuthor%5D&cauthor=true&cauthor_uid=12910361), [Arnaud JP](https://www.ncbi.nlm.nih.gov/pubmed/?term=Arnaud%20JP%5BAuthor%5D&cauthor=true&cauthor_uid=12910361). Primary anastomosis after intraoperative colonic lavage vs. Hartmann's procedure in generalized peritonitis complicating diverticular disease of the colon. [Int J Colorectal Dis.](https://www.ncbi.nlm.nih.gov/pubmed/?term=Int+J+Colorectal+Dis.+2003+Nov%3B18(6)%3A503-7) 2003;18(6):503-7
18. Zorcolo L, Covotta L, Carlomagno N, Bartolo DC. [Safety of primary anastomosis in emergency colo-rectal surgery.](https://www.ncbi.nlm.nih.gov/pubmed/12780890) Colorectal Dis. 2003;5(3):262-9.
19. [Chouillard E](https://www.ncbi.nlm.nih.gov/pubmed/?term=Chouillard%20E%5BAuthor%5D&cauthor=true&cauthor_uid=17294319), [Maggiori L](https://www.ncbi.nlm.nih.gov/pubmed/?term=Maggiori%20L%5BAuthor%5D&cauthor=true&cauthor_uid=17294319), [Ata T](https://www.ncbi.nlm.nih.gov/pubmed/?term=Ata%20T%5BAuthor%5D&cauthor=true&cauthor_uid=17294319), [Jarbaoui S](https://www.ncbi.nlm.nih.gov/pubmed/?term=Jarbaoui%20S%5BAuthor%5D&cauthor=true&cauthor_uid=17294319), [Rivkine E](https://www.ncbi.nlm.nih.gov/pubmed/?term=Rivkine%20E%5BAuthor%5D&cauthor=true&cauthor_uid=17294319), [Benhaim L](https://www.ncbi.nlm.nih.gov/pubmed/?term=Benhaim%20L%5BAuthor%5D&cauthor=true&cauthor_uid=17294319), [Ghiles E](https://www.ncbi.nlm.nih.gov/pubmed/?term=Ghiles%20E%5BAuthor%5D&cauthor=true&cauthor_uid=17294319), [Etienne JC](https://www.ncbi.nlm.nih.gov/pubmed/?term=Etienne%20JC%5BAuthor%5D&cauthor=true&cauthor_uid=17294319), [Fingerhut A](https://www.ncbi.nlm.nih.gov/pubmed/?term=Fingerhut%20A%5BAuthor%5D&cauthor=true&cauthor_uid=17294319). Laparoscopic two-stage left colonic resection for patients with peritonitis caused by acute diverticulitis. [Dis Colon Rectum.](https://www.ncbi.nlm.nih.gov/pubmed/?term=Dis+Colon+Rectum.+2007+Aug%3B50(8)%3A1157-63) 2007;50(8):1157-63.
20. [Zeitoun G](https://www.ncbi.nlm.nih.gov/pubmed/?term=Zeitoun%20G%5BAuthor%5D&cauthor=true&cauthor_uid=11044163), [Laurent A](https://www.ncbi.nlm.nih.gov/pubmed/?term=Laurent%20A%5BAuthor%5D&cauthor=true&cauthor_uid=11044163), [Rouffet F](https://www.ncbi.nlm.nih.gov/pubmed/?term=Rouffet%20F%5BAuthor%5D&cauthor=true&cauthor_uid=11044163), [Hay J](https://www.ncbi.nlm.nih.gov/pubmed/?term=Hay%20J%5BAuthor%5D&cauthor=true&cauthor_uid=11044163), [Fingerhut A](https://www.ncbi.nlm.nih.gov/pubmed/?term=Fingerhut%20A%5BAuthor%5D&cauthor=true&cauthor_uid=11044163), [Paquet J](https://www.ncbi.nlm.nih.gov/pubmed/?term=Paquet%20J%5BAuthor%5D&cauthor=true&cauthor_uid=11044163), [Peillon C](https://www.ncbi.nlm.nih.gov/pubmed/?term=Peillon%20C%5BAuthor%5D&cauthor=true&cauthor_uid=11044163), [Research TF](https://www.ncbi.nlm.nih.gov/pubmed/?term=Research%20TF%5BAuthor%5D&cauthor=true&cauthor_uid=11044163). Multicentre, randomized clinical trial of primary versus secondary sigmoid resection in generalized peritonitis complicating sigmoid diverticulitis. [Br J Surg](https://www.ncbi.nlm.nih.gov/pubmed/?term=Br+J+Surg.+2000+Oct%3B87(10)%3A1366-74.) 2000;87(10):1366-74.
21. [Jiménez Fuertes M](https://www.ncbi.nlm.nih.gov/pubmed/?term=Jim%C3%A9nez%20Fuertes%20M%5BAuthor%5D&cauthor=true&cauthor_uid=22402970), [Costa Navarro D](https://www.ncbi.nlm.nih.gov/pubmed/?term=Costa%20Navarro%20D%5BAuthor%5D&cauthor=true&cauthor_uid=22402970). Resection and primary anastomosis without diverting ileostomy for left colon emergencies: is it a safe procedure? [World J Surg.](https://www.ncbi.nlm.nih.gov/pubmed/?term=World+J+Surg.+2012+May%3B36(5)%3A1148-53) 2012;36(5):1148-53
22. [Trillo C](https://www.ncbi.nlm.nih.gov/pubmed/?term=Trillo%20C%5BAuthor%5D&cauthor=true&cauthor_uid=9731807), [Paris MF](https://www.ncbi.nlm.nih.gov/pubmed/?term=Paris%20MF%5BAuthor%5D&cauthor=true&cauthor_uid=9731807), [Brennan JT](https://www.ncbi.nlm.nih.gov/pubmed/?term=Brennan%20JT%5BAuthor%5D&cauthor=true&cauthor_uid=9731807). Primary anastomosis in the treatment of acute disease of the unprepared left colon. [Am Surg.](https://www.ncbi.nlm.nih.gov/pubmed/?term=The+American+Surgeon%2C+64(9)%2C+821-4) 1998;64(9):821-4
23. [Hoemke M](https://www.ncbi.nlm.nih.gov/pubmed/?term=Hoemke%20M%5BAuthor%5D&cauthor=true&cauthor_uid=10567805), [Treckmann J](https://www.ncbi.nlm.nih.gov/pubmed/?term=Treckmann%20J%5BAuthor%5D&cauthor=true&cauthor_uid=10567805), [Schmitz R](https://www.ncbi.nlm.nih.gov/pubmed/?term=Schmitz%20R%5BAuthor%5D&cauthor=true&cauthor_uid=10567805), [Shah S](https://www.ncbi.nlm.nih.gov/pubmed/?term=Shah%20S%5BAuthor%5D&cauthor=true&cauthor_uid=10567805). Complicated diverticulitis of the sigmoid: a prospective study concerning primary resection with secure primary anastomosis. [Dig Surg.](https://www.ncbi.nlm.nih.gov/pubmed/?term=Dig+Surg.+1999%3B16(5)%3A420-4) 1999;16(5):420-4
24. [Umbach TW](https://www.ncbi.nlm.nih.gov/pubmed/?term=Umbach%20TW%5BAuthor%5D&cauthor=true&cauthor_uid=10515537), [Dorazio RA](https://www.ncbi.nlm.nih.gov/pubmed/?term=Dorazio%20RA%5BAuthor%5D&cauthor=true&cauthor_uid=10515537). Primary resection and anastomosis for perforated left colon lesions. [Am Surg.](https://www.ncbi.nlm.nih.gov/pubmed/?term=Am+Surg.+1999+Oct%3B65(10)%3A931-3) 1999 ;65(10):931-3.
25. [Schilling MK](https://www.ncbi.nlm.nih.gov/pubmed/?term=Schilling%20MK%5BAuthor%5D&cauthor=true&cauthor_uid=11357032), [Maurer CA](https://www.ncbi.nlm.nih.gov/pubmed/?term=Maurer%20CA%5BAuthor%5D&cauthor=true&cauthor_uid=11357032), [Kollmar O](https://www.ncbi.nlm.nih.gov/pubmed/?term=Kollmar%20O%5BAuthor%5D&cauthor=true&cauthor_uid=11357032), [Büchler MW](https://www.ncbi.nlm.nih.gov/pubmed/?term=B%C3%BCchler%20MW%5BAuthor%5D&cauthor=true&cauthor_uid=11357032). Primary vs. secondary anastomosis after sigmoid colon resection for perforated diverticulitis (Hinchey Stage III and IV): a prospective outcome and cost analysis. [Dis Colon Rectum.](https://www.ncbi.nlm.nih.gov/pubmed/?term=Dis+Colon+Rectum.+2001+May%3B44(5)%3A699-703%3B+discussion+703-5.) 2001;44(5):699-703
26. [Gooszen AW](https://www.ncbi.nlm.nih.gov/pubmed/?term=Gooszen%20AW%5BAuthor%5D&cauthor=true&cauthor_uid=11213818), [Gooszen HG](https://www.ncbi.nlm.nih.gov/pubmed/?term=Gooszen%20HG%5BAuthor%5D&cauthor=true&cauthor_uid=11213818), [Veerman W](https://www.ncbi.nlm.nih.gov/pubmed/?term=Veerman%20W%5BAuthor%5D&cauthor=true&cauthor_uid=11213818), [Van Dongen VM](https://www.ncbi.nlm.nih.gov/pubmed/?term=Van%20Dongen%20VM%5BAuthor%5D&cauthor=true&cauthor_uid=11213818), [Hermans J](https://www.ncbi.nlm.nih.gov/pubmed/?term=Hermans%20J%5BAuthor%5D&cauthor=true&cauthor_uid=11213818), [Klien Kranenbarg E](https://www.ncbi.nlm.nih.gov/pubmed/?term=Klien%20Kranenbarg%20E%5BAuthor%5D&cauthor=true&cauthor_uid=11213818), [Tollenaar RA](https://www.ncbi.nlm.nih.gov/pubmed/?term=Tollenaar%20RA%5BAuthor%5D&cauthor=true&cauthor_uid=11213818). Operative treatment of acute complications of diverticular disease: primary or secondary anastomosis after sigmoid resection.
27. [Eur J Surg.](https://www.ncbi.nlm.nih.gov/pubmed/?term=Eur+J+Surg.+2001+Jan%3B167(1)%3A35-9.) 2001;167(1):35-9.
28. [Maggard MA](https://www.ncbi.nlm.nih.gov/pubmed/?term=Maggard%20MA%5BAuthor%5D&cauthor=true&cauthor_uid=11768827), [Chandler CF](https://www.ncbi.nlm.nih.gov/pubmed/?term=Chandler%20CF%5BAuthor%5D&cauthor=true&cauthor_uid=11768827), [Schmit PJ](https://www.ncbi.nlm.nih.gov/pubmed/?term=Schmit%20PJ%5BAuthor%5D&cauthor=true&cauthor_uid=11768827), [Bennion RS](https://www.ncbi.nlm.nih.gov/pubmed/?term=Bennion%20RS%5BAuthor%5D&cauthor=true&cauthor_uid=11768827), [Hines OJ](https://www.ncbi.nlm.nih.gov/pubmed/?term=Hines%20OJ%5BAuthor%5D&cauthor=true&cauthor_uid=11768827), [Thompson JE](https://www.ncbi.nlm.nih.gov/pubmed/?term=Thompson%20JE%5BAuthor%5D&cauthor=true&cauthor_uid=11768827). Surgical diverticulitis: treatment options. [Am Surg.](https://www.ncbi.nlm.nih.gov/pubmed/?term=Am+Surg.+2001+Dec%3B67(12)%3A1185-9.) 2001;67(12):1185-9.
29. [Biondo S](https://www.ncbi.nlm.nih.gov/pubmed/?term=Biondo%20S%5BAuthor%5D&cauthor=true&cauthor_uid=12190679), [Parés D](https://www.ncbi.nlm.nih.gov/pubmed/?term=Par%C3%A9s%20D%5BAuthor%5D&cauthor=true&cauthor_uid=12190679), [Martí Ragué J](https://www.ncbi.nlm.nih.gov/pubmed/?term=Mart%C3%AD%20Ragu%C3%A9%20J%5BAuthor%5D&cauthor=true&cauthor_uid=12190679), [Kreisler E](https://www.ncbi.nlm.nih.gov/pubmed/?term=Kreisler%20E%5BAuthor%5D&cauthor=true&cauthor_uid=12190679), [Fraccalvieri D](https://www.ncbi.nlm.nih.gov/pubmed/?term=Fraccalvieri%20D%5BAuthor%5D&cauthor=true&cauthor_uid=12190679), [Jaurrieta E](https://www.ncbi.nlm.nih.gov/pubmed/?term=Jaurrieta%20E%5BAuthor%5D&cauthor=true&cauthor_uid=12190679). Acute colonic diverticulitis in patients under 50 years of age. [Br J Surg.](https://www.ncbi.nlm.nih.gov/pubmed/?term=Br+J+Surg.+2002+Sep%3B89(9)%3A1137-41.) 2002;89(9):1137-41.
30. [Blair NP](https://www.ncbi.nlm.nih.gov/pubmed/?term=Blair%20NP%5BAuthor%5D&cauthor=true&cauthor_uid=12034385), [Germann E](https://www.ncbi.nlm.nih.gov/pubmed/?term=Germann%20E%5BAuthor%5D&cauthor=true&cauthor_uid=12034385). Surgical management of acute sigmoid diverticulitis. [Am J Surg.](https://www.ncbi.nlm.nih.gov/pubmed/?term=Am+J+Surg.+2002+May%3B183(5)%3A525-8.) 2002;183(5):525-8.
31. [Richter S](https://www.ncbi.nlm.nih.gov/pubmed/?term=Richter%20S%5BAuthor%5D&cauthor=true&cauthor_uid=16736332), [Lindemann W](https://www.ncbi.nlm.nih.gov/pubmed/?term=Lindemann%20W%5BAuthor%5D&cauthor=true&cauthor_uid=16736332), [Kollmar O](https://www.ncbi.nlm.nih.gov/pubmed/?term=Kollmar%20O%5BAuthor%5D&cauthor=true&cauthor_uid=16736332), [Pistorius GA](https://www.ncbi.nlm.nih.gov/pubmed/?term=Pistorius%20GA%5BAuthor%5D&cauthor=true&cauthor_uid=16736332), [Maurer CA](https://www.ncbi.nlm.nih.gov/pubmed/?term=Maurer%20CA%5BAuthor%5D&cauthor=true&cauthor_uid=16736332), [Schilling MK](https://www.ncbi.nlm.nih.gov/pubmed/?term=Schilling%20MK%5BAuthor%5D&cauthor=true&cauthor_uid=16736332). One-stage sigmoid colon resection for perforated sigmoid diverticulitis (Hinchey stages III and IV). [World J Surg.](https://www.ncbi.nlm.nih.gov/pubmed/?term=World+J+Surg.+2006+Jun%3B30(6)%3A1027-32) 2006;30(6):1027-32.
32. [Dumont F](https://www.ncbi.nlm.nih.gov/pubmed/?term=Dumont%20F%5BAuthor%5D&cauthor=true&cauthor_uid=15982629), [Vibert E](https://www.ncbi.nlm.nih.gov/pubmed/?term=Vibert%20E%5BAuthor%5D&cauthor=true&cauthor_uid=15982629), [Duval H](https://www.ncbi.nlm.nih.gov/pubmed/?term=Duval%20H%5BAuthor%5D&cauthor=true&cauthor_uid=15982629), [Manaouil D](https://www.ncbi.nlm.nih.gov/pubmed/?term=Manaouil%20D%5BAuthor%5D&cauthor=true&cauthor_uid=15982629), [Sredic A](https://www.ncbi.nlm.nih.gov/pubmed/?term=Sredic%20A%5BAuthor%5D&cauthor=true&cauthor_uid=15982629), [Alfahel N](https://www.ncbi.nlm.nih.gov/pubmed/?term=Alfahel%20N%5BAuthor%5D&cauthor=true&cauthor_uid=15982629), [Mauvais F](https://www.ncbi.nlm.nih.gov/pubmed/?term=Mauvais%20F%5BAuthor%5D&cauthor=true&cauthor_uid=15982629), [De Fresnoy H](https://www.ncbi.nlm.nih.gov/pubmed/?term=De%20Fresnoy%20H%5BAuthor%5D&cauthor=true&cauthor_uid=15982629), [Rudant J](https://www.ncbi.nlm.nih.gov/pubmed/?term=Rudant%20J%5BAuthor%5D&cauthor=true&cauthor_uid=15982629), [Katsahian S](https://www.ncbi.nlm.nih.gov/pubmed/?term=Katsahian%20S%5BAuthor%5D&cauthor=true&cauthor_uid=15982629), [Riboulot M](https://www.ncbi.nlm.nih.gov/pubmed/?term=Riboulot%20M%5BAuthor%5D&cauthor=true&cauthor_uid=15982629), [Galy C](https://www.ncbi.nlm.nih.gov/pubmed/?term=Galy%20C%5BAuthor%5D&cauthor=true&cauthor_uid=15982629), [Verhaeghe P](https://www.ncbi.nlm.nih.gov/pubmed/?term=Verhaeghe%20P%5BAuthor%5D&cauthor=true&cauthor_uid=15982629), [Dupont H](https://www.ncbi.nlm.nih.gov/pubmed/?term=Dupont%20H%5BAuthor%5D&cauthor=true&cauthor_uid=15982629), [Regimbeau JM](https://www.ncbi.nlm.nih.gov/pubmed/?term=Regimbeau%20JM%5BAuthor%5D&cauthor=true&cauthor_uid=15982629). Morbi-mortality after Hartmann procedure for peritonitis complicating sigmoid diverticulitis. A retrospective analysis of 85 cases**.** [Ann Chir.](https://www.ncbi.nlm.nih.gov/pubmed/?term=Ann+Chir.+2005+Jul-Aug%3B130(6-7)%3A391-9.) 2005;130(6-7):391-9.
33. [Seah DW](https://www.ncbi.nlm.nih.gov/pubmed/?term=Seah%20DW%5BAuthor%5D&cauthor=true&cauthor_uid=15943733), [Ibrahim S](https://www.ncbi.nlm.nih.gov/pubmed/?term=Ibrahim%20S%5BAuthor%5D&cauthor=true&cauthor_uid=15943733), [Tay KH](https://www.ncbi.nlm.nih.gov/pubmed/?term=Tay%20KH%5BAuthor%5D&cauthor=true&cauthor_uid=15943733). Hartmann procedure: is it still relevant today? [ANZ J Surg.](https://www.ncbi.nlm.nih.gov/pubmed/?term=ANZ+J+Surg.+2005+Jun%3B75(6)%3A436-40) 2005;75(6):436-40.
34. [Constantinides VA](https://www.ncbi.nlm.nih.gov/pubmed/?term=Constantinides%20VA%5BAuthor%5D&cauthor=true&cauthor_uid=17048279), [Tekkis PP](https://www.ncbi.nlm.nih.gov/pubmed/?term=Tekkis%20PP%5BAuthor%5D&cauthor=true&cauthor_uid=17048279), [Senapati A](https://www.ncbi.nlm.nih.gov/pubmed/?term=Senapati%20A%5BAuthor%5D&cauthor=true&cauthor_uid=17048279); [Association of Coloproctology of Great Britain Ireland](https://www.ncbi.nlm.nih.gov/pubmed/?term=Association%20of%20Coloproctology%20of%20Great%20Britain%20Ireland%5BCorporate%20Author%5D). Prospective multicentre evaluation of adverse outcomes following treatment for complicated diverticular disease. [Br J Surg.](https://www.ncbi.nlm.nih.gov/pubmed/?term=Br+J+Surg.+2006+Dec%3B93(12)%3A1503-13.) 2006;93(12):1503-13.
35. [Aydin HN](https://www.ncbi.nlm.nih.gov/pubmed/?term=Aydin%20HN%5BAuthor%5D&cauthor=true&cauthor_uid=16598405), [Tekkis PP](https://www.ncbi.nlm.nih.gov/pubmed/?term=Tekkis%20PP%5BAuthor%5D&cauthor=true&cauthor_uid=16598405), [Remzi FH](https://www.ncbi.nlm.nih.gov/pubmed/?term=Remzi%20FH%5BAuthor%5D&cauthor=true&cauthor_uid=16598405), [Constantinides V](https://www.ncbi.nlm.nih.gov/pubmed/?term=Constantinides%20V%5BAuthor%5D&cauthor=true&cauthor_uid=16598405), [Fazio VW](https://www.ncbi.nlm.nih.gov/pubmed/?term=Fazio%20VW%5BAuthor%5D&cauthor=true&cauthor_uid=16598405). Evaluation of the risk of a nonrestorative resection for the treatment of diverticular disease: the Cleveland Clinic diverticular disease propensity score. [Dis Colon Rectum.](https://www.ncbi.nlm.nih.gov/pubmed/?term=Dis+Colon+Rectum.+2006+May%3B49(5)%3A629-39.) 2006;49(5):629-39.
36. [Constantinides VA](https://www.ncbi.nlm.nih.gov/pubmed/?term=Constantinides%20VA%5BAuthor%5D&cauthor=true&cauthor_uid=16752192), [Tekkis PP](https://www.ncbi.nlm.nih.gov/pubmed/?term=Tekkis%20PP%5BAuthor%5D&cauthor=true&cauthor_uid=16752192), [Athanasiou T](https://www.ncbi.nlm.nih.gov/pubmed/?term=Athanasiou%20T%5BAuthor%5D&cauthor=true&cauthor_uid=16752192), [Aziz O](https://www.ncbi.nlm.nih.gov/pubmed/?term=Aziz%20O%5BAuthor%5D&cauthor=true&cauthor_uid=16752192), [Purkayastha S](https://www.ncbi.nlm.nih.gov/pubmed/?term=Purkayastha%20S%5BAuthor%5D&cauthor=true&cauthor_uid=16752192), [Remzi FH](https://www.ncbi.nlm.nih.gov/pubmed/?term=Remzi%20FH%5BAuthor%5D&cauthor=true&cauthor_uid=16752192), [Fazio VW](https://www.ncbi.nlm.nih.gov/pubmed/?term=Fazio%20VW%5BAuthor%5D&cauthor=true&cauthor_uid=16752192), [Aydin N](https://www.ncbi.nlm.nih.gov/pubmed/?term=Aydin%20N%5BAuthor%5D&cauthor=true&cauthor_uid=16752192), [Darzi A](https://www.ncbi.nlm.nih.gov/pubmed/?term=Darzi%20A%5BAuthor%5D&cauthor=true&cauthor_uid=16752192), [Senapati A](https://www.ncbi.nlm.nih.gov/pubmed/?term=Senapati%20A%5BAuthor%5D&cauthor=true&cauthor_uid=16752192). Primary resection with anastomosis vs. Hartmann's procedure in nonelective surgery for acute colonic diverticulitis: a systematic review. [Dis Colon Rectum.](https://www.ncbi.nlm.nih.gov/pubmed/?term=Dis+Colon+Rectum.+2006+Jul%3B49(7)%3A966-81.) 2006;49(7):966-81.
37. [Stumpf MJ](https://www.ncbi.nlm.nih.gov/pubmed/?term=Stumpf%20MJ%5BAuthor%5D&cauthor=true&cauthor_uid=17879685), [Vinces FY](https://www.ncbi.nlm.nih.gov/pubmed/?term=Vinces%20FY%5BAuthor%5D&cauthor=true&cauthor_uid=17879685), [Edwards J](https://www.ncbi.nlm.nih.gov/pubmed/?term=Edwards%20J%5BAuthor%5D&cauthor=true&cauthor_uid=17879685). Is primary anastomosis safe in the surgical management of complications of acute diverticulitis? [Am Surg.](https://www.ncbi.nlm.nih.gov/pubmed/?term=Am+Surg.+2007+Aug%3B73(8)%3A787-90) 2007;73(8):787-90
38. [Constantinides VA](https://www.ncbi.nlm.nih.gov/pubmed/?term=Constantinides%20VA%5BAuthor%5D&cauthor=true&cauthor_uid=17197971), [Heriot A](https://www.ncbi.nlm.nih.gov/pubmed/?term=Heriot%20A%5BAuthor%5D&cauthor=true&cauthor_uid=17197971), [Remzi F](https://www.ncbi.nlm.nih.gov/pubmed/?term=Remzi%20F%5BAuthor%5D&cauthor=true&cauthor_uid=17197971), [Darzi A](https://www.ncbi.nlm.nih.gov/pubmed/?term=Darzi%20A%5BAuthor%5D&cauthor=true&cauthor_uid=17197971), [Senapati A](https://www.ncbi.nlm.nih.gov/pubmed/?term=Senapati%20A%5BAuthor%5D&cauthor=true&cauthor_uid=17197971), [Fazio VW](https://www.ncbi.nlm.nih.gov/pubmed/?term=Fazio%20VW%5BAuthor%5D&cauthor=true&cauthor_uid=17197971), [Tekkis PP](https://www.ncbi.nlm.nih.gov/pubmed/?term=Tekkis%20PP%5BAuthor%5D&cauthor=true&cauthor_uid=17197971). Operative strategies for diverticular peritonitis: a decision analysis between primary resection and anastomosis versus Hartmann's procedures. [Ann Surg.](https://www.ncbi.nlm.nih.gov/pubmed/?term=Ann+Surg.+2007+Jan%3B245(1)%3A94-103.) 2007;245(1):94-103.
39. [Cirocchi R](https://www.ncbi.nlm.nih.gov/pubmed/?term=Cirocchi%20R%5BAuthor%5D&cauthor=true&cauthor_uid=25437034), [Arezzo A](https://www.ncbi.nlm.nih.gov/pubmed/?term=Arezzo%20A%5BAuthor%5D&cauthor=true&cauthor_uid=25437034), [Vettoretto N](https://www.ncbi.nlm.nih.gov/pubmed/?term=Vettoretto%20N%5BAuthor%5D&cauthor=true&cauthor_uid=25437034), [Cavaliere D](https://www.ncbi.nlm.nih.gov/pubmed/?term=Cavaliere%20D%5BAuthor%5D&cauthor=true&cauthor_uid=25437034), [Farinella E](https://www.ncbi.nlm.nih.gov/pubmed/?term=Farinella%20E%5BAuthor%5D&cauthor=true&cauthor_uid=25437034), [Renzi C](https://www.ncbi.nlm.nih.gov/pubmed/?term=Renzi%20C%5BAuthor%5D&cauthor=true&cauthor_uid=25437034), [Cannata G](https://www.ncbi.nlm.nih.gov/pubmed/?term=Cannata%20G%5BAuthor%5D&cauthor=true&cauthor_uid=25437034), [Desiderio J](https://www.ncbi.nlm.nih.gov/pubmed/?term=Desiderio%20J%5BAuthor%5D&cauthor=true&cauthor_uid=25437034), [Farinacci F](https://www.ncbi.nlm.nih.gov/pubmed/?term=Farinacci%20F%5BAuthor%5D&cauthor=true&cauthor_uid=25437034), [Barberini F](https://www.ncbi.nlm.nih.gov/pubmed/?term=Barberini%20F%5BAuthor%5D&cauthor=true&cauthor_uid=25437034), [Trastulli S](https://www.ncbi.nlm.nih.gov/pubmed/?term=Trastulli%20S%5BAuthor%5D&cauthor=true&cauthor_uid=25437034), [Parisi A](https://www.ncbi.nlm.nih.gov/pubmed/?term=Parisi%20A%5BAuthor%5D&cauthor=true&cauthor_uid=25437034), [Fingerhut A](https://www.ncbi.nlm.nih.gov/pubmed/?term=Fingerhut%20A%5BAuthor%5D&cauthor=true&cauthor_uid=25437034). Role of damage control surgery in the treatment of Hinchey III and IV sigmoid diverticulitis: a tailored strategy. [Medicine (Baltimore).](https://www.ncbi.nlm.nih.gov/pubmed/?term=Medicine+93(25)%3Ae184) 2014;93(25):e184
40. [Sohn M](https://www.ncbi.nlm.nih.gov/pubmed/?term=Sohn%20M%5BAuthor%5D&cauthor=true&cauthor_uid=27448296), [Agha A](https://www.ncbi.nlm.nih.gov/pubmed/?term=Agha%20A%5BAuthor%5D&cauthor=true&cauthor_uid=27448296), [Heitland W](https://www.ncbi.nlm.nih.gov/pubmed/?term=Heitland%20W%5BAuthor%5D&cauthor=true&cauthor_uid=27448296), [Gundling F](https://www.ncbi.nlm.nih.gov/pubmed/?term=Gundling%20F%5BAuthor%5D&cauthor=true&cauthor_uid=27448296), [Steiner P](https://www.ncbi.nlm.nih.gov/pubmed/?term=Steiner%20P%5BAuthor%5D&cauthor=true&cauthor_uid=27448296), [Iesalnieks I](https://www.ncbi.nlm.nih.gov/pubmed/?term=Iesalnieks%20I%5BAuthor%5D&cauthor=true&cauthor_uid=27448296). Damage control strategy for the treatment of perforated diverticulitis with generalized peritonitis. [Tech Coloproctol.](https://www.ncbi.nlm.nih.gov/pubmed/?term=Tech+Coloproctol.+2016+Aug%3B20(8)%3A577-83) 2016;20(8):577-83

**Q5.4:** What is the recommended extent of sigmoid resection and what is the best practice for splenic flexure mobilization?

Expert recommendation: In the setting of an emergency HP, we recommend limiting the resection to the acutely affected segment and not mobilizing the splenic flexure unless necessary.

SOR: Strong for using
LOE: NA

Although there are no studies evaluating the extent of sigmoid resection in the emergency setting, limiting the resection to the segment that is acutely affected without compromising blood supply of the remnant bowel is recommended. Furthermore, a dissection performed close to the bowel wall will decrease the risk of injury to surrounding structures and organs especially in the context of peritonitis and a phlegmon. No studies were identified to guide the management of the rectal stump in the emergency setting to decrease the risk of rectal stump blowouts. Furthermore, despite widespread use, our literature search identified no evidence to support routine use of abdominal drains in the setting of perforated diverticulitis.

**Q5.5:** What is the incidence of postop complications?

Statement: Emergency surgery for perforated diverticulitis is associated with increased morbidity and mortality compared to elective surgery.

Compared to elective surgery, emergency surgery for perforated diverticulitis is associated with increased morbidity and mortality.

LOE: low

No recommendation

Emergency colorectal surgery places patients at increased risk of postoperative complications, and this is still true for patients undergoing surgery for acute perforated diverticulitis. Although the differences in patient characteristics undergoing elective and emergency surgery for diverticulitis would bias a direct comparison between the two settings, it remains important to be aware of this heightened risk of complications in the emergency setting (Alvez 2007, Kim 2006).

1. Alves, Arnaud Panis, Yves Mantion, Georges Slim, Karem Kwiatkowski, Fabrice Vicaut, Eric Journal Article Multicenter Study United States Ann Surg. 2007 Jul;246(1):91-6.
2. Kim, Justin Mittal, Raj Konyalian, Viken King, Justin Stamos, Michael J Kumar, Ravin R Journal Article United States Am Surg. 2007 Oct;73(10):991-3.

Note: this may be better integrated into the part discussing outcomes for elective diverticulitis.

**Q5.6:** How should complicated emergency diverticulitis be managed among specific patients gp?

Statement:

Immunosuppressed patients have increased mortality and morbidity following emergency surgery compared to immunocompetent individuals.

Statement:

Elderly patients with perforated diverticulitis have a higher mortality rate following emergency surgery.

LOE: Low

Amongst the most important recurrent risk factors identified in the literature for increasing morbidity and mortality for patients undergoing surgery for acute perforated diverticulitis are age and immunosuppression status. Immunosuppression was identified as a risk factor for mortality and morbidity in several retrospective series^1-5^. Similar to other colorectal pathologies, age has been found to be a risk factor for increasing mortality for patients undergoing surgery for acute diverticulitis. Although the age cut-off differed between studies, the importance of age as a risk factor for complications after emergency surgery for diverticulitis is to be noted given the increasingly aging population being treated and presenting with acute complicated diverticulitis requiring surgical intervention^6-9^.

1. Al-Khamis, Ahmed Abou Khalil, Jad Demian, Marie Morin, Nancy Vasilevsky, Carol-Ann Gordon, Philip H Boutros, Marylise Journal Article United States Dis Colon Rectum. 2016 Feb;59(2):101-9. doi: 10.1097/DCR.0000000000000513.
2. Hansen, O Graupe, F Stock, W English Abstract Journal Article Germany Chirurg. 1998 Apr;69(4):443-9.
3. Brandl, Andreas Kratzer, Theresa Kafka-Ritsch, Reinhold Braunwarth, Eva Denecke, Christian Weiss, Sascha Atanasov, Georgi Sucher, Robert Biebl, Matthias Aigner, Felix Pratschke, Johann Ollinger, Robert Journal Article Canada Can J Surg. 2016 Aug;59(4):254-61. doi: 10.1503/cjs.012915.
4. Golda, T Kreisler, E Mercader, C Frago, R Trenti, L Biondo, S Comparative Study Evaluation Studies Journal Article England Colorectal Dis. 2014 Sep;16(9):723-31. doi: 10.1111/codi.12685.
5. Biondo, Sebastiano Trenti, Loris Elvira, Jordi Golda, Thomas Kreisler, Esther Journal Article  Observational Study United States Am J Surg. 2016 Sep;212(3):384-90. doi: 10.1016/j.amjsurg.2016.01.038. Epub 2016 May 7.
6. Anegawa, Go Nakashima, Yuichiro Masuda, Takanobu Shimabukuro, Rinshun Takahashi, Ikuo Nishizaki, Takashi Journal Article Japan Surg Today. 2013 Oct;43(10):1150-3. doi: 10.1007/s00595-013-0514-8. Epub 2013 Feb 19.
7. Harries, Rhiannon L Twine, Chris P Kugathasan, Gana Young, Holly Jones, Evelyn Gomez, Kelvin F Journal Article England Postgrad Med J. 2012 Apr;88(1038):205-9. doi: 10.1136/postgradmedj-2011-130412. Epub 2012 Feb 25.
8. Dumont, F Vibert, E Duval, H Manaouil, D Sredic, A Alfahel, N Mauvais, F De Fresnoy, H Rudant, J Katsahian, S Riboulot, M Galy, C Verhaeghe, P Dupont, H Regimbeau, J-M English Abstract Journal Article France Ann Chir. 2005 Jul-Aug;130(6-7):391-9.
9. Makela, Jyrki Tapani Kiviniemi, Heikki Laitinen, Seppo Journal Article Switzerland Dig Surg. 2005;22(1-2):100-6. Epub 2005 Apr 20.
