# Supplementary material for: EAES and SAGES 2018 consensus conference on acute diverticulitis management: evidence-based recommendations for clinical practice
Source: Surg Endosc. 2019 Jun 27;33(9):2726–41. doi: 10.1007/s00464-019-06882-z (PMC6684540; doi:10.1007/s00464-019-06882-z)
Supplement: Supplementary file 8 — Supplementary material 8 (DOCX 86 kb) [file 464_2019_6882_MOESM8_ESM.docx]

**Topic VI: Elective operative management of acute diverticulitis**

**Q6.1: What is the role of laparoscopy in elective diverticulitis?**

Statement: Laparoscopy is safe in the setting of elective surgery for diverticulitis and is associated with reduced rates of morbidity and length of stay compared to open surgery.

LoE: **** (High quality)

Recommendation: We recommend a laparoscopic approach in elective surgery for diverticular disease, when feasible.

LoE: **** (High quality)

SoR: Strong

The role of laparoscopy in elective surgery for treatment of diverticulitis has been well studied, with a high level of available evidence, which includes four randomized trials[1-4] and three meta-analyses of randomized trials.[5-7] Meta-analyses by Siddiqui et al.[5] and Cirocchi et al.[7] reported significantly reduced rates of postoperative morbidity for patients who underwent laparoscopic surgery; Siddiqui et al. also reported a reduced length of stay (this endpoint was not assessed in the analysis by Cirocchi et al.). An earlier, more limited meta-analysis did not find any difference in morbidity rates, however this was more limited to fewer studies, patients, and of poorer quality.[6] Of the randomized trials, all reported at least equivalent, if not superior, outcomes for laparoscopy. Two of the four studies reported reduced morbidity for laparoscopy,[4,1] while the remaining two studies reported no difference. Two[1,2] of three studies, which assessed length of stay, reported reduced hospital stays and return to function for laparoscopy. Three of four studies reported reduced pain scores or analgesic requirements for laparoscopy,[4,2,1] while one reported no difference.[3]

**Q6.2: When is elective interval sigmoid resection indicated following an episode(s) of complicated acute or chronic diverticulitis?**

Statement: Limited evidence suggests no difference in morbidity or mortality when comparing early (<6 weeks) versus late (>6 weeks) elective resection for diverticular disease; conversion to open rates may be higher in early surgery.

LoE: ** (Low quality)

Recommendation: Consideration should be given to delaying elective interval sigmoid resection for minimum 6 weeks from the most recent episode of acute diverticulitis.

LoE: ** (Low quality)

SoR: Weak

Limited retrospective data is available to make specific recommendations with reference to the optimal interval between episodes of acute diverticulitis and elective resection. A meta-analysis[8] of four low-quality cohort studies compared outcomes for early (< 6 weeks) versus late (≥ 6 weeks) surgery depending on the time interval since the last episode of diverticulitis.. Authors reported no significant difference in morbidity or mortality. However, risk of conversion to open surgery was more than doubled in the early surgery group, while duration of surgery and length of stay were significantly longer in the early surgery group compared to the delayed surgery group.

**Q6.3: When should prophylactic ureteral stents be used prior to elective surgery for diverticulitis?**

Statement: No evidence is available regarding the efficacy of prophylactic ureteric stenting in elective surgery for diverticulitis.

LoE: No evidence

Recommendation: We recommend the utilization of a selective strategy based on imaging and patient characteristics for placement of prophylactic ureteral stents prior to elective surgery for diverticulitis.

LoE: * (Very low quality)

SoR: Weak

No evidence is available which specifically compares outcomes for surgery with or without the use of ureteric stenting in surgery for diverticulitis. A study analysing 51,125 patients from the NSQIP database reported that for all colectomy procedures (regardless of indication), ureteric stent placement was associated with a significantly reduced risk of ureteric injury.[9] Diverticulitis as the indication for surgery was the most common setting in which ureteric injury was seen to occur. Several case series[10-12] describe the safe and effective use of ureteric stents, particularly in the context of complex, chronic, or fistulous diverticular disease where anatomic distortion by the diverticular phlegmon is expected. More specific criteria for the selection of patients who should be considered for prophylactic stenting are not described. Where reported, the use of stents to identify and preserve the ureters is described as safe and successful with no reported intraoperative ureteric injuries. One analysis of the National Inpatient Sample database found the use of ureteric stents to be increasing, with 16.3% of operations for diverticulitis utilizing stents in 2013.[13] Complications resulting from ureteric stenting are reported in by one study to include macroscopic hematuria in up to 95% of cases, and urinary tract infection in 6% of cases.[10] While not specific to diverticular disease, Hassinger et al. found the use of ureteral stents, particularly when bilateral, to be independently associated with the development of postoperative acute kidney injury following colorectal surgery (OR 3.82 95% CI 2.17-6.75; p<0.001).[14]

**Q6.4: What is the role of bowel preparation prior to surgery in the management of diverticulitis?**

Recommendation: While the evidence specific to diverticular disease is limited, evidence exists in the setting of elective colorectal surgery to recommend the use of an isosmotic mechanical bowel preparation with oral antibiotics prior to surgery.

LoE: *** (Moderate quality)

SoR: Strong

Very limited data is available which examines the effect of bowel preparation in the specific setting of surgery for diverticular disease. Van’t Sant et al. report a secondary analysis of a randomized trial of bowel preparation versus no bowel preparation in colonic surgery (not specific to diverticulitis), in which they selected out 190 patients for whom diverticulitis was the indication for surgery. As a secondary analysis, this study cannot be said to have been adequately powered, however authors reported no significant differences in morbidity, leak rates, or intraoperative contamination.[15]

Higher level evidence is available regarding mechanical bowel preparation in colonic surgery in general. Numerous large database analyses report the reduced incidence of surgical site infection, anastomotic leak, and postoperative ileus with the combined use of preoperative mechanical bowel preparation and oral antibiotics.[16-18] This effect is not seen with the use of either mechanical bowel preparation or oral antibiotics alone. These studies included all colonic surgery regardless of indication; we therefore recommend the use of preoperative isosmotic mechanical bowel preparation and oral antibiotics in the context of surgery for diverticulitis as well.

**Q6.5: What is the optimal surgical strategy in the elective setting for complicated and uncomplicated diverticulitis?**

Recommendation: Preservation of the inferior mesenteric artery should be considered to preserve vascular supply of the anastomosis (so long as this does not compromise formation of a tension-free anastomosis).

LoE: ** (Low quality)

SoR: Weak

Literature regarding specifics of the surgical approach to sigmoid resection for diverticulitis is limited. Surgical strategy should thus be guided by surgical principles which allow resection of the diseased segment of colon, preservation of surrounding structures, and a safe colo-rectal anastomosis. One trial of 163 patients randomized to either inferior mesenteric artery (IMA) ligation versus IMA preservation in elective open sigmoidectomy for complicated diverticular disease[19] reported significantly lower rates of anastomotic leak for IMA preservation (7% vs. 18.1% for IMA ligation). A later meta-analysis[20] did not find any statistically significant difference in anastomotic leak rate; however, it is worth noting that this study’s dataset was composed of outcomes from the aforementioned trial grouped together with three small retrospective cohort studies, and thus of poor quality.

**Q6.6: When is a Hartmann’s procedure indicated in the elective setting?**

Recommendation: Every effort should be made to construct a primary anastomosis in the elective setting for complicated and uncomplicated diverticulitis.

LoE: No evidence

SoR: Weak

No evidence is available to aid the selection of patients who should be considered for a Hartmann’s procedure in the elective setting. The decision should be taken on an individual basis considering all appropriate patient and surgical factors, and be guided by standard principles of safe surgery and the formation of a safe anastomosis where possible.

**Q6.7: What is the recommended extent of sigmoid resection, including mobilization of proximal bowel and mesentery/phlegmon dissection?**

Statement: No differences in leak, morbidity or mortality rates are reported when comparing elective diverticular resections with versus without splenic flexure mobilization.

LoE: ** (Low quality)

Recommendation: Although routine mobilization of the splenic flexure is not supported by evidence, we recommend that the descending colon should be fully mobilized to provide sufficient colonic length to form a tension-free anastomosis.

LoE: ** (Low quality)

SoR: Weak

Limited evidence regarding the necessity of splenic flexure mobilization, or necessary extent of dissection, is available. In one retrospective study, comparing splenic flexure mobilization with no mobilization, authors reported no differences in morbidity rate.[21] A second study considered factors associated with recurrence of diverticulitis following colectomy for diverticular disease, and did not find any significant association with splenic flexure mobilization.[22] Finally, another study considered differences in surgical factors between laparoscopic and open sigmoidectomy.[23] Authors reported a significantly shorter specimen length when considering the resected colon in laparoscopic cases, and concluded that full splenic flexure mobilization should be attempted to ensure adequate resection.

**Q6.8: What is the optimal level of resection proximally and distally, and how should the rectum be transected (as it relates to rectal preservation and defecatory function)?**

Recommendation: We recommend transecting the colon proximal to the phlegmon in an area without gross evidence of inflammation. No attempt should be made to resect every diverticulum proximal to the phlegmon.

LoE: No evidence

SoR: Weak

Recommendation: We recommend distal transection at or below the rectosigmoid junction (at the level of the sacral promontory where the tenia coli coalesce) to decrease the risk of recurrent diverticulitis.

LoE: ** (Low quality)

SoR: Strong

The optimal level of both proximal and distal colon resection—and associated length of specimen—for diverticular disease remains an unsettled issue. Published rates of recurrent diverticulitis following resection range from 3 to 13%, with higher rates seen with colosigmoid as compared to colorectal anastomoses [22]. The ability to obtain an adequate specimen length with laparoscopic resection has likewise been debated in the literature; however, there is currently no evidence to suggest that patients undergoing laparoscopic resections experience a higher rate of diverticular disease recurrence [22,23]. Given the lack of evidence, many experts recommend transecting the colon proximal to the phlegmon or area of active inflammation in a location without gross evidence of inflammation. No attempt should be made to resect every diverticulum proximal to the affected segment. Given the increased risk of recurrent disease with a colosigmoid anastomosis, distal transection should occur at or below the rectosigmoid junction, at the level of the sacral promontory where the tenia coli coalesce. If gross inflammation extends past the rectosigmoid junction, the distal transection may need to occur at a lower level.

**Q6.9: What is the optimal strategy of colorectal anastomosis, and how could this be assessed?**

Statement: Postoperative morbidity is not impacted by choice of handsewn or stapled anastomosis for elective resection of diverticular disease.

LoE: ** (Low quality)

Recommendation: We recommend the use of either a handsewn or stapled anastomosis based on individual surgeon preference.

LoE: ** (Low quality)

SoR: Weak

Recommendation: While there is no evidence specific to diverticular disease, based on the evidence from the general colorectal population we recommend use of an air leak test to evaluate the integrity of the colorectal anastomosis and prevent anastomotic leak.

LoE: ** (Low quality)

SoR: Strong

The use of stapling devices to create colorectal anastomoses continues to expand, as many surgeons find the technology efficient and time saving. Low colorectal anastomoses are certainly facilitated with stapling devices; however, the benefits for an anastomosis to the proximal rectum remain less clear. In a non-randomized cohort study of 116 patients undergoing an elective left hemicolectomy with colonic anastomosis to the proximal rectum, Sielezneff et al. identified no difference in short-term morbidity—including anastomotic leak, hemorrhage, and fecal incontinence—or mortality between patients undergoing handsewn or stapled techniques. Rates of constipation and dyschezia were noted to be higher following stapled anastomosis at a 6-month patient-reported follow up, though many of these patients experienced symptoms preoperatively as well [24]. Additionally, Thaler et al. found no difference in recurrence of diverticular disease based on handsewn or stapled colorectal anastomosis [22]. Given evidence of equivalent outcomes, experts recommend the utilization of either handsewn or stapled techniques based on individual surgeon preference. For stapled anastomoses, if the stapler does not reach and efface with the staple line of the rectal stump during construction of the colorectal anastomosis, this is indicative of residual sigmoid colon. In this situation, experts recommend resection of the residual sigmoid colon as opposed to advancement of the stapler out the anterior wall of the rectum.

There is no evidence in the diverticular-specific literature to evaluate the need for, or best modality of, assessment of the anastomosis. Based on the evidence from the general colorectal population, experts recommend the use of an air leak test to evaluate the integrity of the colorectal anastomosis and prevent anastomotic leak [25]. However, direct evaluation of the anastomosis with proctoscope or colonoscope could also be considered based on surgeon experience and preference [26].

**Q6.10: What is the role and management of abdominal/pelvic drains following elective resection for complicated diverticulitis?**

Recommendation: There is no evidence to support routine use of abdominal/pelvic drain in elective surgery for diverticulitis. We recommend the decision to place an abdominal or pelvic drain following elective resection for complicated diverticular disease be left to the surgeon’s discretion.

LoE: No evidence

SoR: Weak

There is no specific evidence available to suggest abdominal/pelvic drains are effective at preventing complications following elective resection for complicated diverticular disease. However, there are multiple studies suggesting no benefit to the use of routine abdominal/pelvic drains following all elective colon or rectal resections, and two recent meta-analyses found no difference in postoperative morbidity (including anastomotic leak) or mortality [27,28]. As such, experts recommend the decision to place an abdominal or pelvic drain following elective resection for complicated diverticular disease be left to the surgeon’s discretion.

**Q6.11: What is the incidence of postoperative complications following elective surgery for diverticular disease?**

Statement: The incidence of postoperative complications following elective surgery for diverticular disease varies widely, ranging from 5%-38%. Laparoscopic surgery conveys a lower risk of postoperative complications as compared to open resection.

LoE: **** (High quality)

The incidence of postoperative complications following elective surgery for diverticular disease varies widely in the publishing literature, ranging from 5 to 38% [1,29]. The most commonly cited complications include surgical site infections, ileus, and anastomotic leak. Laparoscopic surgery has been consistently shown to result in lower rates of postoperative morbidity—particularly major morbidity—following elective surgery for diverticular disease [6,5].

**Q6.12: What are the functional outcomes postoperatively (short-term), including defecatory and sexual function and quality of life following elective surgery for diverticular disease?**

Statement: Short-term functional outcomes and quality of life are improved in patients following elective resection for diverticular disease as compared to patients with conservatively managed disease.

LoE: *** (Moderate quality)

Recommendation: We recommend elective resection in patients with symptomatic diverticular disease that is negatively impacting quality of life.

LoE: *** (Moderate quality)

SoR: Strong

Statement: Short-term functional outcomes and quality of life are improved following laparoscopic elective resection of diverticular disease as compared to open resection.

LoE: *** (Moderate quality)

Recommendation: We recommend a laparoscopic approach for elective resection of diverticular disease when feasible to improve short-term functional outcomes and quality of life.

LoE: *** (Moderate quality)

SoR: Strong

The timing and need for elective surgery in patients following recurrent episodes of diverticular disease or continued abdominal complaints has changed over the past decade. While there is no longer a definite need for elective resection after two episodes of diverticulitis, patients who remain symptomatic benefit from elective resection to improve short-term functional outcomes and quality of life, as suggested by a multicenter randomized trial [30].

For patients who undergo elective resection for diverticular disease, laparoscopic surgery has been shown to result in improved short-term functional outcomes and quality of life as compared to open surgery. Several randomized controlled trials have reported superior scores on patient-reported quality of life surveys and for pain metrics in patients who underwent laparoscopic elective diverticular resection up to 6 months following surgery when compared to patients after open resection [1,31,32]. These differences begin to disappear by 6 to 12 months following resection [1,32]; however, given the improvements in short-term quality of life and functional outcomes, experts recommend a laparoscopic approach for elective resection of diverticular disease, when feasible.

**Q6.14: How should complicated elective diverticulitis be managed among specific patient groups?**

Statement: Complicated elective diverticulitis in overweight/obese patients can be safely managed with laparoscopic surgery with similar morbidity and rate of conversion to normal weight patients.

LoE: ** (Low quality)

Recommendation: We recommend a laparoscopic approach for the elective resection of diverticular disease in obese patients, when feasible.

LoE: ** (Low quality)

SoR: Weak

Recommendation: When considering elective resection of complicated diverticular disease in the immunocompromised patient, we recommend a lower threshold for stoma formation.

LoE: ** (Low quality)

SoR: Weak

Abdominal surgery—particularly laparoscopic surgery—in overweight and obese patients is often more challenging than similar procedures in normal weight individuals due to technical intraoperative issues as well as concern for increased morbidity with increased body mass index (BMI). Tuech et al. performed a prospective study comparing laparoscopic elective colectomy for diverticular disease among normal weight (BMI 18.0-24.9 kg/m^2^), overweight (BMI 25.0-29.9), and obese (BMI 30.0-39.9) patients finding no difference in rates of conversion to open surgery, length of resected colonic specimen, or hospital length of stay between normal weight and overweight patients or between normal weight and obese patients. There was also no difference in postoperative morbidity between groups. While operative time did not differ for the normal weight and overweight patients, it was longer for the obese group as compared to normal weight [33].

Diverticular disease is common among elderly patients, and surgical management is often complicated by the higher rate of comorbid medical conditions and decreased functional reserve among these patients. A single center non-randomized cohort study compared morbidity and mortality among elderly patients (age >75 years) undergoing laparoscopic and open elective colectomy for diverticular disease. Elderly patients undergoing laparoscopic surgery had a lower rate of postoperative morbidity and required intravenous pain medications for a shorter duration, all with a conversion rate comparable to published rates [34].

Immunosuppressed patients represent a unique and increasing subset of patients with diverticulitis. Management of diverticular disease in these patients—including timing and type of operative intervention—remains controversial, as immunosuppressed patients are believed to have both a higher incidence of index and recurrent disease. The controversy is further complicated by the heterogeneity among this group of patients, with differing definitions and subsets of patients included in each study. A small, single institution study by Reshef et al. found no difference in morbidity and mortality following elective resection among solid organ transplant recipients and immunocompetent patients [35]. However, other data—including a large study utilizing the American College of Surgeons National Quality Improvement Project database—indicate that while immunocompromised patients are not at increased risk for mortality following elective resection compared to immunocompetent patients, they are more likely to experience postoperative complications, including wound dehiscence, deep and organ space surgical site infections, and overall major morbidity [36,37]. Given the likelihood for increased complications—including those related to deep and organ space surgical site infections, potentially related to anastomotic leak—experts recommend a lower threshold for stoma formation when performing an elective resection for diverticular disease in an immunosuppressed patient.

**References**

1. Klarenbeek BR, Veenhof AA, Bergamaschi R, van der Peet DL, van den Broek WT, de Lange ES, Bemelman WA, Heres P, Lacy AM, Engel AF, Cuesta MA (2009) Laparoscopic sigmoid resection for diverticulitis decreases major morbidity rates: a randomized control trial: short-term results of the Sigma Trial. Ann Surg 249 (1):39-44. doi:10.1097/SLA.0b013e31818e416a

2. Gervaz P, Inan I, Perneger T, Schiffer E, Morel P (2010) A prospective, randomized, single-blind comparison of laparoscopic versus open sigmoid colectomy for diverticulitis. Ann Surg 252 (1):3-8

3. Raue W, Paolucci V, Asperger W, Albrecht R, Buchler MW, Schwenk W, Group L-CT (2011) Laparoscopic sigmoid resection for diverticular disease has no advantages over open approach: midterm results of a randomized controlled trial. Langenbecks Arch Surg 396 (7):973-980. doi:10.1007/s00423-011-0825-4

4. Rink AD, John-Enzenauer K, Haaf F, Straub E, Nagelschmidt M, Vestweber KH (2009) Laparoscopic-assisted or laparoscopic-facilitated sigmoidectomy for diverticular disease? A prospective randomized trial on postoperative pain and analgesic consumption. Dis Colon Rectum 52 (10):1738-1745

5. Siddiqui MR, Sajid MS, Khatri K, Cheek E, Baig MK (2010) Elective open versus laparoscopic sigmoid colectomy for diverticular disease: a meta-analysis with the Sigma trial. World J Surg 34 (12):2883-2901. doi:10.1007/s00268-010-0762-3

6. Siddiqui MR, Sajid MS, Qureshi S, Cheek E, Baig MK (2010) Elective laparoscopic sigmoid resection for diverticular disease has fewer complications than conventional surgery: a meta-analysis. Am J Surg 200 (1):144-161. doi:10.1016/j.amjsurg.2009.08.021

7. Cirocchi R, Farinella E, Trastulli S, Sciannameo F, Audisio RA (2012) Elective sigmoid colectomy for diverticular disease. Laparoscopic vs open surgery: a systematic review. Colorectal Dis 14 (6):671-683

8. Khan RMA, Hajibandeh S, Hajibandeh S (2017) Early elective versus delayed elective surgery in acute recurrent diverticulitis: A systematic review and meta-analysis. Int J Surg 46:92-101

9. Coakley KM, Kasten KR, Sims SM, Prasad T, Heniford BT, Davis BR (2018) Prophylactic Ureteral Catheters for Colectomy: A National Surgical Quality Improvement Program-Based Analysis. Dis Colon Rectum 61 (1):84-88. doi:10.1097/DCR.0000000000000976

10. Blake MF, Dwivedi A, Tootla A, Tootla F, Silva YJ (2005) Laparoscopic sigmoid colectomy for chronic diverticular disease. Jsls 9 (4):382-385

11. Abbass MA, Tsay AT, Abbas MA (2013) Laparoscopic resection of chronic sigmoid diverticulitis with fistula. Jsls 17 (4):636-640

12. Schwandner O, Farke S, Fischer F, Eckmann C, Schiedeck TH, Bruch HP (2004) Laparoscopic colectomy for recurrent and complicated diverticulitis: a prospective study of 396 patients. Langenbecks Arch Surg 389 (2):97-103

13. Chiu AS, Jean RA, Gorecka J, Davis KA, Pei KY. Trends of ureteral stent usage in surgery for diverticulitis. J Surg Res. 2018 Feb;222:203-211.

14. Hassinger TE, Mehaffey JH, Mullen MG, Michaels AD, Elwood NR, Levi ST, Hedrick TL, Friel CM (2018) Ureteral stents increase risk of postoperative acute kidney injury following colorectal surgery. Surg Endosc. doi:10.1007/s00464-018-6054-y

15. Van't Sant HP, Slieker JC, Hop WC, Weidema WF, Lange JF, Vermeulen J, Contant CM (2012) The influence of mechanical bowel preparation in elective colorectal surgery for diverticulitis. Tech Coloproctol 16 (4):309-314

16. Koller SE, Bauer KW, Egleston BL, Smith R, Philp MM, Ross HM, Esnaola NF (2017) Comparative Effectiveness and Risks of Bowel Preparation Before Elective Colorectal Surgery. Ann Surg. doi:10.1097/SLA.0000000000002159

17. Kiran RP, Murray AC, Chiuzan C, Estrada D, Forde K (2015) Combined preoperative mechanical bowel preparation with oral antibiotics significantly reduces surgical site infection, anastomotic leak, and ileus after colorectal surgery. Ann Surg 262 (3):416-425; discussion 423-415. doi:10.1097/SLA.0000000000001416

18. Klinger AL, Green H, Monlezun DJ, Beck D, Kann B, Vargas HD, Whitlow C, Margolin D (2017) The Role of Bowel Preparation in Colorectal Surgery: Results of the 2012-2015 ACS-NSQIP Data. Ann Surg. doi:10.1097/SLA.0000000000002568

19. Tocchi A, Mazzoni G, Fornasari V, Miccini M, Daddi G, Tagliacozzo S (2001) Preservation of the inferior mesenteric artery in colorectal resection for complicated diverticular disease. Am J Surg 182 (2):162-167

20. Cirocchi R, Trastulli S, Farinella E, Desiderio J, Listorti C, Parisi A, Noya G, Boselli C (2012) Is inferior mesenteric artery ligation during sigmoid colectomy for diverticular disease associated with increased anastomotic leakage? A meta-analysis of randomized and non-randomized clinical trials. Colorectal Dis 14 (9):e521-529. doi:10.1111/j.1463-1318.2012.03103.x

21. Schlussel AT, Wiseman JT, Kelly JF, Davids JS, Maykel JA, Sturrock PR, Sweeney WB, Alavi K (2017) Location is everything: The role of splenic flexure mobilization during colon resection for diverticulitis. Int J Surg 40:124-129

22. Thaler K, Baig MK, Berho M, Weiss EG, Nogueras JJ, Arnaud JP, Wexner SD, Bergamaschi R (2003) Determinants of recurrence after sigmoid resection for uncomplicated diverticulitis. Dis Colon Rectum 46 (3):385-388. doi:10.1097/01.DCR.0000054638.76404.3B

23. Bergamaschi R, Arnaud JP (1998) Anastomosis level and specimen length in surgery for uncomplicated diverticulitis of the sigmoid. Surg Endosc 12 (9):1149-1151

24. Sielezneff I, Malouf AJ, Pirro N, Cesari J, Brunet C, Sastre B (2001) Short-term functional outcome following elective surgery for complicated sigmoid diverticular disease: sutured or stapled end-to-end anastomosis to the proximal rectum? Colorectal Dis 3 (1):23-27

25. Ricciardi R, Roberts PL, Marcello PW, Hall JF, Read TE, Schoetz DJ (2009) Anastomotic leak testing after colorectal resection: what are the data? Arch Surg 144 (5):407-411; discussion 411-402. doi:10.1001/archsurg.2009.43

26. Kamal T, Pai A, Velchuru VR, Zawadzki M, Park JJ, Marecik SJ, Abcarian H, Prasad LM (2015) Should anastomotic assessment with flexible sigmoidoscopy be routine following laparoscopic restorative left colorectal resection? Colorectal Dis 17 (2):160-164. doi:10.1111/codi.12809

27. Zhang HY, Zhao CL, Xie J, Ye YW, Sun JF, Ding ZH, Xu HN, Ding L (2016) To drain or not to drain in colorectal anastomosis: a meta-analysis. Int J Colorectal Dis 31 (5):951-960. doi:10.1007/s00384-016-2509-6

28. Menahem B, Vallois A, Alves A, Lubrano J (2017) Prophylactic pelvic drainage after rectal resection with extraperitoneal anastomosis: is it worthwhile? A meta-analysis of randomized controlled trials. Int J Colorectal Dis 32 (11):1531-1538. doi:10.1007/s00384-017-2891-8

29. Schlachta CM, Mamazza J, Poulin EC (1999) Laparoscopic sigmoid resection for acute and chronic diverticulitis. An outcomes comparison with laparoscopic resection for nondiverticular disease. Surg Endosc 13 (7):649-653

30. van de Wall BJM, Stam MAW, Draaisma WA, Stellato R, Bemelman WA, Boermeester MA, Broeders I, Belgers EJ, Toorenvliet BR, Prins HA, Consten ECJ, collaborators Dt (2017) Surgery versus conservative management for recurrent and ongoing left-sided diverticulitis (DIRECT trial): an open-label, multicentre, randomised controlled trial. Lancet Gastroenterol Hepatol 2 (1):13-22. doi:10.1016/S2468-1253(16)30109-1

31. Andeweg CS, Berg R, Staal JB, ten Broek RP, van Goor H (2016) Patient-reported Outcomes After Conservative or Surgical Management of Recurrent and Chronic Complaints of Diverticulitis: Systematic Review and Meta-analysis. Clin Gastroenterol Hepatol 14 (2):183-190. doi:10.1016/j.cgh.2015.08.020

32. Klarenbeek BR, Bergamaschi R, Veenhof AA, van der Peet DL, van den Broek WT, de Lange ES, Bemelman WA, Heres P, Lacy AM, Cuesta MA (2011) Laparoscopic versus open sigmoid resection for diverticular disease: follow-up assessment of the randomized control Sigma trial. Surg Endosc 25 (4):1121-1126. doi:10.1007/s00464-010-1327-0

33. Tuech JJ, Regenet N, Hennekinne S, Pessaux P, Bergamaschi R, Arnaud JP (2001) Laparoscopic colectomy for sigmoid diverticulitis in obese and nonobese patients: a prospective comparative study. Surg Endosc 15 (12):1427-1430

34. Tuech JJ, Pessaux P, Regenet N, Rouge C, Hennekinne S, Bergamaschi R, Arnaud JP (2001) Laparoscopic colectomy for sigmoid diverticulitis: a prospective study in the elderly. Hepatogastroenterology 48 (40):1045-1047

35. Reshef A, Stocchi L, Kiran RP, Flechner S, Budev M, Quintini C, Remzi FH (2012) Case-matched comparison of perioperative outcomes after surgical treatment of sigmoid diverticulitis in solid organ transplant recipients versus immunocompetent patients. Colorectal Dis 14 (12):1546-1552

36. Samdani T, Pieracci FM, Eachempati SR, Benarroch-Gampel J, Weiss A, Pietanza MC, Barie PS, Nash GM (2014) Colonic diverticulitis in chemotherapy patients: should operative indications change? A retrospective cohort study. Int J Surg 12 (12):1489-1494. doi:10.1016/j.ijsu.2014.10.032

37. Al-Khamis A, Abou Khalil J, Demian M, Morin N, Vasilevsky CA, Gordon PH, Boutros M (2016) Sigmoid Colectomy for Acute Diverticulitis in Immunosuppressed vs Immunocompetent Patients: Outcomes From the ACS-NSQIP Database. Dis Colon Rectum 59 (2):101-109. doi:10.1097/DCR.0000000000000513
